# A comprehensive review of flow chemistry techniques tailored to the flavours and fragrances industries

**DOI:** 10.3762/bjoc.17.90

**Published:** 2021-05-18

**Authors:** Guido Gambacorta, James S Sharley, Ian R Baxendale

**Affiliations:** 1 Department of Chemistry, University of Durham, Stockton Road, Durham, DH1 3LE, United Kingdom

**Keywords:** flavours and fragrances, flow chemistry, process chemistry, synthesis

## Abstract

Due to their intrinsic physical properties, which includes being able to perform as volatile liquids at room and biological temperatures, fragrance ingredients/intermediates make ideal candidates for continuous-flow manufacturing. This review highlights the potential crossover between a multibillion dollar industry and the flourishing sub-field of flow chemistry evolving within the discipline of organic synthesis. This is illustrated through selected examples of industrially important transformations specific to the fragrances and flavours industry and by highlighting the advantages of conducting these transformations by using a flow approach. This review is designed to be a compendium of techniques and apparatus already published in the chemical and engineering literature which would constitute a known solution or inspiration for commonly encountered procedures in the manufacture of fragrance and flavour chemicals.

## Introduction

### The fragrance industry

**Figure 1:**
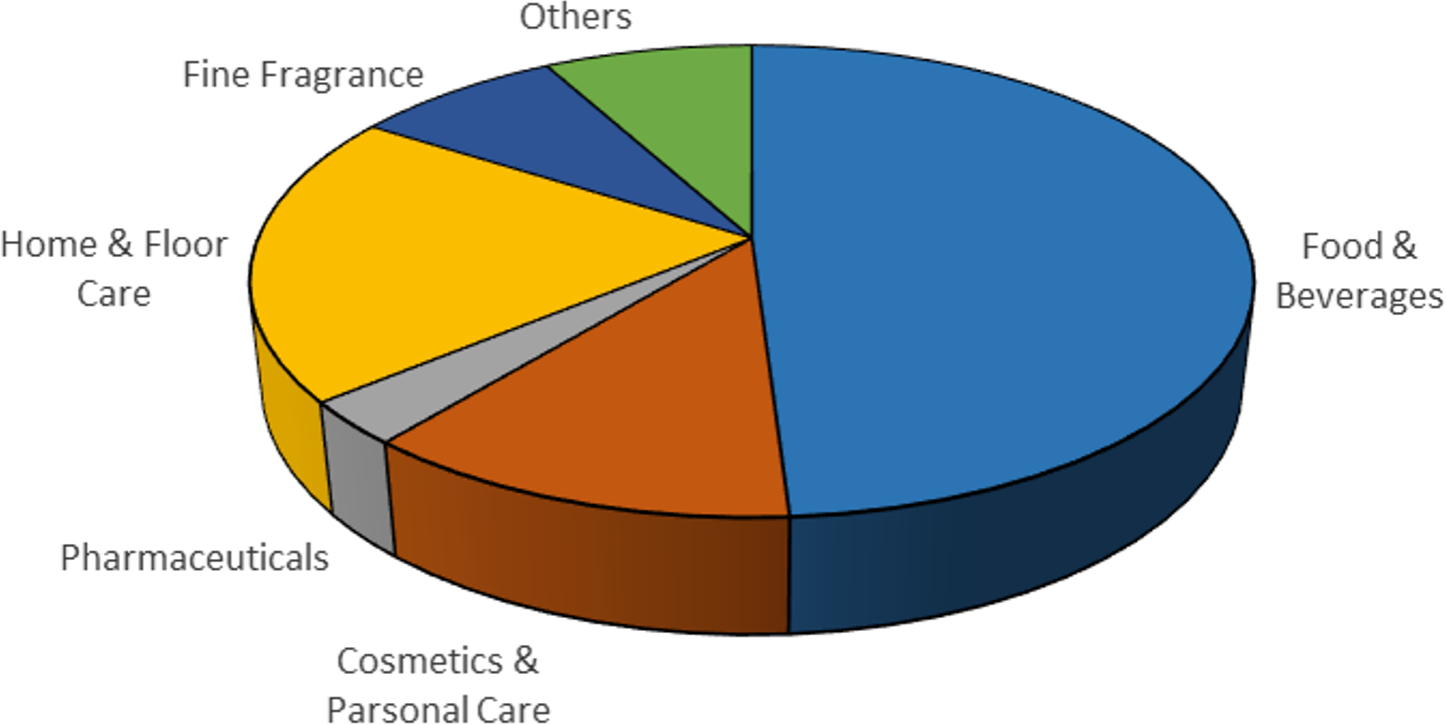
Representative shares of the global F&F market (2018) segmented on their applications [1].

In 2018, the flavours and fragrances (F&F) market was valued $26.5 billion with an annual growth of 4.8%, this industry represents a strong economic market expected to be worth more than $38.5 billion by 2026 in the rapidly expanding global market [1]. In general, there is an approximate equal split in sales value between the fragrance and flavour sectors with synthetic fragrance ingredients contributing a large proportion of these sales (Figure 1). This, therefore, represents a multibillion dollar outlet for synthetic organic chemistry, creating an arena in which chemists are constantly striving to deliver cheaper and more environmentally friendly processes.

**Figure 2:**
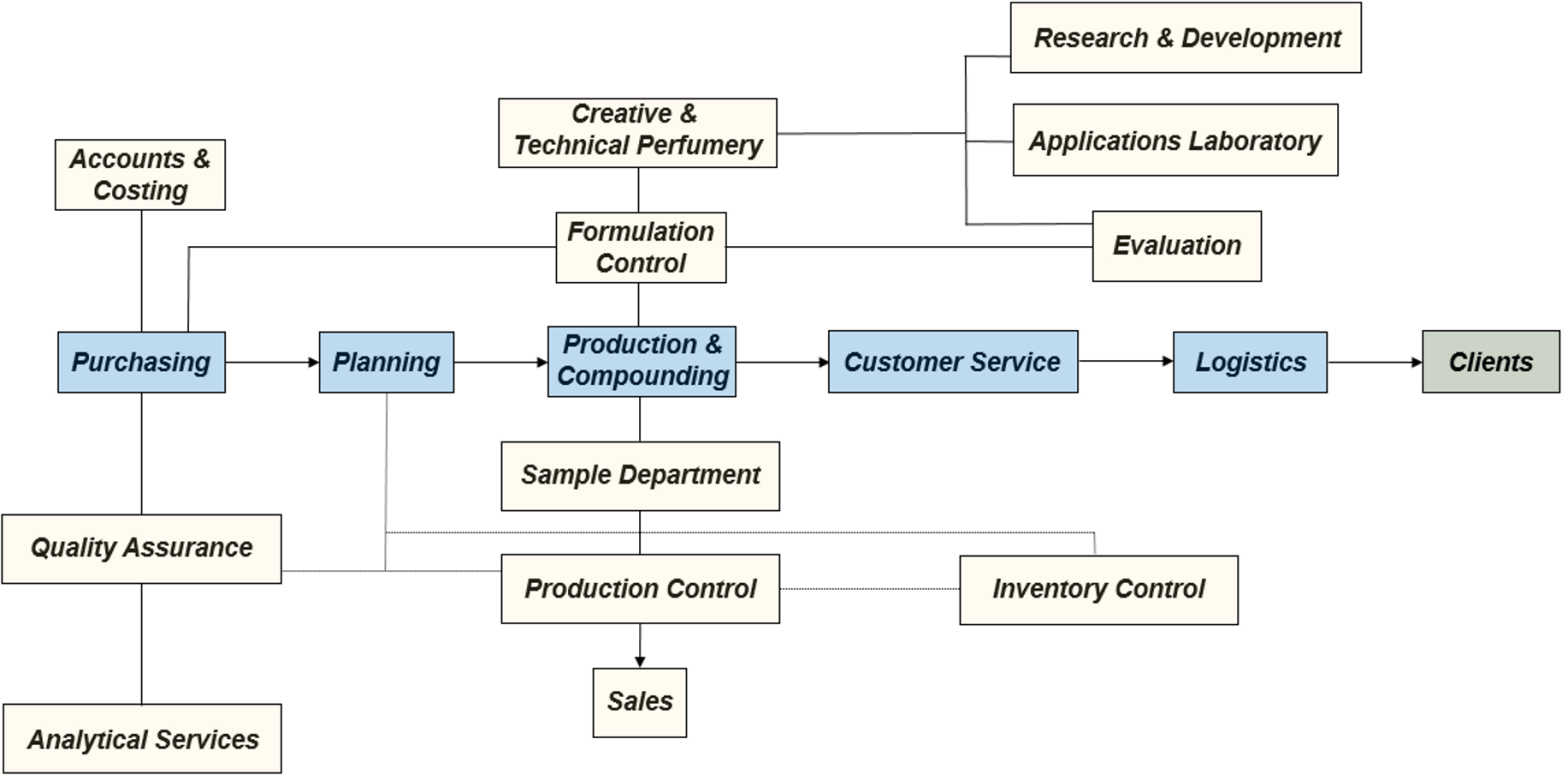
General structure of an international fragrance company [2].

All commercially available products comprised of fragrance ingredients on the market can be traced back to the innovation of fragrance companies as their manufactured products (both of natural and synthetic origin) are sold on to companies further down the supply chain for formulation, packaging and distribution. International fragrance companies are mainly responsible for the discovery, creation, process development and manufacturing of synthetic fragrance ingredients with all of these representing active areas of current research activity within the field today (Figure 2).

### Flow chemistry

Batch-based synthetic methods have enabled a wide variety of transformations to be performed at industrial scales, however, the implementation of flow-based protocols often lends itself to the creation of superior synthetic systems. Improved processing safety (lowered inventory, improved control of heat/mass transfer), enhanced process diagnostics (PAT, inventory management), upgraded yields and selectivity (improved mixing and energetics) as well as allowing access to expanded processing windows (greater temperature, pressure and reaction time domains) means flow is becoming a popular choice. While recent efforts within the flow chemistry community have been mainly geared towards the use of continuous systems for small/research scale chemistry, there is growing interest in the translation of such capabilities to larger/commercial scale syntheses [3–7].

#### Fragrance chemistry

Since the earliest documented use of fragrances in ancient Egypt and Mesopotamia to current times, the human race has always been fascinated by the manipulation and enhancement of scents. Although nature supplies an abundance of inspiration, the fragrance community has always been apt to try and replicate, or sometimes improve upon the initial resource. One of the first recorded preparations of a synthetic aroma chemical was the reaction of oil of amber with fuming nitric acid, which gave a musky odour as described by scientists at the Berlin Academy in 1759 [8]. By the 20th century, an explosion of new synthetic fragrance discovery had occurred and we now have thousands of different materials, each offering unique and tailored aromas (International FragrancAassociation (IFRA) published a list of active F&F chemicals) [9–10]. Table 1 represents some of the most important synthetic fragrances with their approximate associated prices and tonnages. As can be quickly gleaned a substantial quantity of fragrance materials are manufactured annually. However, some of the listed components are also themselves used to produce other ingredients; for example, myrcene (**506**) is used for the preparation of linalool (**10**), geraniol (**343**), nerol, and 4-(4-Hydroxy-4-methylpentyl)cyclohex-3-ene-1-carbaldehyde (lyral, International Flavors & Fragrance, IFF).

**Table 1:** Some of the most important synthetic fragrance materials [2,11–12].

*Name*	*Tonnes per year Annum^a^*	*£/kg*^a^	*Odour type*

myrcene	30,000^b^	1	balsamic
pine oil/terpineol	30,000	1	pine
menthol	12,000	10	mint/coolant
linalool	10,000	4	floral/wood
citral	5,000	5	lemon^c^
dihydromyrcene	5,000^b^	1	–
geraniol	5,000	4	rose
(methyl)ionones	5,000	15	violet
dihydromyrcenol	4,000	4	citrus/floral
limonene	3,500	1	orange^d,e^
citronellol (**299**)	3,000	7	rose
isobornyl acetate (*S,S,S-***664**)	2,000	1	pine
linalyl acetate	2,000	4	fruity/floral
tetrahydrolinalool	2,000	4	floral
carvone	1,500	10	spearmint
hydroxycitronellal	1,000	8	muguet
lyral (IFF)	1,000	12–23^f^	floral
terpenylcyclohexanols	1,000	4–38^f^	sandalwood
zenolide and analogues	1,300–4,300	60–250*^f^*	musk
civetone and analogues	50–500	8–80^f^	musk
cedrene derivatives	500	20	cedarwood
Amberlyn^®^/Ambrox^®^/Ambroxan^®^	50	500–750	ambergris
bangalol and analogues	30	30	sandalwood

^a^These prices and volumes are estimated. ^b^A substantial proportion of the total consumption is used for manufactured of other ingredients. ^c^Citral has little use in fragrance. The tonnage is used for manufacturing ionones and methylionones. ^d^The material used is actually orange terpenes, which is about 80% limonene but the odour comes from minor components. ^e^This figure relates to the use of orange oil in perfumery. About 1,500 tonnes per annum (tpa) are used in the manufacture of carvone. The total production exceeds 50,000 tpa. ^f^Prices are approximate values from Alibaba.com.

**Figure 3:**
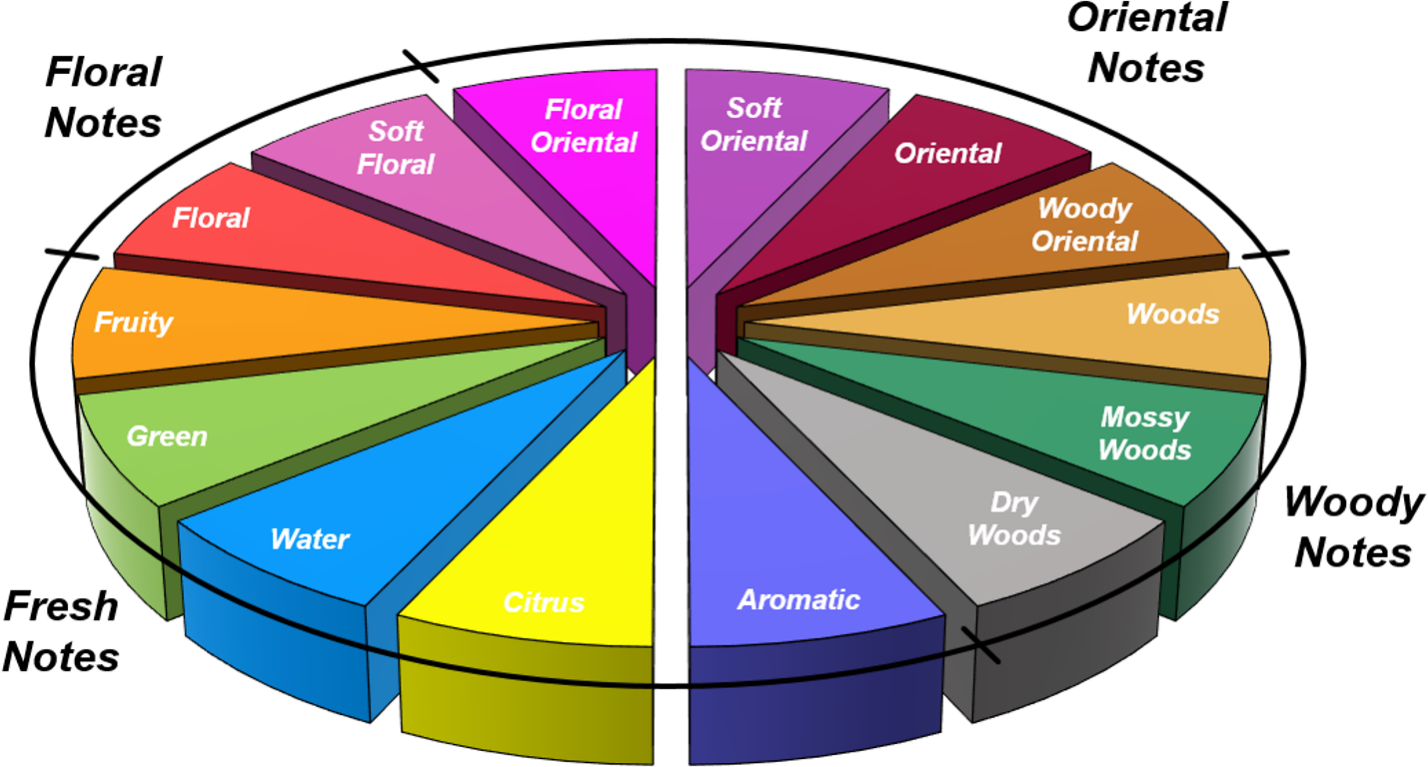
The Michael Edwards fragrance wheel.

The classification of fragrances to instil order and allow comparison of materials has always been a challenge based upon the vagaries and emotive disparity of the human sense of smell. Several approaches have been proposed and over time superseded by improved scientific models. The Michael Edwards Fragrance wheel (Figure 3) was first introduced in 1983 as a perfumery classification system and is divided into the four standard families: floral, oriental, fresh and woody. Until 2010 fougère was also included as a family constituent, however, it has subsequently been integrated as one of the fourteen subclasses (aromatic). While this can be viewed as an overall scent map based upon the experience of a single perfumer, it has been shown to be highly consistent with other studies and approaches from across the scientific literature [13].

**Figure 4:**
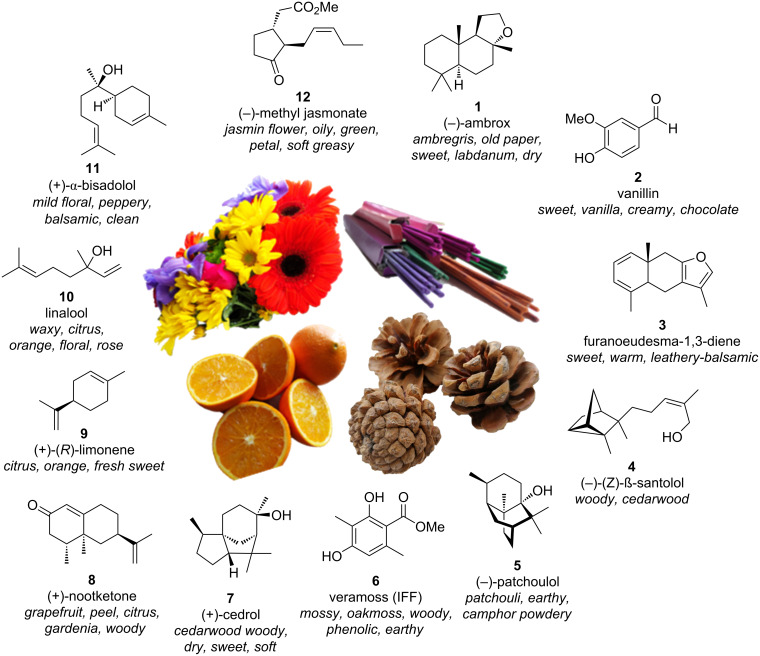
Examples of oriental (**1**–**3**), woody (**4**–**7**), fresh (**8**–**10**), and floral (**11** and **12**) notes.

Each principle note can be further subdivided yielding a related family of scents. For example, the oriental family represent odours that would be associated with amber, incense, vanilla and those possessing spicy and resinous characteristics (i.e., **1**–**3**). Woody notes are often associated with scents such as cedar, patchouli, pine, sandalwood and vetiver, it also includes the more mossy fragrances, for example, oakmoss (i.e., **4**–**7**). Fresh notes are often associated with ozonic or fruity aromas, orange and lemon zest, bergamot and freshly cut grass (i.e., **8**–**10**). The floral note family represents the most popular family with a vast repertoire of aromas mostly derived from plant-based scents (i.e., **11** and **12**). As can be seen the structural diversity, including the range of simplicity and complexity represented by the different scent molecules is impressive (Figure 4).

#### The advantages of a flow approach

Flow chemistry as a branch of organic synthesis has been reviewed extensively through several informative and educational articles [14–21] and books [22–27]. As such our aim is not to include a comprehensive review here but to simply highlight the principle characteristics and direct the reader to the listed references for further consultation.

**Figure 5:**
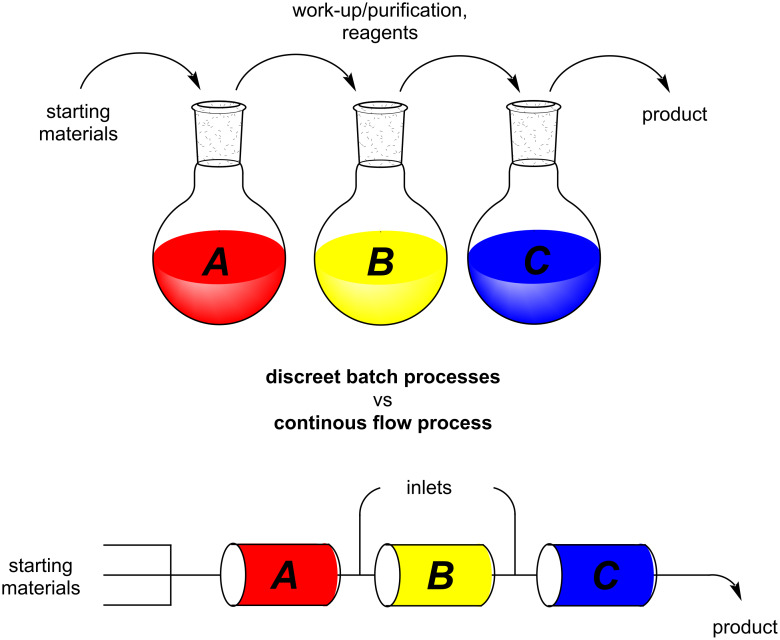
A basic depiction of batch vs flow.

In overview a basic depiction of a sequential batch vs flow approach for a multistep synthesis is shown in Figure 5. In the batch process, multiple discreet steps and reaction vessels are required each often associated with an accompanying work-up stage (and purification).

Industrial syntheses often face lengthy, economically arduous synthetic operations that require an extended labour investment to maintain and operate (requirements for large volumes of solvent during work-ups and cleaning of reactors between processes). Alternatively, in the continuous-flow approach, a fully automated reaction system can be assembled, in which, transformations occur within reactors with a continuous interconnected output. The potential inclusion of downstream inlets allows for further reagent addition and direct telescoping into a subsequent reactor/reaction step. This can in theory be repeated as many times as is desired.

This approach is being increasingly adopted to address synthesis problems across both research and manufacturing setting [28–32]. The massive increase in interest towards flow chemistry throughout the field of organic synthesis has been aided in part by the advent and increasing availability of lab-scale flow systems designed by both academic and industrial sectors. The commercial flow reactors allow for the safe simulation of larger scale processes within the lab environment, facilitating and expediting the process optimisation experience. While some of the implications of the flow chemistry are more obvious than others, i.e., greener processes with potential cost reduction; there are many potential applications of this field in the small scale chemistry as well. For example, areas like combinatorial chemistry [33–35], complex multistep syntheses [14,36–37], synthesis of pharmaceutical substances have witnessed tremendous growth using flow chemistry approach [14,28–29,38–39]. There are many reasons for adopting flow chemistry and continuous manufacturing practices in both industry and academia: Increased levels of automation, inherent health and safety implications due to containment, the implicit higher heat and mass transfer rates, higher temperature and pressure range access, the potential for in-line purification, monitoring and telescoping, linear scalability and more efficient mixing. These applications are very attractive for a synthetic chemist in all settings, especially considering the current ever-increasing industrial pressure to deliver ‘greener’ chemical processes at the lowest possible cost.

The 12 principles of green chemistry were introduced in 1998 by Paul Anastas and John Warner [40] and outline what is meant by and to be expected of a green chemical, process or product. Principles for which flow chemistry may be pertinent are displayed by an upward arrow in Table 2, for others only minor implications can be imagined [41].

**Table 2:** The 12 principles of green chemistry.

	Principle	Flow adherence

1	prevent waste	↑
2	atom economy	↑
3	less hazardous chemical syntheses	↑
4	design benign chemicals	↓
5	benign solvents and auxiliaries	↑
6	design for energy efficiency	↑
7	use of renewable feed stocks	↔
8	reduce derivatives	↔
9	catalysis (vs stoichiometric)	↑
10	design for degradation	↔
11	real-time analysis for pollution prevention	↑
12	inherently benign chemistry for accident prevention	↑

Later, in 2003 Anastas went on to publish the 12 principles of green engineering [42], the relevance of continuous manufacturing to these is displayed in Table 3. Together these clearly demonstrate the compatibility of the flow approach with a green chemical manufacturing future. It is also not difficult to see how continuous manufacturing has implications for important green chemistry metrics such as atom economy, reaction mass efficiency, effective mass yield, carbon efficiency and environmental (E) factor (sometimes referred to as the Sheldon E Factor) [43]. These parameters are an integral part of contemporary process evaluation frameworks such as the SELECT (safety, economics, legal, environmental, control, throughput) criteria and there is an increasing demand to keep these numbers as close to optimal as possible.

**Table 3:** The 12 principles of green engineering.

	Principle	Flow adherence

1	inherent rather than circumstantial	↑
2	prevention instead of treatment	↑
3	design for separation	↑
4	maximize efficiency	↑
5	output-pulled versus input-pushed	↑
6	conserve complexity	↔
7	durability rather than immortality	↔
8	meet need, minimize excess	↑
9	minimize material diversity	↔
10	integrate material and energy flows	↑
11	design for commercial "afterlife"	↔
12	renewable rather than depleting	↔

Certain drawbacks to using a flow-approach must also however be acknowledged. Problems may arise due to quenching requirements, the necessity for solvent switching, concentration limitations, compensating for different encountered reaction kinetics over multiple steps when attempting telescoped reaction sequences, potential requirements for intermediate purification and issues arising due to heterogeneity. Hence, a batch or semi-batch (cascade CSTR’s) approach often offers a more convenient and sometimes superior synthetic approach. Efforts directed towards addressing these drawbacks currently account for a large proportion of the research being conducted within the flow chemistry community. The continuous processing of slurries, for example, is now routinely performed [44–48], also, the use of scavengers (e.g., solid support reagents) [49–51] and devices such as membrane-based continuous separators [52–55] has come a long way in aiding with in-line purification and work-up issues. Table 4 provides a general summary list of advantages and disadvantages of a flow approach.

**Table 4:** Flow approach advantage and disadvantages.

Advantages	Disadvantages

• increased levels of automation	• quenching/work-up issues
• high heat (surface-to-volume ratio) and mass transfer rates	• dilution effects of additional downstream flow streams
• potential for in-line purification and telescoping	• solvent limitations for multistep procedures
• compatibility with “*forbidden chemistries*”	• inability to compensate for reaction kinetics
• efficient mixing	• start-up and shut-down procedures
• linear scalability and high throughput	• issues with heterogeneity
• health and safety implications	• higher training and implementation requirements
• facile access to high temperatures and pressures	

There are multiple possible configuration and module combinations of mixing elements, reactor residence coils, heating/cooling segments and additional downstream components such as phase separators, quenching stages and crystallisers. A typical flow reactor will comprise two or more pumps (isocratic/peristaltic/syringe) that feed an HPLC manifold (into which samples may be injected). Separate reagent streams then meet in a variety of ways, the simplest being within a T- or Y-piece. This method of sample stream merging has been demonstrated to give rise to far more efficient mixing than in the case of standard batch reactors, particularly with microreactors (lateral dimensions < 1 mm). In batch, inhomogeneities due to poor mixing can lead to convective dead zones, giving rise to concentration gradients and hot spots. Flow microreactors allow for rapid mixing on timescales in the order of 100 μs and much more efficient heat transfer is made possible by their high surface-to-volume ratios, typically 30,000 m^2^ m^−3^, compared to 100 and 4 m^2^ m^−3^ for laboratory beakers and batch reactors, respectively [56]. Turbulent (Reynold’s number (Re) > 4000, index defining the flow pattern of a fluid) flow profiles are highly desirable, however, mixing issues associated with laminar (Re < 2000) flow profiles may be easily ameliorated by the incorporation of devices such as mixing chips and structured static mixers [57]. The resultant mixed stream is then directed into a reactor, of which there are many types, these allow for anything from increased simple residence time to active stimulation by application of variable temperature zones, light or microwave irradiation, electrolysis and even high frequency sonication.

The ability to quickly and easily assemble and modify a flow setup allows not only the chemical reaction to be performed as a continuous dynamic flow process but also for direct diagnostics to be obtained facilitating rapid analysis of the content and assessment of the extent of reaction in real-time. Several in-line monitoring tools, for example, ReactIR [58–62] and flow based NMR [63–66] which allow for substrate specific, non-consuming analysis, have also been developed. Other in-line techniques employing simple UV [67–70], MS [71–73], and Raman [74–77] analysis have also been successfully applied enabling efficient optimisation and generation of new reaction chemistry.

Flow-based chemical processing has elevated other areas of chemistry improving performance and utility, for example, reinvigorating the area of solid-supported reagents [78]. The incorporation of solid reagents is facilitated in flow through the use of packed bed columns and channels. Consequently, the filtration necessary as part of a batch process becomes inherently gratuitous, recycling and regeneration also become easier in flow as reactivation flow streams can be directed through the reactors. There are several other adventitious benefits of using solid-supported reagents in flow, for example, a “local excess” at the entry point of a column reactor is, in certain instances, indicated to give rise to superior reaction kinetics [79]. It is also easy to create staged temperature gradients to compensate for changes in reaction kinetics based upon concentration or complicating effects such as product derived auto-catalysis. The use of immobilised reagents has also greatly aided in the development of impressive sequential multistep sequences in flow by acting as direct in-line quenching, work-up and purification steps [28,80–85]. Indeed, the scavenger approach to removing excess solution phase reagents or byproducts has had a significant impact on the use and ultimately the successful adoption of flow chemistry into many research laboratories.

Another inherent benefit of flow is the option of safely using solvents at temperatures significantly above of their standard boiling points, increasingly important for process intensification. The implicit high pressure self-contained environment created by a pumped flow system allows for superior reaction kinetics often achieved by elevating the working temperature of the system. This capability also opens up the opportunity for the integration of volatile and gaseous reagents into flow [86]. Due to the inherently low active volumes of reactants that are processed in the reaction zone within a continuous-flow reactor, the safety risks associated with critical event are greatly diminished. In addition, process reliance and sustainability are further improved by adding extra safety monitoring features such as pressure sensors that can both detect, assess, and if necessary, invoke emergency venting or diversion of material to auxiliary depressurisation stages, often without needing a full reactor shut down.

**Scheme 1:**
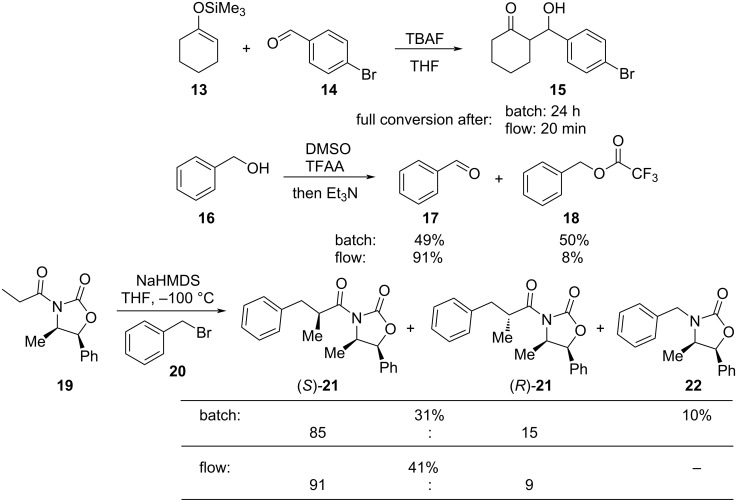
Examples of reactions for which flow processing outperforms batch.

There are numerous examples within the literature in which a flow synthesis has been demonstrated to yield superior results to batch. Higher yields and rates of reaction, and greater selectivities can often be achieved in flow. For example, the rate of the aldol reaction of a silyl enol ether **13** with 4-bromobenzaldehyde (**14**) showed a marked increase upon transposition to flow [87]. The reaction that took 24 hours to run to completion in batch, reached 100% conversion after just 20 minutes in flow (Scheme 1).

The Swern oxidation under trifluoroacetic acid-promoted conditions is often problematic due the formation of side products. In batch, benzaldehyde (**17**) was formed in 49% from benzyl alcohol (**16**), alongside 50% of the trifluoroacetate **18**. Carrying out the same process via a flow-based protocol allowed for the formation of benzaldehyde (**17**) in 91% yield with only 8% of the accompanying side product **18** [88]. Similarly, a stereoselective alkylation of an Evans’ type auxiliary yielded superior results in flow [89]. Alkylation of oxazolidinone **19** with benzyl bromide (**20**) in batch gave only a combined 31% yield of the benzylated products **21**, with a 70% diastereomeric excess (de), accompanied by 10% decomposition to the *N*-benzyl derivative **22**. In flow, however, decomposition was completely avoided and an improved yield of 41% was achieved with an associated increased of the de to 82%.

Whilst the observation of improved processing control under flow conditions compared to batch is not necessarily always the case, there are many instances in which other advantages can be gained by adopting a flow approach. Many of these improvements are inherently linked to the physical engineering of the flow system which are designed to yield improved mixing, better heat transfer and a more structured and consistent processing environment (equating to constant dosing and mixing rates, defined and reproducible reaction timing and exacting control over all processing parameters). Flow chemistry therefore offers a powerful tool for chemists both from a research perspective and later in terms of manufacturing capacity. This has already been widely demonstrated by the pharmaceutical and agrochemical industries where the uptake of flow chemistry has been significant and widely impactful [30]. In the coming years we will undoubtedly see increasing adoption across the entire field of organic synthesis representing a wider spectrum of related industry sectors.

## Review

### Important transformations for the fragrance industry

The following section has been divided according to reaction types that are identified as having particular importance to the fragrance and flavours industry. Within each sub-section, an insight into recent developments and the related literature regarding the performance of these reaction types as a continuous-flow process is provided.

#### Condensation reactions

A carbonyl functionality is encountered in the vast majority of fragrance ingredients. The ability therefore to manipulate carbonyl moieties within a molecule will forever serve as an important transformation to fragrance chemists. This section investigates the aldol reaction as this is a vital C–C and C=C bond forming tool within this class of reactions. Other sequences such as the Knoevenagel and Darzens condensation, however, will also be considered as part of the evaluation.

**Scheme 2:**
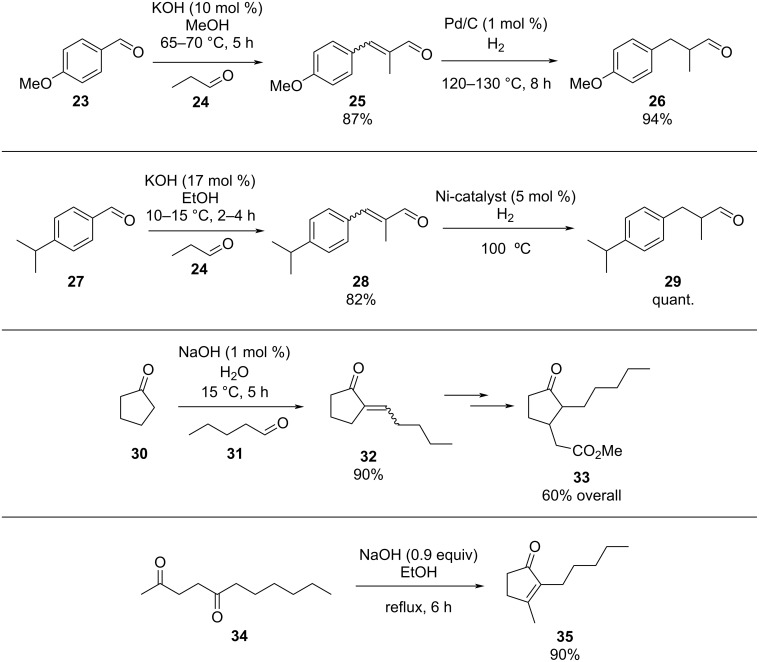
Some industrially important aldol-based transformations.

The condensation of benzaldehyde derivatives, such as *p-*anisaldehyde (**23**) and cuminic aldehyde (**27**), with propanaldehyde (**24**) are important transformations for the preparation of the corresponding anisylpropanal **26**, and cyclamen aldehyde (**29**) (Scheme 2) [90–91].

The syntheses of jasmone derivatives also represent important fragrances whose production involve aldol reactions as key steps. Chapuis et al. have described, several methods for the synthesis of methyl dihydrojasmonate (**33**) and other α*-*substituted cyclopentanone analogues [92], in addition they also performed α*-*functionalisation of the cyclopentanone ring via an aldol reaction [93]. A common synthesis of dihydrojasmone (**35**) uses an intramolecular aldol reaction to simultaneously form the cyclopentanone ring and the double bond in the correct positions [94–95].

**Scheme 3:**
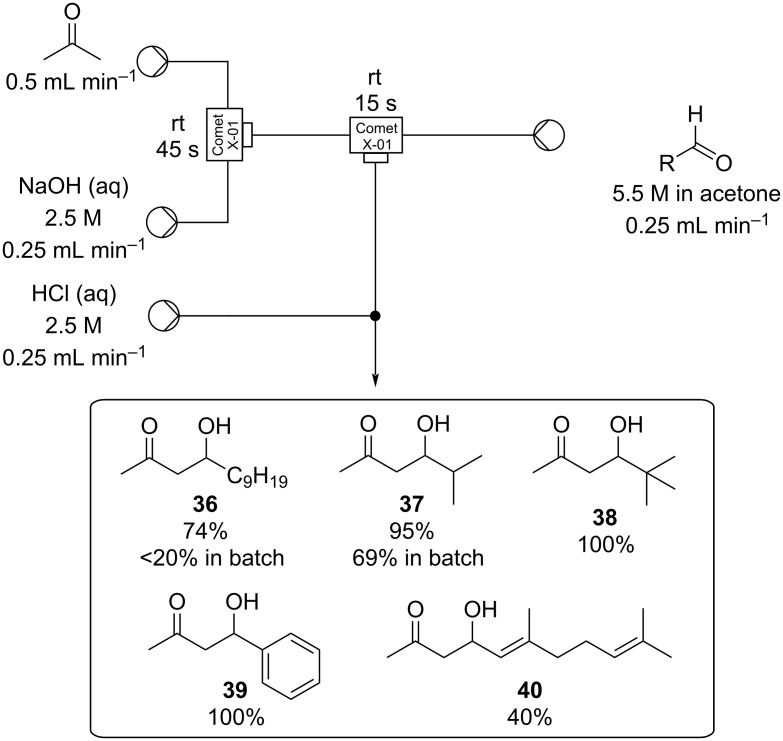
Biphasic continuous aldol reactions of acetone and various aldehydes.

Aldol reactions are most often exothermic, especially those involving highly reactive aldehydes (e.g., benzaldehydes) and low-molecular-weight enolate nucleophiles, therefore, temperature regulation is vital for selectivity. This requirement for strict temperature control makes aldol reactions highly suited to flow processing conditions. In 2008, Tanaka et al. [96] disclosed several examples of aldol reaction in flow reacting acetone with a wide range of aldehydes. In batch, acetone-based aldol reactions are typically performed under biphasic conditions by slow addition of the aldehyde to an acetone/NaOH mixture kept at low temperatures. Upon scale-up, this carries with it problems such as inefficient mixing, difficulty in maintaining the reaction temperature and the occurrence of greater amounts of acetone and aldehyde self-condensation/polymerisation. By adopting a flow protocol and using a Comet X-01 micromixer (with linear scalability), more efficient mixing was attained and less aldehyde self-condensation was evident (Scheme 3).

Within the microreactor acetone was first deprotonated by merging with a 2.5 M sodium hydroxide stream at room temperature, then, after a residence time of just 45 seconds, the resultant enolate was introduced to a stream containing the aldehyde as a 5.5 M solution also in acetone. The combined flow stream was subsequently quenched after 15 seconds by a downstream inlet of aqueous HCl afforded the products **36**–**40**. Significantly higher yields were obtained for **36** and **37** compared to batch and the methodology developed was later applied to a large-scale synthesis of pristane, an immunoactivating natural product (substrate **37**) [97–98].

**Scheme 4:**
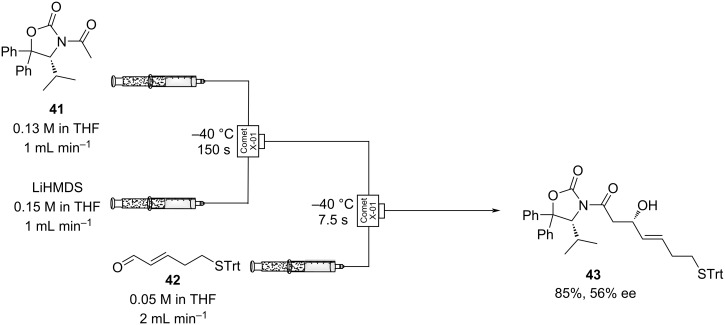
Aldol synthesis of **43** in flow using LiHMDS as the base.

In 2015, the diastereoselective synthesis of (*E*,*S*)-3-hydroxy-7-tritylthio-4-heptenoic acid **43**, a key component of cyclodepsipeptide histone deacetylase (HDAC) inhibitors, was achieved in flow (Scheme 4) [99].

Acetyloxazolidinone **41** was used as a chiral auxiliary which was then removed by hydrolysis in the final step of the overall seven-step synthesis. Deprotonation of **41** using LiHMDS in THF in a primary reactor was performed at −40 °C and telescoped into a second reactor along with a stream containing the trityl-protected aldehyde, **42**. The aldol reaction occurred rapidly (residence time, *t*_R_ = 7.5 s) at −40 °C and furnished **43** in 85% yield, conversion >95%. Simple syringe pumps and cooled micromixers (Comet X-01) were used at both merging points, allowing the reaction to be conducted in very short timescales. Doi’s example also demonstrates the ease with which highly reactive reagents such as lithium bis(trimethylsilyl)amide can be used to effect sensitive aldol reactions in flow.

**Scheme 5:**
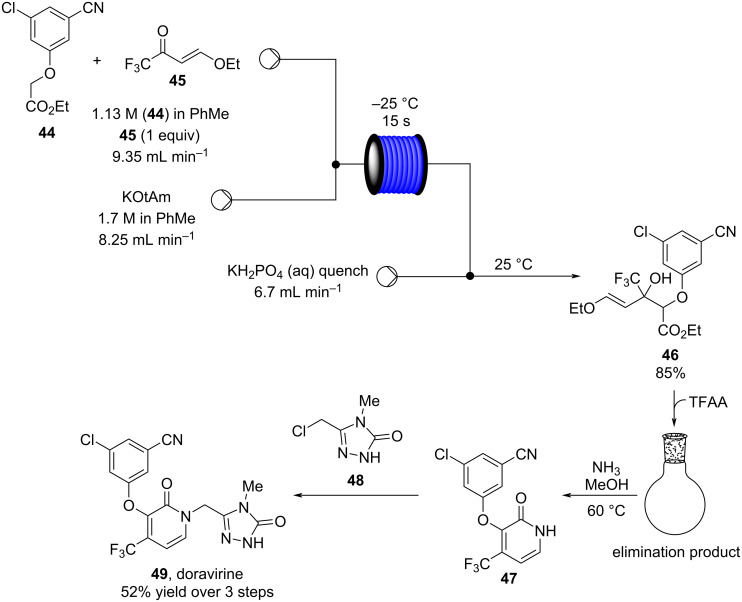
A semi-continuous synthesis of doravirine (**49**) involving a key aldol reaction.

Another recent example of an aldol reaction on a complex system originates from the group of Gauthier, which details the production of the HIV non-nucleoside reverse transcriptase inhibitor (NNRTI) doravirine (**49**) in flow (Scheme 5) [100].

The aldol reaction of an aromatic ester **44** with a vinylogous ester **45** was achieved in a continuous manner, yielding the hydroxyl adduct, **46**, in 85% yield within just a 15 second residence time. Here, a slightly different approach was taken in that the two starting materials were combined into a single stream and the base (potassium *tert*-amyloxide) added via a separate channel. This was presumably established to avoid base-mediated decomposition of the starting material **44** if prepared as a stock solution. The quenched aldol outlet stream was directed into a cooled receiver vessel, to which trifluoroacetic anhydride (TFAA) was simultaneously added, creating a net semi-continuous process. Subsequent ammonia-mediated cyclisation of the resultant diene followed by alkylation with the chlorotriazolinone **48** yielded doravirine (**49**) in an overall 52% yield. The use of an interrupted flow sequence with intermediate batch collection of the output is an easy way of overcoming one of the problematic issues in flow processing; that being assimilating the different residence time requirements in sequential downstream processing. By introducing a batch collection stage this effectively creates a reset on the timing of the individual flow steps. This allows the processing advantages of flow to be utilised for enacting the chemical steps whilst simplifying the timing and connectivity issue. Needless to say, it is inherent to the success of such an approach that there exists at a suitable point in the processing sequence a stable collectable intermediate.

Organocatalysed aldol reactions have become an important tool for the synthesis of chiral molecules as it usually employs cheap and naturally available catalysts such as proline. However, a major drawbacks of these process are the long reaction times and high catalyst loading that often need to be used [101–102].

**Scheme 6:**
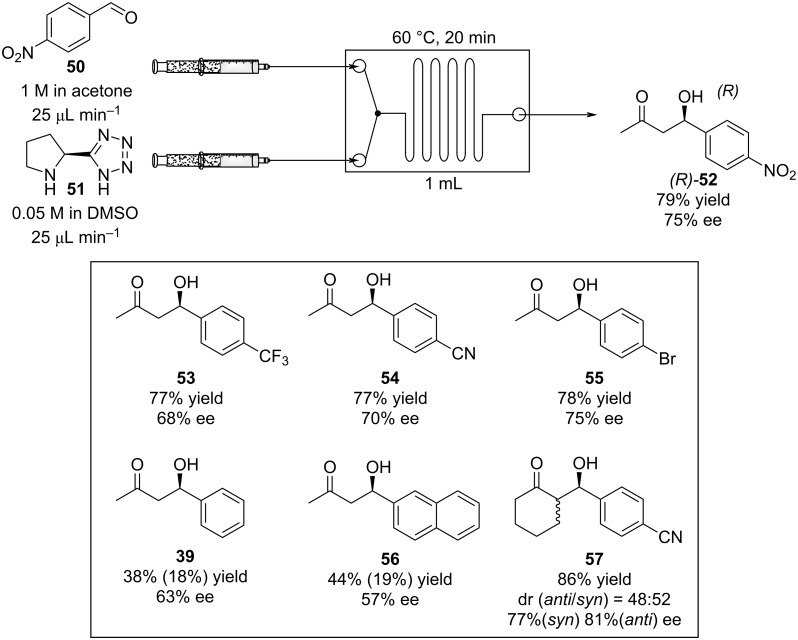
Enantioselective aldol reaction using 5-(pyrrolidin-2-yl)tetrazole (**51**) as catalyst in a microreactor. For compounds **39** and **56** the corresponding dehydrated product yields are presented in parentheses.

In 2009, Seeberger et al. described a version of an enantioselective aldol reaction in flow using a microreactor (Scheme 6) [103]. The approach allowed for a marked reduction in reaction times (20 min vs 40 h) via improved mixing, and the ability to conduct the experiment at higher temperature without detrimental results. They also confirmed the easy scalability of the process from a 1 to 4 mL reactor without loss of selectivity and efficiency. The method was applied on different aldehydes and ketones obtaining moderate-good yields (38–84%). The results obtained for aldehydes **39** and **56** are low as partial dehydration occurs. It was noticed that by reducing the quantity of catalyst less dehydrated product was observed.

**Scheme 7:**
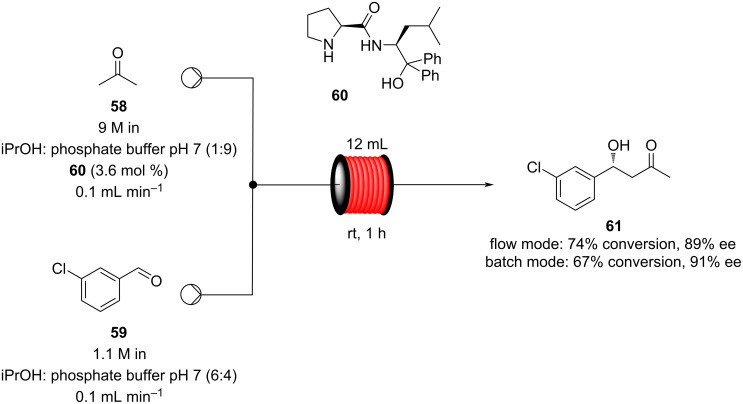
Gröger's example of asymmetric aldol reaction in aqueous media.

A similar approach in aqueous media was optimised by Gröger et al. using a modified proline catalyst. In this example, the authors employed tubular coiled reactors and, to enhance the mixing effect, high flow rates and long tube lengths were applied (Scheme 7). As biocatalysis is becoming more and more important, one of the major issues is the solvent usage. Water is not usually the media of choice to perform organic reactions, therefore, the group of Gröger created a proof-of-concept apparatus capable of subsequently being telescoped directly into a biocatalysed process [104].

**Figure 6:**
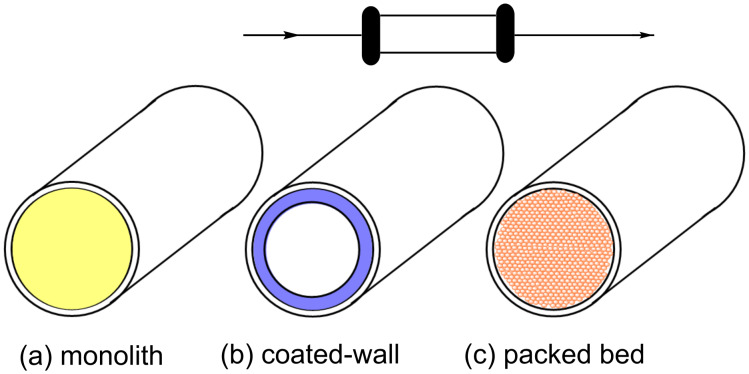
Immobilised reagent column reactor types.

An interesting avenue which can be exploited by carrying out aldol processes in flow is the possibility to incorporate heterogeneous solid-supported reagents as catalysts. The ways in which these are used fall into three main categories; monolithic (a), wall-coated (b) and packed bed (c) reactors (Figure 6) [105].

Monolithic columns are prepared by reagent incorporation into a mixture of monomers which are co-polymerised to create a network of micro- and mesoporous channels that fill the entirety of the tube/column. In a coated wall reactor, the reagent is immobilised onto the inner surface of the reactor. Finally, packed bed reactor columns are typically filled with small beads or particles of the reagents tethered to polymeric or inorganic supports.

**Scheme 8:**
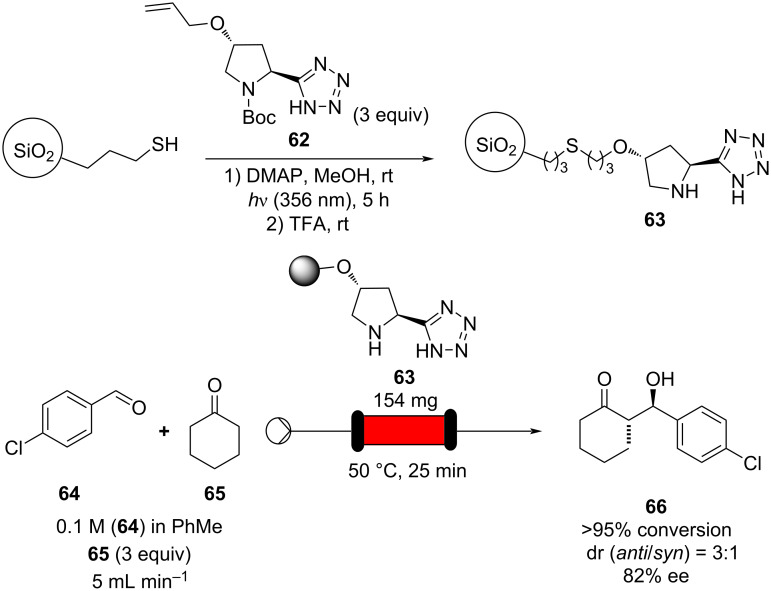
Photoinduced thiol–ene coupling preparation of silica-supported 5-(pyrrolidin-2-yl)tetrazole **63** and its use in the flow aldol reaction of 4-chlorobenzaldehyde (**64**) with cyclohexanone (**65**).

The use of column reactors carries with it many potential advantages. As mentioned previously, the heterogeneous nature of such a system eliminates the requirement for filtration, neutralisation and often a work-up procedure meaning that this approach is very accommodating of direct telescoping into further reaction steps and reactors. So far, heterogeneous catalysis of aldol reactions in flow has been mostly geared towards achieving transformations with high enantioselectivity, towards decreasing the catalyst expense. As enantioselective catalysts require expensive structural modifications, the possibility of immobilisation would facilitate catalyst recovery/recycle and drastically reduce the cost of the associated process. In 2012, silica-supported 5-(pyrrolidin-2-yl)tetrazole **63** was used to effect highly selective aldol reactions of simple ketones and aromatic aldehydes in flow [106]. Photoinduced thiol–ene coupling was used to tether the catalyst onto silica particles, yielding an easily handled powder which was packed into a short stainless steel column (Scheme 8).

The resultant packed-bed microreactor was found to possess good long-term stability and could be used for the catalysis of a range of aldol reactions, one example is given in Scheme 8. Conveniently, just one starting material stock solution was all that was necessary, meaning that 4-chlorobenzaldehyde (**64**) could be introduced to the reactor alongside cyclohexanone (**65**) in a single flow stream. A simple HPLC pump, mild heating (50 °C) and a residence time of 25 minutes was all that was required to give the β*-*hydroxy ketone **66** with >95% conversion, with modest diastereomeric ratio (dr) of 3:1 and high ee (82%).

**Scheme 9:**
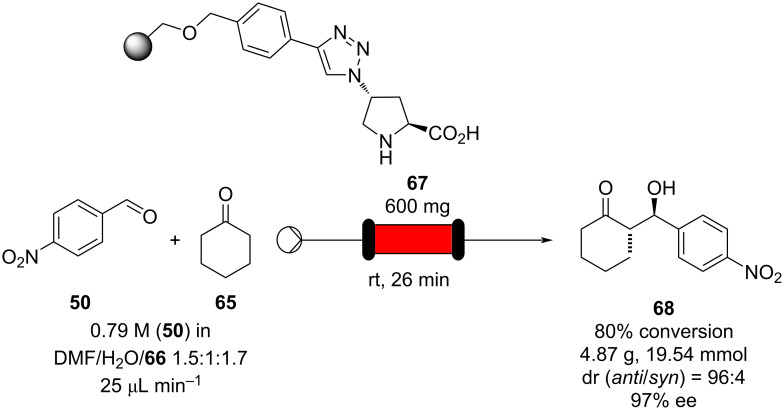
Continuous-flow approach for enantioselective aldol reactions using the supported catalyst **67**.

In the same year, the same group developed a polystyrene resin-supported proline, which was confirmed to be highly suitable for continuous-flow setup whose efficiency and selectivity was highly stable over 30 hours of continuous runtime, allowing to isolate roughly 5 grams of pure aldol **68** (Scheme 9) [107]. The activity and robustness of the catalyst allowed reaction at room temperature over long times and enabled continuous preparation of the desired product with no need for downstream purification.

**Scheme 10:**
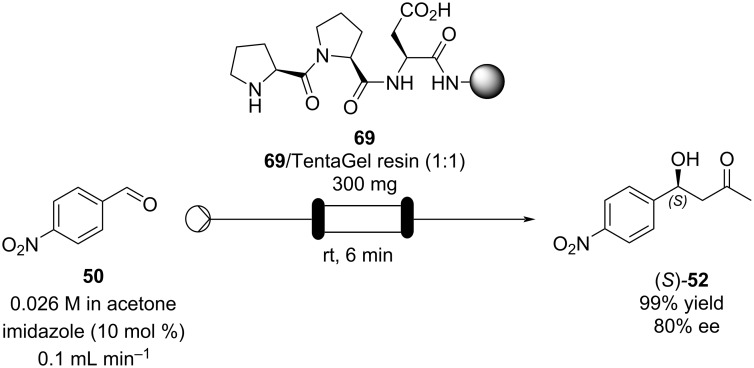
Ötvös’ employment of a solid-supported peptide aldol catalyst in flow.

A similar approach was taken by Ötvös and co-workers in the same year [108]. Asymmetric aldol reactions of aromatic aldehydes with acetone were performed in flow using a solid-supported heterogeneous peptide catalyst. The H-Pro-Pro-Asp-NH-resin **69** which was prepared using Fmoc/*t-*Bu chemistry and assembled onto a TantaGel^®^ polymeric support, then packed into a column reactor (Scheme 10).

Optimisation of the reaction conditions in flow were carried out on *p*-nitrobenzaldehyde (**50**) and resulted in a 99% isolated yield of (*S*)*-***52** (80% ee) with a 6 minutes residence time at room temperature. In an analogous batch experiment, 6 hours were required for the reaction to run to completion and achieve a 94% yield (78% ee).

The optimised conditions were then applied to 12 other aromatic substrates and the column was found to be highly robust, giving consistent results over the course of 20 consecutive experiments (20 × 5 mL starting material solution (4 mg mL^−1^), 50 minutes runtime) (Table 5).

**Table 5:** Outcome of multiple consecutive reaction cycles.

Entry	Cycle	Conversion (%)	Isolated yield (%)	ee (%)

1	1–5	98–100	97–99	79–80
2	6–10	100	97–99	78–81
3	11–15	100	>99	79–80
4	16–20	99–100	98–99	79–80

Conveniently, the (*S*)-enantioselectivity could be reversed by inversion of stereochemistry at the terminal proline moiety of the supported peptide. Ötvös’ protocol highlights some of the key advantages of this immobilisation approach, i.e., improved reaction kinetics over batch (higher effective catalyst loading), column robustness/ease of recyclability and the facile nature of reaction condition and substrate screening.

**Scheme 11:**
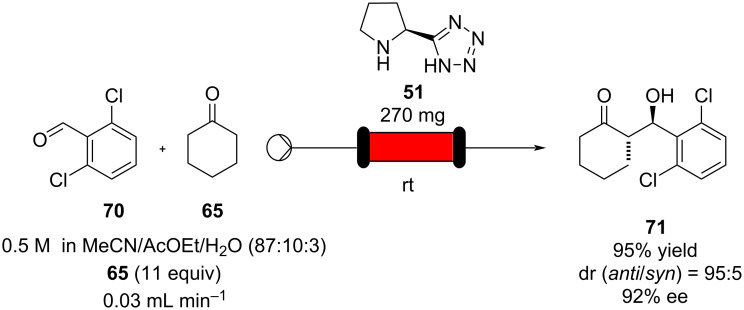
The use of proline tetrazole packed in a column for aldol reaction between cyclohexanone (**65**) and 2,6-dichlorobenzaldehyde (**70**).

To increase the greenness and reduce the consumption of the catalyst, Yamamoto and Nakashima have recently developed a proline tetrazole **51** packed-bed reactor system, exploiting the low solubility of the catalyst in less polar solvents (Scheme 11). Thus, a stream of 2,6-dichlorobenzaldehyde (**70**) and cyclohexanone (**65**) along with 10 mol % of AcOEt and 3 mol % H_2_O were directed as a flow stream into the loaded column to partake in the aldol reaction to furnish adduct **71** in 95% yield and excellent dr and 92% ee. The system allowed a sharp decrease in the loading of the catalyst to 0.052 mg during a 5 mmol scale production run [109].

Yamamoto and Nakashima’s approach is a particularly simple strategy that demonstrates the ease and creativity that can be used in setting up an efficient flow system without the need for supports, hence only modifying the solubility of the catalyst.

**Scheme 12:**
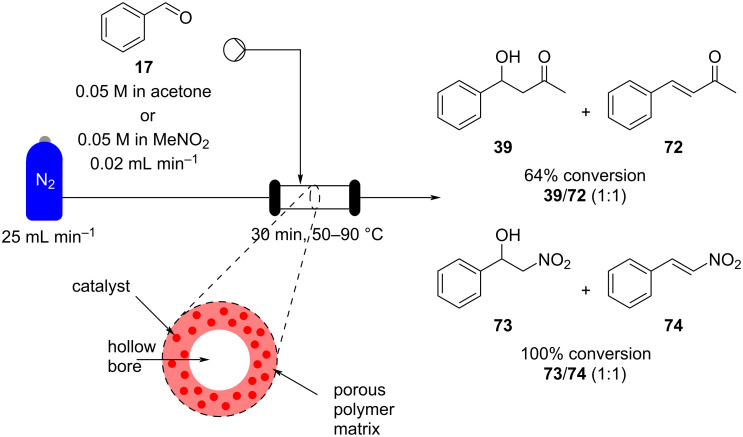
Schematic diagram of an aminosilane-grafted Si-Zr-Ti/PAI-HF reactor for continuous-flow aldol and nitroaldol condensation of benzaldehyde (**17**) with acetone.

However, in the above-described reactors the main drawbacks presented were the high pressure drop along the column and the poor handling of solid particles. This resulted in a reduced reliability especially during scale-up equating to prolonged run times. As a result, alternative support formats have been investigated. In this context there has been a growing interest in the use of functionalised hollow fibres including their application as membranes for gas separation [110] (e.g., CO_2_ absorption [111]) as they offer the advantage of high surface-to-volume ratio and scalability. These systems consist of nanoparticles embedded into a porous polymer matrix. In heterogeneous catalysis, a higher particle stability and good distribution of the catalyst leads to a reduction in pressure drop alongside a stronger interaction with the reagents. As a result, several hollow fibres-immobilised catalysts have been prepared and applied in flow scenarios [112]. A titania-, zirconia- and silica-implanted polyamide-imide (PAI) hollow fibre was grafted with aminosilane functional groups in order to create a bifunctional catalyst for continuous-flow aldol condensations. A solution of the reagents was directed into the stainless steel module, containing a series of five hollow fibres, perpendicular to the fibre alignment. The solution permeates the fibres and is pushed through the reactor using a nitrogen flow stream (Scheme 12) [113].

The reactor output reached a maximum conversion of 64%, generating a 1:1 ratio of the aldol adduct **39** and the further condensation product **72**. It was also shown that the selectivity for **39** could be improved by increase the flow rate, although this also coincided with a lower conversion of just 13%. Nitroaldol reactions were also performed and nitrostyrene (**74**) was achieved with 54% selectivity (1:1 **73**/**74**) and 100% conversion at 90 °C. It is worth noting that the degree of production could be readily increased by numbering up of the hollow fibres which are bundled in the column.

Other examples of the use of column reactors for the catalysis of aldol reactions include: The use of immobilised aldolase enzymes for the synthesis of carbohydrates [114] and the use of a calcinated hydrolactite-packed column for the condensation of furfural with acetone [115], however, this still remains a relatively under-explored area of research. While the majority of reported examples have been of academic origin, there is a high level of interest in the pursuit of aldol reactions in flow by industry. The production of α,β-unsaturated C_10_-aldehydes [116] as multiphase aldol condensations in flow was also patented in 2014 by Strutz et al. [117].

**Scheme 13:**
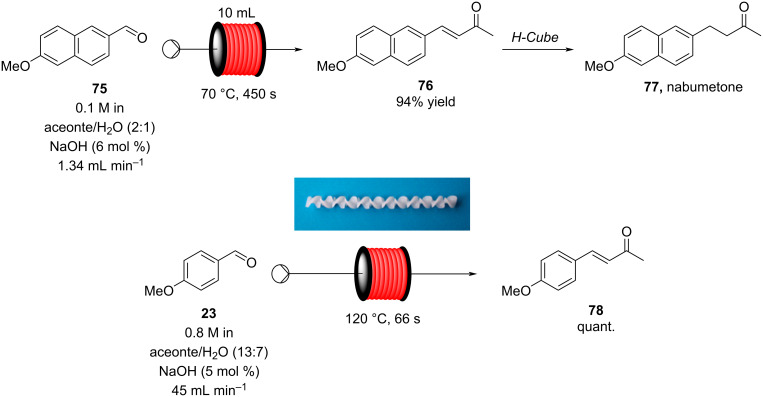
Continuous-flow condensation for the synthesis of the intermediate **76** to nabumetone (**77**) and Microinnova mesofluidic scale-up protocol for the synthesis of **78** [118]. Adapted with permission from [118]. Copyright 2011 American Chemical Society.

Microinnova Engineering has devised a mesofluidic platform for continuous aldol reactions used in the synthesis of nabumetone (**77**) and other 4-aryl-2-butanone derivatives demonstrating the ease of scale-up associated with such transformations in flow [118]. The condensation of a range of aromatic aldehydes with acetone were again studied, with *p*-anisaldehyde (**23**) being used for scale-up. A simple reactor design based upon Teflon^®^ tubing (8 mm inner diameter) was used in conjunction with static mixing elements as shown in Scheme 13, allowing for a flow rate of 45 mL min^−1^ to be used. A 66 second residence time gave full conversion to the α,β-unsaturated product **78** in high isolated yield, generating a calculated theoretical throughput of 0.35 kg of product per hour. The authors claim that, in principle, by simply elongating the reactor by a factor of 5 and increasing proportionally the flow rate a theoretical throughput of 1.75 kg h^−1^ of **78** would be possible. Although such calculations can be made implementing the necessary changes are not as linearly scalable or simple as implied due to change in the mixing efficiency of the reactor as well as effects of back mixing and increased pressure drop, indeed the efficiency of pumps must also be taken into account. The consideration of the pumping device is a critical aspect of each flow process but is often relegated to simple supporting information. However, each pumping unit has by design a specific range of operation and often a very specific window of optimal pumping efficiency (flow rate, pressure drop and viscosity) within this domain. Hence, especially when considering the concepts of direct scale-up this becomes an increasingly important aspect.

Examples of flow systems applied on terpenoids are abundant in the literature. As terpenoids are one of the most important odorants in the F&F industry.

**Scheme 14:**
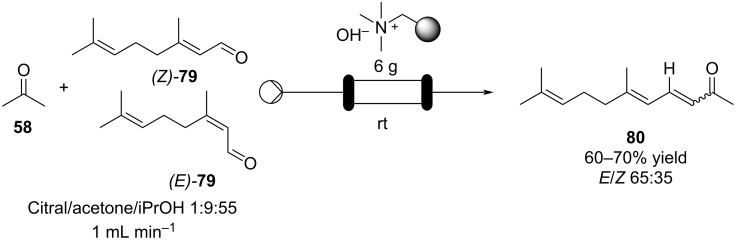
Synthesis of ψ-Ionone (**80**) in continuous-flow via aldol condensation between citral (**79**) and acetone (**58**).

Schütz et al. developed an aldol condensation of citral (**79**, 63:37 mixture of geranial (*Z*)*-***79**/neral (*E*)*-***79**) with acetone to prepare ψ-Ionone or pseudoionone (**80**) in good yield (60–70%) [119]. By using ion-exchange resins such as Amberlyst^®^ A26 (OH form) as a heterogeneous base in a fixed-bed reactor, the α,β-unsaturated compound **80** was obtained and with a stable performance over more than 15 hours of continuous operation (Scheme 14).

In 2019, the group of Kobayashi reoptimised the Schürt’s process for a more general library of molecules (28 compounds were screened) and they managed to overcome the catalyst deactivation enabling a process to be operated continuously for more than 5 days (TOF = 0.09 h^−1^ and STY = 354 g (L day^−1^) using a mixture toluene/EtOH 9:1, noticing that the presence of EtOH drastically increased the catalyst’s activity. A two-step continuous synthesis was also described for the synthesis of donepezil, a top 100 best-selling anti-Alzheimer’s drug [120].

A different approach for the synthesis of ψ-Ionone (**80**) was described by Chen et al., whose group developed an efficient homogenous NaOH-catalysed condensation of citral (**79**) with acetone using microreactors. The setup allowed the preparation of **80** in 93.8% yield with a throughput of 5.24 g h^−1^ [121]. The NaOH aqueous solution was introduced as a mixture with EtOH, which was discovered to notably increase the mass transfer (thus improving yields from 87% to 93.8%). The authors also compared their apparatus with a tubular version described by Dobler et al. claiming a higher yield (93.8 vs 86%) and a faster reaction time (98.6 s vs 120 s) [122].

**Scheme 15:**
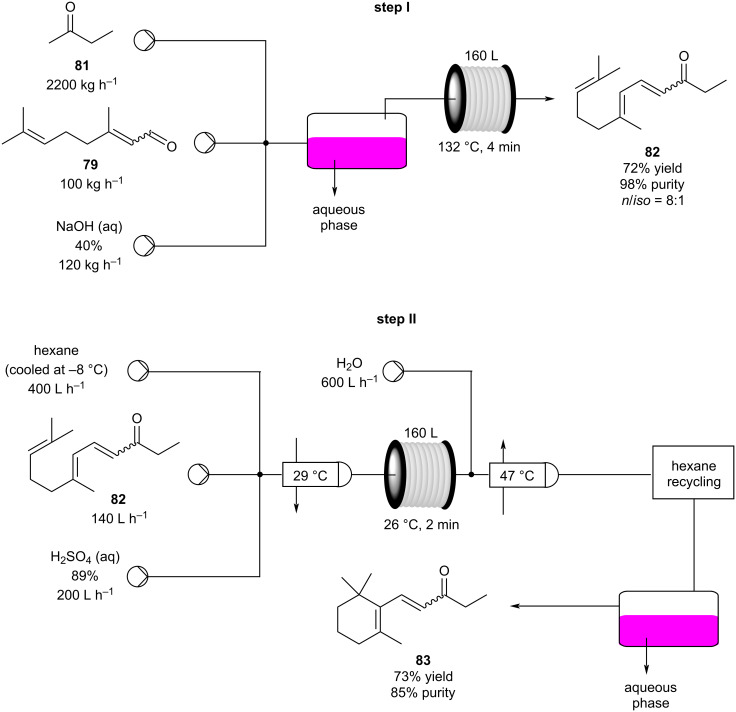
Synthesis of β-methyl-ionones (**83**) from citral (**79**) in flow. The steps are separately described, and no telescope process was claimed.

In the same patent, Dobler et al. described a process for the synthesis of β-methylionones (**83**) in flow. Tubular reactors were employed for the preparation and the two steps were reported separately (Scheme 15). The NaOH-catalysed condensation is performed in a 160 L tubular reactor with a 4 minutes residence time at 132 °C. The initial reaction mixture is biphasic, and a preliminary separation is carried out before entering the reactor coil. After discontinuous purification procedures, the product **82** was yielded in 98% purity (72% yield). The second step, instead, is an acid-catalysed cyclisation which employs the same tubular reactor at lower temperature (26 °C) and reaction time (2 minutes). The cooled solution of **82** in hexane is mixed in flow with sulfuric acid and then heated at 29 °C. After the cyclisation, the stream was quenched with water whilst the temperature was maintained around 45 °C. The hexane was then removed using a countercurrent system with the steam being recycled back through the reactor. After phase separation, the obtained mixture was enriched to 85% of **83** (equating to a 73% isolated yield).

**Scheme 16:**
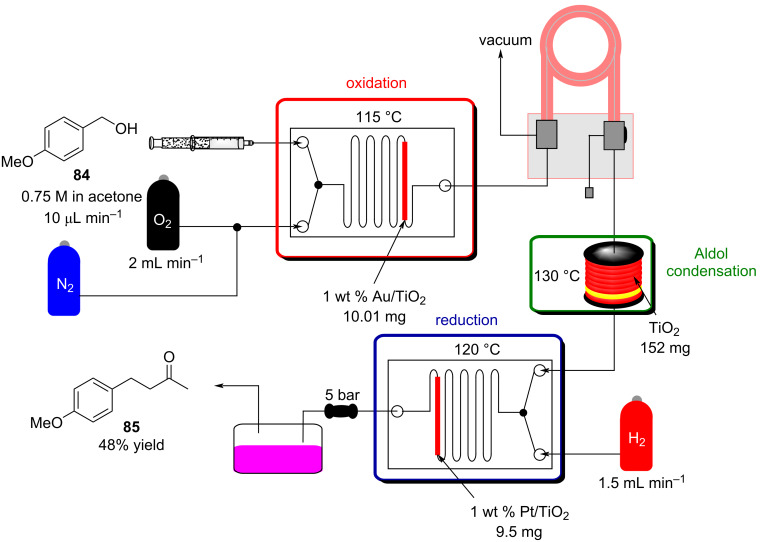
Continuous-flow synthesis of **85** from **84** described by Gavriilidis et al.

Once the reaction conditions were optimised, the apparatus can merge with other flow systems to develop a parallel continuous multistep process. A recent example, Gavriilidis et al. described a heterogeneous version for the synthesis of 4-(4-methoxyphenyl)butan-2-one (**85**) starting from 4-methoxybenzyl alcohol (**84**). In this case, the preparation is divided in 3 stages: oxidation, aldol condensation, and reduction (Scheme 16). The setup employs silicon-glass and tubular reactors packed with nanoparticle supported catalysts. The apparatus allows the desired material to be prepared in 48% yield. The authors pointed out better outcomes were obtained in batch using a MgO-based catalyst, whose usage proved troublesome in flow due to clogging of the reactor channels [123]. This article undoubtedly shows the capacity of flow systems in being easily telescoped for multistep syntheses.

**Scheme 17:**
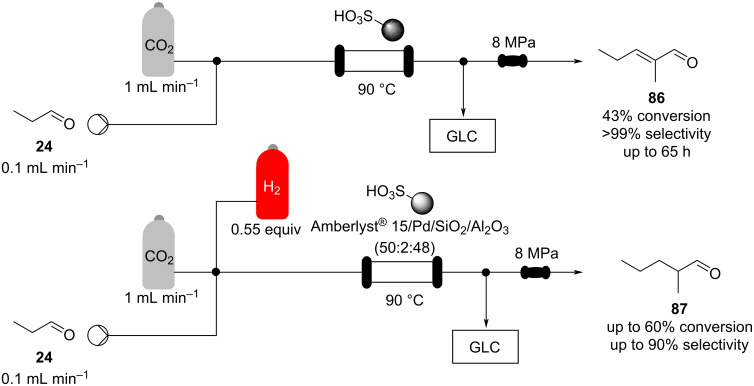
Continuous-flow scCO2 apparatus for the synthesis of 2-methylpentanal (**87**) and the self-condensed using Amberlyst^®^ 15 as a solid catalyst.

A self-condensation for the synthesis of 2-methylpentenal (**86**) was also developed by the group of Poliakoff (Scheme 17). The system exploits a sulfonic acid resin (Amberlyst^®^ 15) in scCO_2_ as the solvent system.

The continuous-flow apparatus had an in-line GC for faster screening of reaction conditions. The optimised setup allows to continuously transform propanal (**24**) in the corresponding self-condensed material **86** in 43% conversion for up to 65 hours. They also proved the possibility to perform an in situ hydrogenation of **86** by mixing the resin with a Pd catalyst (50% Amberlyst^®^ 15/2% Pd on silica/alumina). The desired material was indeed obtained with high yields (up to 80%) and selectivity (up to 95%) [124].

**Scheme 18:**
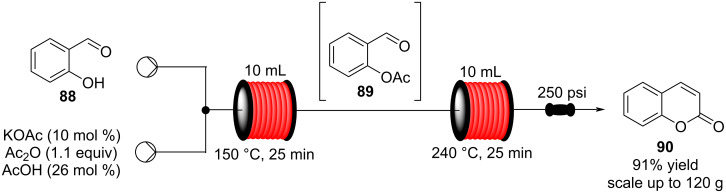
Chen’s two-step flow synthesis of coumarin (**90**).

In 2013, a continuous-flow multistep synthesis of coumarin (**90**) was also reported, which involved an intramolecular aldol-type condensation of the acetylated intermediate **89** derived from salicylaldehyde (**88**, Scheme 18) [125]. The initial *O*-acetylation step was telescoped directly into the next reactor; the two-step process gave an overall yield of 91% under optimal conditions. Beneficially as all components were liquids the need for a solvent was completely avoided; from a green chemistry standpoint this is highly attractive and the intrinsic low melting points of many fragrance ingredients and precursors lend themselves to continuous-flow approaches in this regard. To show the practicability of the process, a 0.12 kg scale-up operation was also performed.

**Scheme 19:**
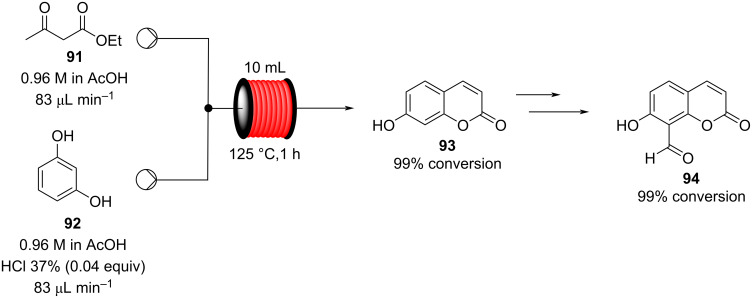
Pechmann condensation for the synthesis of 7-hydroxyxcoumarin (**93**) in flow. The setup extended to comprise 3 additional steps to prepare aldehyde **94**.

A Pechmann condensation in flow for the synthesis of 7-hydroxycoumarin (**93**) was also reported in 2012 (Scheme 19). The single-step process proved highly efficient forming the desired material in essentially quantitative conversion within 1 hour of processing [126]. The flow output was connected to other apparatus to create a continuous multistep process for the preparation of the 8-formyl-7-hydroxycoumarin (**94**).

**Scheme 20:**
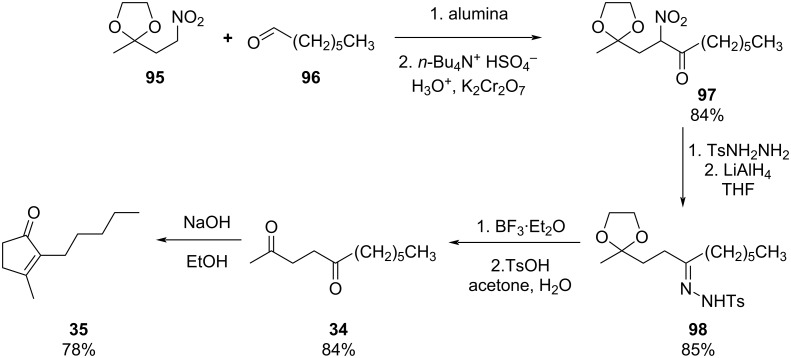
Synthesis of the dihydrojasmonate **35** exploiting nitro derivative proposed by Ballini et al.

The nitroaldol condensation (Henry reaction) is another important direct C–C and C=C bond-forming tool which has been widely described in the literature to prepare a range of different valuable intermediates [127–131]. Applications in the F&F industry are sparse which is probably due to the unpredictable nature of some nitro intermediates. However, late in 1983, Ballini et al. represented the potentiality of the reaction for the synthesis of dihydrojasmone derivatives (Scheme 20) [132].

Flow chemistry embodies the safest conditions for handling hazardous chemicals, therefore opening new opportunities for nitro compounds to be industrially employed.

**Scheme 21:**
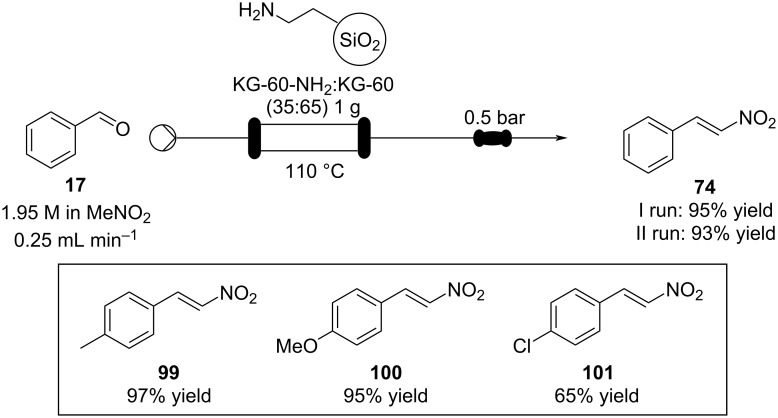
Silica-supported amines as heterogeneous catalyst for nitroaldol condensation in flow.

In 2008, the group of Sartori developed a flow setup for nitroaldol condensations of different benzaldehydes exploiting a silica-supported amine (KG-60-NH_2_) as a heterogeneous catalyst (Scheme 21) [133]. Secondary and tertiary amine-supported catalysts were also investigated, although they were found less active than the primary amine. The authors suggest the reaction occurs through an imine/iminium intermediate as they confirmed a first order relationship between the reaction rate and the catalyst concentration. After washing with nitromethane, the catalyst could be reused a second time, obtaining comparable outcomes.

**Scheme 22:**
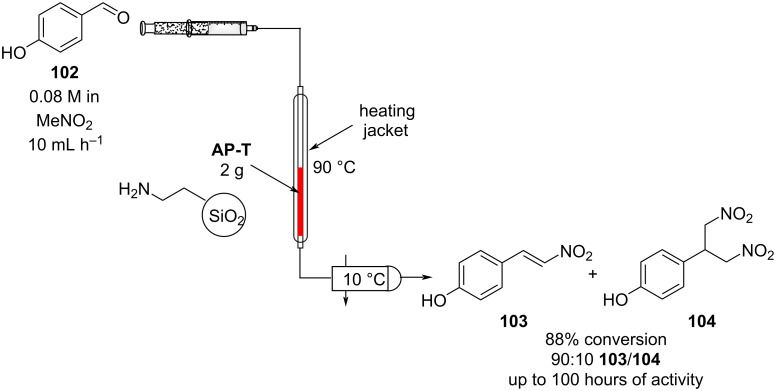
Flow apparatus for the nitroaldol condensation of *p*-hydroxybenzaldehyde (**102**) to nitrostyrene **103** as described by Asefa et al.

A similar system was later described by Asefa et al. The authors packed a jacketed glass capillary microreactor with an amine-functionalised mesoporous silica (AP-T) pressed into small pellets (Scheme 22) [134].

The apparatus enabled the preparation of *p*-hydroxy-β-nitrostyrene (**103**) in good conversion and selectivity. Its synthesis was revealed to be highly dependent on the residence time as lower flow rates brought about the secondary Michael reaction adduct **104**. A secondary amine-functionalised silica was also investigated, although yielding the product in lower selectivity. The optimised setup was shown to be reasonably stable for continuous runtimes of up to 100 hours.

**Scheme 23:**
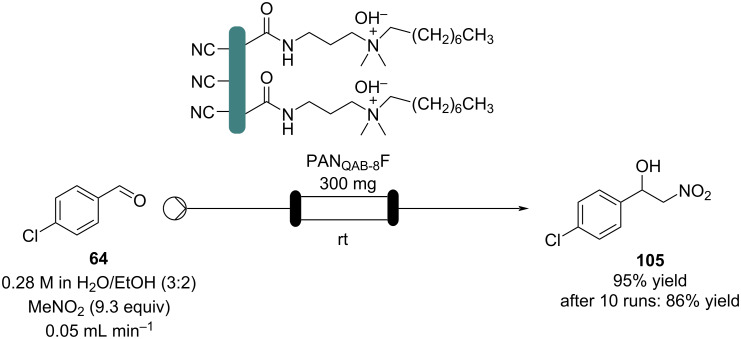
Nitroaldol reaction of **64** to **105** employing a quaternary ammonium functionalised PANF.

In the last few years, polyacrylonitrile fibres (PANF) along with other synthetic textiles have seized attention in heterogeneous catalysis as supports thanks to their incomparable mechanical properties and low-cost [135]. These fibres are also suitable for flow systems as they can be easily shaped into the reactors. As an example, Tao’s group optimised several reactions in batch exploiting a quaternary ammonium functionalised PANF as catalyst (PAN_QAB-8_F). They also reported the Henry reaction of 4-chlorobenzaldehyde (**64**) in flow (Scheme 23) [136]. The catalyst enables the use of water as the solvent; however, in the flow setup, an organic solvent was needed to form a monophasic solution. The apparatus shows high stability (86% yield even after 160 hours of runtime) and reusability (after 10 runs 86% yield).

**Scheme 24:**
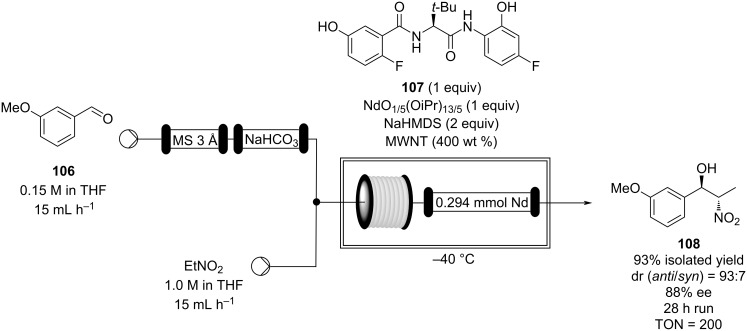
Enantioselective nitroaldol condensation for the synthesis of **108** under flow conditions.

Packed-bed reactors have also been exploited to facilitate enantioselective nitroaldol processes. In 2013, Shibasaki and Kumagai made use of an entangling network of multiwalled carbon nanotubes (MWNT) to trap a Nd/Na bimetallic catalyst, which was shown to be an efficient combination for nitroaldol condensation (Scheme 24) [137].

In 2014, the authors modified their approach and packed the catalyst into a stainless steel column (with 19.5 μmol in Nd) to create a flow system. The catalyst column was cooled (−40 °C) and a stream of aldehyde **106** was first passed through a pre-column containing MS 3 Å and NaHCO_3_ to prevent poising from moisture and acids. Once mixed with the nitroethane, the mixture was directed into the catalyst. The catalyst was found to be highly efficient operating at high conversion (96–91%) for 30 hours (TON = 204) with high enantio- and stereoselectivity (96:4 *anti*/*syn*, 91–92% ee). To confirm the ease of scaling the process, a larger column was prepared (with 0.294 mmol in Nd) and 12.4 g of **108** were obtained (93% yield, 93:7 *anti*/*syn*, 88% ee, TON = 200) after 28 hours [138].

Subsequently, the same group optimised the catalyst preparation using NdCl_3_·6H_2_O and *t-*BuONa, equating to a 120-fold less expensive system than the original employed salts. In this case, the flow system was operated for 24 hours with high conversion and selectivity (90% yield, 20:1 *anti*/*syn*, 90% ee, TON = 145) [139]. The group also optimised the apparatus for the synthesis of other potential useful intermediates [140–141].

**Scheme 25:**
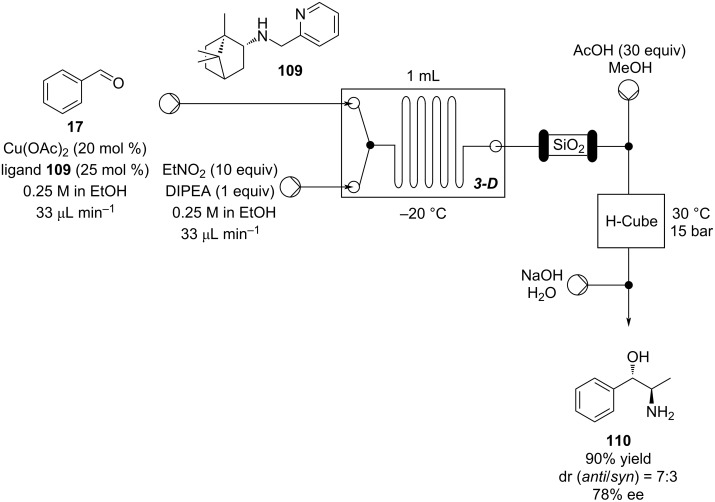
Enatioselective synthesis of 1,2-aminoalcohol **110** via a copper-catalysed nitroaldol condensation.

The introduction of 3D printing devices has aided flow chemistry, especially the prototyping of reactor designs (Scheme 25).

The possibility to quickly investigate several reactors of different volumes, shapes, and materials increases the chance for applicability and improves the understanding of the process itself [142–144]. Benaglia et al. exemplified 3D printing with a copper-catalysed Henry reaction [145]. Benzaldehyde (**17**) and the homogenous catalyst were mixed with nitroethane and the DIPEA base solution in a 1 mL polylactic acid (PLA) square-channelled microreactor. The authors screened several other 3D printed reactors evaluating materials (PTFE, PLA or Nylon), volumes (1 or 10 mL), and channel shape (circular, square, or rectangular). The process was then merged with a hydrogenation step using the H-Cube^®^ apparatus (Pd/C, 30 °C, 15 bar) for the continuous preparation of 1,2-aminoalcohol **110** (90% yield, *anti*/*syn* 7:3, 78% ee). At the end of the Henry reaction an in-line plug of silica gel removes the catalyst before the second reduction step to prevent issues of chelation and palladium deactivation.

**Scheme 26:**
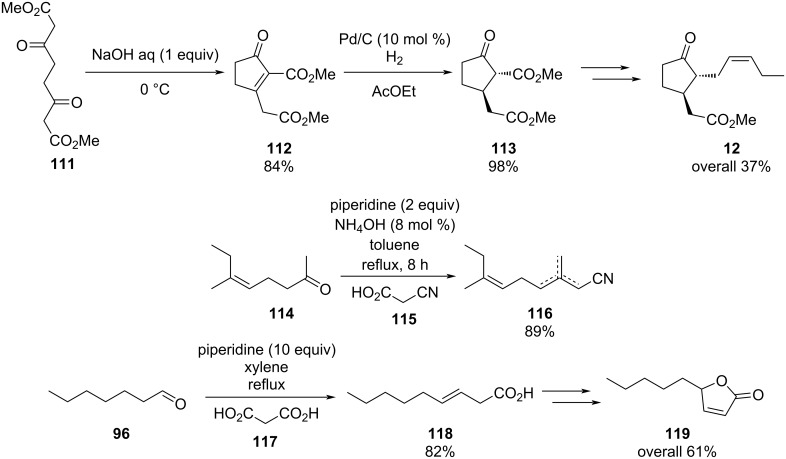
Examples of Knoevenagel condensations applied for fragrance components.

Knoevenagel condensations are also of considerable interest to the F&F industry (Scheme 26). The transformation is generally used to form α,β-unsaturated acids or nitriles from condensation with aldehydes or ketones [146–151], however, there are also some examples where cyclic or tetra-substituted olefins are formed [152–153].

**Scheme 27:**
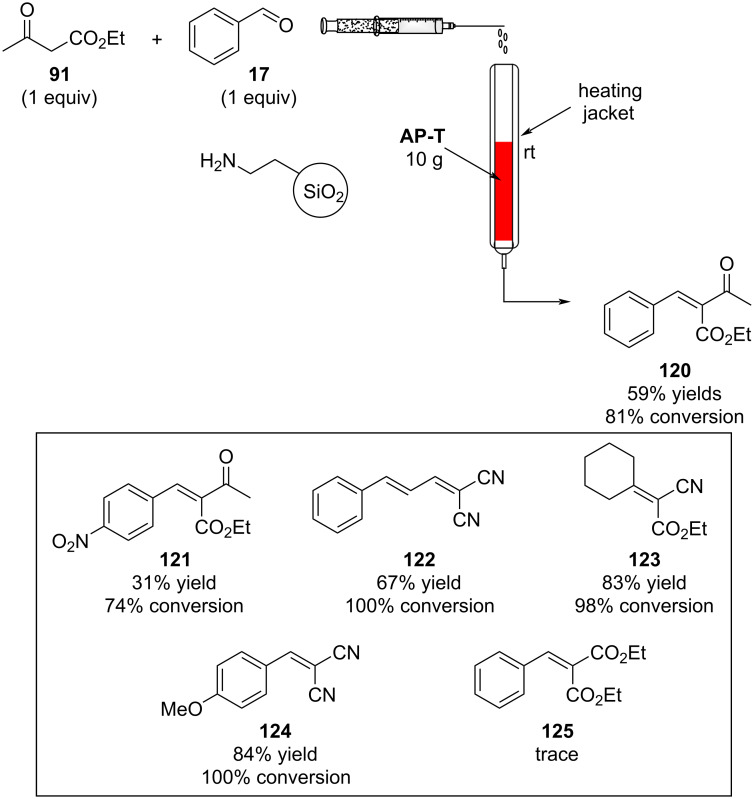
Flow apparatus for Knoevenagel condensation described in 1989 by Venturello et al.

The high processing volumes and short reaction times make the reactions quite suitable for continuous production via a flow system. Needless to say, the first example of Knoevenagel condensations in flow comes from Venturello et al. back in 1989 [154]. The authors exploited a glass column filled with 10 grams of aminopropyl-functionalised silica gel (AP-T). The reagents (10 mmol) were eluted through the column in the same way as gravimetric chromatography is performed (Scheme 27).

In this way, the authors claimed the product was completely collected after 250 mL of toluene elution. The silianol groups on the AP-T silica are suggested to play a key role in the water elimination step. Furthermore, the primary amine function on the silica shows better outcomes compared to a tertiary amine. Different products were prepared in moderate to excellent yields, however, with the less acidic diethyl malonate the catalyst proved to be less active.

In 2013, the Knoevenagel reaction between benzaldehyde (**17**) and malononitrile was performed using a similar flow apparatus, however, using a polystyrene-supported DABCO as catalyst. Wang’s group confirmed the stability of the latter, which provides excellent conversions (95–90%) over 10 hours of continuous runtime [155]. As such a simple chromatographic column can be exploited as flow reactor to perform initial feasibility tests.

**Scheme 28:**
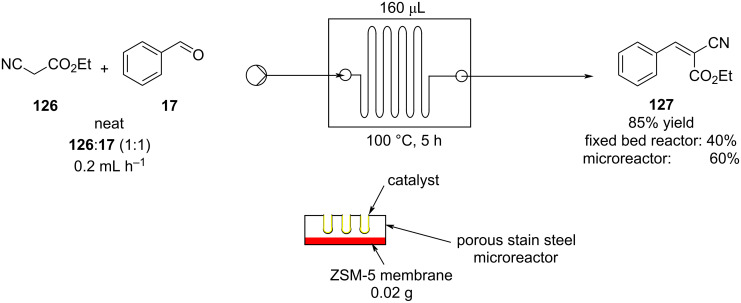
Knoevenagel reaction using a coated multichannel membrane microreactor.

As with all condensation reactions, the main problem when performed in flow is the elimination of water. Yeung et al. provided a solution using a coated multichannel microreactor with an integrated zeolite ZSM-5 membrane (Scheme 28) [156–157]. The heterogeneous catalyst used was a Cs-exchanged faujasite NaX. The membrane maximised the outcomes in comparison to tested fixed-bed and microreactors. The ZSM-5 membrane allowed fast water exclusion, however, long residence times (5 hours) were required. The authors claimed a more active catalyst would reduce the reaction times. As a result, over the years, the same group has provided improved alternative microreactors and catalysts; for example, a capillary microreactor coated with Cs-exchanged faujasite NaX was reported [158], along with a ZIF-8/NaA composite membrane microreactor [159]. These systems embodied more active and stable catalysts which were able to consequently operate for over 50 hours with delivery of excellent product yields (>95%). Also these systems, instead of using membranes for the water removal instead employed either DMSO or ionic liquids as additives for the water removal.

The same condensation (of **17** to **127**) was also performed by Clark et al. using a silica-coated microreactor with an AP-T catalyst deposited on the reactor’s walls [160]. The solvent-free approach brought about the product **127** in roughly 80% yield, however, effects of poisoning and catalyst leaching reduces the conversion by 20% over 8 hours of runtime. The poising was attributed to the presence of stationary areas caused by non-uniform flow in the reactor. Generally, this issue could be addressed by changing the configurational shape of the apparatus so to increase the mixing and flow uniformity. Nonetheless, new technique could also be applied, such as electroosmotic flow (EOF). The later technique has been widely used in flow for its incredible advantages compared to common pressure-driven systems [161]. Watts and Nikbin employed EOF in a piperazine-functionalised silica-catalysed Knoevenagel rreaction for the preparation of **127**. The authors managed to reduce the reaction times (2 hours vs 5 minutes) and yielded the product in 82% conversion, confirming the applicability of EOF technology in heterogeneous catalysed-flow systems [162].

Many of the products, such as **127** and its derivatives, are considered potential intermediates in the pharmaceutical industry [163–164], many other applications in flow have been described improving the catalyst stability. In 2016, Tao’s group developed a bioinspired metal-oxide-immobilized monolith, which was optimised for several reactions (reduction, esterification, and Knoevenagel) [165]. The authors employed a hierarchically porous silica (HPS) coated with a pyrogallol-based polyphenol film (PG). This type of supported system allowed incorporation of a greater amount of metal oxide compared to the standard impregnation. For the Knoevenagel reaction, Al_2_O_3_ was immobilised on the PG-HPS. The reagents were streamed into the reactor at 0.05 mL min^−1^ (roughly 30 minutes residence time) at 80 °C, with compound **127** being obtained in 89% conversion. The apparatus could be operated for over 24 hours, however, leaching of Al_2_O_3_ reduces the conversion to 76% [166].

**Scheme 29:**
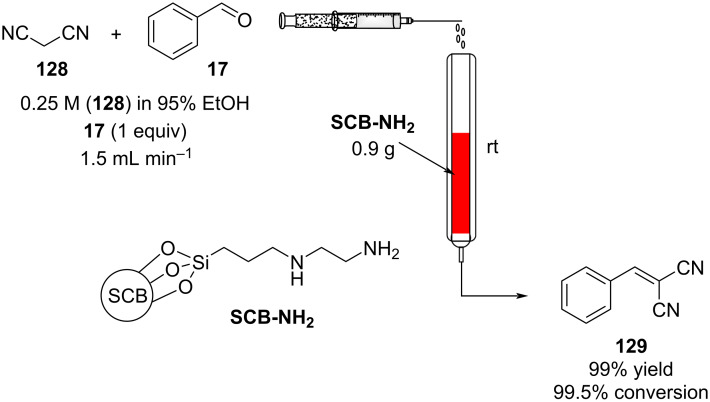
Continuous-flow apparatus for Knoevenagel condensation employing sugar cane bagasse as support developed by Ma et al.

The preparation of a heterogeneous catalyst often has higher associated costs, therefore once applied the species must be efficient and stable to generate over time good economic margins. Consequently, developing supports for stable catalysts in flow is becoming a major research task. To manage costs and increase the greenness of the process, many new supports have been developed. An interesting example are aerogels which present low densities, large surface areas, and interconnected porous structures which makes them highly suitable for low pressure drop column based catalysis [167]. In 2014, Isogai et al. employed nanofibrillar chitin aerogel as a potential sustainable heterogeneous catalyst for Knoevenagel condensations. The catalyst, presenting the amine functional groups on its surface, was loaded in a syringe along with the mixture of reagents (benzaldehyde and ethyl acetonitrile) and pumped at 0.01 mL min^−1^ at room temperature. The authors managed to obtain compound **127** in good to excellent yields (85–99%), and the catalyst could be reused over 5 times without signs of detriment [168]. In 2017, Ma et al. developed a sustainable amine-functionalised catalyst using sugarcane bagasse (SCB) as a support (Scheme 29). The as-prepared catalyst was found to be highly active for effecting Knoevenagel condensation at room temperature [169].

Polyacrylonitrile fibres (PNAF) were also successfully employed as alternative supports by the groups of Ma and Zhang in 2019. The supports were functionalised with different bases, such as 1,8-bis(dimethylamino)naphthalene, and prolinamide, and provided excellent mechanical properties and stability to the catalysts which could be reused successfully several times [170–171].

**Scheme 30:**
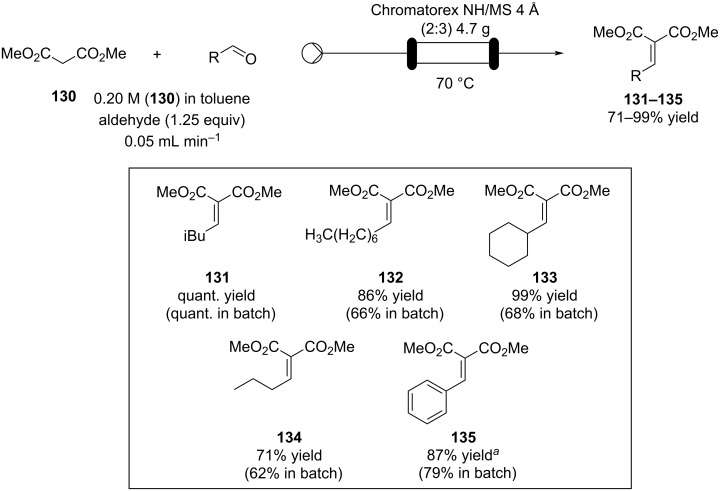
Knoevenagel reaction for the synthesis of **131**–**135** in flow using an amine-functionalised silica gel. ^a^A 10 × 200 mm column was used instead of a 10 × 100 mm column.

Knoevenagel reactions of aliphatic aldehydes are much more challenging, often the freshly formed double bond can further react and form Michael adducts with the more reactive starting materials. Kobayashi et al. developed an efficient flow apparatus able to overcome such issues. A primary amine-functionalised silica (Chromatorex NH) blended with molecular sieves 4 Å (MS 4 Å) were employed to increase the conversion toward the condensed products. The compounds **131**–**135** (Scheme 30) were gained in high to excellent yields compared to the corresponding batch method. In particular, species **131**, an intermediate in the synthesis of pregabalin (**138**), was obtained in >95% yield, which could be maintained for over 50 hours. Using a longer column (10 × 200 mm vs 10 × 100 mm), the apparatus operated over 100 hours with no signs of deterioration (90–100%).

**Scheme 31:**
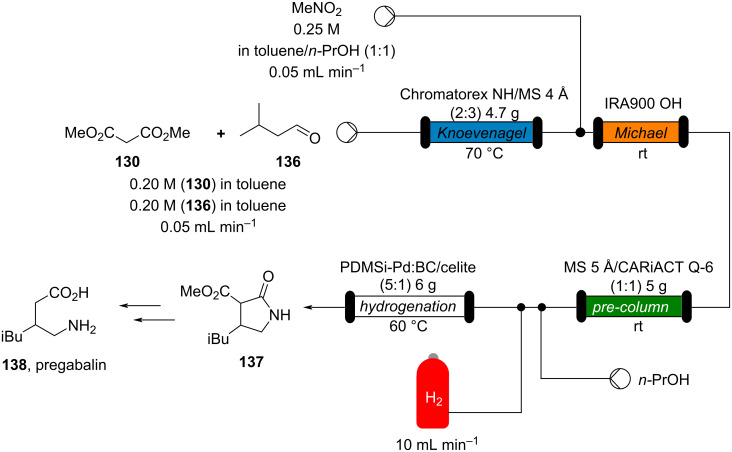
Continuous-flow synthesis of compound **137**, a key intermediate for the synthesis of pregabalin (**138**). PDMSi-Pd/BC: polysilane/bone charcoal-supported Pd catalyst. CARiACT Q-6: silica support.

The flow system was coupled with other reactor units to develop a continuous synthesis of compound **137**, an intermediate for (±)-pregabalin (**138**; Scheme 31). From compound **130**, a Michael reaction with nitromethane catalysed by basic Amberlite^®^ IRA900 (hydroxide form), installed the nitro group which was hydrogenated using a poly-silane-supported bone charcoal palladium catalyst previously developed within the same group [172].

The cyclisation occurs as soon as the amine group is formed (**137**). The authors revealed a column filled with MS 5 Å and silica had to be placed between the second and third step as low conversions were detected over the time (59–78%). The pre-column acts as an in-line purification for unreacted reagents or any byproducts formed during the first parts of the process. The following flow setup allowed **137** to be formed in 76–99% yields over 45 hours of runtime (space time yield 52.2 g L^−1^ d^−1^).

To fully satisfy the 12 principles of greenness, the Knoevenagel reactions can also be exploited in a continuous solvent-free process using screw reactors [173–175]. In this particular example, a twin-screw extruder (TSE) was employed. The reactants were added as solids in the apparatus. The rotating screws push the solid forwards, mixing the solid reactants together. The extruder has several heating channels which can be individually set at different temperatures. Once the mixture of vanillin (**2**), barbituric acid (**139**), and sodium carbonate were heated at 160 °C for 2 minutes, the product **140** was obtained quantitatively. The TSE apparatus was able to manufacture the material at 0.520 kg h^−1^.

**Scheme 32:**
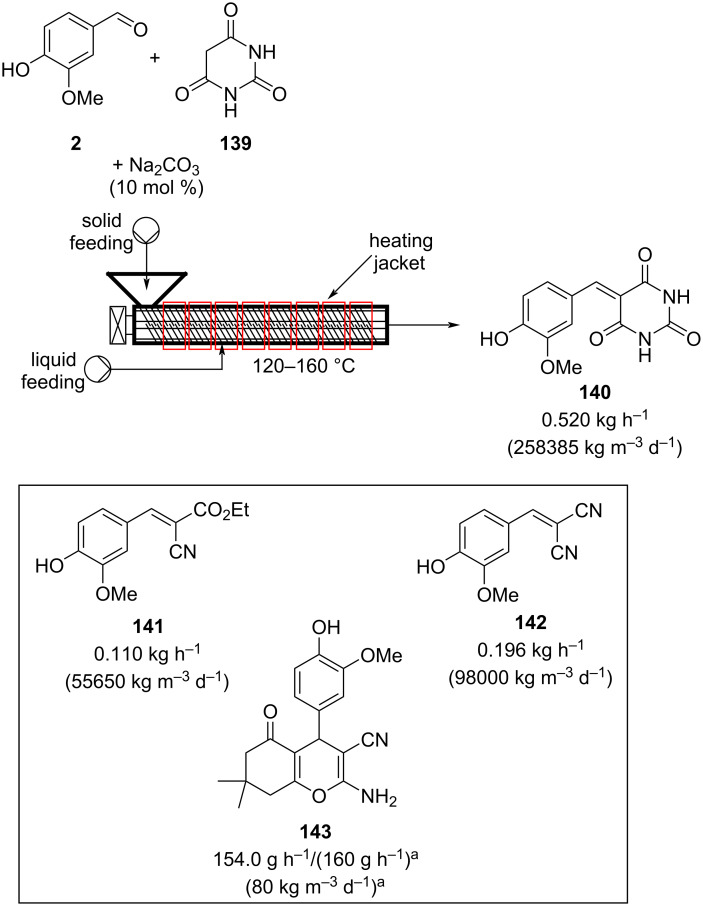
Continuous solvent-free apparatus applied for the synthesis of compounds **140**–**143** using a TSE. Throughput rate and space time yields (in brackets) are reported. ^a^The data are from the multicomponent reaction process instead of the Knoevenagel–Michael addition telescoped one.

Solid–liquid systems can also be exploited simply by adding a syringe pump inlet to the extruder. Malononitrile and ethyl cyanoacetate were used as examples, and the products **141** and **142** were produced in quantitative yield with extremely high throughputs (Scheme 32).

The authors also described the telescoping of a Knoevenagel–Michael addition process for the synthesis of **143**. The optimised method enables isolation of the desired material quantitatively at 154 g h^−1^. A one-pot like process, where all the solids were fed from the same feeder, was also developed proving the potentiality of this technique for immediate industrial applications.

**Scheme 33:**
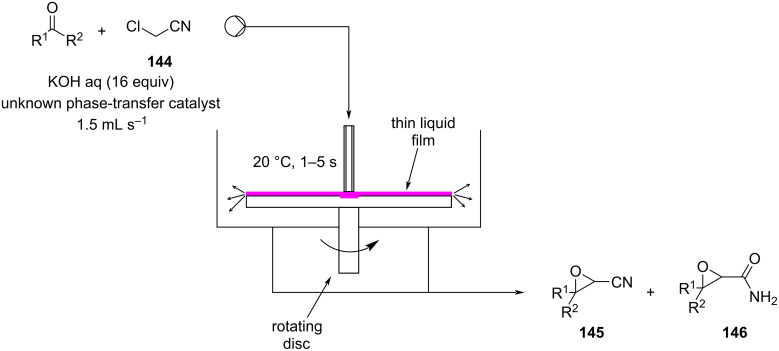
Lewis et al. developed a spinning disc reactor for Darzens condensation of **144** and a ketone to furnish the α,β-epoxynitrile **145**.

The Darzens reaction can also be considered as a condensation type reaction. Darzens products (epoxides, aziridines) are valuable building blocks, especially for the pharmaceutical and agrochemical industries [176–178]. Although a great number of publications are present in the literature, few continuous approaches have been described thus far. In 2001, Lewis et al. published a conference paper describing a continuous phase-transfer-Darzens condensation of a ketone for the preparation of pharmaceuticals (Scheme 33) [179].

A spinning disc reactor (SDR), which is depicted in Scheme 33, was employed. The solution of starting materials entered from the centre of the disc and drained out from a vessel which is placed below the disc assembly. The spinning applies a very high shear stress on the thin film (thickness ranges 50–600 μm) which leads to very high mass and heat transfer. The residence time is around 1–5 seconds, and the disc spinning was maintained at 4500 rpm. The system allowed for operation under milder reaction conditions (20 °C) compared to the original batch process (0 °C), and to give improved control over the formation of the byproduct **146** (0.1% vs 1.4%). The authors highlighted the linear relationship between the disc spinning rate and the product formation, suggesting better mixing was a key factor in the reaction’s outcomes. To improve the efficiency of the mixing, a textured disc with a counterclockwise spiral shape carved on its surface was employed. The desired product **145** was obtained in 88% conversion with a calculated throughput of 8 tonnes per year.

#### Conjugate addition

**Scheme 34:**
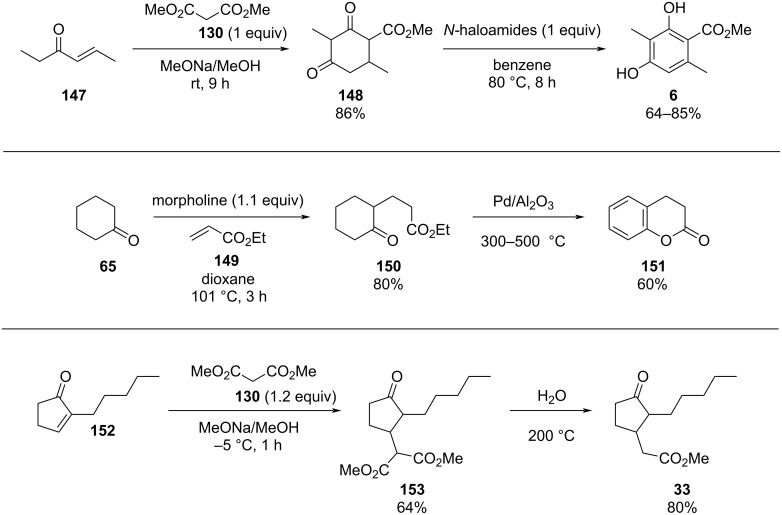
Some key industrial applications of conjugate additions in the F&F industry.

Conjugate addition reactions are commonly utilized in synthetic fragrance chemistry to generate new C–C and C–heteroatom bonds. The Michael addition, discovered in 1887 by Arthur Michael [180], has clearly been a milestone in the field of organic chemistry. Being versatile in the substrates and operationally simple to perform, Michael additions immediately found widespread industrial applications. Several examples of fragrance preparation using conjugate additions and more specifically Michael additions are described in the literature. Dihydrocoumarin (**152**) [181–182], methyl dihydrojasmonate (**33**) [183], resorcinol esters such as veramoss (**6**) [184], and many other well-known fragrances and cosmetic additives are industrially prepared via such related routes (Scheme 34).

**Scheme 35:**
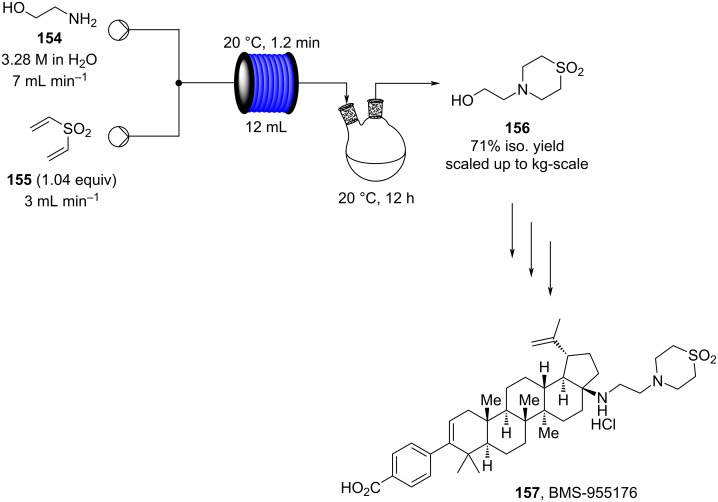
Continuous-flow synthesis of 4-(2-hydroxyethyl)thiomorpholine 1,1-dioxide (**156**) via double conjugate addition on route to BMS-955176 (**157**).

Flow chemistry has also been enacted to assist in several conjugate additions, reducing associated safety risks and improving selectivity. The continuous-flow synthesis of 4-(2-hydroxyethyl)thiomorpholine 1,1-dioxide (**156**), a key intermediate for the synthesis of the HIV maturation inhibitor (MI) BMS-955176 illustrates the benefits with respect to reduced handling of toxic/reactive intermediates and easy scaling to kilogram levels (Scheme 35) [185].

**Scheme 36:**
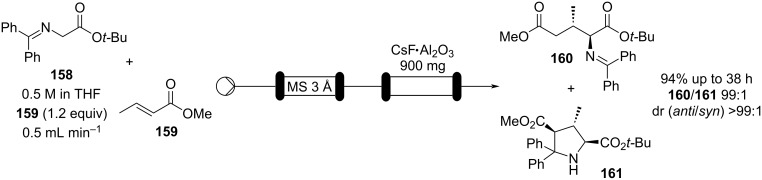
Continuous-flow system for Michael addition using CsF on alumina as the catalyst.

When nucleophiles containing an active methylene group are involved, the addition reaction can often be performed using catalytic amounts of base. Over time a broad range of different catalysts have been evaluated resulting in varying conversions and associated levels of regio- and stereoselectivity for the addition process [186–189]. Several solid inorganic bases have been considered as heterogeneous catalysts for Michael addition [190], for example, caesium fluoride on alumina has been recently exploited in the continuous-flow synthesis of glutamic acid derivatives (Scheme 36). The flow setup devised was used to reduce the residence time (increase throughput) and suppress the competitive [3 + 2] cycloaddition generating the pyrrolidine compound **161**. It was demonstrated that the flow system could be continuously run for 38 hours without any sign of decline in conversion or selectivity [191].

**Scheme 37:**
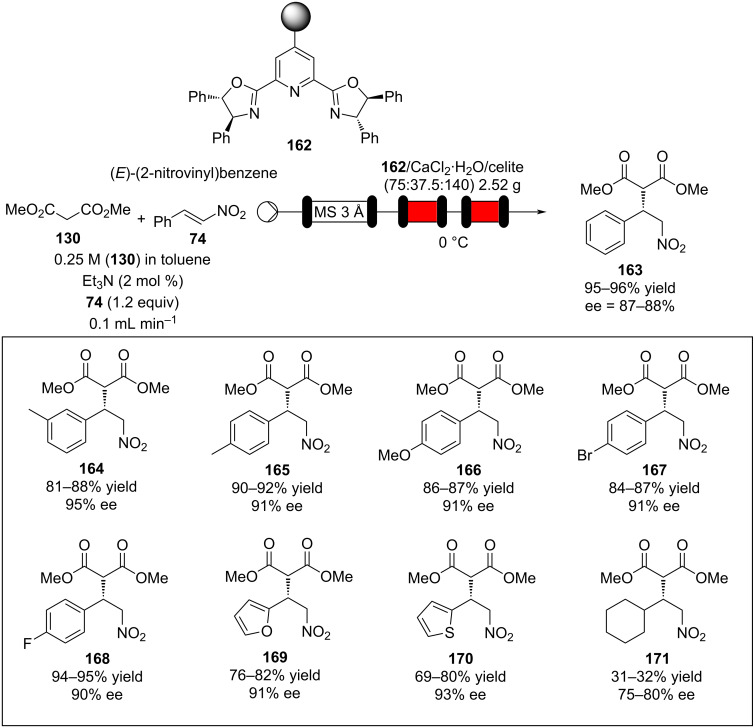
Calcium chloride-catalysed asymmetric Michael addition using an immobilised chiral ligand.

Similarly, calcium chloride has been used to promote asymmetric addition of malonates into unsaturated nitro compounds using an immobilised chiral bisoxazoline ligand in a flow process (Scheme 37) [192]. The reactor setup proved highly efficient with activated aromatic and heteroaromatic nitrostyrenes **163**–**170**, however, it showed reactivity limitations when aliphatic α,β-unsaturated nitro compounds were employed (**171**).

**Scheme 38:**
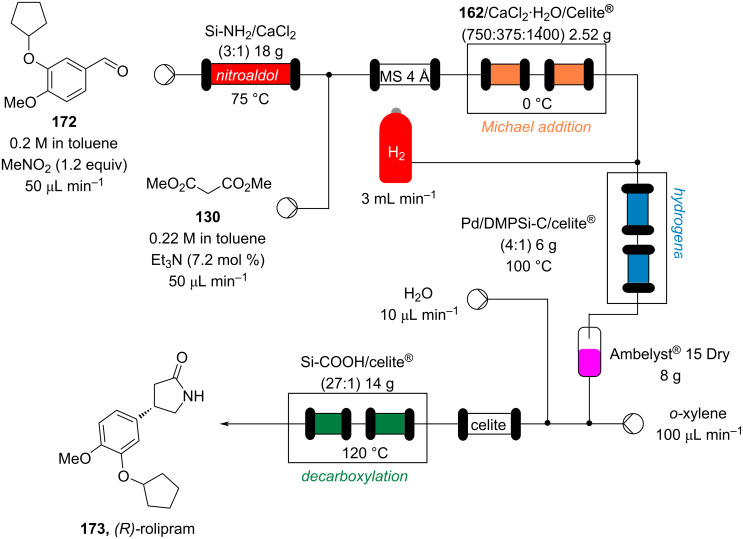
Continuous multistep synthesis for the preparation of (*R*)*-*rolipram (**173**). Si-NH_2_: primary amine-functionalised silica gel.

This procedure proved highly advantageous for adaption to the multistep continuous-flow synthesis of (*R*)-rolipram (Scheme 38) [193].

Starting from aldehyde **172**, a Henry condensation was enacted to prepare the corresponding nitrostyrene. After performing the Michael addition, as described above, hydrogenation using a developed polysilane-supported palladium/carbon (Pd/DMPSi-C) occurred. The flow stream was then degassed in a syringe filled with a sulfonic acid resin, which contributes to the ring formation. Decarboxylation was accomplished using a silica-supported carboxylic acid (Si-COOH) column at 120 °C streamed from top to bottom. The desired material was gained in 50% yield, and roughly 1 gram of enantiopure (>99% ee) **173** was isolated after 24 hours of collection.

**Scheme 39:**
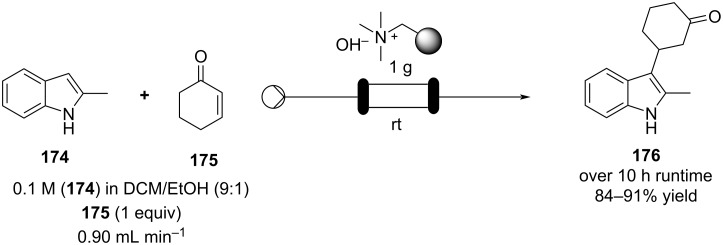
Continuous-flow Michael addition using ion exchange resin Amberlyst^®^ A26*.*

The use of several common ion exchange resins have been tested as catalysts for Michael addition between 2-methylindole and several α,β-unsaturated ketones and nitro compounds (Scheme 39).

Dried Amberlyst^®^ A26 (hydroxide form) was shown to be the most efficient species resulting in good to excellent yields. The synthesis of **176** in flow was demonstrated, the simple packed column system allowed easy isolation of the product and the catalyst activity was maintained over 10 hours [194]. A similar approach using Amberlyst^®^ IRA-900 (OH form) was employed for the Michael addition in the multistep flow synthesis to a key intermediate of pregabalin (**173**) by Kobayashi et al. (Scheme 38) [172].

**Scheme 40:**
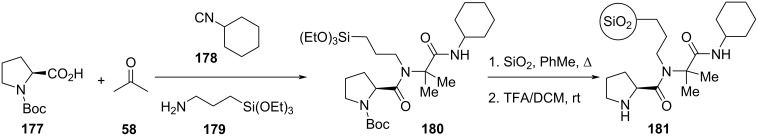
Preparation of the heterogeneous catalyst **181** developed by Paixão et al. exploiting Ugi multicomponent reaction.

Organocatalysis has been widely employed for Michael addition reactions and several metal-free enantioselective versions have been reported over the years [101,195–196]. However, most systems suffer from catalyst deactivation which necessitates the use of high catalyst loading but still reduces productivity over time. Heterogeneous catalysis paired with flow chemistry has proven a fertile area of research [197]. Systems using silica gel as a supporting matrix have been widely employed. For instance, Paixão et al. developed a silica-supported proline-based peptide **181**, whose preparation requires only three steps exploiting a Ugi multicomponent reaction (Scheme 40) [198–199].

**Scheme 41:**
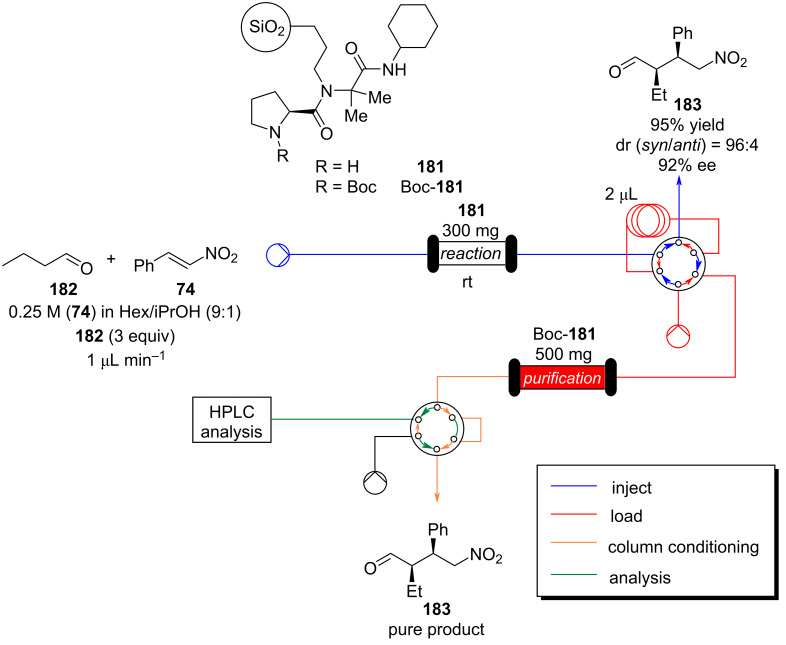
Continuous-flow system developed by the Paixão’s group for the preparation of Michael asymmetric adducts such as **183**.

Additionally, the authors applied a continuous-flow setup, which provides inline monitoring and product isolation (Scheme 41).

The system consists of two switching valves; the first one fills a 2 μL sample loop, which is then sent to a chromatographic column packed with Boc-**181** (red line), and the second one connects the latter output with a chiral column, which analyses the enantiopurity of the purified adduct **183** (green line) [199]. The system allows a significant reduction in the analysis and purification times. The flow system employing the immobilised catalyst **181** resulted stable for over 11 rounds (TON = 304). The apparatus was also tested for the preparation of other 9 substrates, giving good to excellent yields (67–95%) with high selectivity (*syn*/*anti*, 96:4, 82–92% ee).

**Scheme 42:**
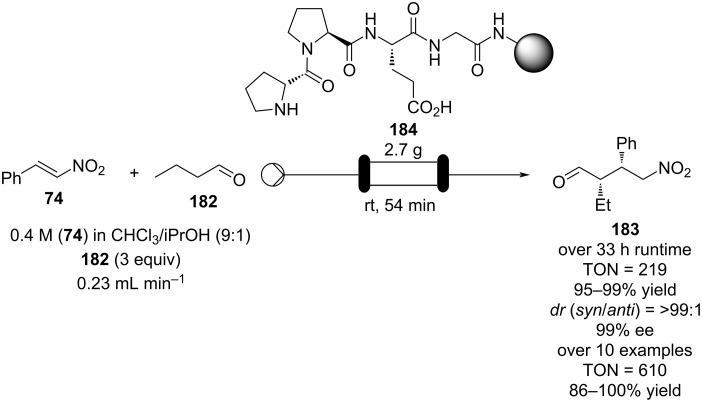
Continuous-flow synthesis of nitroaldols catalysed by supported catalyst **184** developed by Wennemers and Arakawa.

Using a different peptidic catalyst supported on polystyrene **184**, Wennemers and Arakawa developed a flow system capable of preparing over 450 mmol (>100 g) of Michael addition products in good to excellent yields (87–99%) and high selectivity (*syn*/*anti*, 30:1, 94–97% ee) using only 0.8 mmol of catalyst (TON = 610) (Scheme 42) [200].

**Scheme 43:**
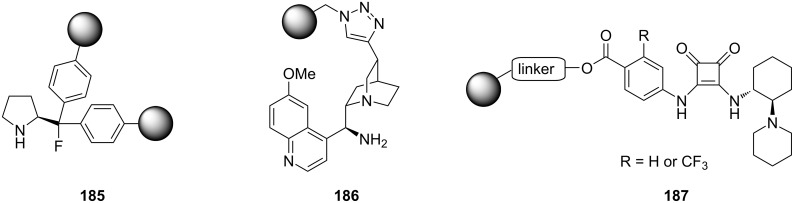
Heterogenous polystyrene-supported catalysts developed by Pericàs and co-workers.

Other polystyrene (PS)-supported catalysts for asymmetric conjugate additions have additionally been developed. PS-supported fluorinated proline **185**, quinine **186**, and squaramide **187** have all been prepared and exploited as organocatalysts by the group of Pericàs (Scheme 43) [201–204]. Interestingly, all the immobilised versions have demonstrated greater stability with lower deactivation rates when used in continuous-flow systems.

**Scheme 44:**
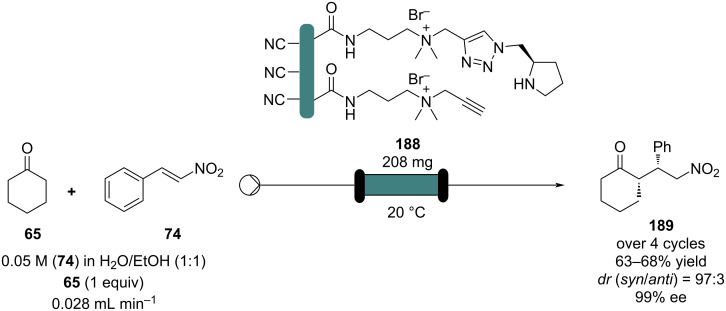
PANF-supported pyrrolidine catalyst for the conjugate addition of cyclohexanone (**65**) and *trans-β-*nitrostyrene (**74**) in flow.

Wang and Zhang et al. applied PNAF-supported catalyst in flow for asymmetric conjugate addition of cyclohexanone (**65**) and *trans-*β*-*nitrostyrenes (e.g., **74**) [205]. The PNAF enables the use of greener solvents such as water and ethanol in the Michael reaction and creates a stable and robust heterogeneous catalyst which can be used over 4 cycles with comparable results (Scheme 44). The authors also claimed better outcomes using the flow approach compared to batch (68% in 12 h vs 63% in 24 h), which could be due to the higher effective concentration of the catalyst.

**Scheme 45:**
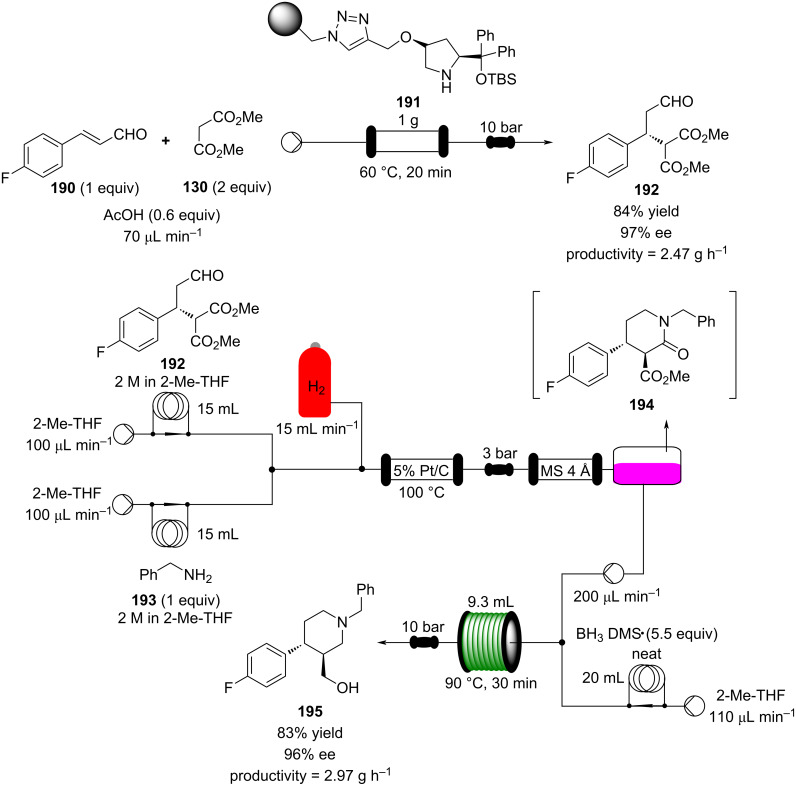
Synthesis of (−)-paroxetine precursor **195** developed by Ötvös, Pericàs, and Kappe.

In 2019, Pericàs, Kappe and Ötvös adopted a polymer-supported organocatalyst **191** for the multistep synthesis of (−)-paroxetine precursor **195**, a serotonin uptake inhibitor (Scheme 45) [206]. The synthesis was developed starting from dimethyl malonate (**130**) and 4-fluorocinnamaldehyde (**190**) for the synthesis of the intermediate **192**. The such-developed catalyst delivers the desired material in good yield (84%) with a high productivity (2.47 g h^−1^). The flow system developed for the conjugate addition operates in solvent-free conditions (SFC), therefore is isolated from the subsequent two steps of the multi-synthesis. A solution of **192** in 2-methyltetrahydrofuran (2-Me-THF) was then mixed with benzylamine for reductive amination to form the cyclic amide **194**. Further reduction of the both amide and ester groups with borane dimethyl sufide (BH_3_∙DMS) allowed to isolate the desired material **195**. With this two-steps continuous-flow system, 4.95 g of pure (−)-paroxetine were isolated in 100 minutes of collection (productivity = 2.97 g h^−1^).

**Scheme 46:**
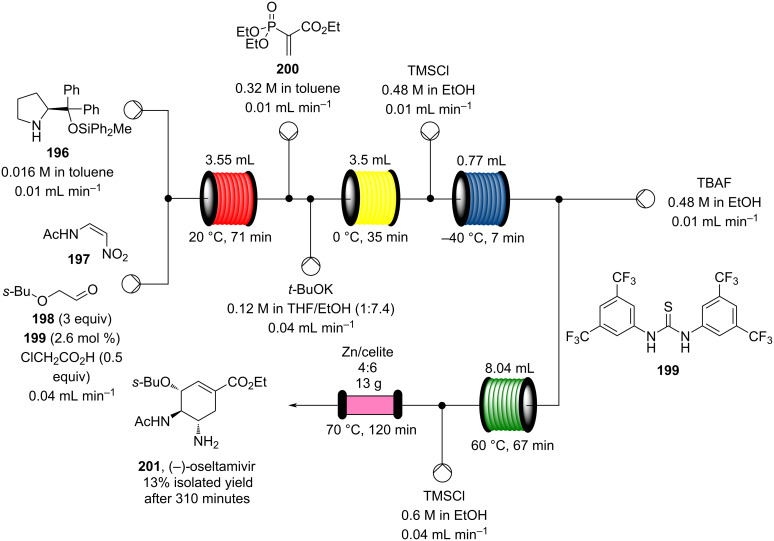
Continuous-flow approach for the 5-step synthesis of (−)-oseltamivir (**201**) as devised by Hayashi and Ogasawara.

Other examples utilising homogenous catalysts have also been shown to be effective in flow such as in the synthesis of (−)-oseltamivir (**201**) by Hayashi and Ogasawara (Scheme 46) [207]. The flow system is divided in 5 units comprising the 5 reaction steps: conjugate addition (red reactor), Michael addition and intramolecular Horner–Wadsworth–Emmons reaction (yellow reactor), nitrate anion protonation (blue reactor), epimerization (green reactor), and reduction (pink reactor). The apparatus employs a Comet-X-01 micromixer for better mixings, Teflon^®^ tube reactors, and one Biotage SNAP empty cartridge. After 310 minutes of collection, the mixture was manually extracted and purified through standard column chromatography to gain **201** in 13% overall yield. The result is comparable to the one-pot batch procedure previously described (14%) [208].

**Scheme 47:**
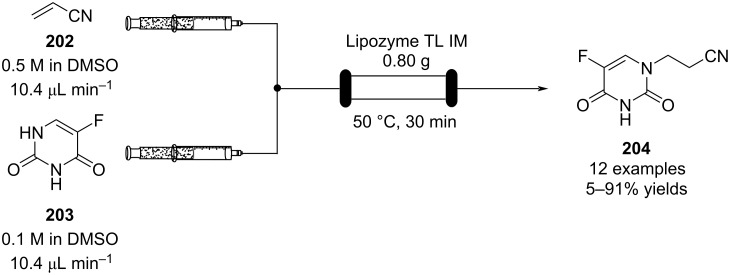
Continuous-flow enzyme-catalysed Michael addition*.*

In 2014, a continuous enzyme-catalysed conjugate addition system was reported by Du et al. [209]. Using silica-absorbed Lipozyme^®^ as the catalyst, the authors managed to synthesise twelve adducts between various pyrimidine derivatives (5-fluorouracil, uracil, and thymine) and acrylate acceptors with a reduction in the reaction times compared to the batch reaction (Scheme 47).

**Scheme 48:**
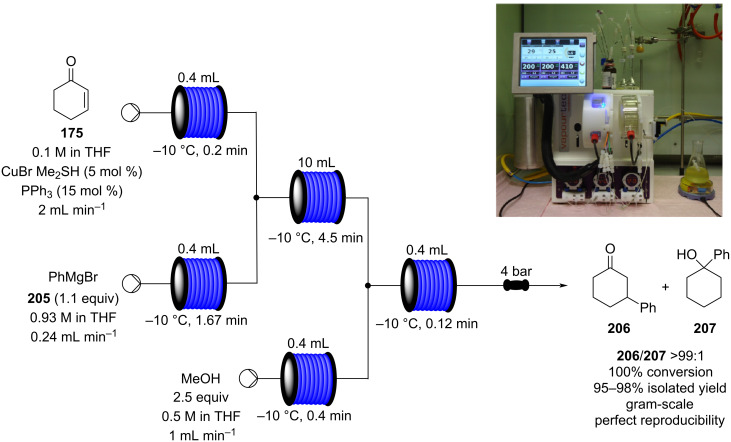
Continuous-flow copper-catalysed 1,4 conjugate addition of Grignard reagents to enones. Reprinted with permission from [212]. Copyright 2013 American Chemical Society.

Other types of conjugate addition processes have also been performed within continuous-flow systems, for example, 1,6-conjugate addition to *para*-quinone methide using either thiols [210] or alkylzinc compounds [211]. A continuous-flow copper-catalysed 1,4-conjugate addition to α,β-unsaturated carbonyls has been developed by Ley et al. [212]. For this example, the commercial flow platform Vapourtec E-series was employed. As organometallic compounds are moisture sensitive, the choice of the pumping system was crucial in order to avoid quenching or long-term stability issues. Alternative HPLC-type pumps are more prone to fouling and blocking, due to precipitation of hydrolytic materials. On the other hand, peristaltic pumps, in which the fluid is contained within a tube and wetted parts are thus minimised at the pump, allows better long term performance and reduces maintenance. Thus, in the work of Ley et al. better results in terms of regioselectivity 1,4:1,2 were obtained compared to the corresponding batch process (99:1 vs 1:1), which also required lower temperatures (−60 °C vs −10 °C) and longer reaction time (2 hours vs ≈6 minutes) (Scheme 48) [213]. To highlight the reproducibility of the system, a second operator was asked to optimise the same experiment and essentially identical outcomes were obtained.

#### Hydrogenation

**Scheme 49:**
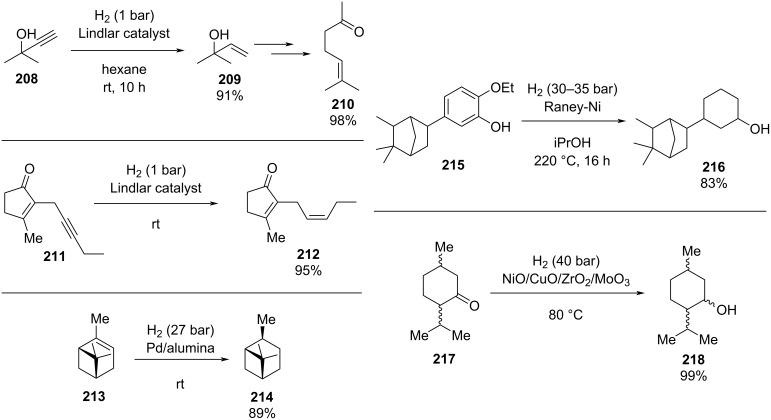
A collection of commonly encountered hydrogenation reactions.

The reduction of alkynes to alkenes/alkanes, alkenes to alkanes and ketones/aldehydes to alcohols are important transformations producing countless valuable products for which catalysed hydrogenation represents a key process. Alkyne hydrogenation is particularly relevant to the synthesis of monoterpene derivatives, for example, 6-methyl-5-hepten-2-one (**210**) [214], itself a fragrance ingredient but also a precursor to many others, which is prepared from the hydrogenation product **209** of 2-methylbut-3-yn-2-ol (**208**) [215–216]. Other examples include the synthesis of *cis*-jasmone (**212**) [217] and methyl jasmonate (**12**) which requires hydrogenation over Lindlar’s catalyst [218]. Examples of alkene hydrogenation include the reduction of α*-*pinene (**213**) to *cis*-pinane (**214**) [219], a useful intermediate in the preparation of linalool (**10**) and the aromatic reduction of catechol derivative **215**, to prepare isocamphyl cyclohexanol (**216**) [220], a synthetic sandalwood equivalent. Also, the hydrogenation of ʟ-isopulgol to ʟ-menthol (ʟ*-***218**) [221] and menthone/isomenthone **217** to menthol/isomenthol diastereomers **218** [222] are all important and commonly used transformations (Scheme 49).

**Figure 7:**
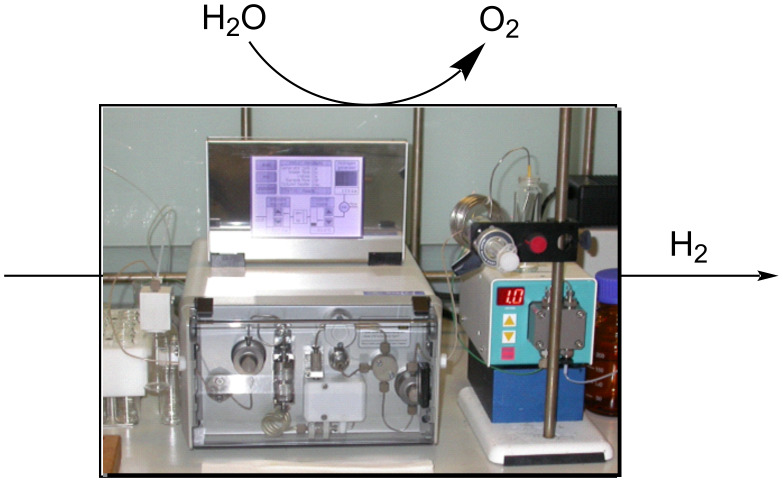
The ThalesNano H-Cube^®^ continuous-flow hydrogenator.

Improvements in safety, performance and environmental impact can be expected when carrying out hydrogenation reactions under flow conditions [223]. Batch hydrogenation processes are often limited by gas solvent diffusion rates (interfacial contact having a large impact). The implementation of flow technology allows for the rapid saturation of reaction media by making use of biphasic in-line mixing or gas-permeable membranes, leading to improved reaction kinetics. Devices such as the ThalesNano H-Cube^®^ [118,224–233] (Figure 7) are used to effect highly efficient in-line mixing of hydrogen on a laboratory scale. Hydrogen is generated from electrolysis of water giving oxygen (or ozone) as a byproduct. The hydrogen gas is sparged into the liquid flow stream, creating bubble or pipe flow. A variety of semi-disposable catalyst cartridges can be incorporated into the system which minimise user contact with potentially toxic and pyrophoric materials (i.e., Raney nickel) and facilitate a broad range of transformations.

**Scheme 50:**
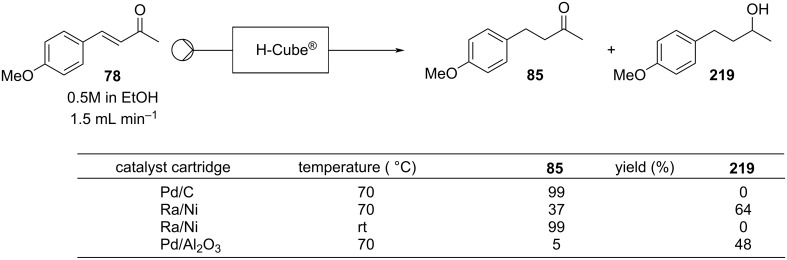
Chemoselective reduction of an α,β-unsaturated ketone using the H-Cube^®^ reactor.

The convenience of the H-Cube^®^ with the facility to swap catalyst cartridges offers an attractive approach. In 2011, it was reported for the chemoselective reduction of an α,β-unsaturated ketone **78** [118]. The reduction could be controlled for the ketone **85** by using either a Pd/C cartridge at 70 °C or a Raney nickel cartridge at room temperature or, alternatively, the reaction can be progressed through to the alcohol **219** under harsher conditions (Scheme 50).

**Scheme 51:**
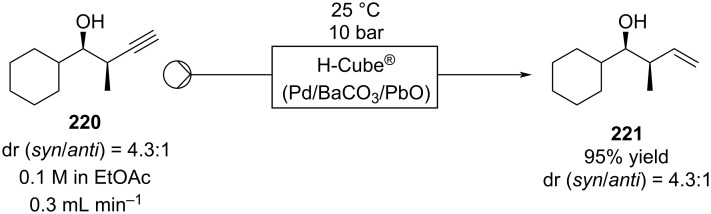
Incorporation of Lindlar’s catalyst into the H-Cube^®^ reactor for the reduction of an alkyne.

The chemoselective reduction of alkynes to alkenes has also been achieved in flow with a Lindlar catalyst cartridge (Scheme 51) [234]. The product, **220**, of a Marshall homopropargylation flow procedure was subjected to hydrogenation following dissolution in EtOAc to effect partial reduction of the triple bond, yielding the olefin **221** in 95% yield with a high diastereomeric ratio (4.3:1). The standard conditions were 25 °C at 10 bar using a Pd/BaCO_3_/PbO catalyst cartridge. However, by increasing the pressure to 30 bar it was possible to carry out the transformation in CH_2_Cl_2_.

**Scheme 52:**
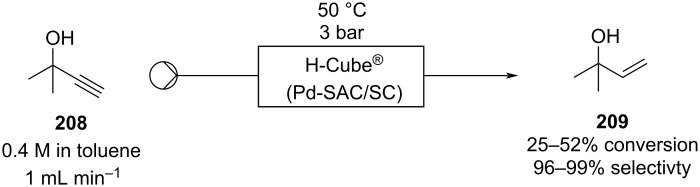
Continuous-flow semi-hydrogenation of alkyne **208** to **209** using SACs with H-Cube^®^ system.

Recently, the H-Cube^®^ reractor has also been employed for the semi-hydrogenation of intermediate **208** using single-atom heterogeneous catalysts (SACs) [235–238]. These catalysts, developed by Pérez-Ramírez and co-workers, consist of atomically dispersed noble metals on an organic host, usually nitrogen-enriched carbon materials. The catalysts are mixed over silicon carbide (SC), placed in a cartridge, and then tested in the H-Cube^®^ apparatus (Scheme 52). The catalysts were highly selective (96–99%) and reusable (up to 50 hours of runtime), however, low conversions are still recorded (25–50%).

In addition, many other diverse reduction processes have been effected by the H-Cube^®^ system. It has been utilised for the reduction of amides [239], nitro compounds [240–242], nitriles [243–245], azides [246–247], diazo compounds [248], and for carrying out reductive aminations [249–251] on a laboratory scale, while the H-Cube^®^ midi, has been designed specifically to be used in larger scale processes [252–254]. Recently, the H-Cube^®^ apparatus has also found use in the evaluation for biomass-derived chemicals recovery, such as Ru-catalysed hydrogenation of methyl levulinate (**262**) from lignocellulosic biomass to γ-valerolactone (**263**) [255], and scrap waste recovery, such as the scrap ceramic-cores of automotive catalytic converters (CATs) employed in the chemoselective hydrogenation of cinnamaldehyde to hydrocinnamyl alcohol [256–257].

**Figure 8:**
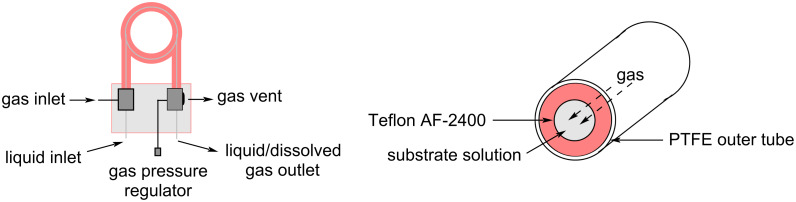
The standard setups for tube-in-tube gas–liquid reactor units.

Alternatively, tube-in-tube gas–liquid reactors [258–259] have become popular to facilitate efficient mixing in flow by enabling pressurised gas permeation across a membrane into a coaxial liquid flow. The most common design comprises a coiled non-permeable outer tube (typically stainless steel, polytetrafluoroethylene or PTFE) under positive gas pressure and an inner gas-permeable tube (Teflon^®^ AF-2400) that thus allows the gas to diffuse from the outer tube to the inner tube. The high surface area of the permeable tube enables higher gas–liquid contact (Figure 8), and leads to rapid, highly efficient gas saturation of the reaction medium.

Both approaches have been used with great success in recent years, with the latter being used for several other gaseous reactions including ozonolysis, carbonylations, carboxylations, reactions requiring ammonia- and oxygen-mediated reactions (increasingly in combination with photochemistry) as well as hydrogenations [17]. In many instances, telescoping of reaction steps is entirely possible and this is indeed being illustrated by their increasing incorporation into unified multistep total syntheses [260–264].

**Scheme 53:**
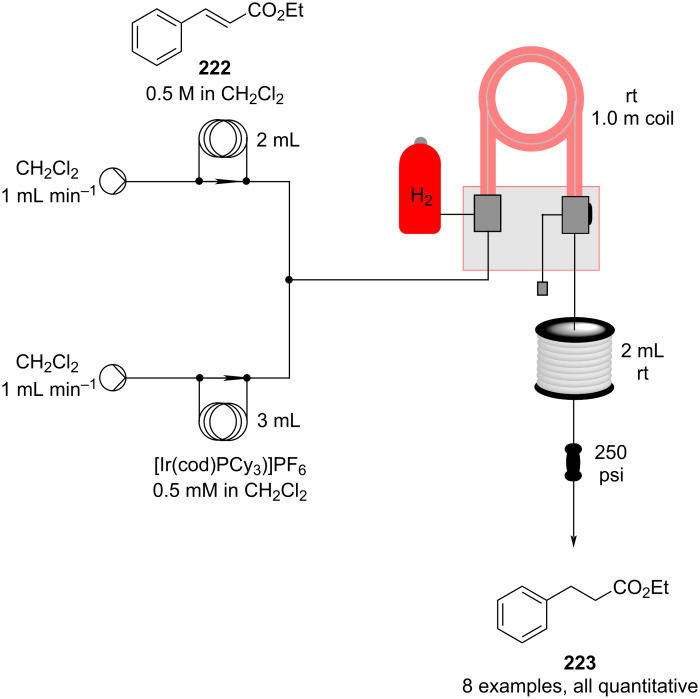
Homogeneous hydrogenation of olefins using a tube-in-tube reactor setup.

In 2011, the tube-in-tube reactor was used for the hydrogenation of a range of olefins in flow by the Ley group [265]. Crabtree’s catalyst (0.1 mol %) was used (injected as a separate flow stream via an injection loop) to study the hydrogenation of ethyl cinnamate (**197**) under continuous hydrogenation conditions (Scheme 53). The conversion was found to be highly dependent on the applied hydrogen pressure (≈50% at 10 bar vs 100% at 30 bar). Having highly precise pressure control, coupled with a large temperature window are significant advantages of the tube-in-tube reactors. The methodology was applied to seven other structures vaguely reminiscent of fragrances/fragrance intermediates and quantitative yields were obtained in each case after rapid optimisation.

**Scheme 54:**
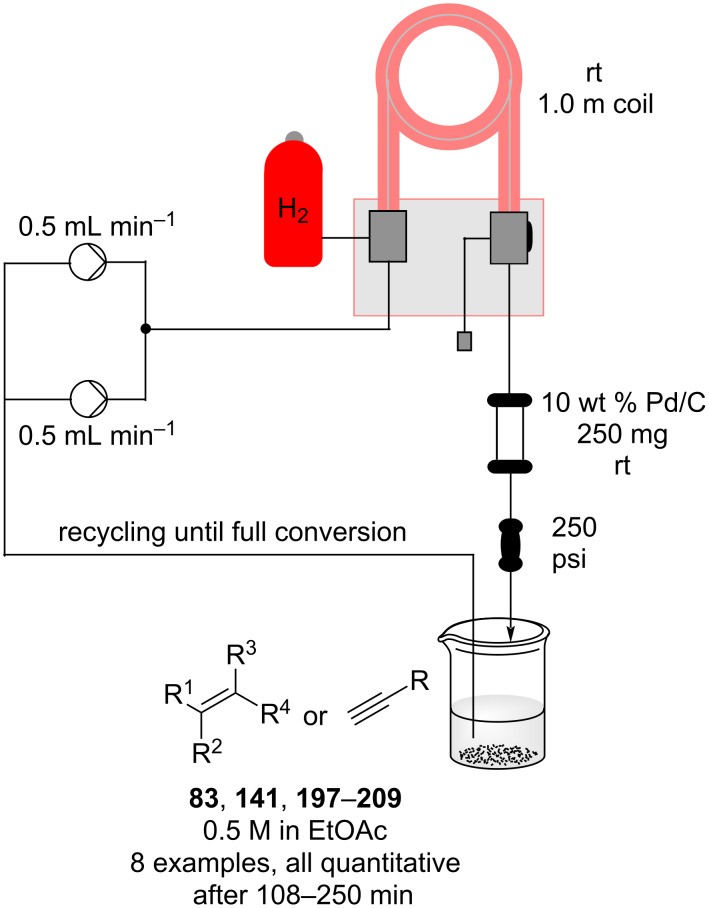
Recyclable heterogeneous flow hydrogenation system.

A heterogeneous hydrogenation protocol, utilising a Pd/C-packed catalyst cartridge, was also developed (Scheme 54) and applied to a further 8 substrates with all being obtained in quantitative yields (Table 6). Here, a recycling approach was taken, ensuring that all starting materials reached full conversion, the number of passes necessary varied according to the substrate (alkyne **230**, for example, took much longer than all others ≈250 min).

**Table 6:** Substrate screening of the recyclable heterogeneous flow hydrogenation system.

Entry^a^	Substrate	Product	Time (min)	Yield (%)

1	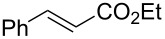 **222**	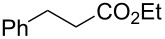 **223**	125	quant.
2	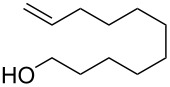 **224**	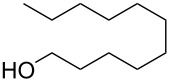 **225**	130	quant.
3	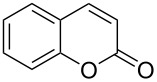 **90**	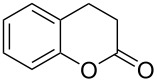 **151**	120	quant.
4	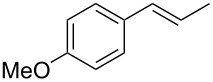 **226**	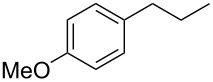 **227**	115	quant.
5	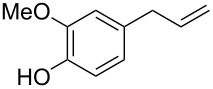 **228**	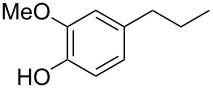 **229**	120	quant.
6	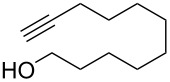 **230**	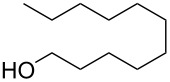 **225**	250	quant.
7	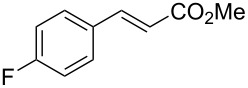 **231**	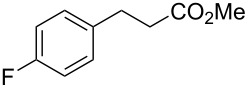 **232**	108	quant.
8	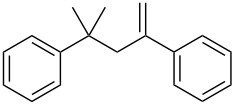 **233**	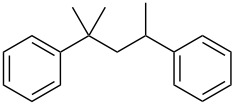 **234**	110	quant.

^a^Reactions conducted on 5 mmol scale.

**Scheme 55:**
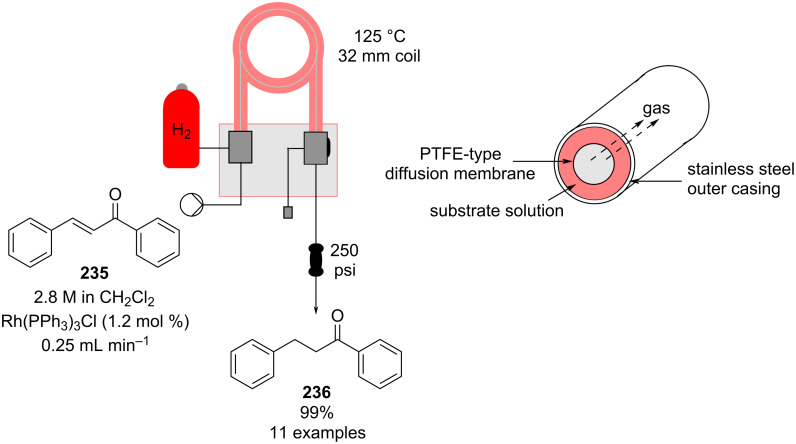
Leadbeater’s reverse tube-in-tube hydrogenation system for olefin reductions.

In a similar approach, the Leadbeater group investigated the incorporation of Wilkinson’s hydrogenation catalyst in a reverse tube-in-tube reactor (gas permeation from inner to outer tubing) to effect reduction of a set of 11 compounds (Scheme 55) [259]. A scale-up operation for the hydrogenation of chalcone (**235**) gave a productivity of 9 g h^−1^, equating to roughly 227 g d^−1^.

**Scheme 56:**
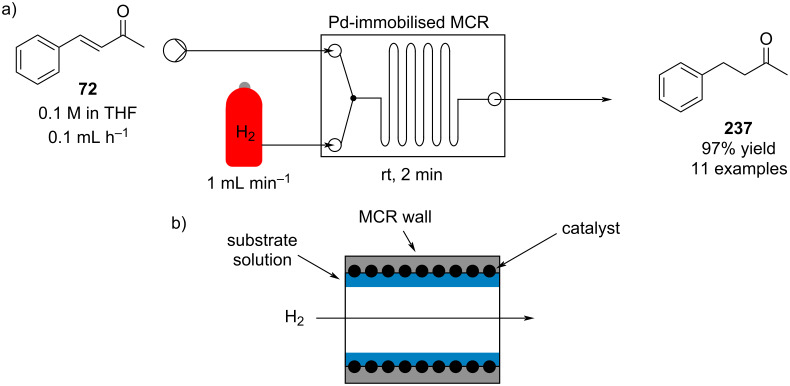
a) Hydrogenation using a Pd-immobilised microchannel reactor (MCR) and b) a representation of the ideal conditions for an optimum interphase system.

To achieve better gas–liquid–solid interaction, microreactors are a good choice for small scale processes. As an example, Kobayashi’s group performed a microencapsulation of Pd to immobilise the metal on the glass microchannel walls within the reactor (a microchannel reactor ‘MCR’) [266]. The authors pointed out the importance of the flow rates which affected contact times and thus overall conversions. As shown in Scheme 56, a thin film of liquid wets the microreactor’s coated wall where the catalyst was deposited, whereas the less dense hydrogen flows through the centre (an example of pipe flow). The same group also employed the reactor for use with scCO_2_ [267].

**Scheme 57:**
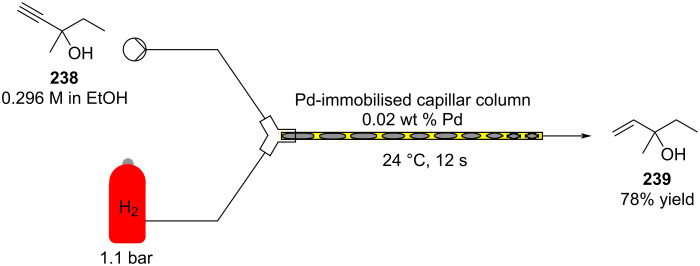
Hydrogenation of alkyne **238** exploiting segmented flow in a Pd-immobilised capillary reactor.

A different method for surface contact enhancement was explored by Kreutzer et al., who developed a hydrogenating system using segmented flow (Scheme 57) [268].

The internal glass capillary tubes were coated with alumina and treated to deposit a layer of Pd nanoparticles. The authors employed a segmented gas–liquid flow where alternating bubbles of hydrogen and substrate are generated inside the capillary. The hydrogen consumption could be visually followed from the decrease in size (volume) of the gas bubble as it progresses along the tube. The system was applied to the semi-hydrogenation of 3-methyl-1-pentyn-3-ol (**238**) reaching a theoretical optimum yield around 78%. The authors also claimed lower outcomes were obtained without the use of segmented flow (57%).

**Scheme 58:**
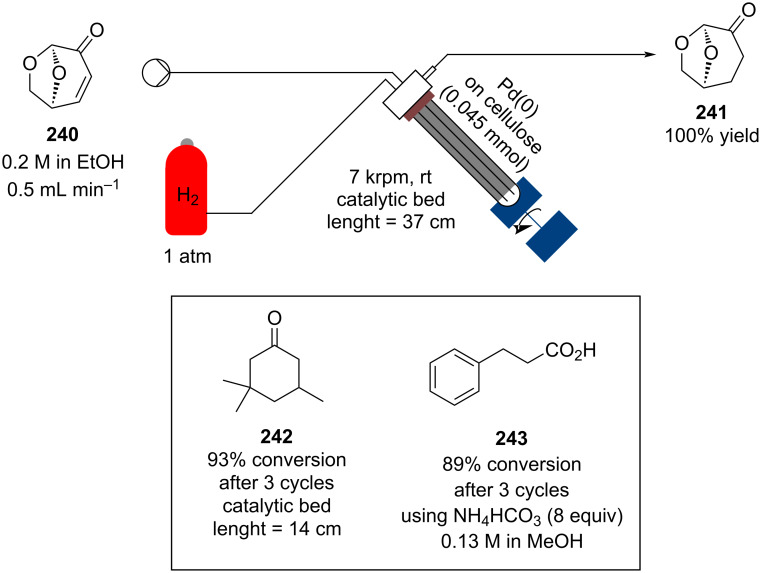
Continuous hydrogenation system for the preparation of cyrene (**241**) from (−)-levoglucosenone (**240**).

Recently, a Vortex fluidic device (VFD) has emerged as a promising process intensification tool. The apparatus consist of a fast rotating surface that creates dynamic thin films which generates a high mass and heat transfer regime. Several examples have already been setup to perform different chemical transformations [269]. In 2019, the group of Chalker and Raston utilised an VFD-based hydrogenating system demonstrating sucessful reduction of nitro groups and alkenes [270]. A catalyst system comprising Pd nanoparticles dispersed on cellulose paper was easily prepared and could be reusable over 10 times without deactivation. The apparatus was also explored for the continuous hydrogenation of (−)-levoglucosenone (product **240**) (Scheme 58) and reduction of isophorone (product **242**) in 93% conversion after recycling the solution three times back through the device. The VFD has also found utility in transfer hydrogenations using ammonium formate, hydrogenated cinnamic acid (**243**) was detected in 89% conversion after the third cycle.

**Scheme 59:**
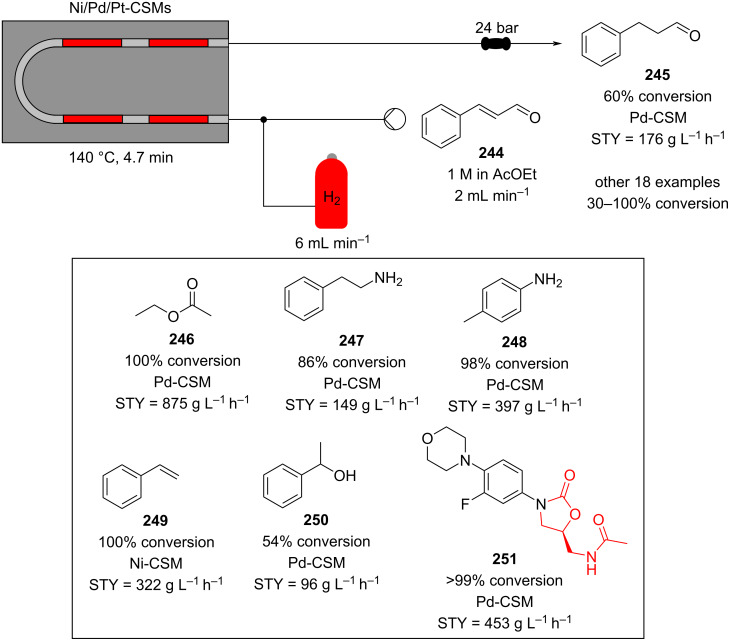
Continuous hydrogenation system based on CSMs developed by Hornung et al.

A valuable alternative to the above described apparatus has been developed by Hornung et al. The authors addressed the need for efficient mixing against the drawback of the many common catalyst supports; high pressure drops (small particle/high surface area), brittleness, inhomogeneity, and often long complex preparations. The flow system selected adopted a tubular reactor filled with catalytic static mixers (CSM’s) [271]. These static mixers were coated with metals such as nickel, palladium, or platinum through either electroplating or cold spraying. The supporting matrix were made from robust metallic alloys (Ti, CoCr, and stainless steel) and manufactured using 3D printer techniques. The CSM designs enhance the biphasic mixing ensuring a high interfacial contact area. Such system has been investigated for the reduction of alkenes, alkynes, ketones, diazo-, nitro compounds, nitriles, imines, and aryl halides [272]. In all the investigated examples, the desired products were detected in moderate to excellent conversions (50–100%). Furthermore, the group also optimised various hydrogenation processes of industrial interest such as the hydrogenation of vinyl acetate and synthesis of **225**; a key intermediate for linezolide (**251**), a valuable last-line-of-defence antibiotic (substructure depicted in red, Scheme 59) [273–274].

**Scheme 60:**
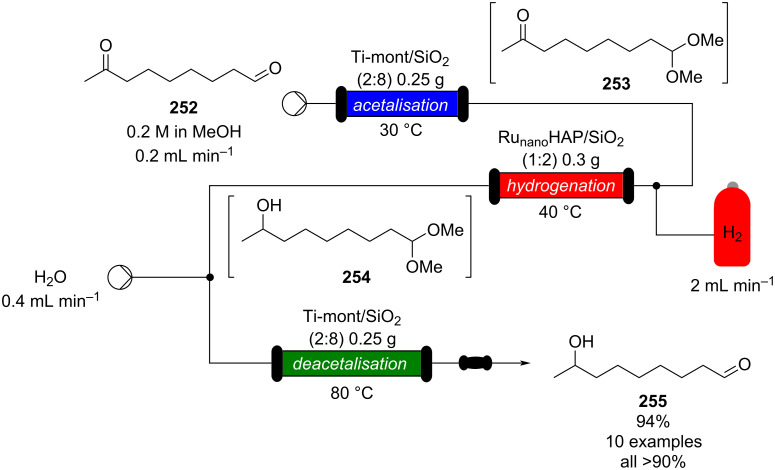
Chemoselective reduction of carbonyls (ketones over aldehydes) in flow.

In 2013 a selective flow reduction of ketones over aldehydes using direct in-line H_2_ mixing was reported [275]. The substrates **252** comprised both aldehyde and ketone functionalities and it was found that, by initial acetalisation of the more reactive aldehyde moiety, the ketone could be selectively hydrogenated, leaving the masked aldehyde intact (Scheme 60). A Ti(IV) cation exchanged montmorillonite (Ti-mont) was used as a heterogeneous acid catalyst for the acetalisation/deacetalisation and a hydroxyapatite (HAP)-supported Ru nanoparticle catalyst packed reactor was used for the hydrogenation, both operated at slightly elevated temperatures.

**Scheme 61:**
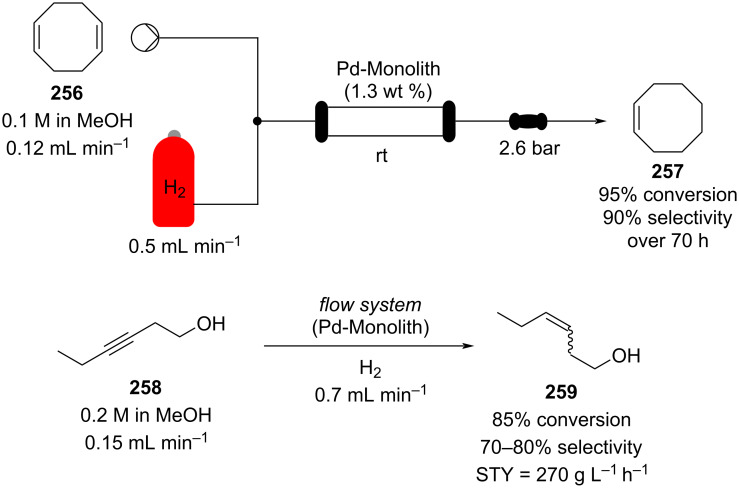
Continuous system for the semi-hydrogenation of **256** and **258**, developed by Galarneau et al.

Other examples of heterogeneous catalysts used for generating telescoped multistep synthesis have been reported. For example, Pd-immobilised silica monoliths have been exploited in continuous hydrogenation [276–277]. The isotropic network created by the monolith allows the preparation of highly dispersed catalysts with good mechanical properties and catalytic activities [7]. In 2013, Galarneau et al. used such an immobilised Pd system for the semi-hydrogenations of 1,5-cyclooctadiene (COD, **256**) and 3-hexyn-1-ol (**258**). The system provided a constant 95% conversion and 90% selectivity to the corresponding monoalkene **257** over 70 hours of runtime, and 85% conversion, 70–80% selectivity for the hydrogenation product **259** over 7 hours (Scheme 61) [277]. The authors claimed the selectivity to **259** could be improved via a silanol group deactivation treatment of the support.

Further examples of semi-hydrogenation using supported Pd nanoparticles have also been developed by Barbaro et al. A titanate nanotube (TiNT) support was explored, with the catalyst being prepared through an ion-exchange/in situ-reduction process. This allowed for the generation of well-dispersed nanoparticles with narrow diameter distribution, essential to the selectivity of the catalyst system. As such compound **259** was obtained at 83% conversion and 82% selectivity (TOF = 850 h^−1^, STY = 0.83 kg L^−1^ h^−1^) [278].

**Scheme 62:**
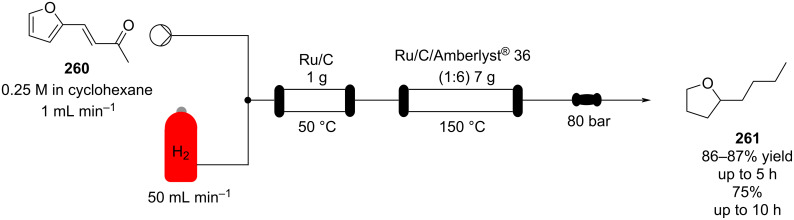
Continuous synthesis of biodiesel fuel **261** from lignin-derived furfural acetone (**260**).

In 2019, the Vorholt and Leitner groups developed a mini-plant for continuous hydrogenation of the lignin-derived building block furfural acetone (**260**) to 2-butyltetrahydrofuran (**261**), a promising biodiesel fuel. The flow process consisted of two reaction steps: hydrogenation and hydrodeoxygenation. For the first step, a Ru/C catalyst was employed, whereas in the second step, a sulfonic resin (Amberlyst^®^ 36) was added as the acid catalyst (Scheme 62). The apparatus was operated efficiently for up to 5 hours, yielding **261** in a consistent 86–87%. A slight decrease of yield to 75% was detected during the following 5 hours of runtime due to gradual catalyst deactivation [279]. The authors claimed the water formed during the sequence is absorbed onto the support, reducing the active surface area of the catalyst in the second step. Obviously, it would be relatively easy to build a secondary exchangeable cartridge into the system to allow cleaning and reactivation whilst maintaining continuous operation.

**Scheme 63:**
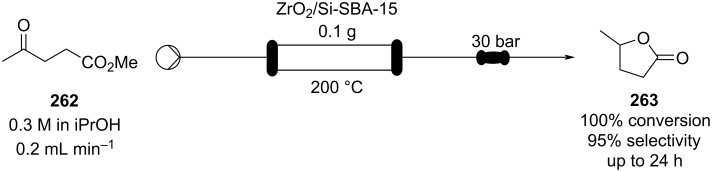
Continuous synthesis of γ-valerolacetone (**263**) via CTH developed by Pineda et al.

Pineda et al. optimised continuous-flow catalytic transfer hydrogenation (CTH) for the synthesis of γ-valerolactone (**263**) from lignin-derived methyl levulinate (**262**) in flow (Scheme 63) [280–281]. One of the systems operated with a mesoporous acidic material (Si-SBA-15) and iPrOH as the hydrogen donor. When a stream of **262** is heated at 200 °C and passed over the catalyst 100% conversion and 95% selectivity to the desired product **263** was achieved. The catalyst was able to operate for over 24 hours with no substantial change in productivity giving a throughput of 30 mmol g^−1^ h^−1^ [280].

**Scheme 64:**
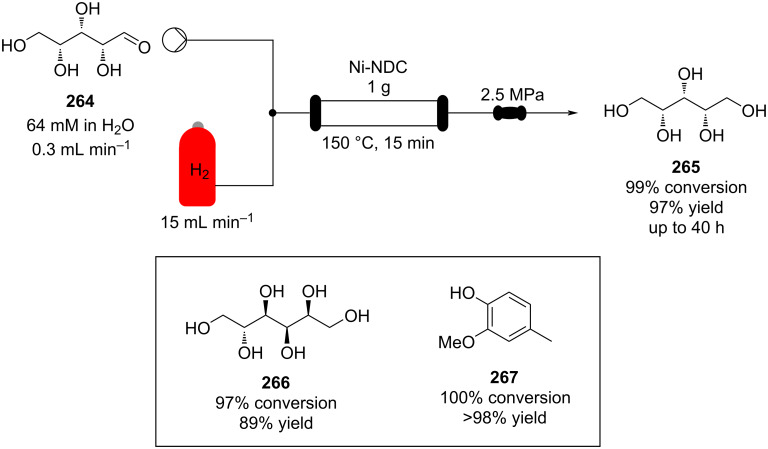
Continuous hydrogenation of lignin-derived biomass (products **265**, **266**, and **267**) using a sustainable Ni-NDC catalyst.

Recently, an alternative eco-friendly supported Ni-based catalyst has also been developed for the continuous hydrogenation of lignin-derived biomass sources such as glucose, xylose, and vanillin [282]. The heterogeneous catalyst was prepared from semolina, urea, glucose, and water to form after processing a nitrogen-doped carbon (NDC) support. The nickel nanoparticles were then dispersed via wet impregnation. The thus developed catalyst was found to be highly active and selective for water-based hydrogenation in continuous-flow. The resulting Mott–Schottky heterojunction formed between the metal and the NDC support provides high catalyst stability and enhanced activity (Scheme 64). The system could be operated for up to 40 hours without any changes and negligible metal leaching (total 11% inductively coupled plasma, ICP).

**Scheme 65:**
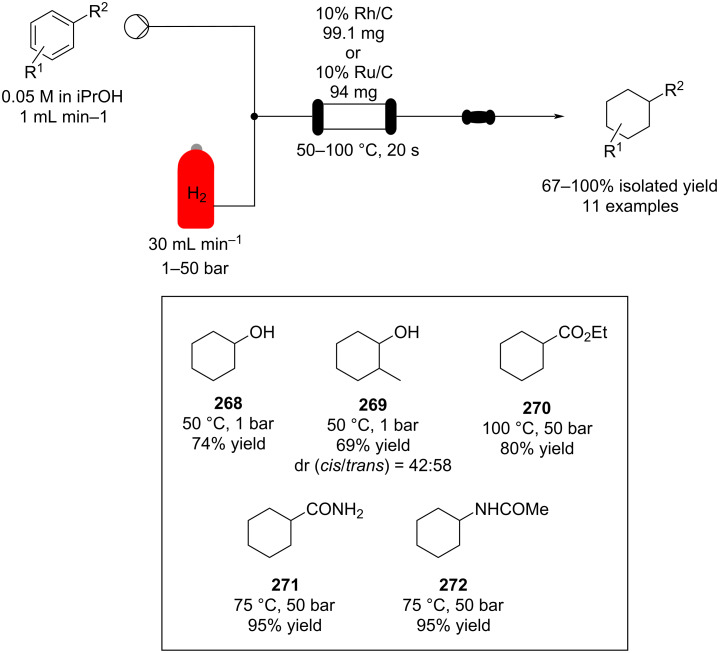
Ru/C or Rh/C-catalysed hydrogenation of arene in flow as developed by Sajiki et al.

Hydrogenation of arenes and heteroarenes have also been carried out in flow. As shown earlier, selective reduction of aromatic systems is a key transformation but typically requires harsh conditions due to the aromatic stabilisation. In 2015, the Sajiki and Monguchi group developed a rhodium/ruthenium-catalysed hydrogenation in flow able to operate under relatively mild conditions (Scheme 65). A solution of the arene in iPrOH was mixed with hydrogen and the stream directed through a 30 mm long cartridge packed with the catalyst and heated in a range of 50–100 °C. The system was shown to be efficient for several substrates, however, on some examples high pressure was still required to obtain full conversion. The authors claimed that more concentrated solutions reduced the effective contact with the catalyst thereby lowered the conversions (from 100% to 52%) [283].

**Scheme 66:**
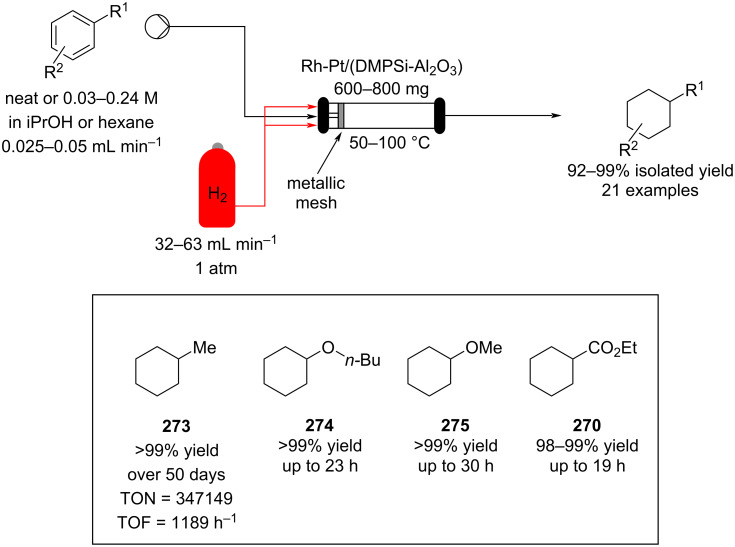
Polysilane-immobilized Rh–Pt-catalysed hydrogenation of arenes in flow by Kobayashi et al.

In a later publication, the Kobayashi group developed a polydimethylsilane/alumina-encapsulated Rh–Pt bimetallic nanoparticle catalyst, Rh–Pt/(DMPSi–Al_2_O_3_) [284]. In the flow system, the two streams (gas and substrates) were introduced in the column through a double-layer structured injector. To improve the initial gas–liquid mixing, a plug of metallic mesh filter was placed before the column packed with the pregenerated catalyst. The flow apparatus was tested on 21 examples; highlighted examples are shown in Scheme 66. The hydrogenation system was left running for over 50 days (TON = 347,149) and showed no signs of deactivation. The authors claimed the activity of the catalyst was over five times higher in flow compared to batch.

The same support and encapsulation process was subsequently used to immobilise Pd nanoparticles and the flow system was found to be highly suitable for the hydrogenation of compound **222** (TON = 5024, TOF = 1004 h^−1^) [285–286]. This hydrogenating system proved to be useful for the multistep synthesis of rolipram (**173**), previously described in Scheme 38.

**Scheme 67:**
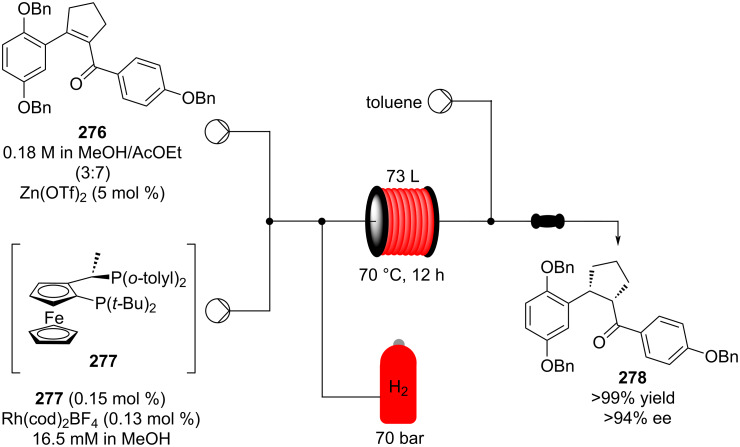
High-pressure in-line mixing of H_2_ for the asymmetric reduction of **278** at pilot scale with a 73 L plug flow tube rector (PFR) coil used.

Due to their importance the scale up of hydrogenations have been explored by numerous industrial as well as academic organisations. A pilot scale continuous-flow synthesis of LY500307 (**278**), an estrogen receptor β-agonist, using an asymmetric hydrogenation was performed at Eli Lilly & Co. (Scheme 67) [287].

**Figure 9:**
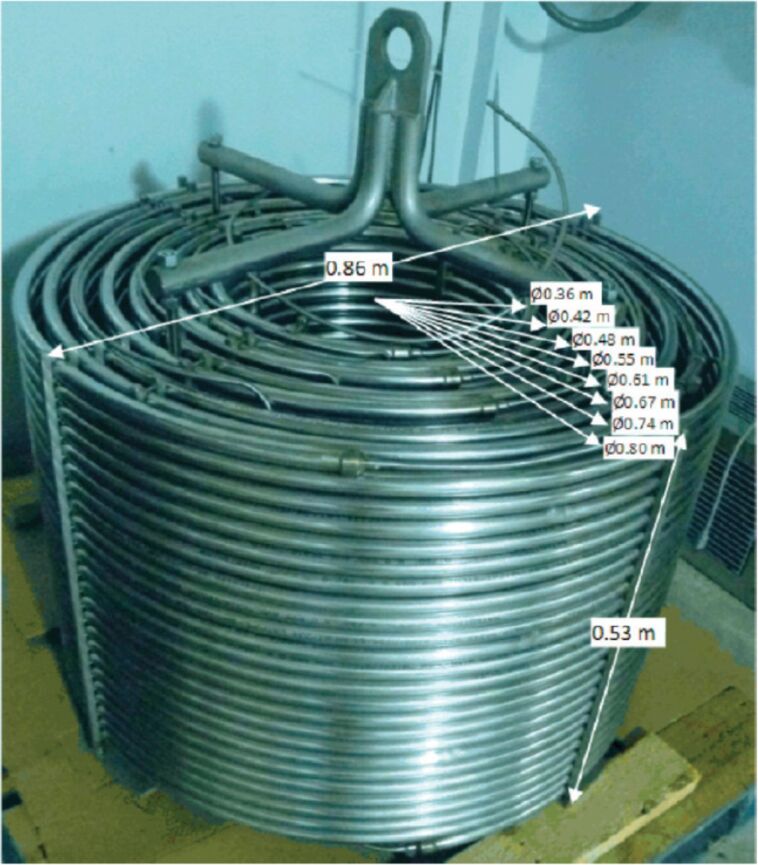
Picture of the PFR employed at Eli Lilly & Co. for the continuous hydrogenation of **278** [287]. Reprinted with permission from [287]. Copyright 2012 American Chemical Society.

A simple T-piece connector was used to introduce the gas to the liquid stream at an elevated pressure of 70 bar. During the optimisation, the starting material solubility was problematic and thus required more than 35 L of solvent to fully solubilise a 3.6 kg batch (0.18 M). A Rh–Josiphos catalyst at a loading of 0.05 mol % was found to be sufficient for the homogeneous hydrogenation. The output flow was directed into a 73 L plug-flow tube reactor (PFR) (Figure 9) maintained at 70 °C allowing a residence time of 12 hours and enabling excellent yields with high ee (>94%). This setup allowed a processing rate of 130 L d^−1^ of the starting material **276** stock solution, equating to a productivity of over 10 kg d^−1^ of the hydrogenation product **278**.

Solubility would only rarely present an issue when considering most fragrance ingredients and their intermediates which tend to be liquid at room temperature, making them readily ‘flowable’, subject to viscosity*.* Interestingly, this also makes fragrance chemicals highly suited to operation under solvent-free conditions (SFC) or at very high concentrations in flow.

**Scheme 68:**
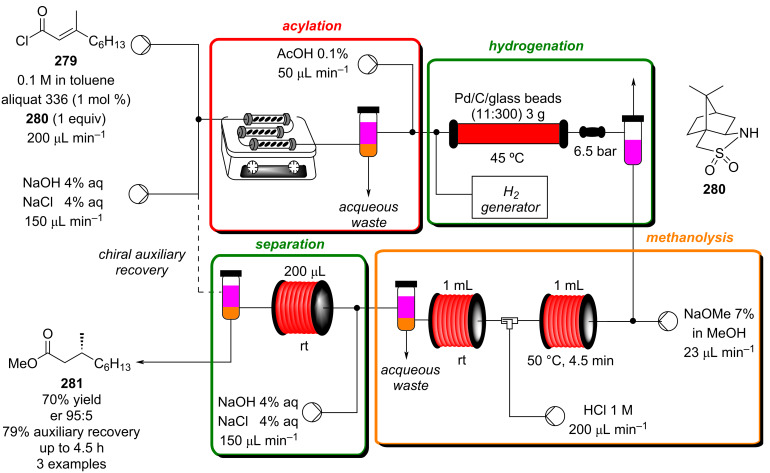
Continuous-flow asymmetric hydrogenation using Oppolzer's sultam **280** as chiral auxiliary.

Despite the enormous range of catalysts developed for asymmetric hydrogenations, the use of chiral auxiliary methods are still commonly utilised in bulk chemical manufacture [288–289]. This can be attributed to their high robustness and selectivity; however, the need for additional steps and stoichiometric reagents raises concerns about their green and economic consideration. In 2018, Newman and his group addressed such concerns by developing an asymmetric hydrogenation of α,β-unsaturated acids using Oppolzer’s sultam **280**. By telescoping the three reaction steps, the preparation times reduces to 30 minutes and the auxiliary recycling efficiently improved compared to batch (79% recovery) [290]. The assembled flow system consists of 4 steps: acetylation, hydrogenation, methanolysis, and product/auxiliary separation (Scheme 68).

The use of PTC biphasic acylation is performed in an oscillating reactor column. The three columns were placed in series on a stirring plate and filled up with oscillating magnetic bars which created a turbulent flow increasing the phase mixing. After separation, acetic acid was mixed with the flow stream to reduce the pH and avoid Pd/C catalyst deactivation by basic conditions. The hydrogenated intermediate amide was then deprotected using sodium methoxide and HCl as a quencher. The chiral auxiliary was recovered by selective deprotonation and continuous extraction. The apparatus allowed the recovery of Oppolzer’s sultam, which can be either isolated and crystallised or cycled back in the flow system where it could be reused eight times (4.5 hours of runtime).

#### Oxidation and related transformations

**Scheme 69:**
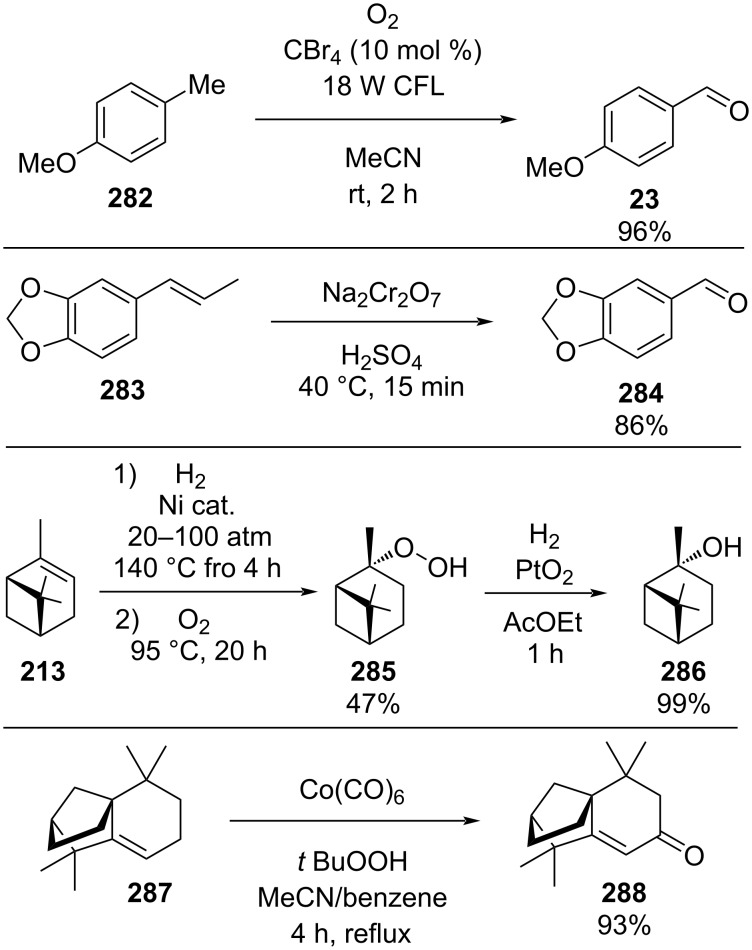
Some examples of industrially important oxidation reactions in the F&F industry. CFL: compact fluorescence light.

The formation of aldehydes/ketones from alcohols or other functionalities is powerful when considering the dissonant olfactory characteristics of products comprised of such moieties (Scheme 69).

Examples of such oxidation reactions include the synthesis of *p*-anisaldehyde (**23**) from anisole (**282**) [291], the formation of piperonal (**284**) from isosafrole (**283**) [292–293] and the generation of pinane hydroperoxide (**285**) from *cis*-pinane (the hydrogenation product of α-pinene (**213**)) [294]. Additional procedures such as the oxidation of isolongifolene (**287**) to isolongifolenone (**288**) using *tert*-butyl hydroperoxide and cobalt hexacarbonyl [295] are also important industrial sequences.

The disposal of metal waste streams such as those containing manganese and chromium oxides are heavily regulated and so recycling/recovery is necessary. Current industrial focus is now being increasingly placed on finding greener oxidation alternatives, such as the use of elemental oxygen. Indeed, many oxidation protocols rely on the use of gaseous O_2_, so economic and safety implications addressed in the previous section are also applicable here. Like with H_2_, in-line mixing of O_2_ is possible and it has also been successfully incorporated into the tube-in-tube reactor setups.

**Scheme 70:**
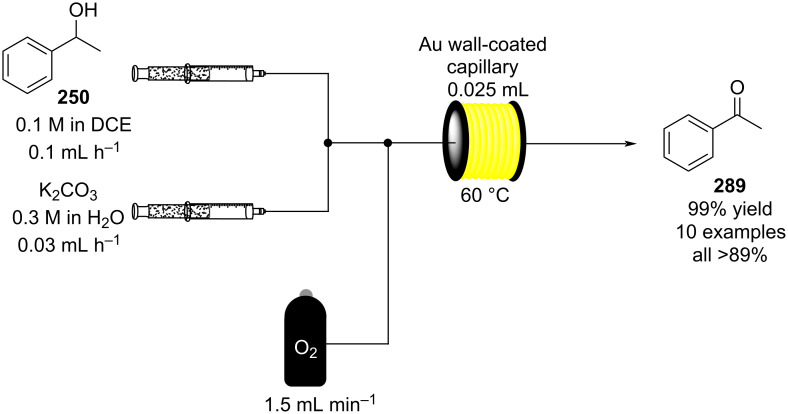
Gold-catalysed heterogeneous oxidation of alcohols in flow.

Aerobic oxidation in flow under both hetero- and homogeneous catalysis conditions has been extensively reviewed with special emphasis on the improvement in safety [296–297]. In 2009, the use of a gold wall-coated capillary reactor for the oxidation of alcohols with molecular O_2_ was reported (Scheme 70) [298].

The column was prepared by attaching microencapsulated gold onto amine functionalities within a modified polysiloxane capillary performed at elevated temperatures. The optimisation of the oxidation process was carried out on 1-phenylethanol (**250**) and the established conditions were then applied to 9 additional substrates. Good yields were obtained in all cases except for the oxidation of benzyl alcohol which required a column coated with a bimetallic Pd/Au matrix. Despite the low flow rates necessary and subsequent low throughput, the column was found to be highly robust with no Au leaching even after four days of continuous processing.

**Scheme 71:**
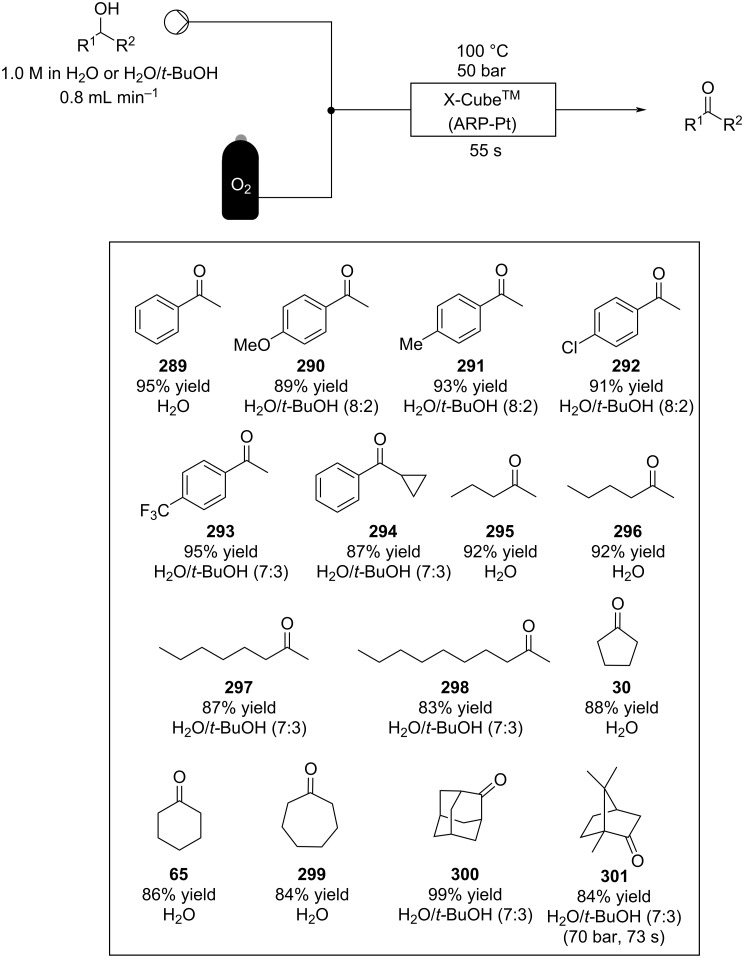
Uozumi’s ARP-Pt flow oxidation protocol.

Heterogeneous catalyst systems with improved throughput have subsequently been developed. Uozumi and co-workers reported a continuous process in which a column reactor containing platinum nanoparticles dispersed on an amphiphilic polystyrene–poly(ethylene glycol) resin (ARP-Pt) was used to effect oxidation of alcohols (Scheme 71) [299]. By using an X-Cube^TM^ reactor, much shorter residence times and higher throughputs were possible employing this catalytic system. In-line mixing of the O_2_ (50 bar) was used with column contact times of less than one minute, effecting full conversion of various alcohols evaluated. The reactor was additionally used for a range of 28 aldehydes, all of which gave the corresponding carboxylic acids in good yields. Other examples of heterogeneous aerobic oxidation of alcohols in flow include the use of a Ru(OH)_x_/Al_2_O_3_ catalyst packed bed reactor [300–301] and carbon black-stabilized polymer-incarcerated gold/platinum bimetallic nanocluster catalysts (PI-CB/Au–Pt) [302]. In-line oxygen delivery has also been performed using the tube-in-tube reactor system whilst making use of a downstream packed bed Au−Pd/TiO_2_ PTFE reactor [303]. Metal organic framework (MOF) have also been considered as potential candidates for heterogeneous catalysts due to their high surface area and porosity. Flow systems exploring this area have been developed and recently reviewed by Garcia and Dhakshinamoorthy [304].

**Scheme 72:**
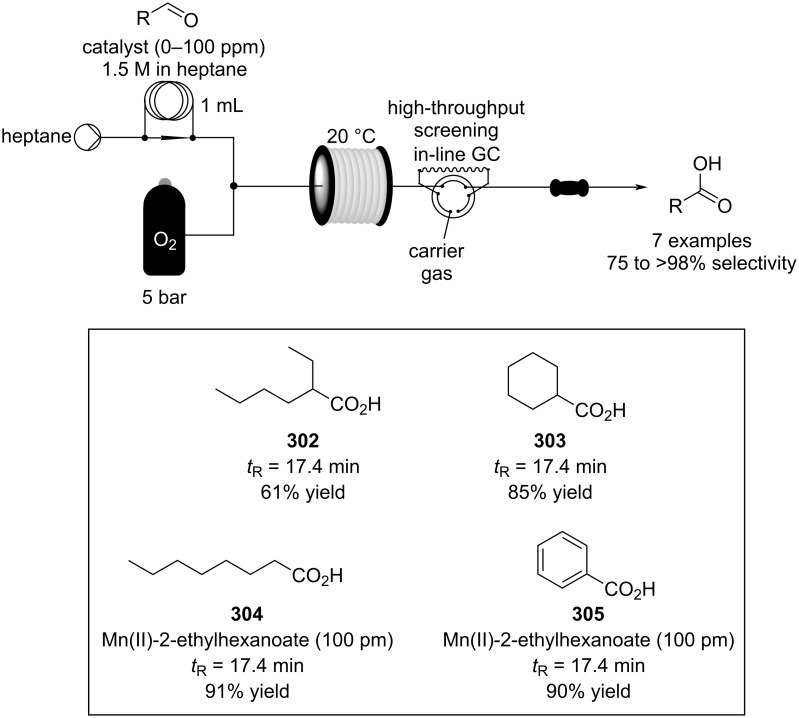
High-throughput screening of aldehyde oxidation in flow using an in-line GC.

There has also been significant effort directed towards systems enabling homogeneous aerobic flow oxidation. The work conducted by Favre-Réguillon et al. has revealed that the autoxidation of aldehydes in flow using elemental O_2_ is possible with and without the use of a catalyst (Scheme 72) [305–306]. In-line O_2_ mixing at 5 bar was used and the incorporation of in-line GC–MS analysis allowed for high-throughput screening, facilitating rapid optimisation of the reaction conditions. The addition of manganese and chromium salts enhanced conversions and selectivity towards the carboxylic acid such that generation of the problematic Criegee intermediate was almost completely avoided.

In 2020, Kappe and co-workers employed the aerobic oxidation of aldehydes to acids to develop a de novo synthesis of γ-nitrobutyric acids, key intermediates of several GABA derivatives. The authors described a highly effiencient and selective two-step telescoped synthesis with productivities ranging between 2.77 and 3.14 g h^−1^ [307].

**Scheme 73:**
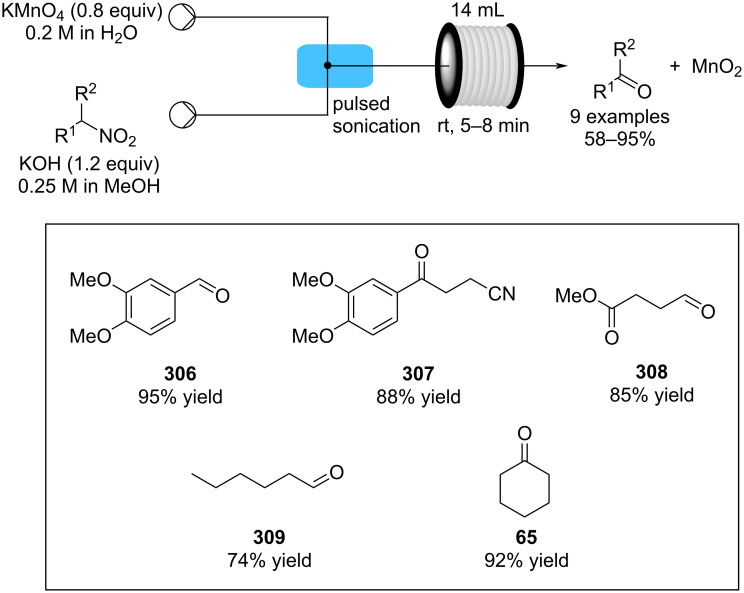
Permanganate-mediated Nef oxidation of nitroalkanes in flow with the use of in-line sonication to prevent reactor clogging.

A method for performing permanganate-mediated oxidation of alcohols and aldehydes to carboxylic acids as well as the Nef oxidations of nitroalkanes was developed in 2010 [308], in this process, ultrasound was employed to aid with the continuous processing of downstream MnO_2_ slurries which prevented fouling of the reactor (Scheme 73). This was one of the early examples of overcoming the potential clogging issue when solids are produced in flow as part of the product stream. Sonication or mechanical agitation have subsequently become a common tool to prevent accumulation and blockage in a flow channel [44–48].

**Scheme 74:**
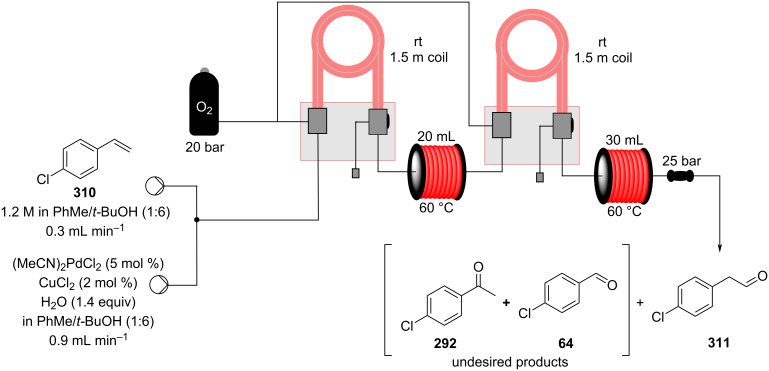
Continuous-flow aerobic anti-Markovnikov Wacker oxidation.

In 2013 a continuous-flow aerobic anti-Markovnikov Wacker oxidation was reported by Ley and Bourne (Scheme 74) [309]. The precise control of reaction conditions afforded by the microreactor system and the tube-in-tube reactor technology made it possible to use gaseous O_2_ for the oxidation of olefins to aldehydes while avoiding overoxidation. Selectivity was fine-tuned by systematic modification of the reaction parameter (oxygen pressure, H_2_O equivalents and reactor temperature), resulting in a system that was used for the selective oxidation of 12 different functionalised styrene derivatives (77–92% selectivity). A larger scale production of 2-(4-chlorophenyl)acetaldehyde (**302**), utilising a “double dosing” of O_2_ (Scheme 74), boosted the throughput seven-fold to 21.6 mmol h^−1^, yielding 17.6 g of the aldehyde **311** after 6 hours of processing.

In 2019, Gupton et al. explored the use of an X-Cube^TM^ reactor for the aerobic oxidation of benzaldehyde (**17**) using a commercial palladium catalyst. When the tube-in-tube apparatus was placed in-line, the conversions increase 9-fold using oxygen (31%) and 5-fold using air (10%) as the oxidant. The authors claimed high selectivity (100%) and high productivity (STY = 22000 g L^−1^ h^−1^ using air, 64000 g L^−1^ h^−1^ using oxygen) at lower pressure (2.8 bar) compared to the results acquired using higher pressures (25 bar) [310].

**Scheme 75:**
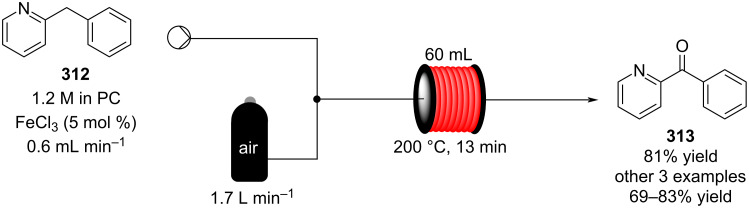
Continuous-flow oxidation of 2-benzylpyridine (**312**) using air as the oxidant.

Other examples of homogeneous gas–liquid aerobic oxidation in flow include the direct oxidation of 2-benzylpyridines (Scheme 75). The system used employed air as the oxidant and propylene carbonate (PC) as a green solvent. The authors pointed out the usage of PC and the flow system allowed them to work at high temperature reducing the reaction time to 13 minutes, compared to 1 hour in batch mode [311].

**Scheme 76:**
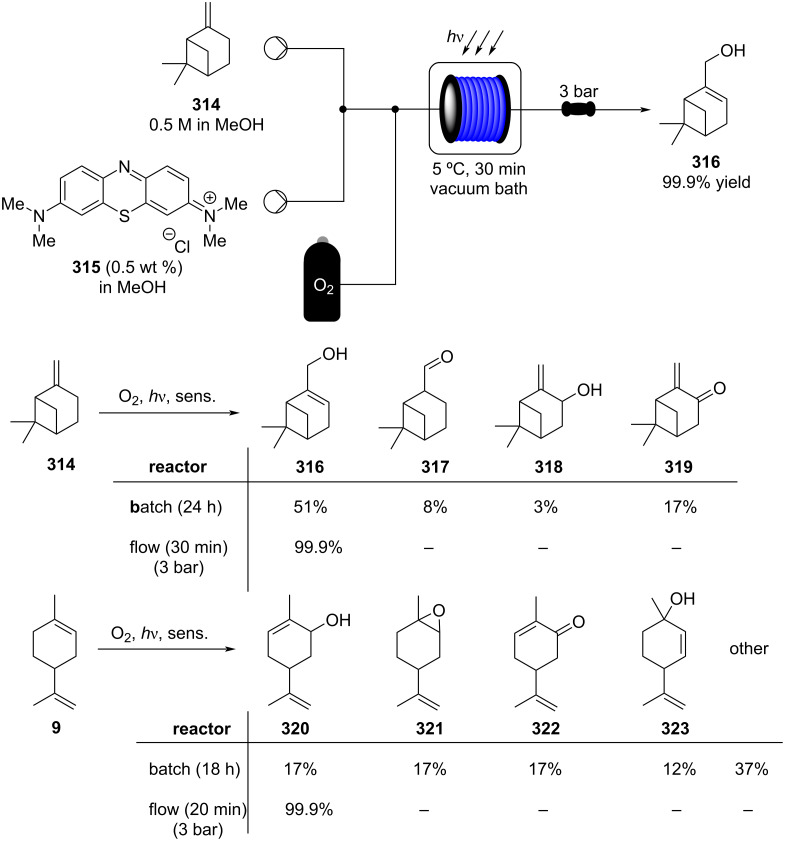
Continuous-flow photo-oxygenation of monoterpenes.

Of particular interest to many chemists in the industry is photo-oxidation in flow. Indeed, a number of such transformations have already been investigated on fragrance derivatives. As with heat transfer, the high apparent surface contact area in flow makes it highly compatible with procedures in which light/radiation transmittance is key. The continuous-flow photo-oxygenation of hexamethylbenzene [312] and monoterpenes [313] has been demonstrated by Park et al. Several additional substrates such as β-pinene (**289**), α-pinene (**188**), δ-limonene (**9**), (−)-citronellol (**299**) and α-terpinene have all been explored in various oxidative transformations and comparisons to batch protocols have been made (Scheme 76). As is typical of photo-oxygenation processes, batch experiments required longer reaction times and generally gave poor selectivities. The reaction of β-pinene (**314**), for example, gave the four products **316**–**319** upon treatment with oxygen and a sensitizer (desired product, 51%) after 24 hours in batch. However, the desired product (**316**) could be obtained exclusively in 99.9% yield after just 30 minutes in flow. Similar observations have been made for the formation of the cyclohexenol **320**, from δ-limonene (**9**), with a marked improvement upon reaction time, selectivity and yield observed in flow.

**Scheme 77:**
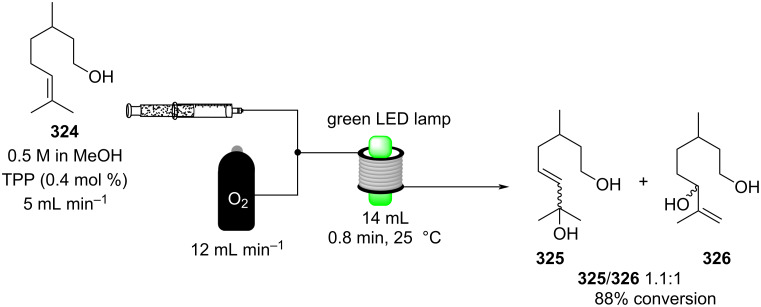
A tubular reactor design for flow photo-oxygenation.

A polydimethylsiloxane-based single channel microreactor has been engineered with direct in-line mixing of O_2_ via a T-junction or via presaturation of the reagent input solution [314]. After optimisation of the reaction conditions and profiling of the reactor, a tube-in-tube reactor approach was used for scale-up procedures, affording significant improvements over batch methods in terms of daily product output. Irradiation using a lamp within a vacuum bath (inner walls mirror coated) proved very effective in this instance. Additional work by Seeberger et al. [315] has demonstrated an approach based upon the original GSK-Booker–Milburn-type reactor [316], where the reactor coil surrounds the light source generating singlet oxygen in situ (Scheme 77). This design was successfully applied to the oxidation of substrates such as citronellol (**299**), α-pinene (**188**) and 2-methyl-2-butene.

**Scheme 78:**
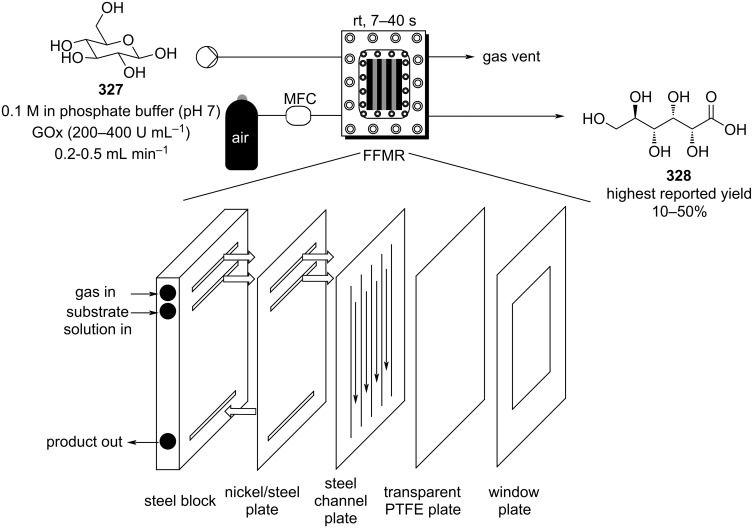
Glucose oxidase (GOx)-mediated continuous oxidation of glucose using compressed air and the FFMR reactor.

Enzymes have also been productively incorporated into flow systems, leading to novel technologies such as “packed-bed microbioreactors” [114,317–318]. Enzyme-catalysed gas–liquid reactions have been exploited in falling-film microreactor (FFMR) for example for the glucose oxidase (GOx)-mediated oxidation of β-ᴅ-glucose to gluconic acid (Scheme 78) [319]. The FFMR was designed to create a thin film interface of the substrate solution and gas (thickness in the range of 10–100 μm) leading to a high gas–liquid contact area. The high surface area to volume ratio also conveniently allows for efficient heat transfer in exothermic processes.

More recently, a continuous-flow aerobic oxidation of primary alcohols with a Cu(I)/TEMPO catalyst system was achieved (Table 7) [320]. This was developed as an alternative to a previously reported palladium-catalysed continuous-flow protocol [321], with the new Cu(I)/TEMPO methodology offering shorter residence times and decreased susceptibility to catalyst decomposition as well as being applicable to a broader substrate scope.

**Table 7:** Continuous-flow aerobic oxidation of primary alcohols with Cu(I)/TEMPO.

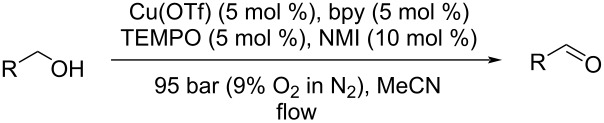					

Entry	Substrate	Product	Time (min)	*T* (°C)	Yield (%)

**1**	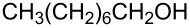 **329**	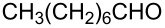 **330**	45	60	95
**2**	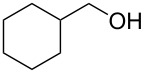 **331**	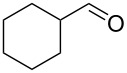 **332**	45	60	95
**3**	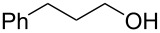 **333**	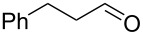 **245**	30	60	99
**4**	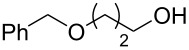 **334**	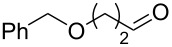 **335**	30	60	98
**5**	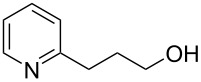 **336**	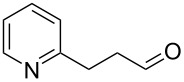 **337**	30	60	99
**6**	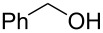 **16**	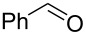 **17**	5	100	>90
**7**	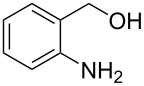 **338**	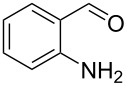 **339**	5	100	>99
**8**	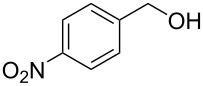 **340**	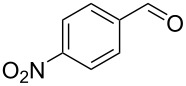 **50**	5	100	>99
**9**	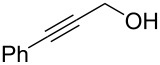 **341**	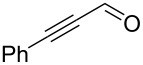 **342**	5	100	>99

Of the 9 substrates tested (Table 7), all gave yields of 95% and above and a 100 g-scale version based upon benzyl alcohol (**16**) oxidation was carried out over a 24 hour period with a sustained yield of >99%. To improve the safety of the process a 9% O_2_ in N_2_ mixture was used as the oxygen source thus avoiding the highly flammable oxygen and explosive composition.

**Scheme 79:**
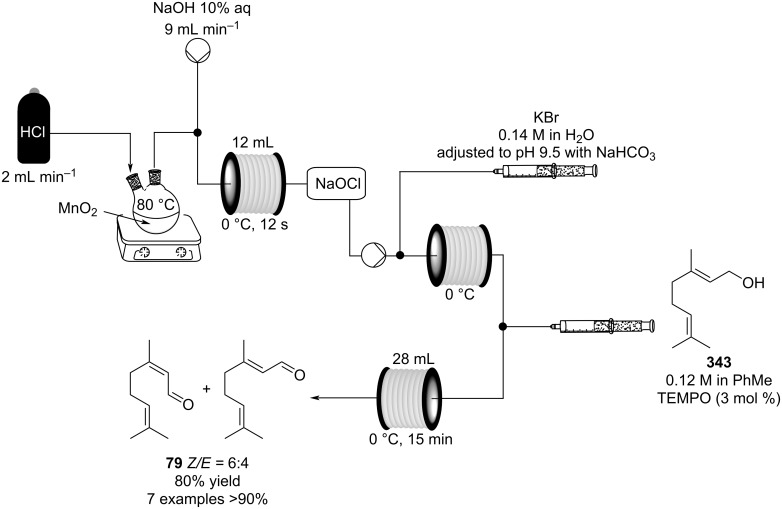
Schematic continuous-flow sodium hypochlorite/TEMPO oxidation of alcohols.

TEMPO has also been utilised as a transfer catalyst for selective alcohol oxidation using in situ-generated sodium hypochlorite as a stoichiometric oxidising agent [322]. Chlorine was produced by adding hydrochloric acid to solid MnO_2_ and the gas stream directed to react with a flow of aqueous sodium hydroxide to yield sodium hypochlorite. To prevent its rapid decomposition, a buffer solution was combined to adjust the pH to around 9.5 (Scheme 79). The individual flow rates were then set in order to generate a specified range of NaOCl concentration, which had the correct strength to avoid the production of overoxidation products. In this setup, the alcohols along with TEMPO were mixed with the oxidising agent (NaOCl) at 0 °C. A mixture of geranial ((*Z*)*-***79**) and neral ((*E*)*-***79**) in a 6:4 ratio was produced in 80% yield, additionally 7 other aldehydes were synthesized in moderate to good yields.

**Scheme 80:**
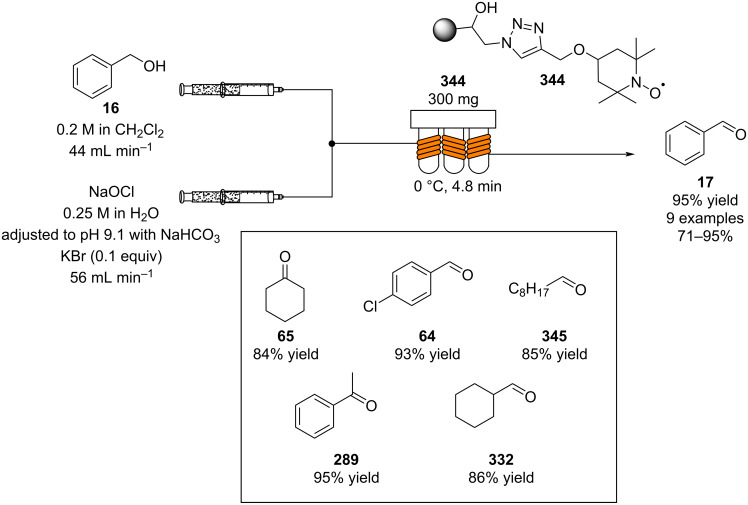
Oxidation using immobilised TEMPO (**344**) was developed by McQuade et al.

Immobilisation of TEMPO ((2,2,6,6-tetramethylpiperidin-1-yl)oxyl) has also been explored. In 2009, TEMPO was efficiently tethered on an AMBERZYME^®^ oxirane resin [323]. The catalyst was then introduced into a fluoroelastomeric tube and wrapped around metal bars used as a heat sink cooling system. The reaction media was formed of two phases: organic, and aqueous phase. The segmented flow produced provides enough phase interaction to allow a rapid oxidation of benzyl alcohol (**16**, 4.8 minutes). The use of the system resulted in efficient oxidation of many different types of aromatic and aliphatic alcohols, some highlighted examples are depicted in Scheme 80. Beneficially, the heterogeneous catalyst remains highly active even after hours of operation (up to 9 hours). Other TEMPO-immobilised systems have also been developed; Sato and Okuno et al. explored immobilisation on a polyethylene–polyacrylic acid polymer, and Smarsly et al. using mesoporous silica particles [324–325]. Each support strategy offers different processing advantages and limitations based upon pressure drop, loading and ease of preparation. Herman and his group developed a continuous triphasic aerobic oxidation which involved nitric acid as co-oxidant and employed a silica-immobilised TEMPO [326].

**Scheme 81:**
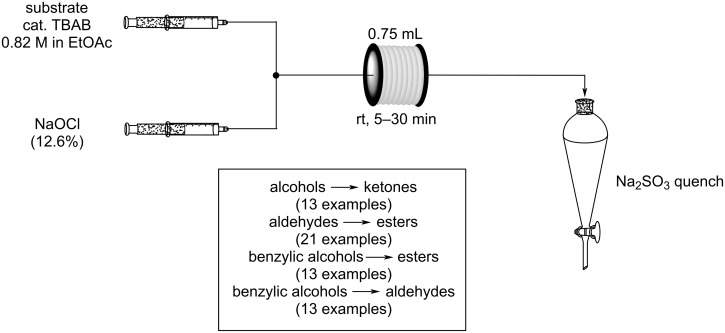
General protocol for the bleach/catalytic TBAB oxidation of aldehydes and alcohols.

Phase transfer catalysts (PTC) in biphasic oxidation reaction are well-known and described widely in the literature [327]. In fact, PTC-assisted oxidations have been comprehensively reviewed by Penso et al. in 2016. Flow examples have also been successfully conducted. As an example, the use of bleach and catalytic tetrabutylammonium bromide (TBAB) for the oxidation of alcohols and aldehydes in flow was reported by Jamison et al. in 2012 (Scheme 81) [328]. The simple setup and relatively safe reagent combination makes the approach particularly attractive [329].

**Scheme 82:**
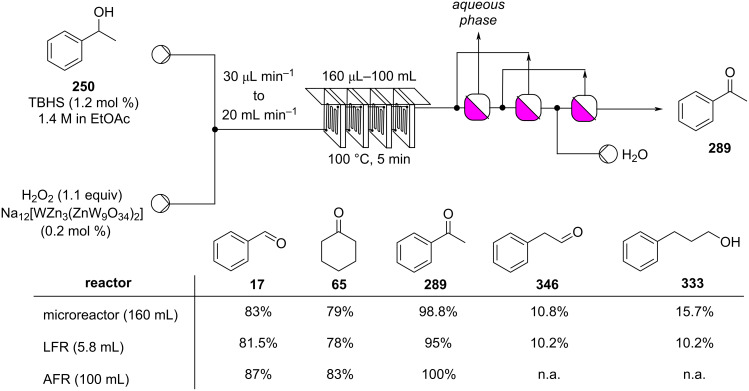
Continuous-flow PTC-assisted oxidation using hydrogen peroxide. The process was easily scaled up by different types of microreactors (LFR and AFR).

Continuous-flow biphasic PTC-assisted oxidations using NaOCl has also been explored by Jensen and his group using a Corning Low Flow reactor (LFRs) and advanced flow reactors (AFRs). These types of mesoscale device allowed easy scale-up of a process up to 4 g min^−1^ without losing efficiency [330]. The same group later developed a flow system using hydrogen peroxide which was able to quantitatively transform **250** into acetophenone (**289**, Scheme 82). The authors pointed out the simplicity of scaling up the process from an 160 μL microreactor to an 100 mL reactor (AFR) retaining the same processing efficiency [331]. To improve the phase separation, the authors employed a set of in-line PTFE membranes which enabled over 90% of the PTC to be extracted from the product.

Epoxidation in flow represents a potentially valuable tool for fragrance and flavour chemists with perceivable safety implications for the handling of hazardous or unstable epoxidising agents and reactive intermediates.

**Scheme 83:**
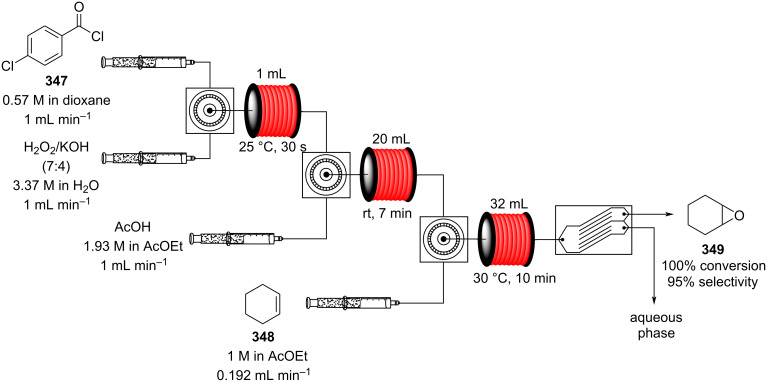
Continuous-flow epoxidation of cyclohexene (**348**) and in situ preparation of *m-*CPBA.

In 2016, Guo et al. addressed the problem of handling the hazardous epoxidising agent *m-*chloroperbenzoic acid (*m-*CPBA) by in situ preparation from the corresponding acyl chloride and potassium peroxide (Scheme 83) [332]. The group explored an engineered micromixer to increase mass transfer for the biphasic system. Further optimisation of the process allowed the yield of the epoxidising agent to be enhanced to 87%, and obtaining better selectivity than material provided by commercial suppliers. The setup was ultimately utilized for cyclohexene oxide preparation. Of particular value was that upon exiting the capillary tube reactor, the biphasic solution undergoes a continuous phase separation through an engineered device, which is based on the principles of inclined plate settler. The optimised flow apparatus allowed collection of 97% of the organic phase and isolation of cyclohexene oxide (**349**) in 100% conversion and 95% selectivity. The authors highlighted the advantage of the in situ preparation of *m-*CPBA, which cuts down the cost of a possible industrial production. The same group developed a similar system for the preparation of epoxidised soybean oil [333].

**Scheme 84:**
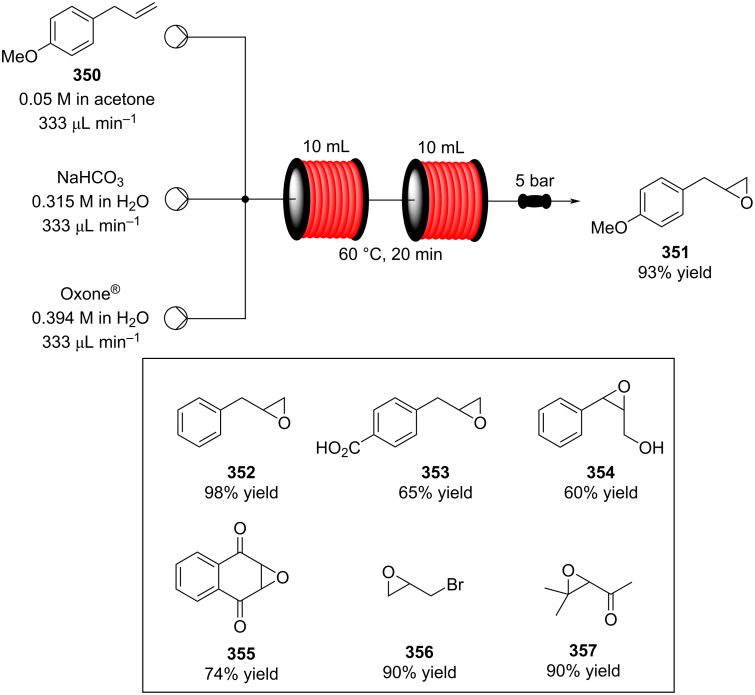
Continuous-flow epoxidation using DMDO as oxidant.

In situ formation of dimethyldioxirane (DMDO) has also been employed for the epoxidation of miscellaneous alkenes by McCluskey and his group in a flow based process [334] (Scheme 84). Acetone was used as the solvent in the alkene solution, and solutions of NaHCO_3_ and Oxone^®^ were directly mixed with the main stream. The author showed how important the temperature was, being maintained at 60 °C, no epoxide formation was detected below this critical temperature and a too high temperature generated multiple byproducts. The generated epoxides were then employed for creating a compound library of potential new pharmaceuticals but could be used in many different applications.

**Scheme 85:**
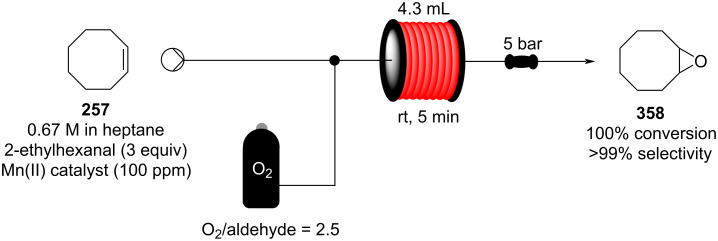
Mukayama aerobic epoxidation optimised in flow mode by the Favre-Réguillon group.

Mukayama-type aerobic epoxidation has also been implemented in flow mode by Favre-Réguillon and his group [335]. A segmented flow regime increases the mass transfer and allowed a reduction in the required reaction time to 5 minutes (Scheme 85). Additionally, the system requires lower catalyst loading compared to the batch approach (0.1 mol % vs 100 ppm) and quantitative conversion and high selectivity (>99%). The same system was also explored for the epoxidation of several fatty acids [336].

Various zeolite supports have found use as heterogeneous catalysts in flow through filled reactor cartridges and channels. Wan et al. used a titanium silicate zeolite micro-engineered reactor for the epoxidation of 1-pentene in flow [337]. However, the system showed deactivation over prolonged usage equating to ≈100 hours (≈500 mg of starting material), meaning the catalyst had to be eventually replaced. Similarly, Okrut et al. developed a crystalline Ti-based zeolite for the epoxidation of 1-octene (**650**) with ethylbenzene hydroperoxide (EBHP) [338]. The catalyst was designed as a valid replacement for the silica supported Ti-based catalyst employed from Shell in propylene oxide preparation [339]. The Shell catalyst allows to convert the alkene up to 84% and higher temperatures are required to complete the transformation, leading to reduced catalyst selectivity and deactivation. The zeolite-based Ti catalyst proved to be more robust and active than the amorphous silica-supported one, showing no evidence of deactivation over 72 hours of runtime [338]. After use, the zeolite can be calcinated to regenerate its activity.

**Scheme 86:**
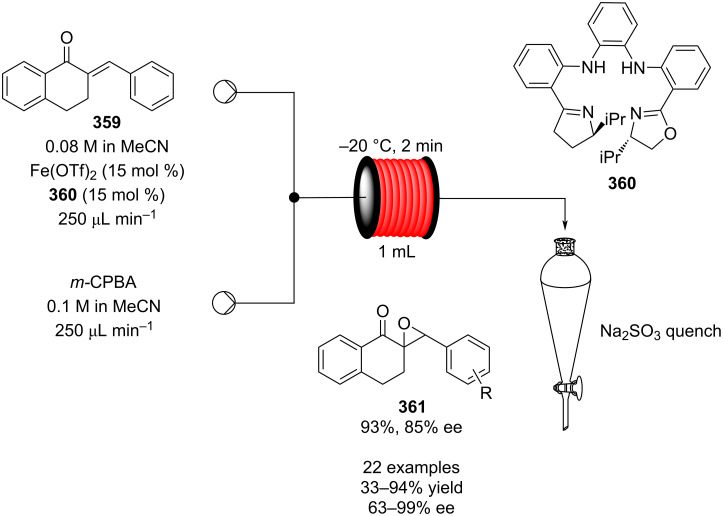
Continuous-flow asymmetric epoxidation of derivatives of **359** exploiting a biomimetic iron catalyst.

In 2015, Chen and Gao et al. demonstrated one of the few asymmetric epoxidations in flow exploiting a previously described biomimetic iron catalyst with ligand **360** (Scheme 86) [340–341].

The system was tested on several electron-deficient olefin derivatives **359** and the corresponding epoxides **361** were obtained in good to excellent yields (52–90%) and moderate to good selectivity (63–92% ee). The apparatus presented some advantages over the batch reaction such as reduced reaction times (2 minutes vs 2 hours) and decreased peroxyacid equivalents (1.25 equiv vs 2 equiv).

**Scheme 87:**
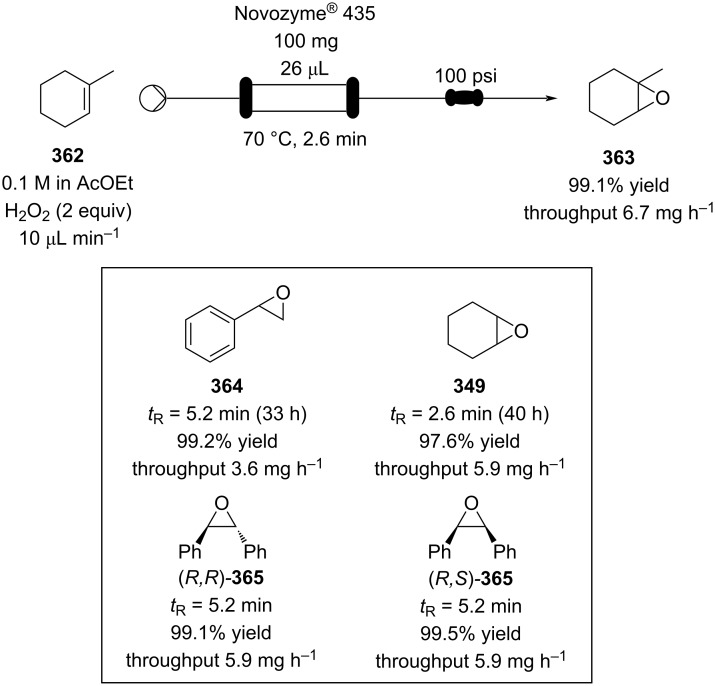
Continuous-flow enzymatic epoxidation of alkenes developed by Watts et al.

A micro-structured PEEK (polyether ether ketone) reactor was developed in 2009 for the enantioselective epoxidation of chalcone (**235**), catalysed by poly-ʟ-leucine [342]. A staggered herringbone micromixer design generated efficient mixing, however, productivity amounted to only 0.5 g day^−1^ due to the small reactor dimensions. In the same year, Watts and Wiles et al. developed a continuous-flow system using a commercially available lipase supported on a macroporous acrylic polymer (Novozyme^®^ 435) for epoxidation [343]. In this example, as in many others, the authors demonstrated the use of flow allows for intensified reaction conditions to be used, increasing throughput and beneficially preventing enzyme deactivation (Scheme 87). Indeed, higher concentrations of hydrogen peroxide were tolerated (0.3 M vs 0.05 M) alongside higher reaction temperatures (rt vs 70 °C). The system could be efficiently operated for over 25 hours without any detrimental effects.

**Scheme 88:**
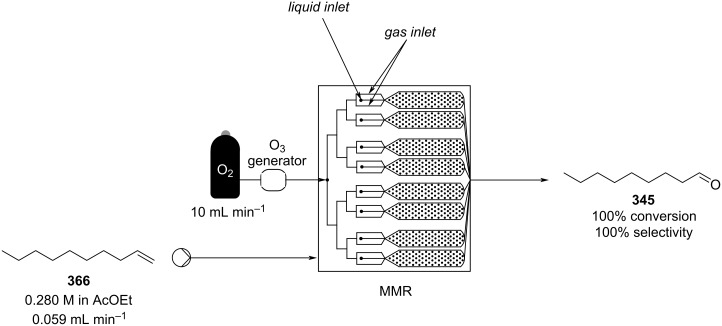
Engineered multichannel microreactor for continuous-flow ozonolysis of **366**.

Ozonolysis is a related electrophilic oxygenation process which carries with its huge potential safety benefits with regards to handling explosive and hazardous reaction intermediates meaning likewise it is highly beneficial to perform in flow. In 2006, Jensen and his group developed an engineered multichannel microreactor for the ozonolysis of 1-decene (**366**) [344]. The reactor was fabricated in Pyrex^®^ with silicon posts placed in the channels to increase the pressure drop, and allow liquid–gas interactions. The apparatus was explored for the oxidations of three different functional groups; phosphites, amines, and alkenes. The authors pointed out a residence time as short as 0.32 seconds was enough to efficiently convert 1-decene (**366**) to nonanal (**345**, Scheme 88).

**Scheme 89:**
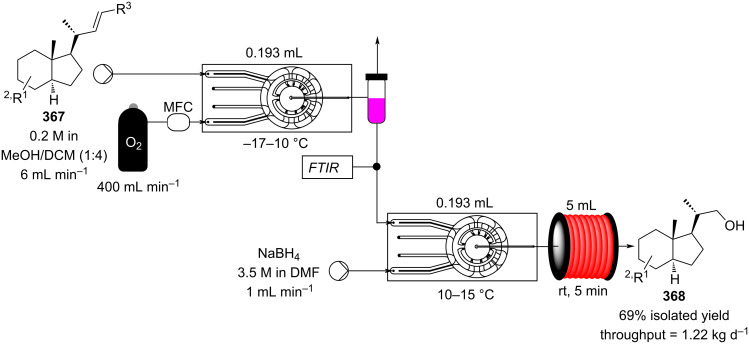
Continuous-flow synthesis of the vitamin D precursor **368** using multichannel microreactors. MFC: mass flow controller.

Jähnisch and co-workers also developed a micro-structured device for ozonolysis, which was employed for the preparation of a vitamin D precursor, **368** [345]. The system exploited a 5-channel micromixer called “cyclone”, which allows a high mixing effect increasing the flow rate. The ozolonysis step was coupled with a reductive one and the system could produce 1.22 kg of desired material per day in 69% overall isolated yield. The same group also developed the ozonolysis for 1-decene (**366**) and acetic acid 1-vinyl ester using a falling-film microreactor (FFMR) [346–347]. To monitor the conversion of the reaction, all these experiments were monitored using an on-line FTIR with an attenuated total reflectance (ATR) sensor (Scheme 89).

**Scheme 90:**
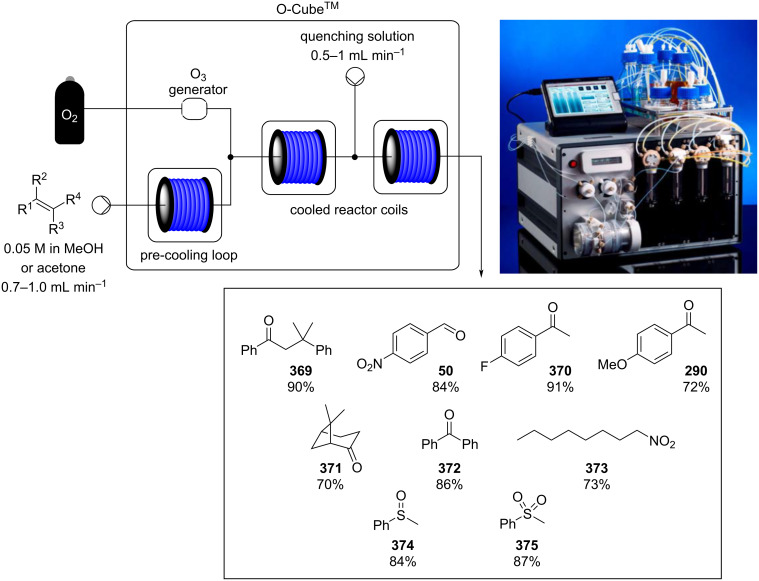
Continuous ozonolysis setup used by Kappe et al. for the synthesis of various substrates employing ThalesNano^®^ O-Cube™ (**50**, **290**, **369**–**375**) [348]. Reprinted with permission from [348]. Copyright 2011 American Chemical Society.

The general flow setup shown in Scheme 90 was used by Kappe et al. for the ozonolysis of various compounds, affording products **47**, **264**, and **344**–**348** all in good yields [348]. The ozonolysis was performed using a ThalesNano^®^ device called the O-Cube™. Ozone was generated in-line from electrolysis of O_2_ and was delivered through a PTFE frit to a precooled stream of starting material solution. The mixture was then run through a second reactor coil before being met by a stream of quenching solution. Conditions used were substrate specific and tuning of temperature and quenching solution could be used to achieve selectivity between sulfone **349** and sulfoxide **350**.

**Scheme 91:**
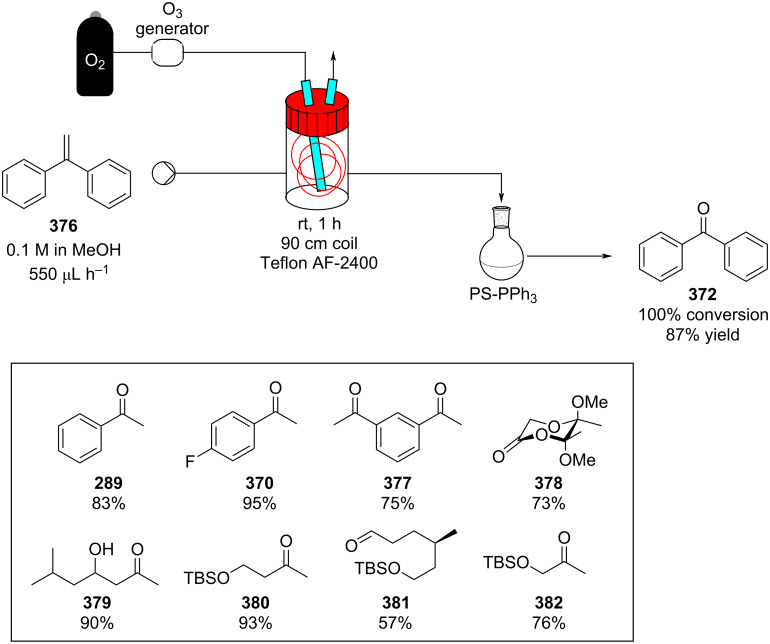
Continuous-flow apparatus for ozonolysis as developed by Ley et al.

In 2010, Ley and his group exploited a semi-permeable material (Teflon AF-2400) to build a simple and efficient flow apparatus for ozonolysis [349]. A coil of semi-permeable material was inserted into a glass bottle where ozone was flushed in, and a stream of alkene solution was pumped through the coil. The system was capable of oxidising different alkenes at room temperature in moderate to excellent yields and few examples are depicted in Scheme 91. After collection, the so formed ozonides were quenched using polymer-supported triphenylphosphine (PS-PPh_3_).

**Scheme 92:**
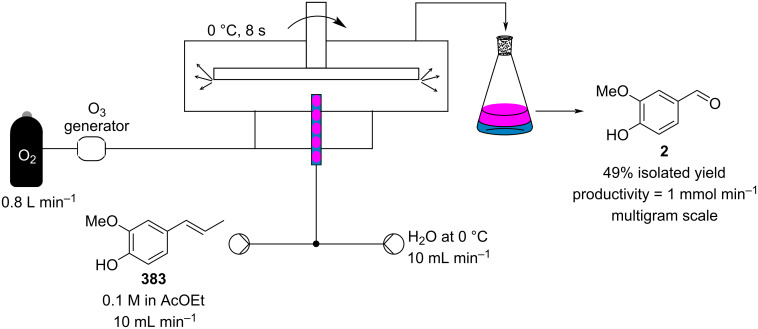
Continuous-flow ozonolysis for synthesis of vanillin (**2**) using a film-shear flow reactor.

In 2016, Tyler and his group explored the use of a film-shear flow reactor to enhance liquid–gas interaction [350]. The authors pointed out the possibility to achieve a high throughput, as the residence time was only a few seconds, and helped to avoid falling-film microreactor and the need for stoichiometric reducers. In fact, the system employed water as a mild reductant (Scheme 92), following a modification described by Dussault and Schiaffo [351]. The ketones and aldehydes formed could be easily isolated from the organic phase. However, this flow methodology is not applicable for alkylalkenes as they produce too unstable Criegee intermediates, which rapidly decompose to the ozonide.

Significant work in this area by Gavriilidis and co-workers [352–353] and Sun et al. [354] has further demonstrated the potential of performing ozonolysis reactions and handling their intermediates in flow. In 2017, Subramaniam et al. used static mixer to enhance mixing for the ozonolysis of several fatty acid esters [355]. They confirmed that scCO_2_ could be used as the solvent, and additionally, the authors developed a CSTR for continuous ozonide decomposition. This system consists of a Parr reactor set to a certain temperature, which was continuously fed by a stream of ozonide reaction mixture. For safety reasons, the reactor was kept under nitrogen atmosphere and filled only one third of its full capacity. After 20 minutes of residence time, the outlet stream was collected in an open container to vent the generated gas. The authors investigated a large range of temperatures (80 to 150 °C) finding highest selectivity at 80 °C.

As flow chemistry facilitates the use of in-line analysis, different mechanistic studies on ozone-driven oxidations of alkene compounds in air have been performed, leading to interesting results difficult to be obtained without a flow approach [62,77,356–358].

An alternative oxidative cleavage of olefins can be achieved through the use of molecular oxygen itself as the sole oxidant in a process catalysed by Pd^II^ [359]. Improvements to the process have been obtained with a flow reactor system compared with the batch equivalent decreasing the time of reaction and the catalyst loading. Undoubtedly, the incorporation of flow technology into transformations requiring oxygen as well as other gaseous reagents will become increasingly popular for the reasons discussed above.

**Scheme 93:**
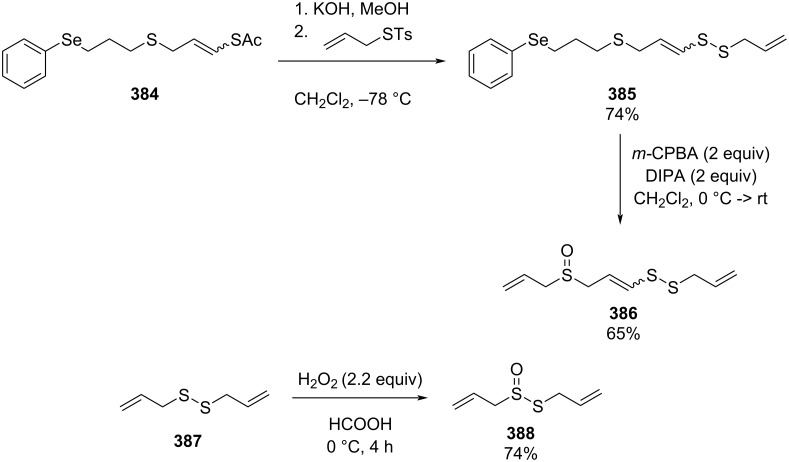
Examples of preparative methods for ajoene (**386**) and allicin (**388**).

Organosulfur compounds such as ajoene (**386**) and allicin (**388**) are key components in onion and garlic extracts. These products provide odour and some therapeutic benefits to these herbs, and consequently are important materials in F&F and pharmaceutical industry [360]. Different procedures for their synthesis have been disclosed so far and sulfide oxidation is described as the key step (Scheme 93) [361–364]. For instance, ajoene (**386**) can be prepared by hydrolysis and sulfenylation of the thioenaloate starting material **384** to prepare the intermediate **385**, which can then be oxidised to the desired material **386**. Allicin (**388**) could be prepared by oxidation of diallyl disulphide (**387**).

**Scheme 94:**
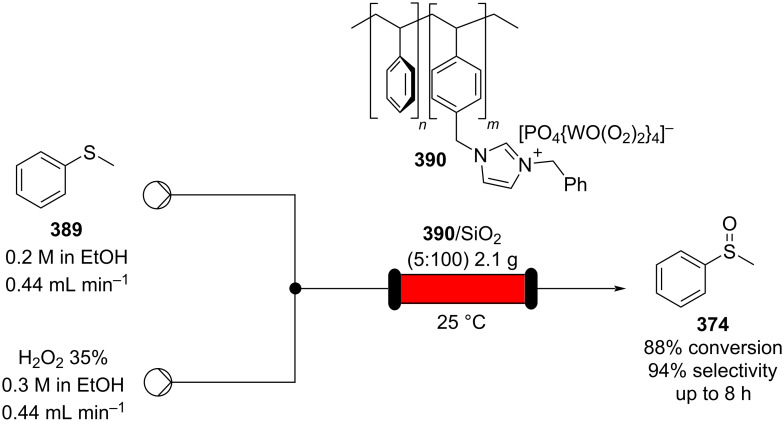
Continuous-flow oxidation of thioanisole (**389**) using styrene-based polymer-supported peroxytungstate catalyst **390**.

Over the years, many oxidising agents and conditions have been tested to selectively convert sulfides to the corresponding sulfoxides. However, due to their propensity for overoxidation, exact control of the reaction time and oxidant stoichiometry is of paramount importance to avoid sulfone formation, hence flow chemistry has been evaluated as a processing solution. While pursuing this work, a review on this subject has also been published by Colomer and co-workers [365]. In this area Doherty et al. have published widely on the application of peroxotungstates being immobilised on ionic resins that allow for selective oxidisation along with a low catalyst leaching. The application in flow enabled a lower catalyst loading and reduced residence times (Scheme 94) [366–367].

**Scheme 95:**
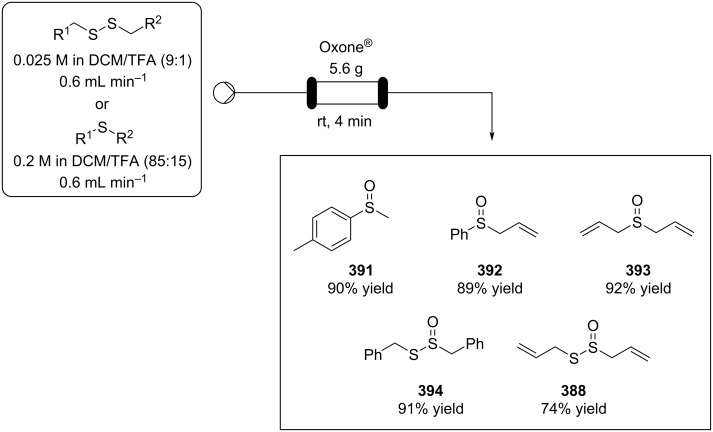
Continuous oxidation of thiosulfinates using Oxone^®^-packed reactor.

Along with the sulfoxides, thiosulfinates have been synthesised in flow using a packed-bed reactor filled with oxone as described by Wirth et al. (Scheme 95) [368]*.* This approach has been used to prepare very reactive metabolic sulfoxides and sulfenic acids present in garlic at increased scale.

**Scheme 96:**
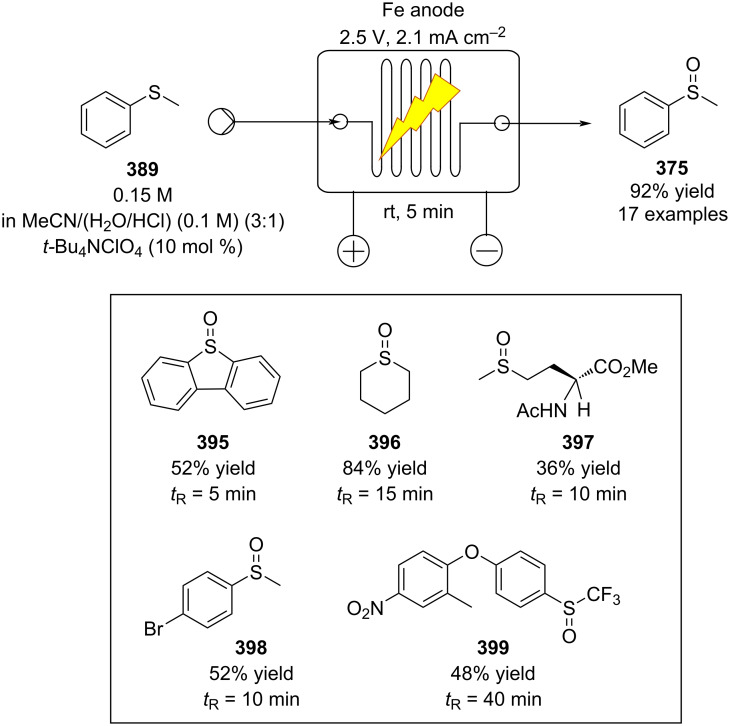
Continuous-flow electrochemical oxidation of thioethers.

Electrochemical reactions have various disadvantages which prevent them from being widely performed on scale. Organic decomposition on the electrode, and hot-spot formation reduce the performance over the time and limit process scalability. The usage of flow technology could overcome such issues. In 2017, Noël et al. assembled a microflow electrocell for the electrochemical oxidation of thioether compounds. This apparatus enable the avoidance of stoichiometric oxidising agents, and reduced reaction times (Scheme 96) [369]. Fine tuning the voltage and reaction times of the flow system, allowed 17 different substrates to be oxidised to their corresponding sulfoxides or sulfones in moderate to good yields (31–92%).

**Scheme 97:**
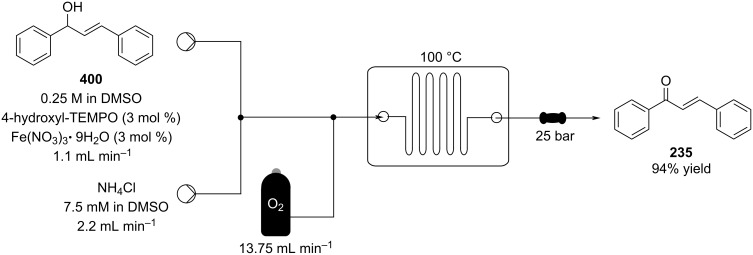
Continuous-flow oxidation of **400** to cinnamophenone (**235**).

Recent industrial interest in establishing continuous oxidation processes is demonstrated by the rapidly increasing patent literature, for example, for the synthesis of hexanoic acid from 2-octanol [370] and the microreactor-based continuous aerobic oxidation to chalcone (**235**, Scheme 97) [371].

**Scheme 98:**
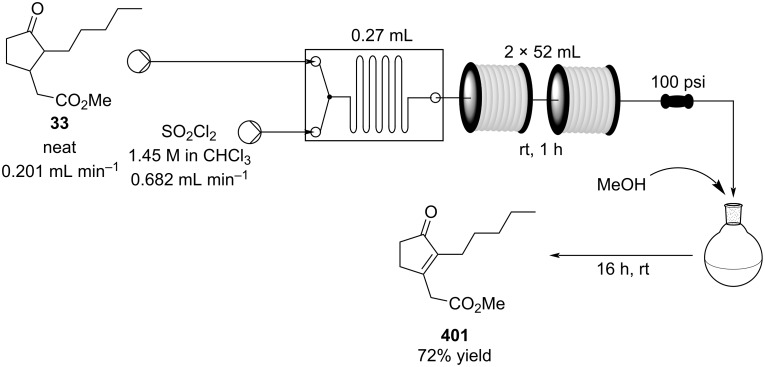
Continuous-flow synthesis of dehydrated material **401** via oxidation of methyl dihydrojasmonate (**33**).

In 2018, IFF, patented a continuous-flow process for the oxidation of methyl dihydrojasmonate (**33**) through a two-step chlorination–elimination reaction. The system enabled the efficiently synthesis the desired dehydrated material **401** in 72% yield safely using sulfuryl chloride as the chlorinating agent (Scheme 98) [372]. The chlorination step was carried out in an Uniqsis FlowSyn reactor and, after collection and MeOH addition, the elimination step occurred spontaneously in batch over a period of 16 hours.

#### The use of Grignard reagents

**Scheme 99:**
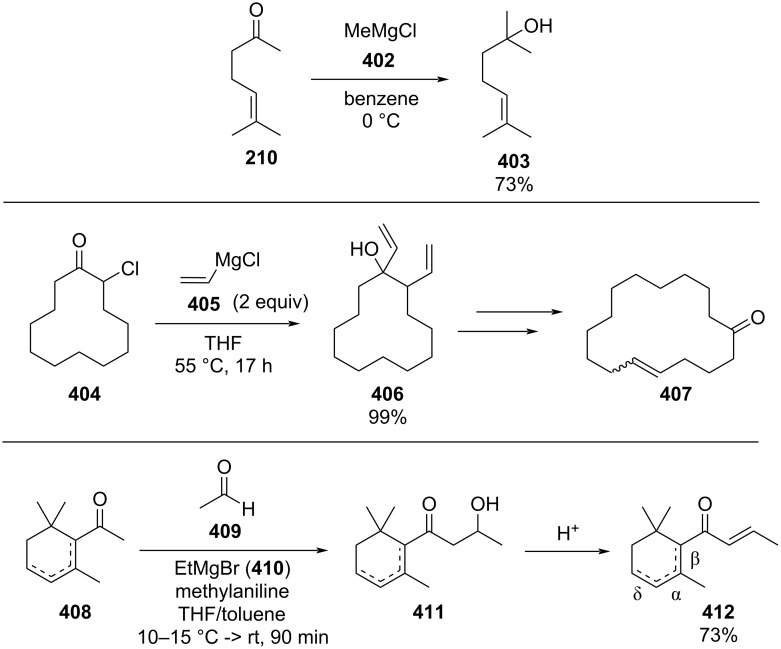
Some industrially important transformations involving Grignard reagents.

The Grignard reaction is a particularly important C–C bond forming sequence for the synthesis of higher order alcohol products and intermediates. Pertinent examples relating to the fragrance and flavours industry include the methylation of 6-methyl-5-hepten-2-one (**210**) [373] and for the introduction of vinyl groups to 2-chloro-5-cyclohexadecen-1-one (**404**), in a synthesis of velvione (**407**) (Scheme 99) [374]**.** Grignard reagents are also commonly employed as strong bases, for example, in the aldol reactions of cyclohexenyl methyl ketones, key intermediates in the synthesis of α-, β- and δ-damascones (**412**) [375–376]. These structures represent a very reactive class of compounds that require strict control over the conditions including an inert environment and are renowned for possessing highly exothermic reaction profiles. The application of flow to the use and preparation of Grignard reagents has been explored fairly extensively in the past few years.

Industrially, Grignard reagents are mainly prepared in stirred tank reactors via halogen/magnesium insertion reaction. The process can be highly exothermic and can therefore be problematic as the ideal preparative solvents are normally ethers which have typically low boiling points and are highly flammable. Moreover, heat removal during activation can be difficult and inhomogeneous hot spots readily occur inducing side reactions (i.e., Wurtz coupling), reducing the efficacy of the preparation and increasing associated risk at scale.

**Scheme 100:**
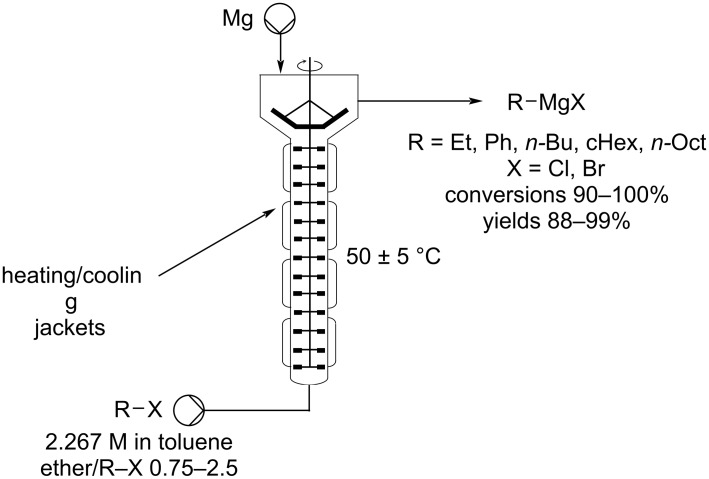
Grachev et al. apparatus for continuous preparation of Grignard reagents.

In 2012, a continuous preparation of Grignard reagents was performed using a heated column with a multi-bladed stirrer filled with granulated magnesium (Scheme 100) [377]. The halide was fed from the bottom as a mixture in ether (diethyl ether or THF) and toluene. The organomagnesium halide compound stream was collected from the top of the reactor. The magnesium was first activated by introducing a mixture of ether and dibromoethane in toluene and then a 2.267 M solution of alkyl halide was constantly introduced. The preparation of different halides was optimised using ether/R–X ratios from 0.75 and 2.5 gaining excellent conversions (90–100%) and excellent yields (88–99%).

**Scheme 101:**
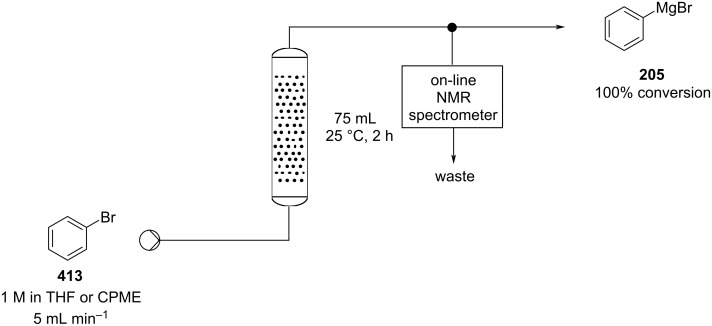
Example of fluidized Mg bed reactor with NMR spectrometer as on-line monitoring system.

A few years later, the group of Duchateau also reported a continuous preparation of phenylmagnesium bromide using a magnesium fluid bed reactor (Scheme 101) [378].

Granular magnesium was loaded into a 75 mL stainless steel tube reactor and activated by flushing with a premade solution of the Grignard reagents in THF. A 1 M solution of bromobenzene in THF or cyclopentyl methyl ether (CPME) was pumped through the tube reactor at a settling velocity to allow the solid to fluidize into the flow stream. Instead of titrating, the air-sensitive magnesium halide was monitored using an on-line 42 MHz NMR spectrometer. A screening of different reaction conditions enabled optimisation to ensure complete conversion of the halide input at 25 °C with a 2 hours residence time.

Alcázar et al. also used this approach to generate a stream of Grignard reagents from a column reactor (magnesium particle size 20–230 mesh) [379]. The solid packed reactor was initially flushed with a solution of DIBAL-H in THF/toluene (1:1) and then activated by flowing TMSCl and 1-chloro-2-bromoethane in toluene through the system. The Grignard reaction (section of aryl and alkyl halides) was then performed in the presence of LiCl (0.5 M) in THF to avoid clogging of the column due to solid formation. After 7.5 minutes a solution 0.38 M of the organomagnesium halide was collected (estimated metallic composition 76%). On larger scales this performance did not change until 60–70% of the amount of magnesium had been consumed. Several examples of Grignard reagents were synthesised through this method and these were then directly mixed in-line with electrophiles gaining different addition products in good to excellent yields (Table 8). The freshly-made Grignards were also collected and employed in other reactions where the batch protocol is more suitable such as conjugate addition, sulfone formation and Negishi couplings. More recently, Noël et al. described a similar telescoped system for the in situ preparation of organomagnesium bromides to be employed in the iron-catalysed cross-coupling with alkenyl and styrenyl chlorides [380].

**Table 8:** Continuous synthesis of Grignard reagents and subsequent quenching with electrophiles.

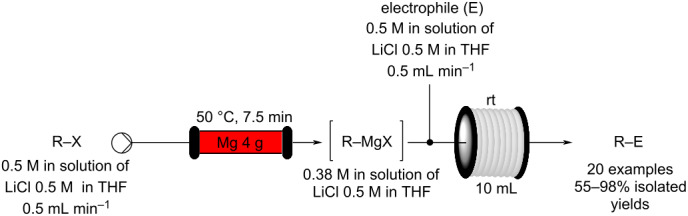				

Entry	Electrophile	R–X	Product	Yield (%)

1	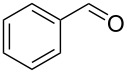 **17**	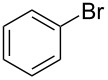 **413**	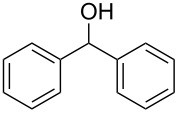 **414**	94
2	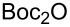 **415**	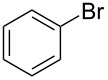 **413**	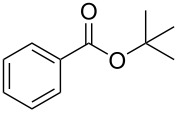 **416**	97
3	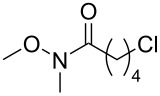 **417**	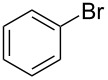 **413**	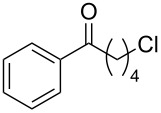 **418**	58
4	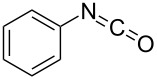 **419**	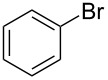 **413**	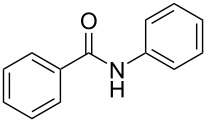 **420**	84
5	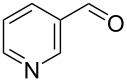 **421**	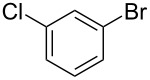 **422**	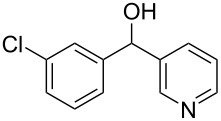 **423**	74
6	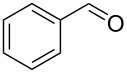 **17**	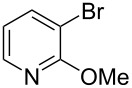 **424**	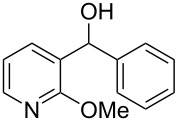 **425**	75
7	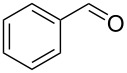 **17**	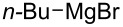 **426**	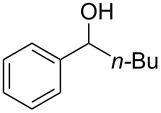 **427**	98
8	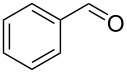 **17**	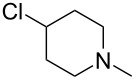 **428**	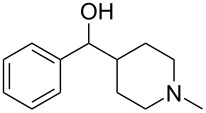 **429**	81

**Scheme 102:**
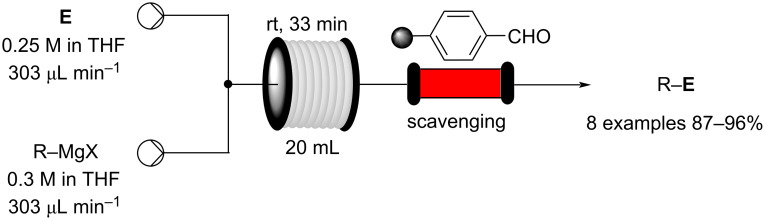
Continuous-flow synthesis of Grignard reagents and subsequent quenching reaction.

In 2010, a collection of secondary and tertiary alcohols, including the analgesic tramadol (*anti-***443**), were prepared from their corresponding ketones in flow (Scheme 102) [381].

After merging the starting material streams at room temperature and passage through a series of reactor coils the mixture was directed through a packed column containing a polymer-supported benzaldehyde resin which was used to scavenge any excess Grignard reagent. This yielded after a batch quenching step and work-up the alcohol products in high yields (Table 9).

**Table 9:** Continuous-flow reactions of aromatic aldehydes/ketones with Grignard reagents and substrates synthesised.

Entry	Electrophile	R–MgX	Product	Yield (%)

1	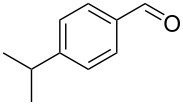 **27**	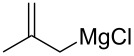 **430**	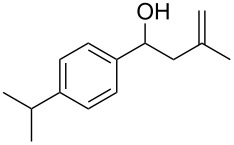 **431**	92
2	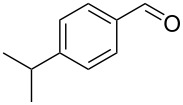 **27**	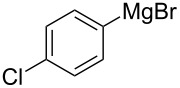 **432**	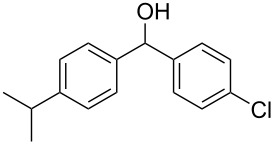 **433**	93
3	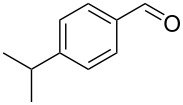 **27**	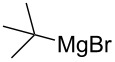 **434**	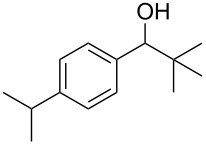 **435**	87
4	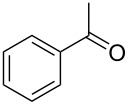 **289**	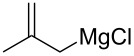 **430**	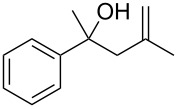 **436**	94
5	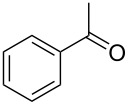 **289**	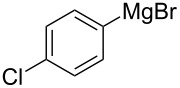 **432**	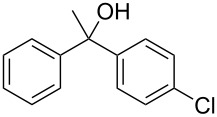 **437**	95
6	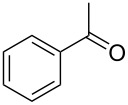 **289**	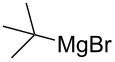 **434**	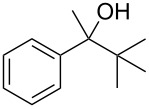 **438**	90
7	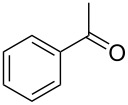 **289**	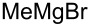 **439**	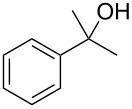 **440**	95
8	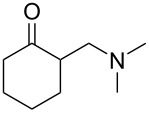 **441**	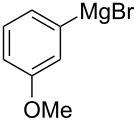 **442**	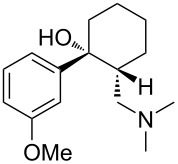 *anti*-**443**	96 dr (*syn*/*anti*) 2:8

Significant effort has been directed towards in-line work-ups for flow protocols involving Grignard reagents as a safer way of quenching the reaction and reducing the decomposition of the intermediate metallated species. A continuous-flow approach to 3,3,3-trifluoromethylpropenes was reported in 2014 [382]. A range of derivatives were prepared via an initial Grignard reaction followed by a Peterson elimination in a fully continuous process that made use of both an in-line extraction and solvent switching (Table 10).

**Table 10:** Synthesis of 3,3,3-trifluoromethylpropenes by continuous Grignard reaction.

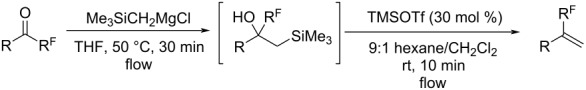				

Entry	Ketone	Product	Yield in flow (%)	Yield in batch (%)

1	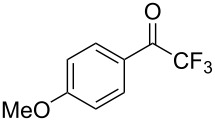 **444**	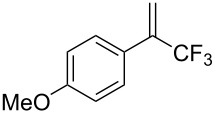 **445**	90	68
2	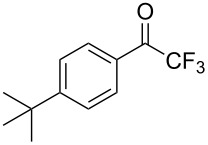 **446**	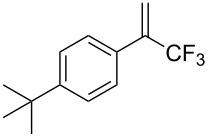 **447**	90	65
3	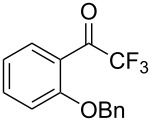 **448**	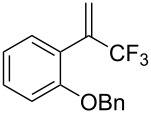 **449**	89	67
4	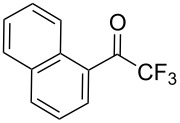 **450**	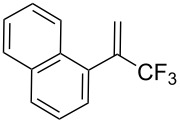 **451**	92	72
5	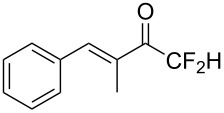 **452**	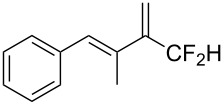 **453**	92	75
6	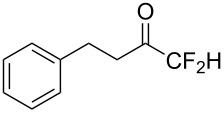 **454**	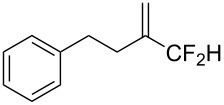 **455**	94	85

**Figure 10:**
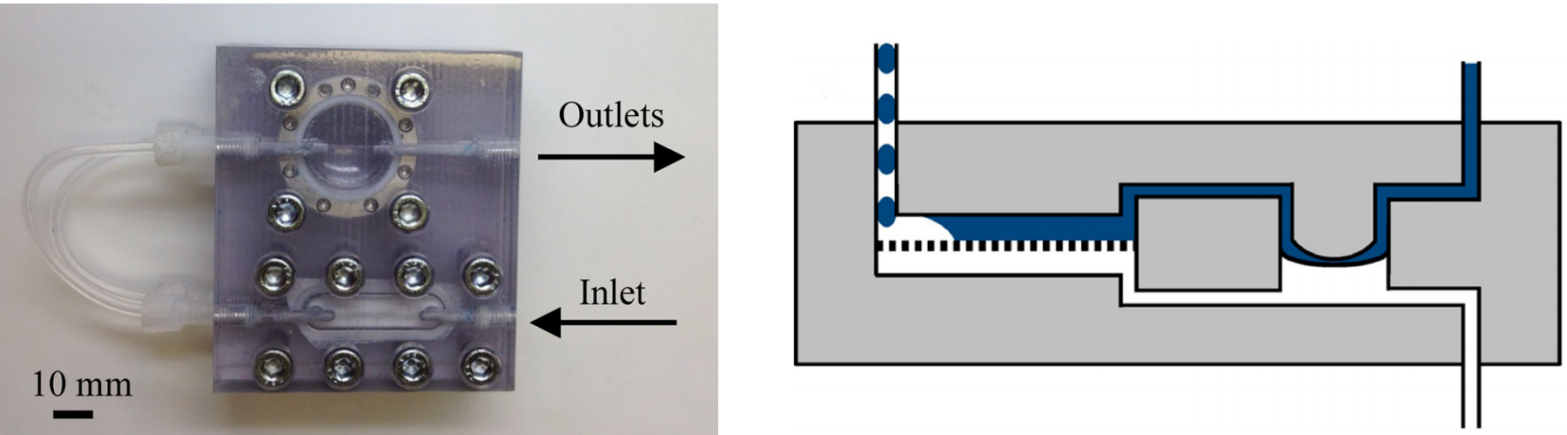
Membrane-based, liquid–liquid separator with integrated pressure control [52]. Adapted with permission from [52]. Copyright 2013 American Chemical Society.

Reaction times were shorter and the yields obtained were markedly better than analogous batch procedures reported by the same group later in the same year [383]. The crude product from the Grignard reaction was hydrolysed in-line using downstream inlets of aqueous acid followed by a 9:1 hexane/chloroform mixture which aided extraction via a liquid–liquid membrane-based continuous separator (Figure 10) [52].

Many other multistep sequences that make use of Grignard reactions in flow can be found in the literature; excellent examples are found in the preparation of amitriptyline [384], (*E*/*Z*)-tamoxifen [212], and 2-aminoadamantane-2-carboxylic acid [385–386].

**Scheme 103:**
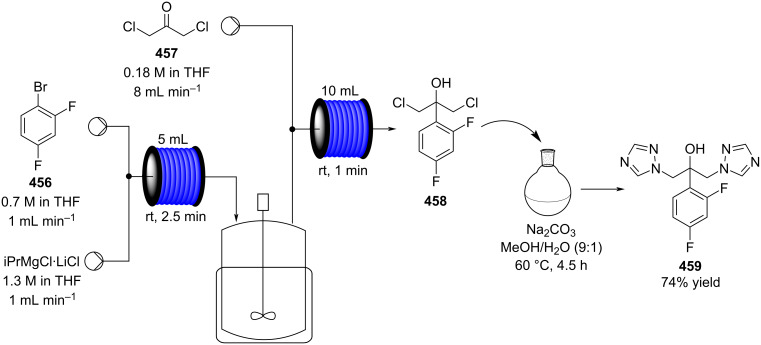
Continuous-flow synthesis of **458**, an intermediate to fluconazole (**459**).

In 2017, Gupton et al. developed a continuous-flow preparation for the intermediate of fluconazole (**459**), a potent antifungal, anti-HIV, and anticancer agent (Scheme 103) [387].

The flow apparatus consists of two steps: Grignard preparation and carbonyl addition. Initially, the two steps were optimised singularly and then telescoped. An excess of iPrMgBr∙LiCl (1.8 equiv) was used to form the desired organomagnesium reagent from the 1-bromo-2,4-difluorobenzene (**456**). An excess of Grignard reagent was also employed in the second step to increase the conversion. After the flow systems, the authors realised the presence of a continuous stirring tanks reactor (CSTR) before the quenching with the ketone intermediate **457** (longer residence time) increased the yield of **458** (74% vs 87%). Due to the long reaction times of the last step, the synthesis of fluconazole (**459**) was finished using a batch step. For the specific Grignard addition into the carbonyl, the volume–time output (VTO) was calculated to be 8.962 × 10^−7^ m^3^ h kg^−1^, which is in accordance with the aims of process chemistry (VTO < 1).

**Scheme 104:**
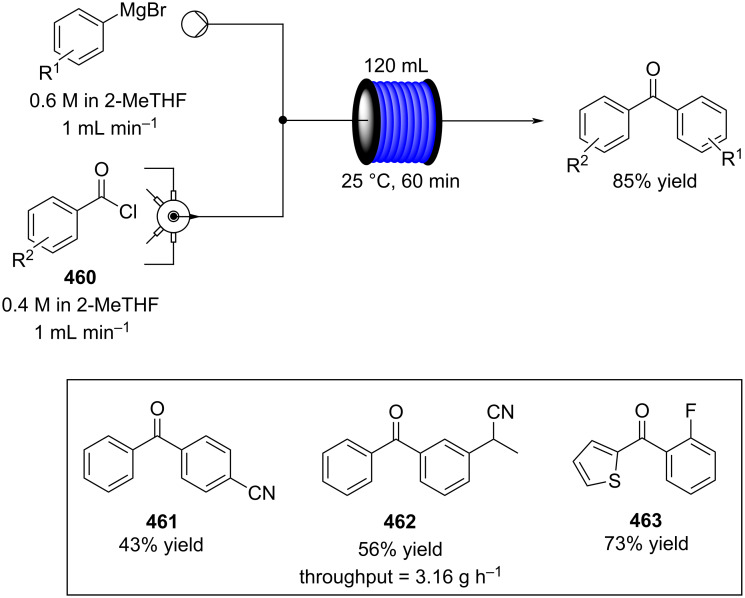
Continuous-flow synthesis of ketones starting from benzoyl chlorides.

Utilising Grignard reagents as strong bases to effect transformations in flow has also been shown with the Bodroux amide formation in 2012 [388] and continuous-flow Grignard addition to nitriles, acting as carbonyl surrogates, being reported in the following year [389]. Recently, amide formation was achieved using isocyanates as electrophiles in a copper-catalysed flow reaction. The flow system developed by Kerr, Leach and their co-workers employed stoichiometric amounts of reagents yielding amides in moderate to excellent yields [390]. In 2019, Wang, Castle and co-workers optimised the addition of Grignard reagents to benzoyl chlorides (Scheme 104) [391].

The reaction is extremely exothermic due to the combination of two strong electrophile/nucleophile being mixed. Also in this example, flow chemistry facilitates optimal mixing and heat transfer allowing a high selectively and good yield of the ketones (43–85%). Interestingly, automation was incorporated into the reactor allowing several benzoyl chlorides to be screened sequentially using a 5-ways valve placed before the T-mixer. The system proved to be more efficient than the batch method (85% vs 34%) and it could be employed for on-demand preparation of substrates enabling high throughputs (e.g., 3.16 g h^−1^ for the ketoprofen intermediate **462**).

**Scheme 105:**
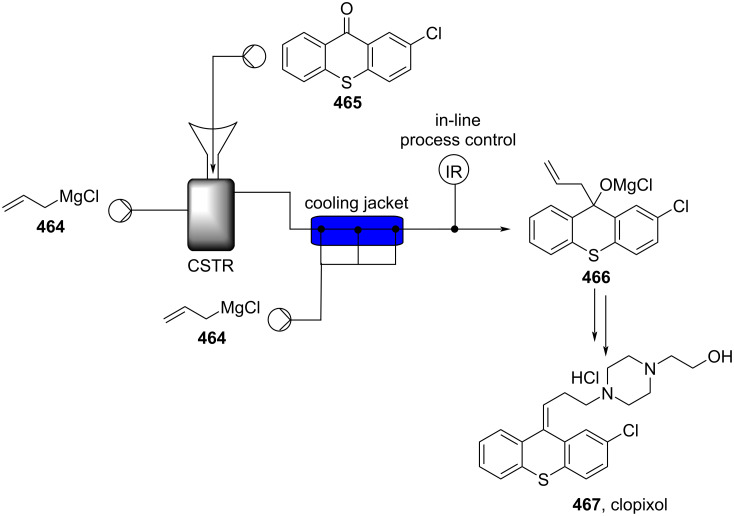
A Grignard alkylation combining CSTR and PFR technologies with in-line infrared reaction monitoring.

When moving to larger scales involving highly exothermic transformation such as Grignard additions, it is often problematic to conduct such processes in batch as safety concerns are greatly increased. The feasibility to scale up Grignard reactions in flow was demonstrated in a full-scale heterogeneous Grignard alkylation of a pharmaceutical intermediate **466** by Kiil et al. in 2013 (Scheme 105*,* Table 11) [392]. Due to solubility issues of **465**, the design was based around a continuous stirred tank reactor (CSTR) which fed the solid **465** via a screw feeder and the Grignard solution, allylmagnesium chloride (**464**), via a pump. The reactor outlet was encased in a filter cartridge to avoid further transfer of insoluble material into the secondary plug flow reactor (PFR).

**Table 11:** Batch vs flow process comparison for the Grignard alkylation of **465**.

Parameter	Full-scale (semi-batch)	Laboratory (continuous)	Full-scale (continuous)

active volume	1600 L	250 mL	8 L
total size	≈10 m^2^	2 m^2^	1.45 m^2^
production time (h)	4	900	<50
yield (%)	>95	>96	95–99
purity (%)	>95	>99	96.5–99.8
solvent consumption (L/kg of **465**)	5.8	2.3	3.8
cleaning	every batch	dedicated	dedicated

Hydrolysis of the Grignard adduct **466** followed by further steps eventually produced clopixol (**467**), an antipsychotic drug. Switching from a batch to flow regime afforded numerous advantages; a lower active volume, smaller setup size, improved yield and increased purity of product, a reduction in solvent consumption and favourable cleaning requirements. The incorporation of on-line infrared monitoring allowed for facile process control (process analytical technology, PAT), this is something that is well precedented and highly valuable for Grignard reactions [393–395].

**Scheme 106:**
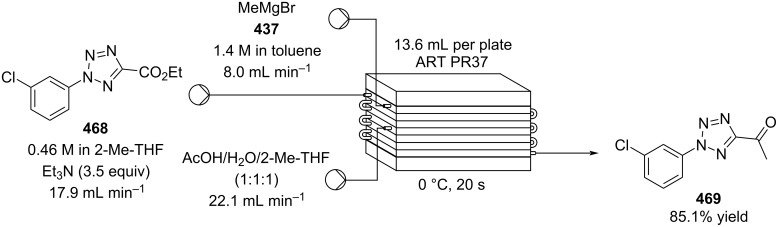
Continuous-flow preparation of **469** from Grignard addition of methylmagnesium bromide.

In 2014, a mesoreactor scale-up was reported for the manufacture of ketones from the corresponding esters using a Grignard reagent [396]. Initial studies involved performing the reaction in a microchip reactor (internal volume < 1 mL), an ART PR37 mesoreactor (internal volume = 13.6 mL) was then used, resulting in a scalable procedure that could be viewed as an alternative to the Weinreb ketone synthesis (Scheme 106).

**Scheme 107:**
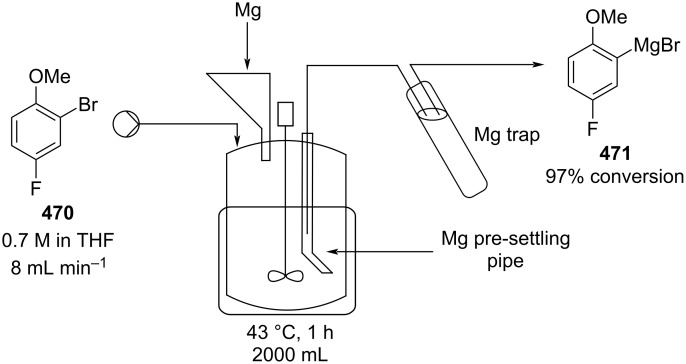
Continuous-flow synthesis of Grignard reagents **471**.

Continuous-flow processes can also be developed by modifying batch vessels. This approach presents several advantages, for instance it better tolerates solids, allows the use of higher concentration solutions, and it can make use of pre-existing plant equipment. At Eli Lilly batch vessels were explored for Grignard preparation, as magnesium solids may result in clogging of PFR’s [397]. The CSTR system was continuously fed with aryl halide and the solution has a residence time in the vessel of roughly an hour. The so formed Grignard reagent was then removed and, to control the solid presence in the solution, passed through a pre-settling pipe and a Mg trap. The apparatus operated over 14 hours (2 working days) yielding the desired reagent **471** in 97% conversion, reducing the amount of Mg by 50% compared with the batch process (Scheme 107).

**Scheme 108:**
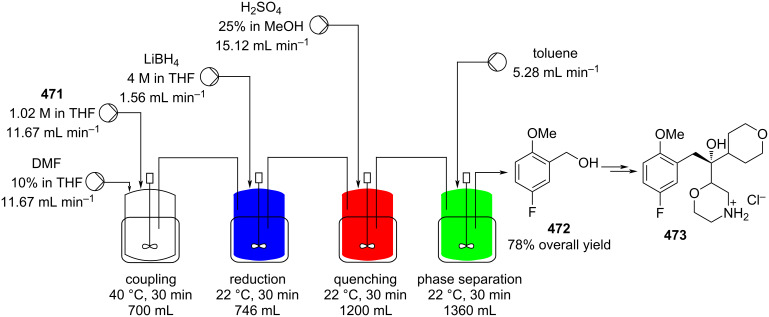
Preparation of the Grignard reagent **471** using CSTR and the continuous process for synthesis of the edivoxetine's intermediate **472**.

The newly formed organomagnesium halide was employed for the development of the continuous synthesis of **472**, an intermediate of edivoxetine hydrochloride (**473**), an antidepressant agent (Scheme 108).

Interestingly, the authors conducted a comparison of the synthesis of compound **472** using the described CSTR Grignard process and employing a PFR-optimised lithiation. The Grignard process was selected as the most suitable method of preparation for their needs, as such a 2 L scale-up was carried out and after 32 hours (5 working days) roughly 4 kg of **472** was obtained in a yield of 82%. Crystallisation of the crude product afforded the desired intermediate in >99.8% purity with an overall yield of 78%; an improvement in the yield of over 10%.

**Scheme 109:**
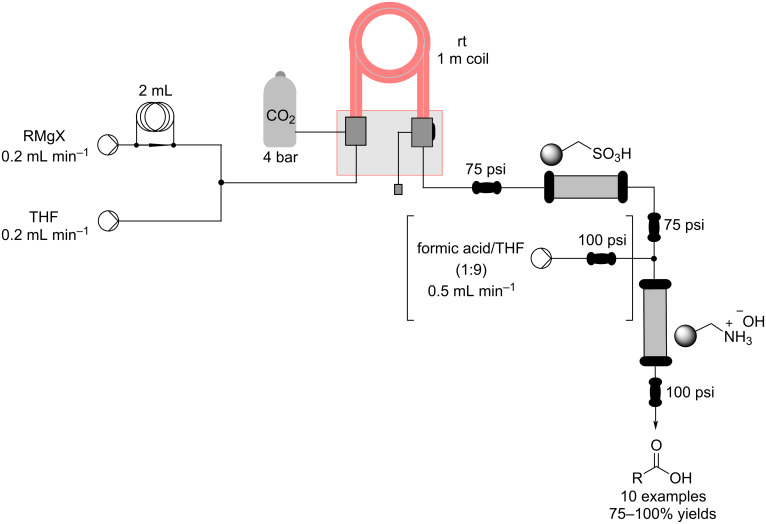
Continuous process for carboxylation of Grignard reagents in flow using tube-in-tube technology.

Combining Grignard reactions with various gases in flow has also been achieved furnishing ketones [398], carboxylic acids [258] and phenols [399–400]. As early as 2011, the Ley group reported the use of a tube-in-tube reactor to effect the conversion of a collection of aromatic Grignard reagents to their corresponding carboxylic acids in good to excellent yields (Scheme 109) [258].

A polymer-supported sulfonic acid column allowed in-line scavenging of the magnesium salts upon exiting the main reactor while simultaneously protonating the carboxylate product. A “catch-and-release” strategy was then adopted to further purify the product by using a polymer-supported ammonium hydroxide column.

**Scheme 110:**
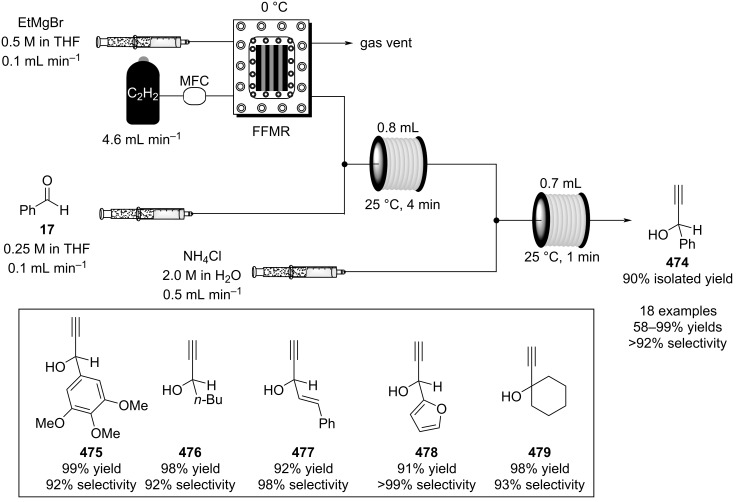
Continuous synthesis of propargylic alcohols via ethynyl-Grignard reagent.

In 2014, the use of acetylene gas for the generation of an ethynyl-Grignard reagent and subsequent synthesis of the propargylic alcohols **474–479** was reported in flow (Scheme 110) [401]. The initial formation of the Grignard reagent from ethylmagnesium bromide and acetylene gas was conducted in a falling film microreactor (FFMR) with the output being telescoped into a second reactor along with a range of ketones. After acidification using ammonium chloride, the corresponding propargylic alcohols could be isolated. The methodology was applied to eighteen different substrates achieving mostly high yields (one 58% and all others >88%) and high selectivity (all >92%). In a similar way a Chinese patent application has been made for the process to be performed within a microstructured reactor [402].

**Scheme 111:**
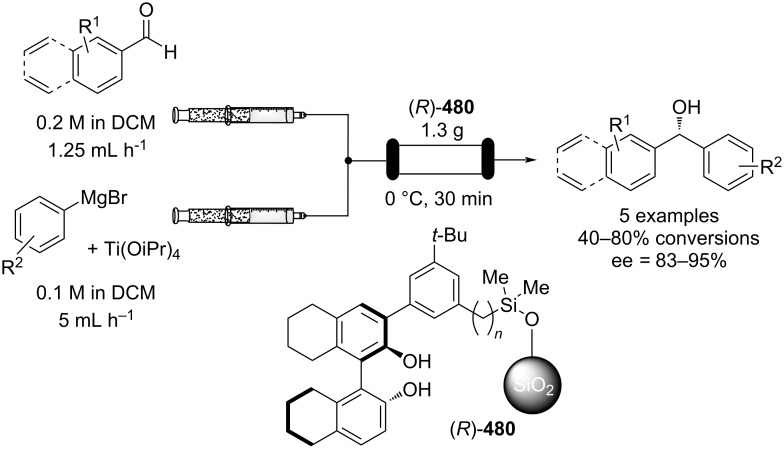
Silica-supported catalysed enantioselective arylation of aldehydes using Grignard reagents in flow conditions.

Recently, Grignard reagents have been exploited in enantioselective arylation of aldehydes using a silica-supported catalyst (H_8_-BINOL-derivative) [403]. The grafted catalyst was placed in a disposable pipette (6 × 100 mm) connected to syringe pumps. At first, a solution of the Grignard and titanium isopropoxide in DCM (0.1 M) was eluted to form the activated Ti catalyst. Once formed, a solution of the aldehyde in DCM (0.2 M) was introduced at a set flow rate that maintained the molar ratio between the reagents at 2 equivalents. After 30 minutes residence time, the solution was quenched. Several substrates were screened furnishing moderate to good conversions (40–80%) and good to excellent enantioselectivites (83–95%). Of particular importance is that the column could be repeatedly used for several experiments revealing the catalyst to be stable under the flow processing conditions (Scheme 111).

#### Isomerisations and rearrangements

**Scheme 112:**
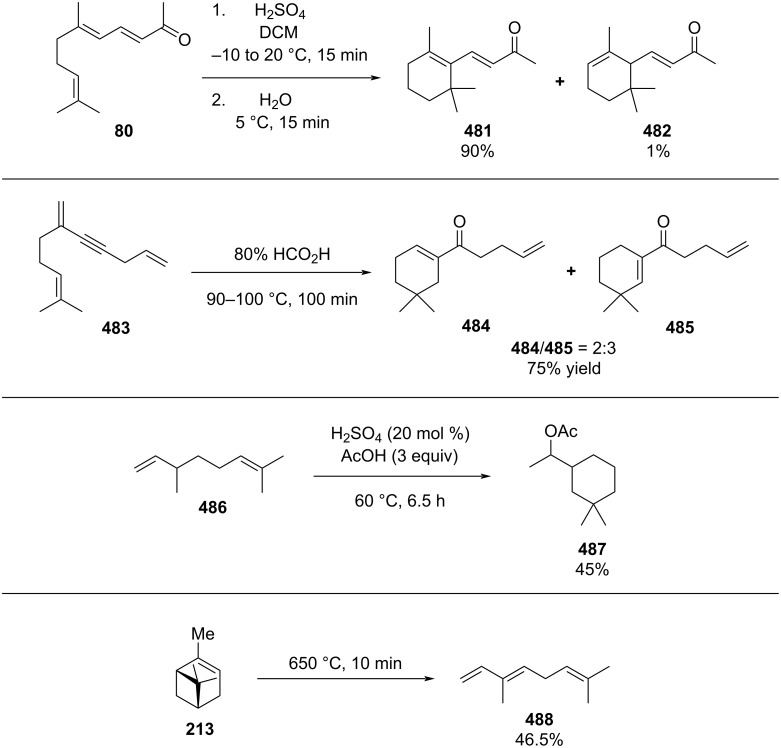
Acid-catalysed rearrangement of citral and dehydrolinalool derivatives.

Terpene-like products are key materials in medicinal and F&F chemistry, where several total syntheses have been developed [376]. Isomerisation and rearrangement reactions are commonly employed during several stages of their production (Scheme 112) [404]. For instance, the synthesis of β-ionone (**481**) is made possible via an acid-catalysed rearrangement of Ψ-ionone (**80**) [405]. Similarly, galbascone (**484**) may be prepared by an acid-catalysed rearrangement of the dehydration product of dehydrolinalool (**454**) [406]. Acid-catalysed rearrangement have also been employed for the synthesis of musk acetate (**487**) [407]. Pyrolysis of α-pinene to *o-*cimene (**488**) has also found great interest in preparation of F&F intermediates [408].

**Scheme 113:**
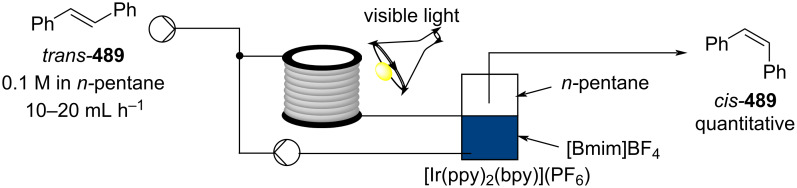
Continuous stilbene isomerisation with continuous recycling of photoredox catalyst.

The selectivity and efficiency of many isomerisation and rearrangement reactions are highly dependent on the reaction temperature or irradiation wavelength and are therefore well suited to flow processing (improved contact surface areas resulting in better mass and heat transfer). This has been exemplified for the photo-oxidation of a range of fragrance-type substrates [312–313,315,409]. More recently, a continuous-flow system for catalytic alkene isomerization using visible light was reported by Rueping et al., for which the conversion of *trans*- (**489**) to *cis*-stilbene (**489**) was studied (Scheme 113) [410].

A batch process was initially developed involving the use of an [Ir(ppy)_2_(bpy)](PF_6_) photoredox catalyst with the ionic liquid, [Bmim]BF_4_, which was eventually translated into a flow system with recycling of the easily spreadable photoredox catalyst system. Despite the relatively low flow rates investigated (<20 mL h^−1^), quantitative conversions were achieved without loss of the valuable iridium catalyst. In a related set of examples an iridium catalysed alkene isomerisation has also been shown to be possible in flow by using an immobilised version of Felkin’s catalyst [126,411]. *Trans*-to-*cis* alkene photoisomerisation was also performed in flow on a ^18^F-labelled *cis-*cyclooctene employed as dienophile for Diels–Alder (DA) reaction [412].

Claisen rearrangements in flow have been studied extensively [413–419] and in 2011 a report by scientists at Eli Lilly & Co. looked at comparing the thermal *ortho*-Claisen rearrangement of the allyl aryl ether, **490**, an important early phase intermediate, in flow to batch (Table 12) [417]. Significant advantages were associated with the flow protocol, stemming from the ability to use higher concentrations (even solvent-free conditions), pressures and temperatures in a safe manner [419]. As a result, a much safer and highly reproducible system was achieved which was not easier to perform at higher temperatures, but also was associated with less problematic workup. Interestingly, in this area continuous-flow microwave reactors have also been developed for high temperature and pressure Claisen rearrangements [413,418,420].

**Table 12:** Batch vs flow for the thermal Claisen rearrangement of an ally aryl ether **490**.

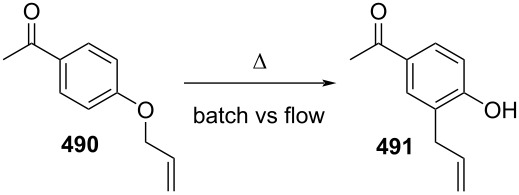		

	Batch process	Flow process

solvent	diphenyl ether	*N*-methylpyrrolidinone
concentration	33 wt %	50 wt %
temperature	220 °C	230 °C
solvent bp	259 °C	202–204 °C
operating pressure	1 bar	15 bar
reaction time	5 h	4 h
workup	crystallisation on cooling (potential to freeze solvent below 27 °C)	aqueous drown out and extraction
safety	potential for runaway reaction	improved heat transfer provides better temperature control; contained system; potential to safely run at higher concentrations
manufacturability	220 °C not easily reached by typical batch reactor heat transfer systems	230 °C easily reached in ovens
robustness	reaction temperature variations leading to variation in yields and purity	highly reproducible

**Scheme 114:**
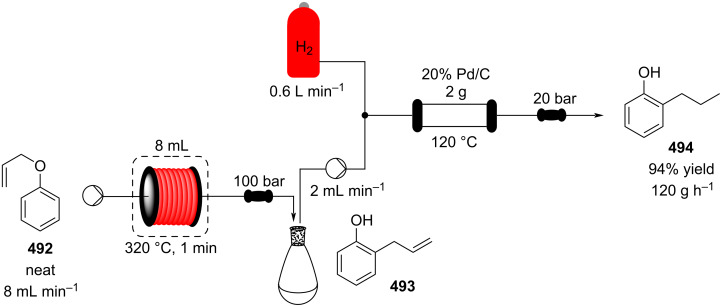
Continuous-flow synthesis of compound **494** as developed by Ley et al.

In 2015, a continuous-flow method for the preparation of *o-*cimene (**488**) from thermoisomeric α-pinene (**213**) in the liquid phase was patented by Jiangxi Jiayuan Fragrance Co Ltd. [421]. One year later, Ley and co-workers developed a continuous-flow 2-step telescoped solvent-free synthesis of 2-propylphenol (**494**), a smoky, and phenolic fragrance compound, starting from allyl phenyl ether (**492**, Scheme 114) [422].

The flow approach employed a Phoenix reactor (ThalesNano^®^) as a high temperature operating device. In this example, a Claisen rearrangement was performed in 1 minute at 320 °C and 100 bar. The reaction was also easily scale-up from 60 g h^−1^ to 480 g h^−1^ by only changing the reactor heated volume (from 1 to 8 mL). As different flow rates were required to perform the two reaction steps, the intermediate **493** was collected in a reservoir, ready to be streamed in the secondary hydrogenator. The final step involves a 3 mL column reactor packed with 20% Pd/C heated at 120 °C. The optimised setup enabled the preparation of the desired material **494** in 94% yield with a promising throughput of 120 g h^−1^.

Isomerization and rearrangements in flow chemistry are relatively underexplored and is an area of active research currently.

#### Cycloaddition reactions

**Scheme 115:**
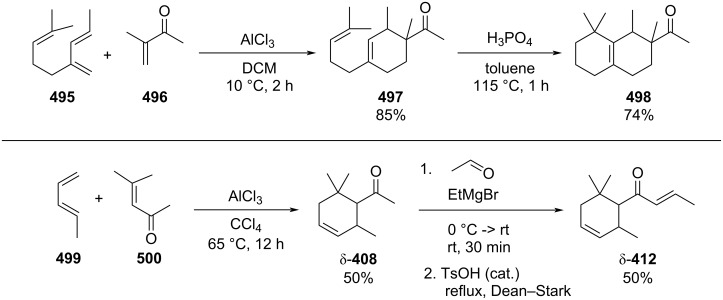
Selected industrial applications of DA reaction.

Cycloaddition reactions represent a powerful tool for the construction of rings, especially fused systems. It can also provide precursors to linear molecules through ring opening via ozonolysis and metathasis processes. Diels–Alder (DA) reactions have been extensively exploited by the F&F industry and a rich body of work has been published in the literature [423]. DA synthesis of an Iso-E-Super intermediate **498** has been performed using aluminium trichloride as a Lewis acidic catalyst [424]. A δ-damascone (δ-**412**) precursor is also prepared in this way as a further important example (Scheme 115) [425].

Although widely explored, the number of industrial applications is still low compared to other chemical transformations, mainly due to safety concerns originating from the inherent reactivity of dienes and dienophiles with regard to competing runaway reactions like polymerisation. Hence, flow chemistry and its advantage in respect to exacting control over reaction conditions has proved useful, reducing the likelihood of exothermic side reactions.

**Scheme 116:**
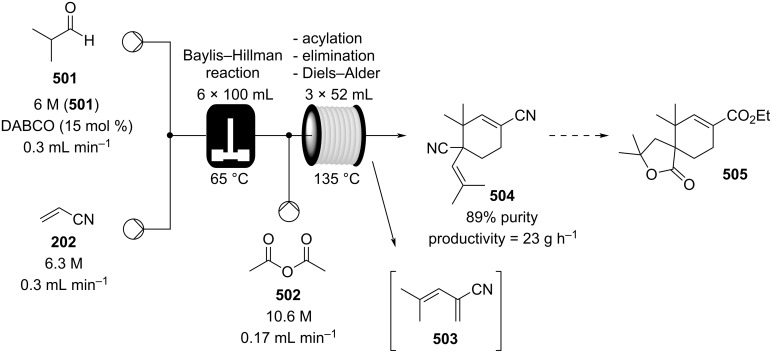
Multistep flow synthesis of the spirocyclic structure **505** via employing DA cycloaddition.

In 2015, a multistep flow synthesis of a spirocyclic compound **505**, which is a potential fragrance compound, was achieved [426]. It’s precursor **504** can be synthesised via a DA dimerization of the intermediate diene **503** which was obtained via a Baylis–Hillman transformation and activated elimination of the hydroxy group. This process provided a robust way to generate product **504** in 89% purity, obtaining after 6.5 days 3.58 kg of product with a throughput of 23 g h^−1^ (Scheme 116).

**Scheme 117:**
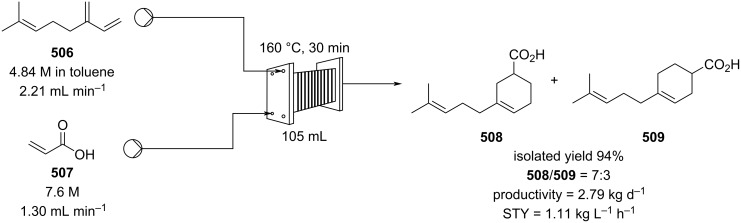
Continuous-flow DA reaction developed in a plater flow reactor for the preparation of the adduct **508** from myrcene (**506**).

Recently, continuous-flow DA cycloadditions have been performed on myrcene (**506**), which is an acyclic monoterpene widely used as a building block in the F&F industry [427]. The authors wanted to prepare a new potential surfactant using myrcene (**506**) and acrylic acid (**507**). The DA adduct **508** obtained was scaled up from the batch to flow systems. The flow apparatus enables a reduction in reaction time (10 h vs 30 min) through an increase in temperature in the flow system (110 °C vs 160 °C), demonstrating a notable process intensification. Two different flow reactors of different sizes were employed in the scale-up: a 10 mL plug reactor (Vapourtec R-series), and a 100 mL plate reactor (Chemtrix Plantrix^®^ MR260) (Scheme 117). In both case similar results were obtained (99% vs 100%), with an increase of 10-times the productivity to 2.79 kg of **508** per day (STY = 1.11 kg L^−1^ h^−1^).

DA reactions mostly involve the use of an α,β-unsaturated ketone or aldehyde as the dienophile component. Lowering the energy of the LUMO of the dienophile via the incorporation of a Lewis acid is a commonly employed strategy in catalysing such reactions. While the use of salts such as aluminium(III) chloride may pose solubility issues in flow, numerous methods for the continuous processing of reaction slurries have been reported [44,428–431]. Alternative methods of catalysis have also been developed over the past few decades which offer feasible transposition to flow. Zeolite [432–433], heteropoly acids (HPA) [434] and other Lewis acid catalysis [435] have been exploited under flow conditions.

**Scheme 118:**
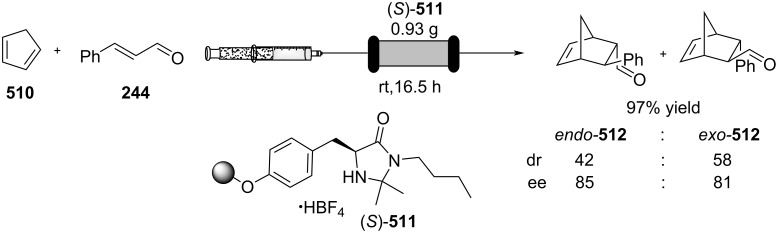
Continuous-flow DA reaction using a silica-supported imidazolidinone organocatalyst.

In addition, enantioselective organocatalytic DA reactions were first reported in 2000 by MacMillan et al. [436], following further discoveries [437], such catalysts have been incorporated into flow. Silica-supported imidazolidinones were used by Benaglia et al. in 2013 for stereoselective continuous-flow DA reactions of cyclopentadiene (**510**, Scheme 118) [438–439]. More recently, a monolith reactor containing polymer-based imidazolidinones has been developed by the same group [440]. An TADDOL-based organometallic version has also been attempted although this was less effective delivering poor ee (6.5–25%) [441].

**Scheme 119:**
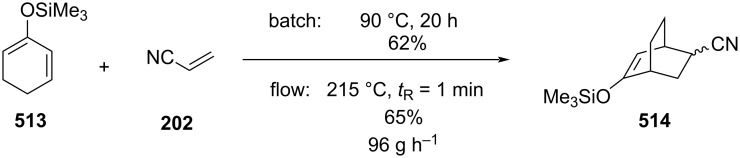
Batch vs flow for the DA reaction of (cyclohexa-1,5-dien-1-yloxy)trimethylsilane (**513**) with acrylonitrile.

The advantages of conducting DA reactions in flow have been more widely demonstrated. In 2012 Abele et al. reported the DA reaction of (cyclohexa-1,5-dien-1-yloxy)trimethylsilane (**513**) with dienophiles α-acetoxyacrylonitrile and acrylonitrile in flow (Scheme 119) [442]. Batch to flow comparisons revealed that overcoming thermokinetic issues associated with batch scale-ups was possible by adopting a flow approach. For the process involving acrylonitrile, access to a much larger temperature window (<215 °C compared to <90 °C) in flow allowed for accelerated reaction. A residence time of only 1 minute (cf. reaction time of 20 hours in batch at 90 °C) using a simple stainless steel tube reactor design gave a productivity of 96 g h^−1^. Other DA reactions have similarly shown reductions in reaction time [443–446].

**Scheme 120:**
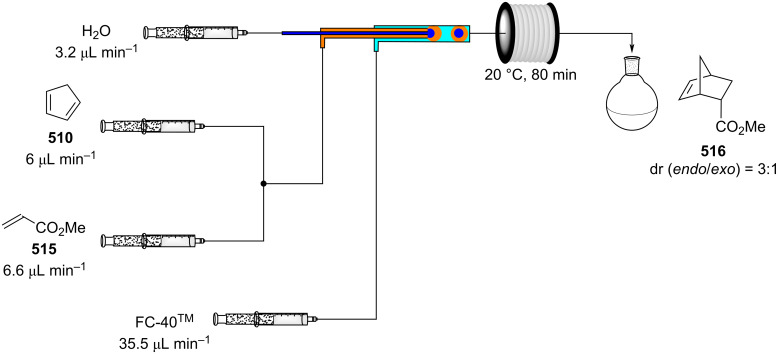
Continuous-flow DA reaction between **510** and **515** using a shell-core droplet system.

As DA reactions are characterised by a negative volume change, biphasic systems notably accellerate the reaction due to solvophobic effects. Karl and Löwe developed a flow system where this effect is exploited to enhance the reaction rates (Scheme 120) [447].

The apparatus consists of a segmented flow stream where core–shell droplets made of water (core) and the reaction mixture (shell) were directed into a horizontally placed coil. The droplets were formed utilising a engineered processing unit [448] and streamed by means of a fluorinated solvent (FC-40™). The DA reaction between cyclopentadiene (**510**) and methyl acrylate (**515**) was optmised. The authors pointed out the importance of the reactor positioning as gravity-induced mixing significantly improved the outcomes. The system was found to be more efficent than a slug flow one, allowing to perform the experiment at room temperature without the need for temperature and pressure control.

**Scheme 121:**
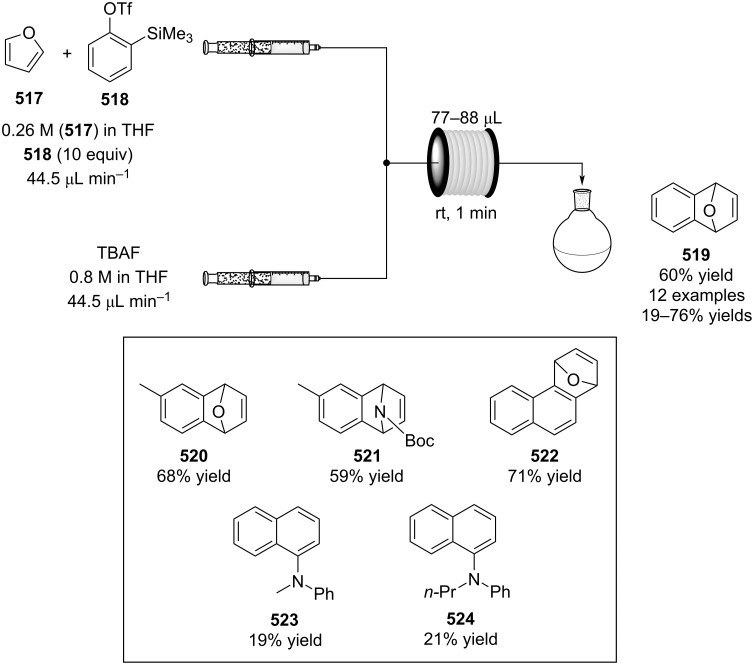
Continuous-flow synthesis of bicyclic systems from benzyne precursors.

As discussed in previous sections, reactive intermediates can be preprared in situ and immediately utilised in consecutive steps of a integrated flow system. An example, of this was developed by Organ et al. where precursor **518** was used to form the benzyne intermediate, which then underwent a DA cycloaddition with cyclic dienes (furan, imidazole) to yield a range of scaffolds **519**–**524** (Scheme 121) [449]. The microflow system was able to rapidly investigate several derivatives allowing the isolatation of 12 examples in low to moderate yields (19–76%).

**Scheme 122:**
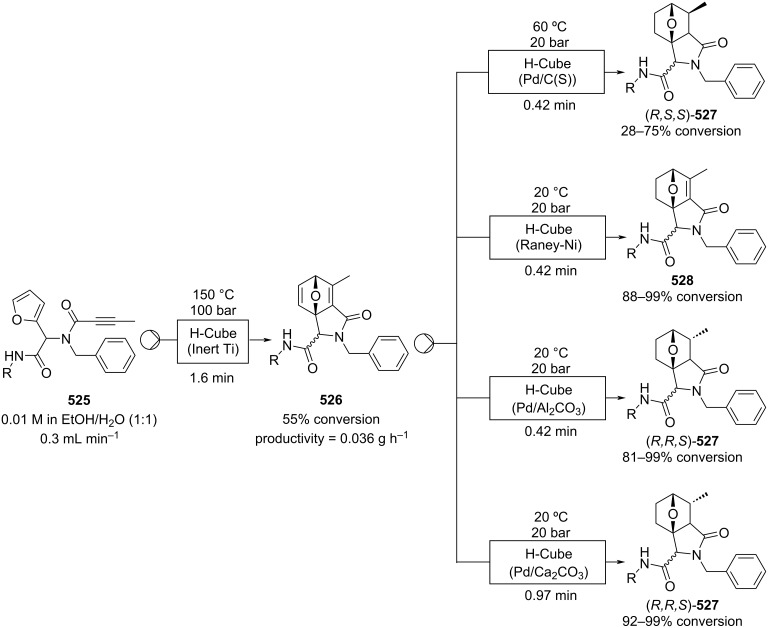
Continuous-flow synthesis of bicyclic scaffolds **527** and **528** for further development of potential pharmaceutical compounds.

In 2018, Gordon and co-workers optimised a continuous-flow protocol for the development of new drug compounds. The authors exploited an H-Cube^®^ Pro system, which can provide high pressures and temperatures, to facilitate an intramolecular DA reaction [450]. The apparatus allowed access to the key intermediate **526** in higher throughput than the previously optimised batch mode (0.002 g h^−1^ vs 0.035 g h^−1^). The obtained scaffold **526** then underwent reduction using the same H-Cube^®^ Pro but using different cartridges (Scheme 122). This example shows how a system can be easily reconfigured for different flow purposes.

**Scheme 123:**
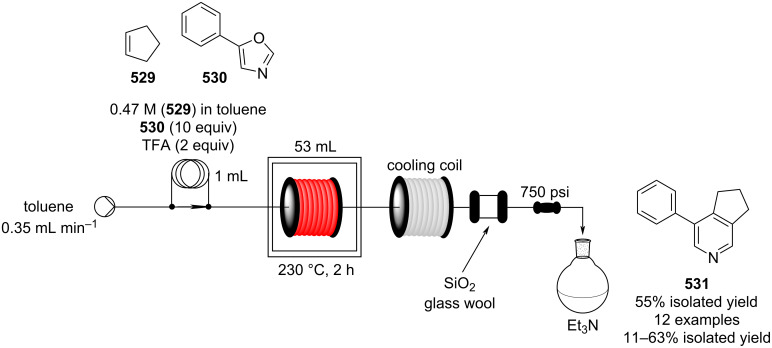
Continuous-flow inverse-electron hetero-DA reaction to pyridine derivatives such as **531**.

Inverse-electron demand hetero-DA reactions require high temperatures to occur, therefore high boiling point solvents are often needed, many of these are inherently toxic and obviously difficult to remove. The works of Britton and Martin show how flow chemistry can offer simple ways to address this issue [451–452]. In these examples, they employed a lower boiling point solvent such as toluene at high temperature (250 °C) and high pressure (750 psi) [452]. This was achieved using a modified GC oven with a stainless steel coil reactor inserted inside. The mixture of **529** and **530** in toluene was pumped through the coil reactor heated at 230 °C under a pressure 750 psi (Scheme 123). After 2 hours of residence time, the mixture was passed through a small column filled with silica gel and glass wool to prevent any clogging of the BPR. The flow system allowed to isolated the pyridine derivative **531** in 55% yield. The authors adopted the apparatus for the preparation of other pyridine derivatives yielding low to good results (11–63% yield).

A microwave flow reactor has been employed in the cycloaddition of furan (**486**) and diethyl acetylenedicarboxylate enabling the synthesis of the bicyclic structure in good yields and short residence times of around 1 minute [453]. Although promising this area needs additional research in order to streamline the sequence with other potentially interesting modern strategies for DA catalysis such as carbocation catalysis [454].

**Scheme 124:**
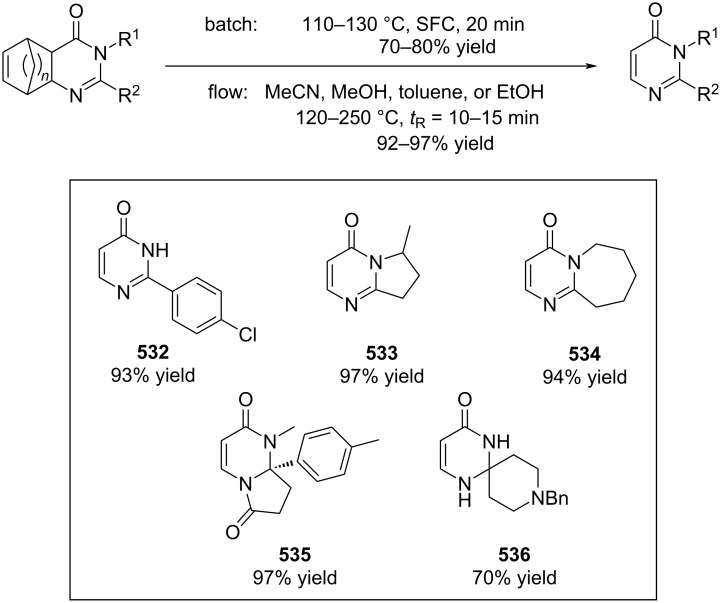
Comparison between batch and flow for the synthesis of pyrimidinones **532–536** via retro-DA reaction using overheating conditions.

Retro-DA reactions have been extensively employed for the synthesis of heterocyclic scaffolds. The reaction requires high temperatures and the setup to avoid degradation process over the desired retro-addition. Flow processing enables precise optimisation of the thermal conditions and because of the reactor containment and applied system pressure the use of low-boiling point solvents become viable. In an example preparation the pyrimidinone scaffolds **532**–**536** was obtained from a retro-DA of the corresponding tricyclic structures (Scheme 124) [455–456]. Using the appropriate solvents in flow under high pressure (BPR 300 bar) the desired pyrimidinone products could be isolated in higher yields compared to the corresponding batch reaction.

**Scheme 125:**
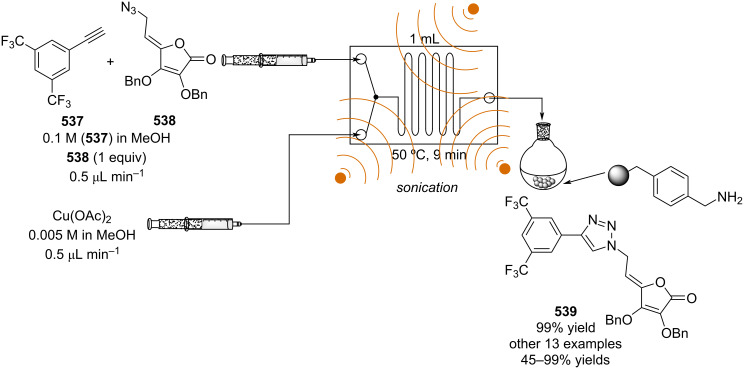
Continuous-flow coupled with ultrasonic system for preparation of ʟ*-*ascorbic acid derivatives **539** developed by Raić-Malić et al.

Several [2 + 3] cycloadditions have been developed in continuous-flow over the years, and some of these have been recently reviewed, such as the copper-catalysed version of Huisgen reaction [457–459]. The latter consists of a 1,3-dipolar cycloaddition between an azide and alkyne to form the 1,2,3-triazoles as a mixture of 1,4- and 1,5-adducts. To ensure the formation of only one regioisomer, few metal-catalysed versions have been developed [460–462]. The CuAAC (Cu-catalysed alkyne–azide cycloaddition) allows the selective formation of the 1,4-adduct and has found a lot of interest particularly in medicinal chemistry. Several flow intensified processes have been described in the last few years permitting a reduction in reaction times due to the improved mixing and precise control of the reaction conditions. Raić-Malić and co-workers assembled a continuous-flow reactor coupled with an ultrasound system for the synthesis of ʟ*-*ascorbic acid derivatives such as **505** (Scheme 125) [463].

The output stream was collected into a batch reactor and quenched using a benzylamine scavenger resin. With this apparatus, the reaction times were reduced from hours to minutes, increasing the throughput and reducing the time for the preparation of a compound series.

**Scheme 126:**
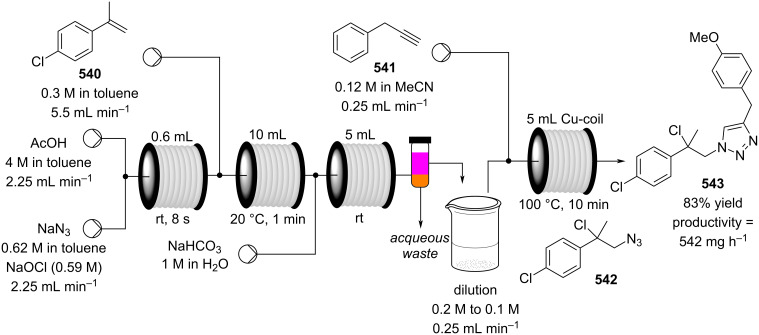
Two-step continuous-flow synthesis of triazole **543**.

Flow chemistry also enables better handling of the hazardous azide reagents [464–465]. In 2016, Vögtle et al. optimised a continuous preparation of 2-chloroazides to utilise in CuAAC (Scheme 126) [466].

The system employed one of the most challenging reagents to use, chlorine azide (ClN_3_). The reagent was prepared in situ and directly utilised for the 1,2-azidochlorination of the olefin **540**. After the reaction, the excess azide was quenched in-line using NaHCO_3_ and the resulting biphasic mixture separated in flow using a Zaiput membrane separator. The product **542** was obtained as a 0.2 M solution which was diluted for the CuAAC reaction. To enact the coupling a copper coil reactor was employed, using a 0.1 M solution which increased the substrate-to-catalyst ratio, improving the conversion. The flow system enabled isolation of **543** via solvent evaporation in 83% yield in a throughput of 542 mg h^−1^.

**Scheme 127:**
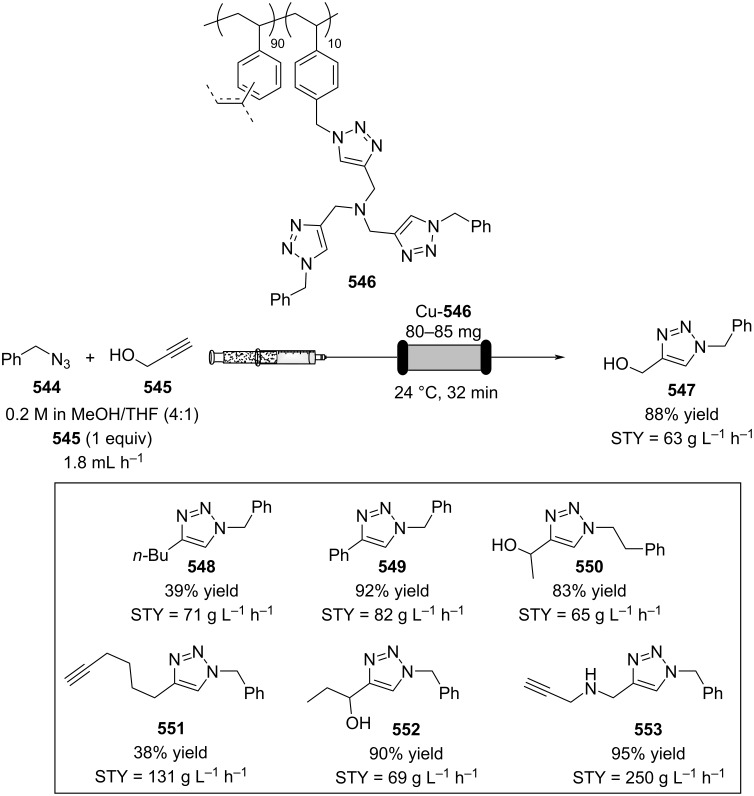
Continuous-flow preparation of triazoles via CuAAC employing **546**-based heterogeneous catalyst.

Immobilisation of copper catalysts has been further developed; alongside the usage of copper coils, already described by Tranmer and Fülöp [457–458,467–468], other new heterogeneous formats have also been reported [469–473]. As an example, in 2020, a polymer-supported tris-triazole ligand was prepared and utilised for Cu immobilisation [474]. The CuAAC was carried out in a monolith and a packed-bed reactor for comparison, and the latter proved to be more efficient, probably due to a higher contact area. To form the active Cu(I) species, sodium ʟ*-*ascorbate was continuously streamed along with the reactants (Scheme 127). The packed reactor was investigated for the preparation of 8 substrates and the catalyst showed reduced activity only after roughly 20 hours of operation providing STYs of 63-250 g L^−1^ h^−1^.

**Scheme 128:**
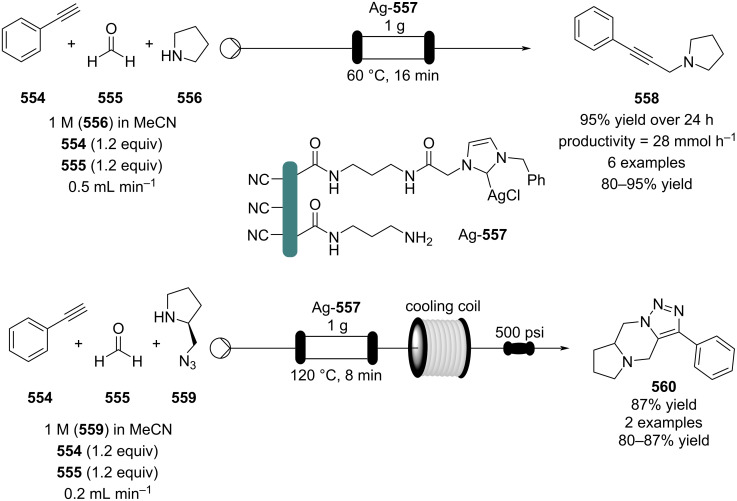
Continuous-flow synthesis of compounds **558** through A3-coupling and **560** via AgAAC both employing the polyacrylonitrile-supported catalyst Ag-**557**.

Silver-catalysed alkyne–azide cycloaddition (AgAAC) have also been investigated under flow conditions. In 2017, Zhang and co-workers developed a immobilised silver catalyst, linked to an *N-*heterocyclic carbene (NHC) ligand itself tethered to a polyacrylonitrile fibre (Ag-**557**) which could be packed into a column reactor for use in flow (Scheme 128) [475]. The authors described a three-component reaction (A3-coupling reaction) catalysed by Ag-**557** under flow conditions, along with a domino A3-coupling/AgAAC. In A3-coupling reaction, the column was heated to 60 °C and after a 16 minutes residence time compound **558** was isolated in 95%. The flow apparatus was run for 24 hours giving no signs of catalyst deactivation with a productivity of **558** of 28 mmol h^−1^. The domino A3-coupling/AgAAC reaction was setup at a higher temperature (120 °C) to complete the reaction. The use of a back pressure regulator (BPR) avoid the need to change the solvent as the pressure is increased to 500 psi.

Along with the Huisgen-type reactions, many other 1,3-dipolar cycloadditions have been developed in flow over the years. Flow conditions have found great utility in the synthesis of a diverse range of scaffolds such as pyrrolidines [476–479], pyrroles [480], pyrazoles [481–483], tetrazoles [484–485], isoxazoles [486–488], and bicyclic compounds [489–491].

**Scheme 129:**
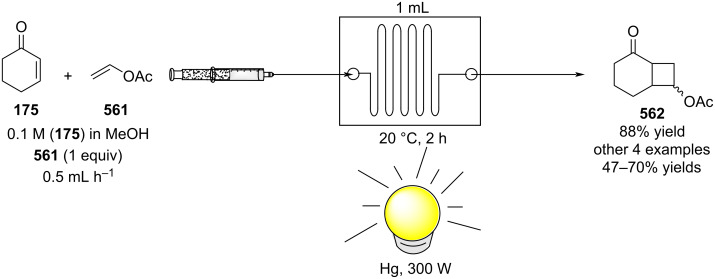
Continuous-flow photoinduced [2 + 2] cycloaddition for the preparation of bicyclic derivatives of **562**.

In addition [2 + 2] cycloadditions have also been of great interest in organic synthesis as cyclobutanes are key intermediates for the preparation of larger rings (via ring expansion) as well as enabling formation of interesting 4-membered rings such as lactam and lactones [492–493]. The [2 + 2] cycloadditions involve an olefin at its singlet/triplet excited state (SOMO) and a alkene at its ground state (LUMO) [494]. Photoreactions performed in batch mode present various limitations, such as low selectivity, and long reaction times. Since the light absorption for a photoreaction performed in a flask follows the Lambert–Beer law, these issues can be attributed to poor irradiation intensity. To overcome these problems, thin, low volume glassware has been developed, however, only small batches can be run resulting in low productivity. For these reasons, microfluidic flow reactor technology has been shown to be a good fit to reduce the reaction times and increase the efficiency, therefore gaining the desired throughputs [495–497]. Many examples of customised flow photoreactors have been developed and investigated for [2 + 2] cycloadditions [316,498–506]. In one of the early examples, Fukuyama, Ryu, and co-workers developed a continuous-flow photochemical [2 + 2] cycloaddition between enones and vinyl acetate (Scheme 129) [502]. The system utilised a glass microreactor irradiated through one side of the device where the reactants undergo irradiation for 2 hours prior to collection. After confirming the efficiency of the approach compared to the batch one (88% vs 8% yield), the authors investigated other derivatives, also yielding the desired materials in moderate to good yields (47–70%).

**Scheme 130:**
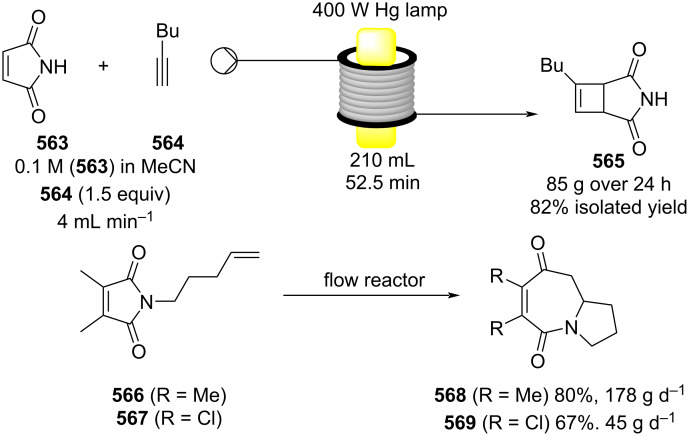
Continuous-flow [2 + 2] and [5 + 2] cycloaddition on large scale employing a flow reactor developed by Booker-Milburn and co-workers.

Working in flow also provides improved options for scaling of photochemical transformations. In 2005, Booker-Milburn et al. developed a flow reactor that could perform a large scale production run of a [2 + 2] cycloadduct **524** (Scheme 130) [316].

In this example, a customised immersion well made in Vycor was tightly wrapped with a fluorinated ethylenepropylene (FEP) tubing (2.7 mm i.d.). Three layers of tubing were coiled around the well, in which was placed a 400 W medium-pressure mercury vapour discharge lamp. The input stream flowed from the outermost layer to the innermost one at a flow rate of 4 mL min^−1^. Using these conditions, a 0.1 M solution of maleimide (**563**) and 1-hexyne (**564**) were combined and reacted over 24 hours through the flow reactor, gaining 85 g of the desired material **565** (isolated yield 82%). The authors also confirmed the usage of a higher power lamp (600 W) allowed an increase in the reaction concentration to 0.4 M with double the flow rate at 8 mL min^−1^ to gain a projected throughput of 685 g d^−1^ when running uninterrupted. The same flow system was also employed for a [5 + 2] cycloaddition for the preparation of azepines **568** and **569**, obtaining again higher throughputs compared to the batch mode.

**Scheme 131:**
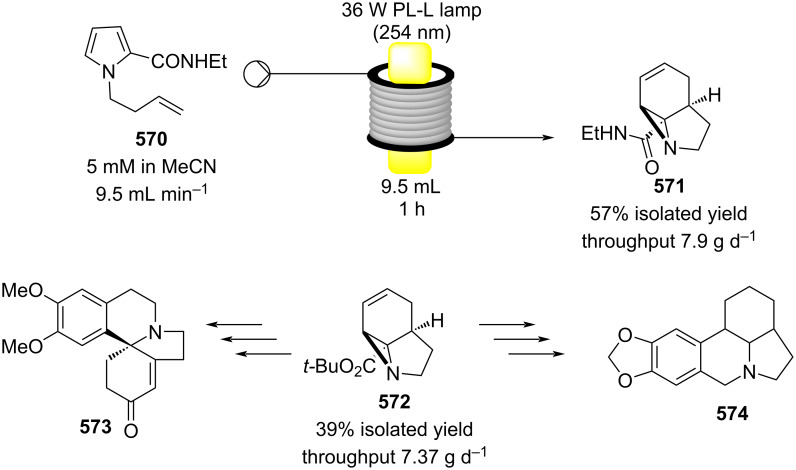
Continuous-flow preparation of the tricyclic structures **573** and **574** starting from pyrrole **570** via [2 + 2] cycloaddition and rearrangement. PL-L lamp: plug-in fluorescent lamp.

The same group has also investigated a series of other [2 + 2] cycloadditions demonstrating their scale-up using flow conditions [507–509]. As an example, in 2013, they developed a methodology for the synthesis of the aza-tricyclic structure **571**, starting from a [2 + 2] cycloaddition reaction of pyrrole **570** (Scheme 131) [510].

In order to increase the throughput, a flow apparatus was employed allowing roughly 8 g of final material to be isolated after 24 hours of runtime (57% isolated yield). The same flow setup used for various other analogues enabled the intermediate structures to be exploit in the synthesis of more complex natural products, such as (+)-3-demethoxyerythratidinone (**573**) and the lycorane alkaloids (illustrated by compound **574**) [511–512].

**Scheme 132:**
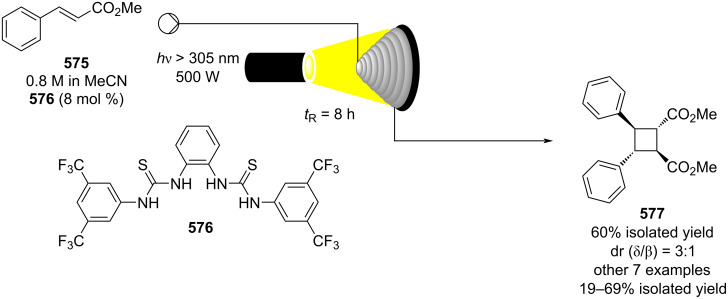
Continuous-flow [2 + 2] photocyclization of cinnamates.

In 2015, Beeler et al. customised a photoreactor where a xenon light source was filtered and focused into a 2 inch beam. A conical coil reactor was placed in front of the light and kept refrigerated. Utilising this flow system, the group developed a thiourea-catalysed [2 + 2] photocycloaddition of cinnamates (Scheme 132) [503].

The first setup consisted of an acetonitrile solution of the reactants passing through the conical flow reactor. However, more recent findings showed that biphasic conditions were more efficient, with slug flow leading to a reduced residence time and higher throughput [504]. Of particular value was that the flow system enabled operation with particularly sensible cinnamates, which had proved problematic during monophasic reactions in batch. The authors attributed these outcomes to the improved mixing, and the smaller UV path length enhancing catalyst-substrate interactions and controlling UV absorbance. These conditions have also proven beneficial when employed for a Paternò–Büchi photoreaction as described by Nishiyama, Kakiuchi and co-workers [513].

Many of the photoinduced reactions employ pressurized mercury lamps as UV light sources. However, they present numerous drawbacks such as high energy consumption, low efficiency (including long start up times), a wide wavelength spectrum, and short bulb lifetimes [514]. As the use of sunlight would be highly desirable, the inhomogeneity of the solar irradiation over time and space makes it quite unfeasible for industrial applications. Therefore, great interest has been seen in the area of high efficiency narrow wavelength light-emitting diodes (LED) as alternative light sources. These systems provide several advantages over the pressurized mercury lamps, although their light spectrum are mainly limited to long wavelengths (>340 nm). To shorten this wavelengths, a catalytic amount of a triplet photosensitizer can be employed, which shortens the long wavelengths to higher-energy waves which is synthetically useful for reactions such as [2 + 2] cycloadditions and other photoreactions.

**Scheme 133:**
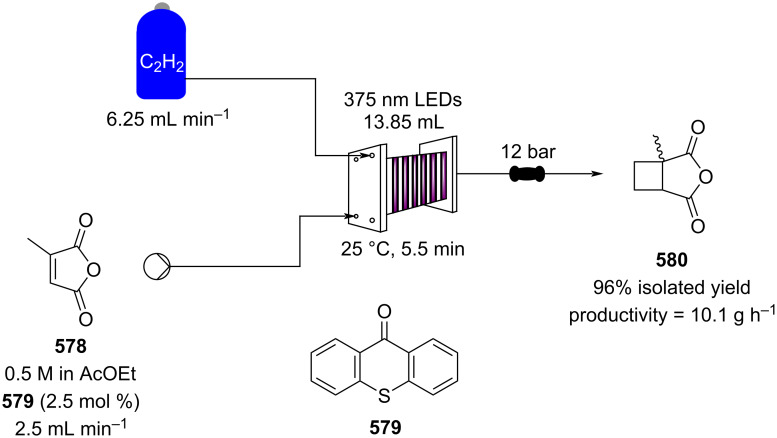
Continuous-flow preparation of cyclobutane **580** on a 5-plates photoreactor.

As an example, Kappe et al. developed a [2 + 2] cycloaddition of maleic anhydride (**578**) and ethylene using 375 nm LED’s as the light source and thioxanthone (**579**) as a photosensitizer (Scheme 133) [515]. The authors first optimised a flow version using benzophenone, however, higher catalyst loading (40 mol %) required an additional purification step at the end. Following optimisation, a 0.5 M solution of methyl maleic anhydride (**578**) and 2.5 mol % thioxanthone (**579**) in ethyl acetate was mixed with ethylene gas and directed into a 2.77 mL plate-based reactor. An array of 375 nm LEDs was used to irradiate the photoreactor to furnish the cyclobutane product **580** in more than 99% conversion and a STY of 759 g L^−1^ h^−1^. Stacking five plates in series allowed a 5-fold scaling up of the process to obtain roughly 100 g of the cyclobutane **580** in 96% isolated yield across a 10 hours runtime (productivity 10.1 g h^−1^) (Scheme 133).

**Scheme 134:**
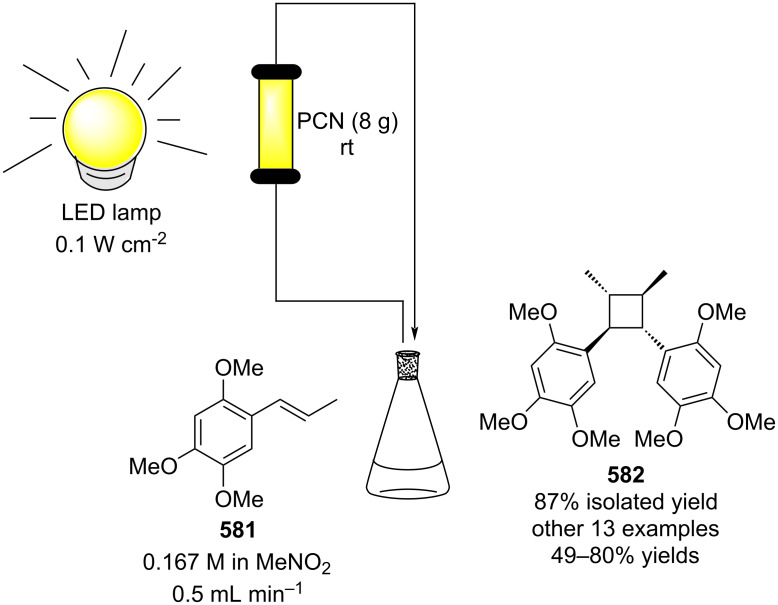
Continuous-flow [2 + 2] photocycloaddition under white LED lamp using heterogeneous PCN as photocatalyst.

Recently, Zhang, Wang and co-workers employed a polymeric carbon nitride (PCN) material as a heterogeneous photocatalyst for [2 + 2] cycloadditions under white LED lighting (Scheme 134) [516].

The PCN catalyst was prepared as a deposit on commercial glass beads which were packed into a fixed-bed photoreactor where a solution of α-asarone (**581**) or other styrene derivatives in a solution of nitromethane was flowed through. Even though, the flow system provided a better tool than a reactor batch (53% vs 87%), interestingly the reaction times were longer for most of the examples explored (24–48 h). This was not investigated or explained but is probably due to reduced catalyst contact times in the flow system.

**Figure 11:**
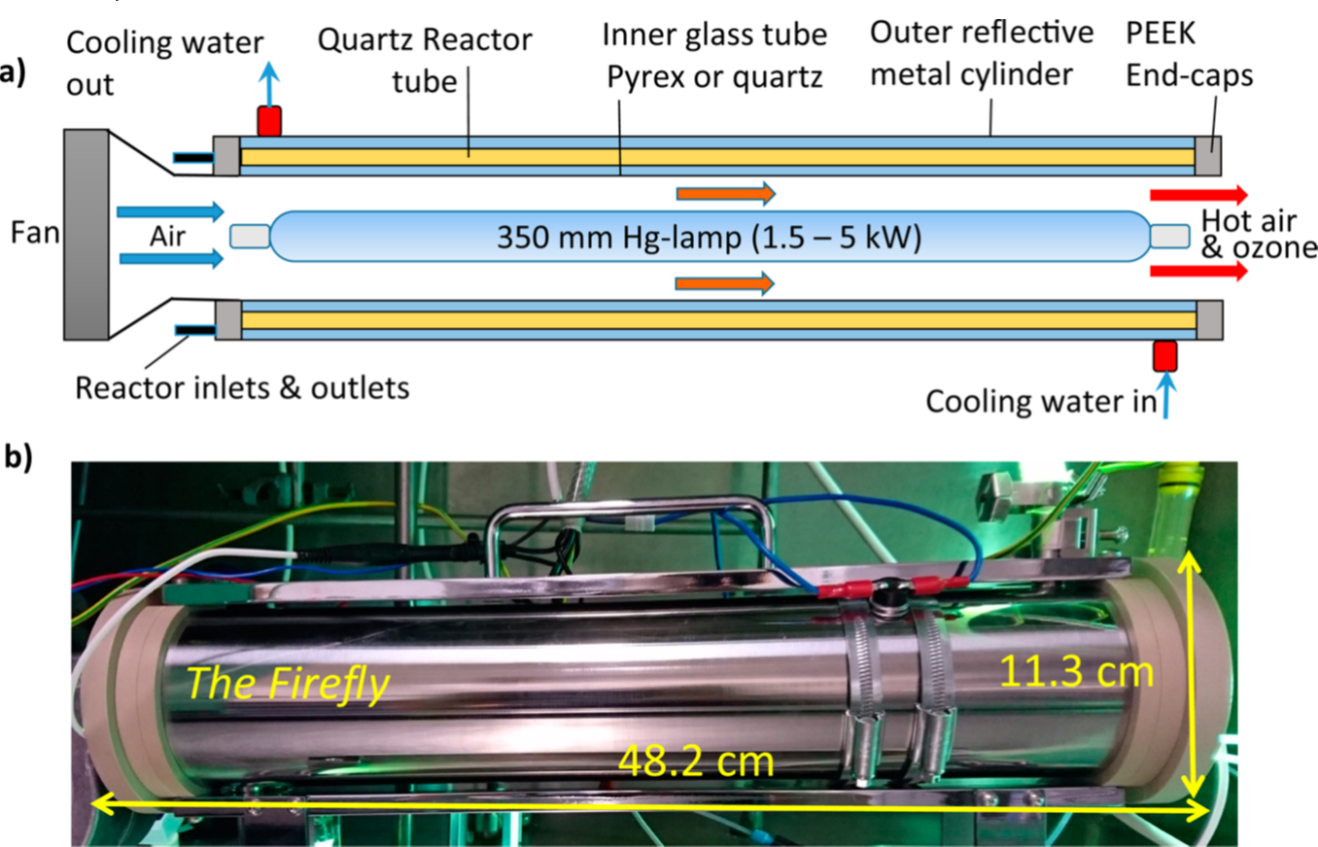
Picture of the parallel tube flow reactor (PTFR) "The Firefly" developed by Booker-Milburn et al. a) Diagram of the photoreactor. b) Rector from the outside [517]. Reprinted with permission from [517]. Copyright 2016 American Chemical Society.

In 2016, the group of Booker-Milburn developed an innovative photoreactor for the preparation of compounds at the kilogram scale [517]. The apparatus nicknamed “The Firefly” (Figure 11) was made from quartz reaction tubes surrounding a powerful Hg lamp (1.5 kW–5 kW of power) and cooled using fans and a water cooling jacket. The reactor had a 120 mL internal volume. To increase the light absorption even at the outermost part of the pipes, a reflective metal film was wrapped around the outside of the reactor.

Seven different photochemical transformations were investigated using “The Firefly” device and in all cases high throughputs were achieved (Table 13). Noteworthy to mention is the intramolecular [2 + 2] cycloaddition of the quinone **589** to Cookson’s dione (**590**) that occurred in high yields and a throughput of roughly 8 kg of material per day (Table 13, entry 5). The authors pointed out how productivity is directly proportional to the power of the light source as the synthesis of the trichloro compound **585** provides 2.85 g h^−1^ in a 400 W FEP reactor and 28.8 g h^−1^ using 3 kW-powered PTFR.

**Table 13:** Photochemical reactions exploited using the parallel tube flow reactor "The Firefly".

Entry	Reaction	Lamp power (kW)	Yield (%)	Productivity (g d^−1^)

1	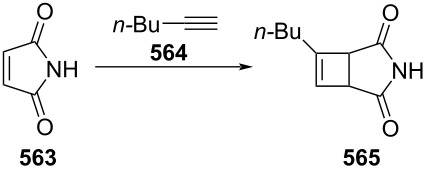	3	65	605
2	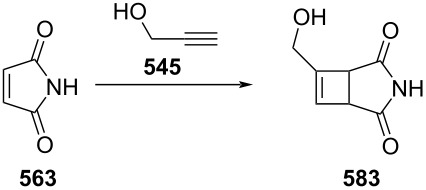	3	64	509
3	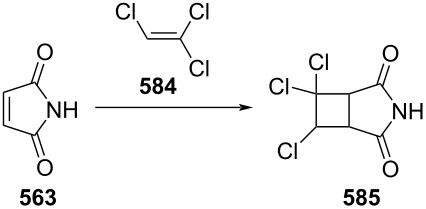	3	66	691
4	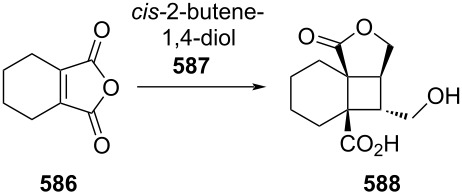	3	80	3,984
5	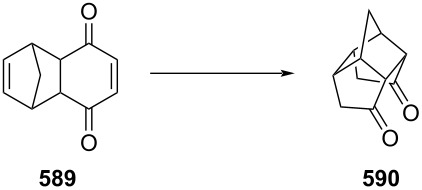	1.5 3	89 89	4,008 8,058
6	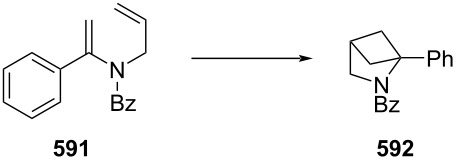	3	86	1,174
7	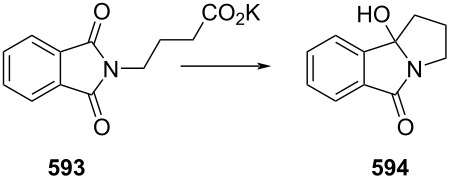	3	85	1,390

**Scheme 135:**
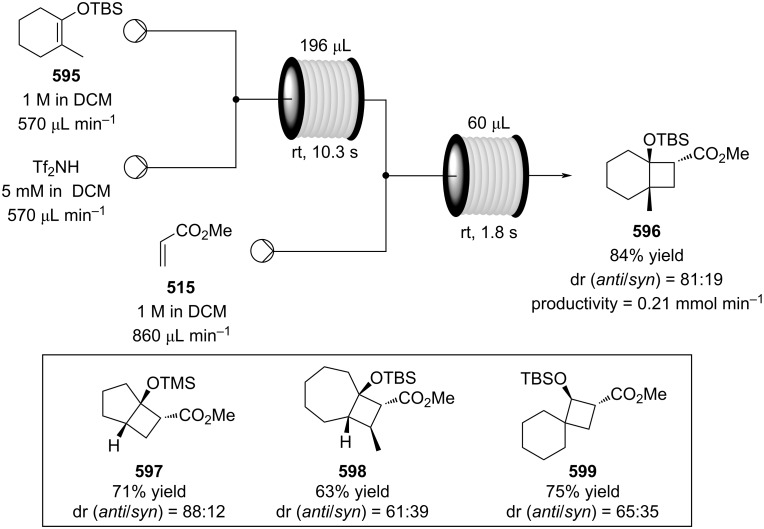
Continuous-flow acid-catalysed [2 + 2] cycloaddition between silyl enol ethers and acrylic esters.

Silyl-protected hydroxycyclobutanes (e.g., **596**) were prepared in flow from silyl enol ethers and α,β-unsaturated esters via an acid-catalysed [2 + 2] cycloaddition [518]. The orbital symmetry of the reacting partners allows the reaction to proceed without the need for light, however, a catalyst is required. The in situ-formed silyl triflic imide (Tf_2_NH) employed as the catalyst proved both efficient and selective for a range of different substrates; however, in few examples oligomerization, polymerisation and product decomposition was observed in minor quantities. Takasu and co-workers setup a flow reactor which reduced the reaction time and temperature, thus eliminating the formation of byproducts. The system consisted of two Y-shape micromixers and two reaction microtubes. The silyl enol ether was first mixed with the Tf_2_NH to form the active silyl triflic imide catalyst. The reaction mixture then merged with the enophile **515** to yield the desired cyclobutane. Under flow conditions, the reaction occurred at higher temperature (rt vs −78 °C) and faster reaction times (12 s vs 2 h) compared with the optimised batch reactor. The ability to efficiently control the reaction timing, thereby limiting the formation of undesired side products, and to adjust the residence times for less reactive substrates enhanced the yields. In this way, more Lewis acid sensitive silyl enol ethers could be used and the desired cyclobutanes such as **596**–**599** were isolated in good yields (Scheme 135).

**Scheme 136:**
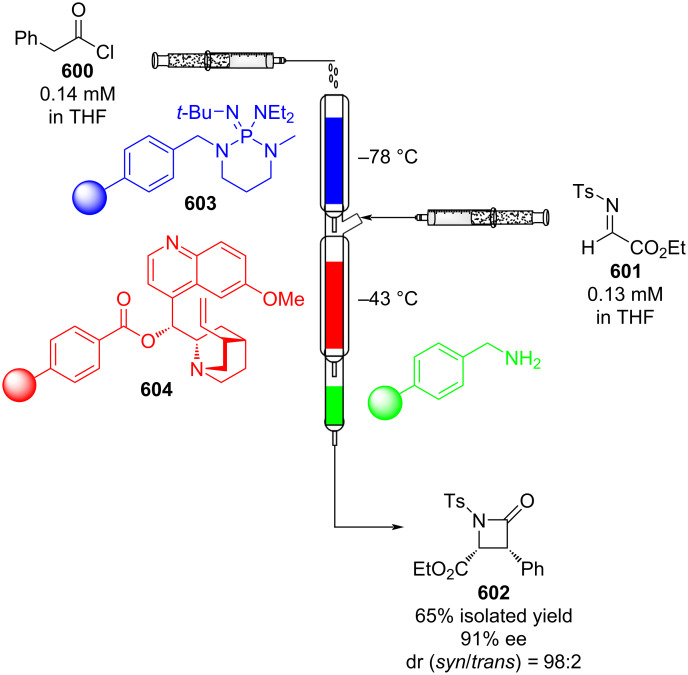
Continuous synthesis of lactam **602** using glass column reactors.

Staudinger [2 + 2] cycloaddition for the synthesis of β-lactams occurs between an imine and ketene. The latter is a highly reactive species usually generated in situ to avoid decomposition through dimerization or polymerisation. As flow chemistry allows to efficiently handle reactive materials, several flow systems have been described over the years to aid this chemistry. A flow approach was first reported by Lectka et al., the system consisted of two jacketed columns joined together and filled with polymer-supported reagents (Scheme 136) [519–520]. In the first glass column the ketene was formed from an acyl chloride **600** using the BEMP-resin **603** as base at −78 °C. The outflow was directed into the second column where it was combined with a stream of α-imino ester **601** (solution in THF). The desired [2 + 2] cycloaddition took place at a reduced temperature of −43 °C using a solid-phase chiral catalyst **604**. After 2 hours complete elution of the reaction mixture occurred and the lactam product **602** was isolated enantiomerically pure in 65% yield.

**Scheme 137:**
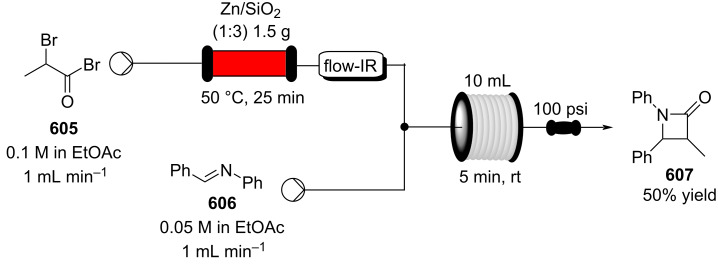
In situ generation of ketenes for the Staudinger lactam synthesis developed by Ley and Hafner.

Ley and Hafner described a different approach for the ketene generation. Starting from α-haloacyl halides such as **605**, the ketene was prepared by a zinc-mediated dehalogenation [521]. The flow system employed a zinc-packed column at rt (Scheme 137). The generation of the reactive species was controlled using an in-line flow IR spectrometer. Using this combination of apparatus, the lactam **607** was obtained after just 5 minutes in 50% yield. Better results (98% conversion) were obtained when diethyl ether was employed in a combined flow-batch mode. Due to high volatility of the ether solvent, the authors preferred to use an easier to handle solvent such as ethyl acetate.

Other continuous-flow systems in which a ketene was generated in situ and then directed into a [2 + 2] cycloaddition have been reported, many of these reactions present improved results compared to their batch modes with higher throughputs [522–523].

**Scheme 138:**
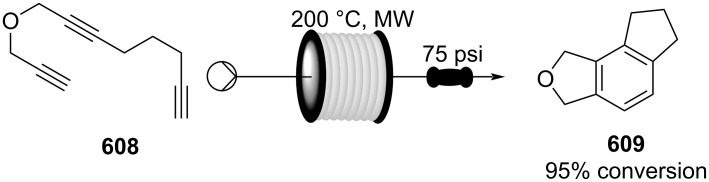
Application of [2 + 2 + 2] cycloadditions in flow employed by Ley et al.

In a related approach a [2 + 2 + 2] cyclotrimerization reaction was developed in flow. The flow mode was found particularly beneficial due to the harsh reaction conditions required and the ability to scale the reaction [524–525]. The system was based upon microwave irradiation instead of thermal heating, a glass coil was inserted into a standard microwave cavity of a Biotage reactor (Scheme 138) [525–526]. In this example, the product **609** was detected in 95% conversion within a 30 minutes residence time.

#### Friedel–Crafts reactions

**Scheme 139:**
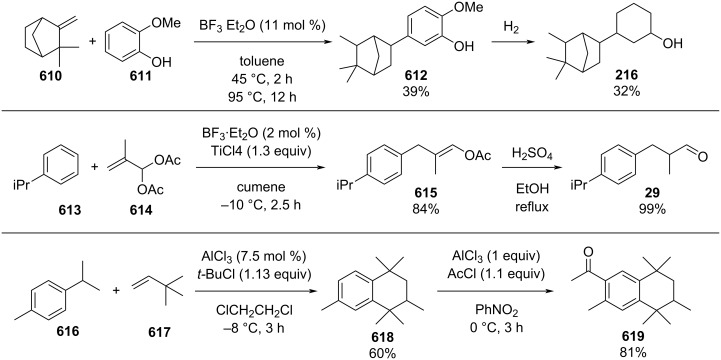
Examples of FC reactions applied in F&F industry.

Friedel–Crafts (FC) reactions have been exploited numerous times for the synthesis of F&F compounds (Scheme 139). A few important examples include isocamphylcyclohexanol (**216**) from guaiacol (**611**) and camphene (**610**) [220], and the synthesis of cyclamenaldehyde (**29**) from cumene (**613**) [527]. Generally, musky odorants present a terpenoid-like skeleton which is usually obtained through a FC cyclisation as in the case of musk tetralin (**619**) prepared from *p*-cymene (**616**) and neohexene (**617**) [528–529].

FC reactions typically require elevated temperatures and also Lewis acids such as aluminium trichloride in high stoichiometry, however, an enormous amount of waste salts are formed as byproducts. To prevent this waste, many heterogeneous catalysts have been investigated and described [530–531]. Flow chemistry has the potential to improve the reusability of these catalysts as has been previously discussed in this review (see the chapters ‘The advantages of a flow approach’ and ‘Condensation reactions’). One of the first papers describing FC reactions in a flow system came in 1977 from Toyoshima and Arata, where they optimised calcinated iron sulfate-catalysed FC reactions between toluene and isopropyl chloride in the gaseous phase [532]. As a first example, low conversions (10–20%) were measured, however, selectivity and conversions remained steady over 50 minutes of runtime.

Over two decades later, Poliakoff et al. described a FC alkylation exploiting supercritical fluids such as scCO_2_ to achieve faster purification steps [533]. A sulfonic acid supported on polysiloxane (Deloxan^®^) was employed as catalyst for the FC reaction of mesitylene with isopropanol. For this system, two different supercritical fluids (scCO_2_ and scPropene) were investigated, and scCO_2_ proved more selective. The optimised flow apparatus allowed a modest yield of 290 mg min^−1^ of mono-alkylated material in 19% yield.

**Scheme 140:**
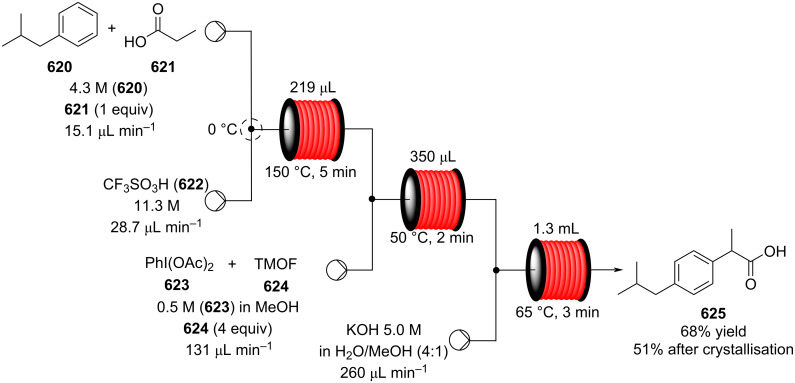
Continuous-flow synthesis of ibuprofen developed by McQuade et al.

In 2009, McQuade and co-workers achieved the FC reaction of isobutylbenzene (**620**) and propionic acid (**621**) in flow for the synthesis of ibuprofen (**625**, Scheme 140) [534].

**Scheme 141:**
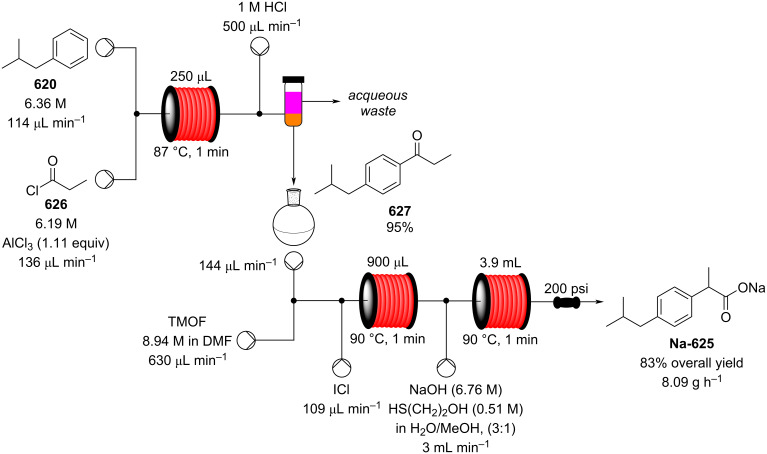
The FC acylation step of Jamison’s three-step ibuprofen synthesis.

For this setup, the authors chose to use triflic acid as the Lewis acid. Aluminium chloride proved to be more efficient, however, its byproducts were found detrimental for the subsequent steps. The neat stream of substrates **620** and **621** was mixed with triflic acid (**622**) in a tee junction cooled at 0 °C and then passed through a microreactor at 150 °C. After 5 minutes, the precursor **627** (see structure of **627** in Scheme 141) was isolated in 95%. The overall synthesis of ibuprofen consisted of three microreactors; after the acylation step, a second coil at 50 °C was setup for the 1,2-aryl migration, and in the third warmed at 65 °C where the methyl ester is hydrolysed to the final product **625**.

More recently, an alternative continuous synthesis of ibuprofen (**625**) was reported by Jamison and Snead (Scheme 141) [535].

In this example, the aluminium chloride was solubilised in the propionyl chloride (**626**) and streamed through a coil reactor at 87 °C along with the isobutylbenzene (**620**). The flow stream was then quenched with HCl to terminate the reaction and avoid clogging of the insoluble aluminium salts. A separator membrane was then employed to separate the intermediate **627** from the aqueous waste. The organic phase containing **627** was collected in a separate flask from which it was then pumped and directly employed for the next steps to form ibuprofen. Additional flow processing of **627** afforded the ibuprofen sodium salt **Na-625** in high yield (83%), resulting in a short three-step continuous ibuprofen synthesis. The system allowed a production of 8.09 g h^−1^ of **Na-625**.

**Scheme 142:**
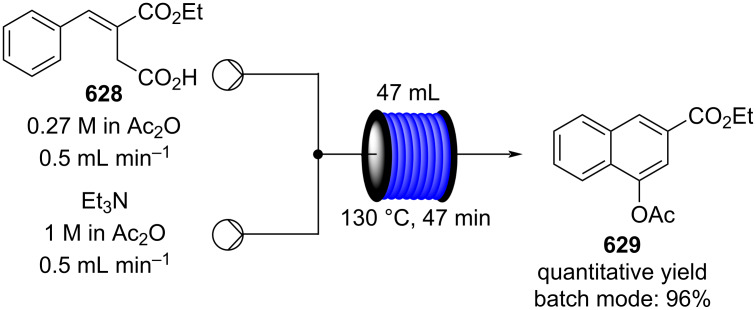
Synthesis of naphthalene derivative **629** via FC acylation in microreactors.

Intramolecular FC acylation using a microreactor setup allowed the synthesis of naphthalene derivative **629** in quantitative yield whilst also drastically reducing the reaction time (Scheme 142) [536]. Recently, microreactors have been used for direct acylation on naphthalene substrates. A solution of propionyl chloride (**626**, 1.3 equiv) and AlCl_3_ (1.5 equiv) in nitrobenzene was mixed at 0 °C with 2-methylnaphthalene and then streamed through a micro-channelled reactor at 40 °C for 60 minutes. The authors claimed high selectivity (87.5%) and yield (85.8%) [537].

**Scheme 143:**
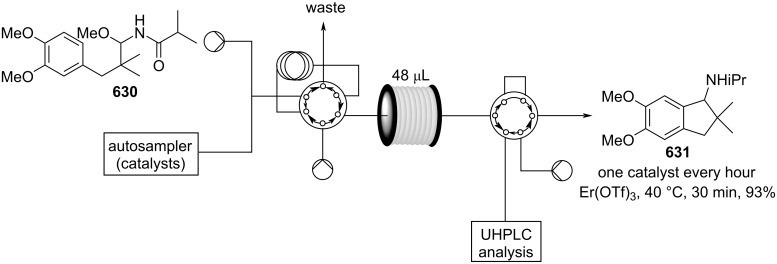
Flow system for rapid screening of catalysts and reaction conditions developed by Weber et al.

Finding the optimal reaction conditions remains a time and resource-consuming process. Flow chemistry techniques can expedite these investigations, drastically reducing the resource required during the process optimisation stages. A rapid catalyst screening system was developed by Weber, Floreancig and co-workers employing a system of loop injectors connected to a UHPLC (ultra-high performance liquid chromatography) system [538]. The loop injector creates individualised reaction zones where different catalysts can be screened (Scheme 143).

The authors explained no mixing between reaction zones occurs due to the narrow diameter of the capillaries and the low flow velocity, which suppress radial diffusion of reagents. At a flow rate of 0.9 μL min^−1^, roughly one catalyst per hour could be sequentially screened (it should be noted that parallel systems could be run). The reactor was applied to the intramolecular FC acylation of acylaminal **630** to the *N-*acyl amine **631**. The authors found that 20 mol % of Er(OTf)_3_ at 40 °C for 30 minutes was optimal furnishing 93% yield. When the catalyst was employed in the related batch reaction, the system down-performed, requiring an increase in the amount of catalyst (1 equivalent) and a prolonged reaction time (8 hours).

**Scheme 144:**
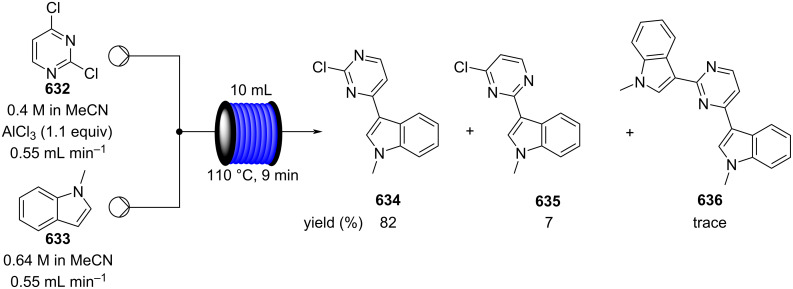
Continuous-flow system developed by Buorne, Muller et al. for DSD optimisation of the FC acylation to **634**.

Similarly, Bourne, Muller and co-workers developed a multistep kinetic model for use with flow conditions [539]. Indeed, flow chemistry has enhanced the ability of organic chemists to study and develop kinetic models of reactions creating reproducible conditions for scale-up. In their study the authors investigated the formation of the pharmaceutical intermediate **634** starting from 2,4-dichloropyrimidine (**632**) and 1-methylindole (**633**). This FC reaction can also lead to other byproducts such as **635** and the dimer **636**. The flow apparatus consists of two HPLC pumps connected via a T-piece to a temperature regulated tubular coil reactor. In order to maximise the yield and reduce the byproduct formation, they developed a kinetic model employing a definitive screening design (DSD), augmenting the level of information acquired and reducing the number of experimental runs. The simulation determined the optimal conditions (Scheme 144) where the lowest amount of byproducts **635** (7%) and **636** (trace) was formed. These results were comparable to the batch optimisation process (82% yield).

**Scheme 145:**
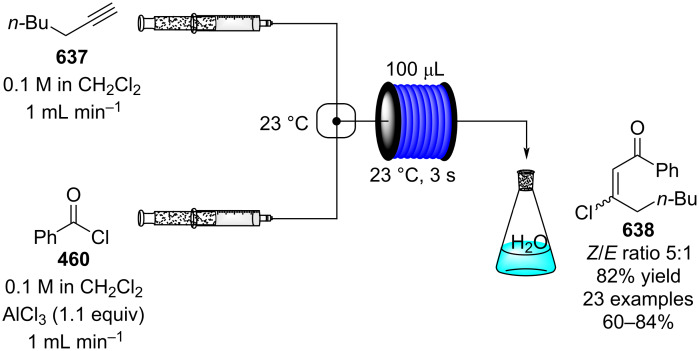
Continuous-flow FC acylation of alkynes to yield β-chlorovinyl ketones such as **638**.

In 2019, Oh and co-workers developed a flow system for the AlCl_3_-catalysed FC reaction of acyl chlorides to alkynes to yield β-chlorovinyl ketones [540]. The reaction in batch mode provided mixtures of the *Z*- and *E-*isomers due to a AlCl_3_-alkyne complex formation, which promotes isomerisation. The authors found that by reducing the reaction times this process was decreased, therefore a mixing chip (MiChS β-type) with a secondary residence time loop (100 μL) used for the task (Scheme 145).

A 0.1 M solution of the alkyne in dichloromethane was mixed with a solution of the acyl chloride **460** and AlCl_3_ in the mixing chip at 23 °C and then sent to the loop reactor for 3 seconds. The output stream was immediately quenched in a conical flask with water. The β-chlorovinyl ketone **638** was obtained in 82% yield, with a 5:1 *E*/*Z* ratio. The substrate was later employed in a flow preparation of isoxazoles through the reaction with vinyl azides [541].

**Scheme 146:**
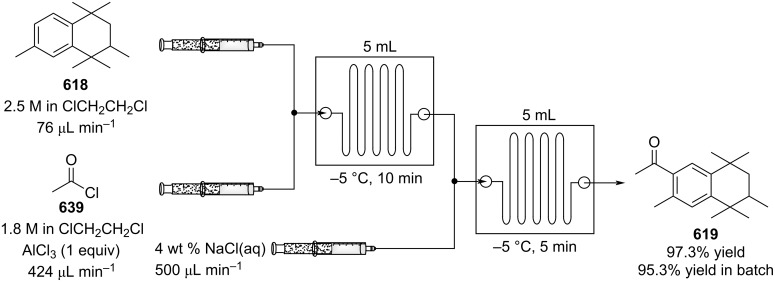
Continuous-flow synthesis of tonalide (**619**) developed by Wang et al.

Very few examples of flow conditions applied to F&F chemicals have recently been reported. However, as previously highlighted, the FC acylation is thought of paramount importance to the industry. Recently, Wang and co-workers developed a flow system for the preparation of tonalide (**619**), from 1,1,3,4,4,6-hexamethyltetralin (**618**) [542]. A 2.5 M stream of **618** in 1,2-dichloroethane was mixed with acetyl chloride (**639**) and the catalyst in a T-shaped mixer and through a microchannel module at −5 °C (Scheme 146). The reaction mixture was then quenched with an aqueous solution of sodium chloride. The system allowed formation of **619** in 97.3% yield, which resulted in a more efficient system then the classic batch mode (95.3%).

**Scheme 147:**
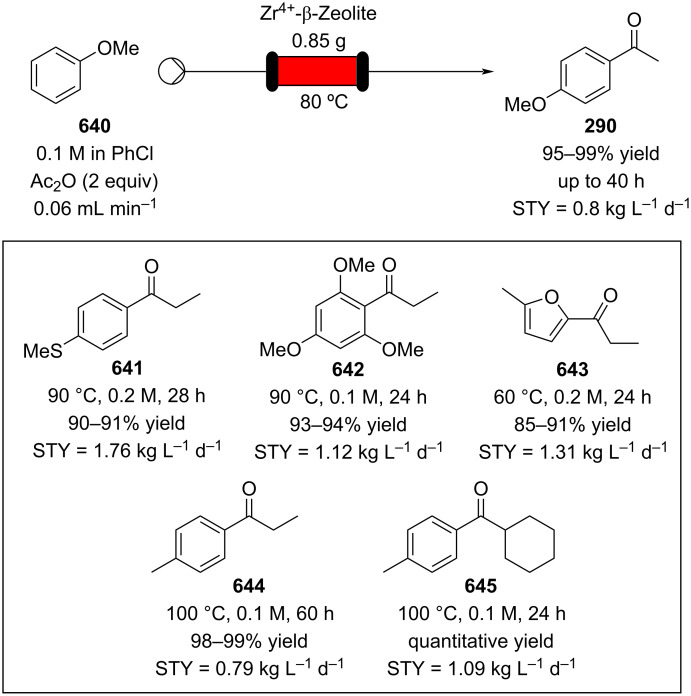
Continuous-flow preparation of acylated arene such as **290** employing Zr^4+^-β-zeolite developed by Kobayashi et al.

Heterogeneous catalysis for FC acylation is still a hard task to achieve due to rapid catalyst deactivation attributed to the adsorption of either byproducts or reactants onto the active sites, bringing about a sudden decrease in the catalytic activity. Aribert and co-workers developed a continuous-flow system employing zeolite Y as the catalyst. After a single use, the yield dropped drastically below 20% from 80%, and calcination is required to re-establish the activity [543]. In total twelve byproducts along with other heavy molecules (“coke”) were found by Zeng et al. on the surface of an HBEA zeolite (acidic form of the β-zeolite polymorph A) when exploited for continuous FC acetylation of toluene. The authors noticed an enhancement in activity using an excess of toluene and acetic acid as an additive [544]. A decrease of the zeolite’s stability was also described by Kobayashi et al. in 2019 [545]. The authors noticed a reduction of aluminium content in the zeolite structure, which inevitably brought about a decrease in activity from 94% down to 59% yield (**640** → **290**) after 28 hours of usage. To their delight, they found that doping the β-zeolite with zirconium cations increased the catalyst stability. The flow system was tested on up to 20 molecules, gaining the acylated materials in 70–99% yield (Scheme 147). The catalyst could be used for up to 5 days without any substantial loss of activity. The flow system employed either chlorobenzene or the arene substrates themselves as the solvent.

Heterogeneous catalysts other than zeolites have also been described lately, such as the α-Fe_2_O_3_ nanoparticles modified by Guo et al. [546], and the metal organic framework (MOF) with zirconium oxides on silica gel developed by Lin and co-workers [547]. These systems can be employed successfully for up to 4 hours prior to loss of activity in a flow reactor.

**Scheme 148:**
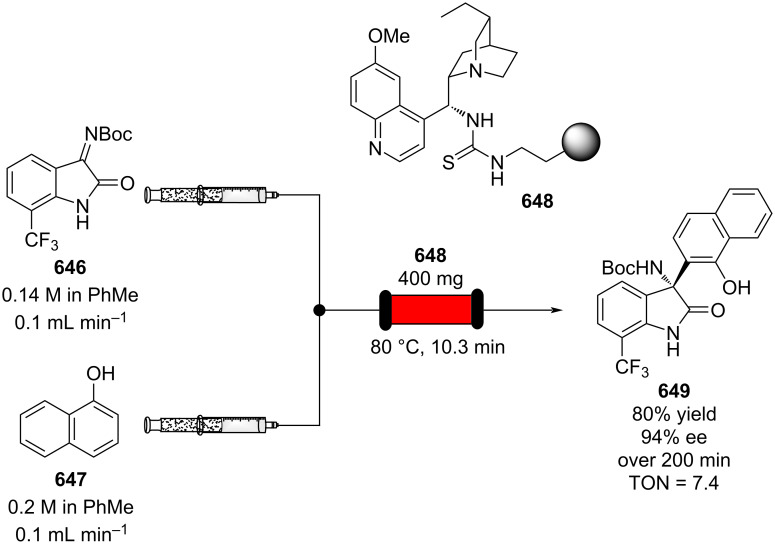
Flow system applied on an Aza-FC reaction catalysed by the thiourea catalyst **648**.

An area which is starting to gain much more interest is the use of immobilised asymmetric catalysts. A highly robust packed-bed column reactor containing a supported phosphoric acid catalyst for enantioselective catalysis of FC reactions involving indoles [548] in flow was reported in 2014 [549]. Recently, a hydroquinine-based polymer supported catalyst **648** has also been developed by the same group for the continuous Aza-FC reaction of α-naphthol (**647**) with 2-amino-2-oxindole derivative **646** [550]. The flow system was allowed to run over 400 minutes to yield the product **649** in 80% yield and 94% ee (Scheme 148). The authors pointed out the reduce reaction times (10.3 min vs 3 h) compared to the batch mode and a productivity of **649** of 2.2 mmol mmol_cat_^−1^ h^−1^.

This area is still relatively underdeveloped due to the complexity and challenges inherent in generating asymmetric catalysts on solid support but will evidently become of greater importance.

#### Hydroformylation

Hydroformylation is a powerful and atom-efficient technique used for the introduction of formyl groups by conversion of alkene functions, often with high selectivity. Aldehydes are among the most important groups of molecules to the F&F industry as both starting materials and also as products. It is reported that 2-methyldecanal and 2-methylundecanal can be prepared from 1-decene (**366**) via a hydroformylation process [551]. As with the other reaction types that require a gaseous reagent supply, flow chemistry presents several advantages for reasons already discussed, such as safety, ease of gas delivery and control over reagent exposure times and stoichiometry.

**Scheme 149:**
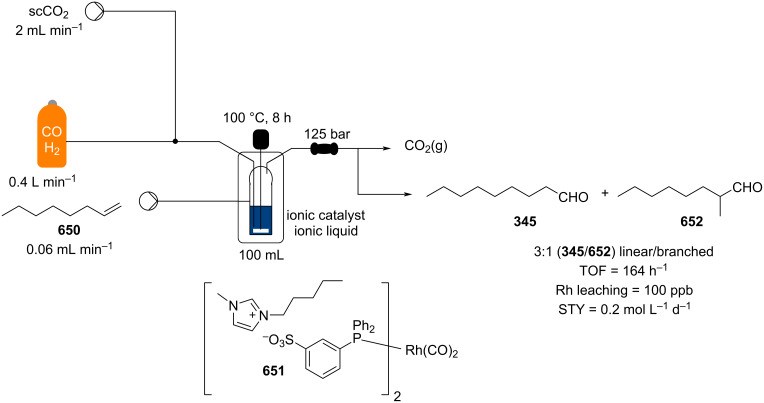
Continuous hydroformylation in scCO_2_.

In one specific reaction, Cole-Hamilton et al. employed scCO_2_ and ionic liquids to increase catalyst surface contact and reduce deactivation (Scheme 149) [552].

The catalyst was dissolved in 1-pentyl-3-methylimidazolium 3-(diphenylphosphaneyl)benzenesulfonate, [PnMIM][TPPMS] (**651**), to which a 1-octene (**650**) solution in the scCO_2_ was flushed through. The flow system allowed preparation of a 3:1 linear/branched (**345**/**652**) mixture of aldehyde with a space time yield of 0.2 mol L^−1^ d^−1^. However, low substrate flow rate could only be used as increasing the flow rate would result in overfilling of the reactor and leaching of the ionic liquid. The system performed at a lower efficiency than the apparatus described by the same group which used a higher pressure (200 bar vs 125 bar) [553]. Continuous-flow hydroformylation processes performed under supercritical conditions and employing heterogeneous catalysts have been comprehensively reviewed recently [554].

To recycle the secondary phase catalyst, supported ionic liquid phase (SILP) technology was also exploited. In 2019, Haumann et al. developed a membrane-based heterogenous catalyst made of a composite material of multiwalled carbon nanotubes modified with silica particles to control the porosity [555]. The composite supported a rhodium catalyst which avoided deactivation due to accumulation of high boiling point cross aldol-derived products. The continuous-flow system employed for the gas-phase hydroformylation of 1-butene gave high linear to branched selectivity (96%) and 64% selectivity towards the *n-*pentanal product. The apparatus allowed a large reduction in byproducts derived from secondary aldol condensation.

**Scheme 150:**
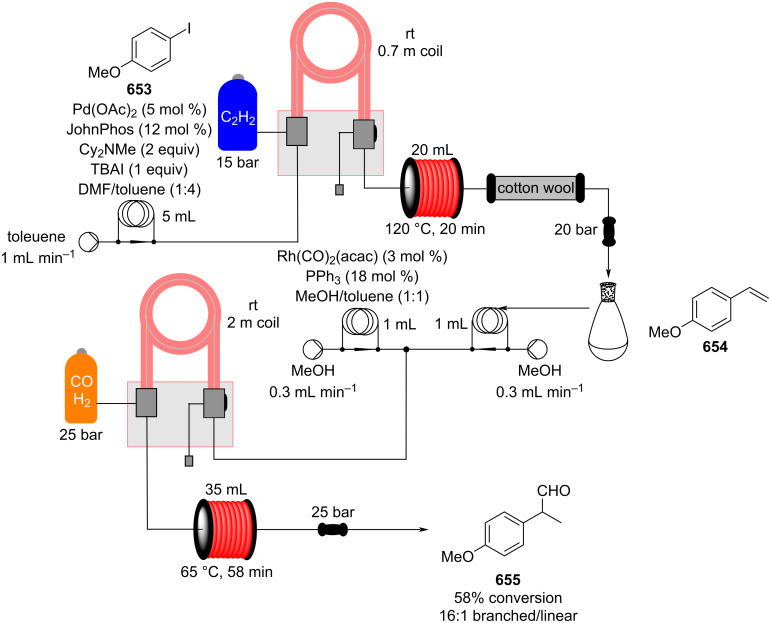
Two-step flow synthesis of aldehyde **655** through a sequential Heck reaction and subsequent hydroformylation.

In 2011, Ley and co-workers developed a flow reactor for the hydroformylation of styrenes based upon gas addition using a tube-in-tube reactor system [556]. The reactor was highly efficient (81–97% conversion) and flexible (12 examples). As a proof-of-concept, the synthesis was further extended to incorporate an initial Heck reaction between *p*-iodoanisole (**653**) and ethylene gas using JohnPhos and a second tube-in-tube reactor preparing styrene **654** which was progressed to aldehyde **655** (Scheme 150*)*. The first stage Heck reaction was found to generate significant amounts of palladium black, however, this was easily removed using a cartridge filled with cotton wool to act as a filter preventing downsteam aggrgation and blockage of the reactor.

Takahashi et al. assembled a simple syringe pump driven system attached to a stainless steel coil which was employed for the hydroformylation of 1-octene (**650**) [557]. The system only gave moderate ≈19% conversions, however, the authors suggested the unreacted alkene could be easily recycled. This simple unit does highlight how to safely handle hazardous reagents such as syngas in a labortary employing a flow reactor.

**Scheme 151:**
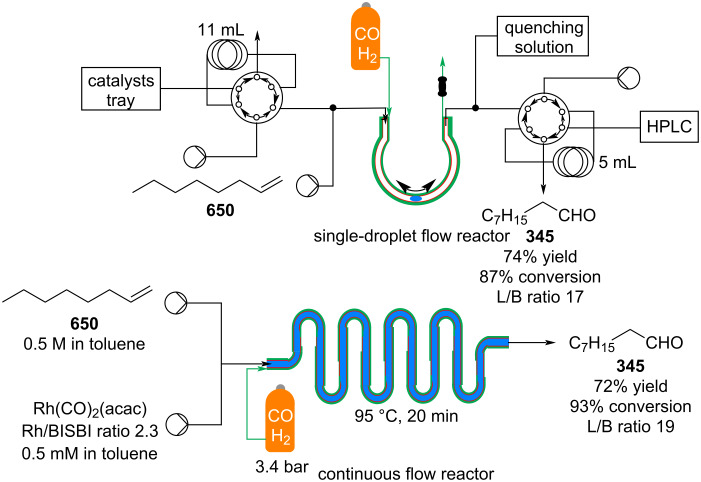
Single-droplet (above) and continuous (below) flow reactors developed by Abolhasani et al. for the process optimisation of hydroformylation reactions.

Studying the kinetics of a process is paramount to its optimisation. The Jensen and Abolhasani groups independently developed two microreactor systems which allow kinetic measurements alongside investigation of optimum reaction conditions. Jensen’s apparatus consisted of a 220 μL-silicon microreactor wherein the solution of catalyst and ligand was mixed with the syngas and 1-octene (**650**). The liquid outlet stream was then separated from the gas phase and analysed with GC and ATR-FTIR [558]. Abolhasani’s system was fully automated and allowed on-demand screening of several mixtures of catalysts and ligands as well as the reaction conditions [559]. The setup employed an horseshoe-shaped tube-in-tube reactor wherein the reaction mixture droplet oscillates reacting with the syngas coming from the outmost tube (Scheme 151).

The authors pointed out the possibility of performing experiments exploiting a wide range of reaction times (10 seconds to 1 day) without changing internal volume reactor or flow rates. The outlet was then mixed with a quenching solution and analysed via a HPLC system coupled with evaporative light scattering detector (ELSD) and MS. The optimal conditions yielded by this screening platform was then applied to the synthesis of nonal in high regioselectivity (linear/branched, L/B > 15) under low syngas pressures (3.4 bar, Scheme 151) [560].

**Scheme 152:**
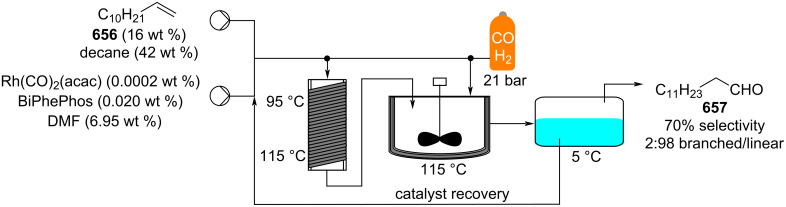
Continuous hydroformylation of 1-dodecene (**655**) using a PFR-CSTR system developed by Sundmacher et al.

Sundmacher and co-workers developed a miniplant setup for the continuous hydroformylation of 1-dodecene (**656**) in a thermomorphic multiphase system (Scheme 152) [561].

To recycle the catalyst, a *N,N-*dimethyformamide (DMF)/*n-*decane solvent system was employed which forms an homogenous phase at high temparatures around 95–115 °C and two separate phases when cooled down. The homogeneous reaction was initially measured and modelled on three different reactor systems: continuous stirred tanks reactor (CSTR), plug flow reactor (PFR) coupled with CSTR, and semibatch reactor (SBR) coupled with CSTR. Comparing all three cases, the last two allowed longer residence times and higher conversion, reducing the need of recycling the reagent. Furthermore, the PFR-CSTR system was experimentally constructed and configured [562]. A solution of 1-dodecene (**656**) in *n-*decane was mixed with the rhodium catalyst and the ligand (BiPhePhos) in DMF and then the biphasic system was streamed through the PFR. The PFR consisted of a helically coiled reactor with two different temperature zones (95–115 °C) which allowed for better reaction kinetic control than a single temperature. The first part was heated at 95 °C, which allowed the hydroformylation rate to be enhanced and the isomerisation rate to be reduced. The final section of the coil was warmed up to 115 °C and the outlet stream was then transfered into a CSTR (also heated at 115 °C) where the hydrogen content was higher. In this reactor, the fraction of isomer formed is allowed to be re-isomerised creating more reagent for hydroformylation The system allowed the tridecanal (98:2 linear/breanched) to form in 70% selectivity and full conversion, with a low rhodium leaching content of between 1.56 and 2.26 ppm.

Recently, Zhang et al. developed a nature-inspired microreactor which is capable of increasing the gas–liquid mixing [563]. The reactor consists of an honeycombed microchannel reactor in which the channel cappilaries and branches assist with breaking the gas slugs up, enhancing the contact area in the mixture. With such a flow apparatus, the hydroformylation of 1-hexene occurs at 90% yield after only 30 minutes residence time compared with the over 3 hours required in batch mode. In addition, the regioselectivty of the reaction (L/B) was also enhanced to 24.7 from 16.9 obtained in batch.

**Scheme 153:**
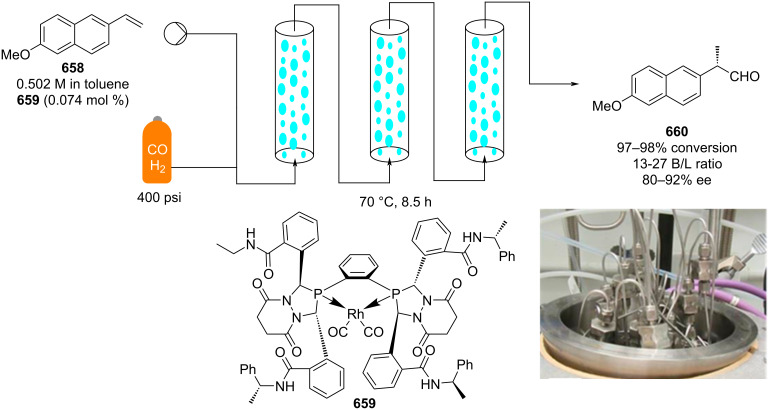
Continuous-flow synthesis of the aldehyde **660** developed by Eli Lilly & Co. [32]. Adapted with permission from [32]. Copyright 2016 American Chemical Society.

In furtherance of this research asymmetic hydroformylations (AHF) have also been investigated in flow. Enantioselecitivity and regioselectivity have been reported to be highly sensitive to changes in CO partial pressure [564]. For this reason, a better gas–liquid mixing as provided in flow would be expected to impact the transformation. In 2016, Landis et al. at Eli Lilly & Co. developed a reactor capable of extremely high mass transfer rates but coupled with long residence times. The reactor consists of a 17.8 mL stainless steel coiled reactor with a small internal diameter (i.d. 0.56 mm) where every 3.66 metres a 0.3 mL vertical pipe was placed [31]. With this setup, the vertical pipes would be principally filled up with liquid and the tubing mostly filled with vapour (90–93%) so that the residence times can be longer without influencing the amount of gas present along the coil reactor. Using this reactor setup, the AHF of 2-vinyl-6-methoxynaphthalene (**658**) was carried out employing the catalyst Rh(acac)(BDP) (**659**) at a loading of 0.074 mol % at high pressure (150–800 psi). A 0.502 M solution of substrate **658** along with the catalyst in toluene was mixed with the syngas flow and pumped through the stainless steel reactor heated up to 70 °C (Scheme 153). The flow system ran uninterrupted for over 130 hours yielding **660** in high conversion (97–98%), high regioselectivity (branched/linear = 13–27), and enantioselectivity (ee = 80–92%) [32].

**Scheme 154:**
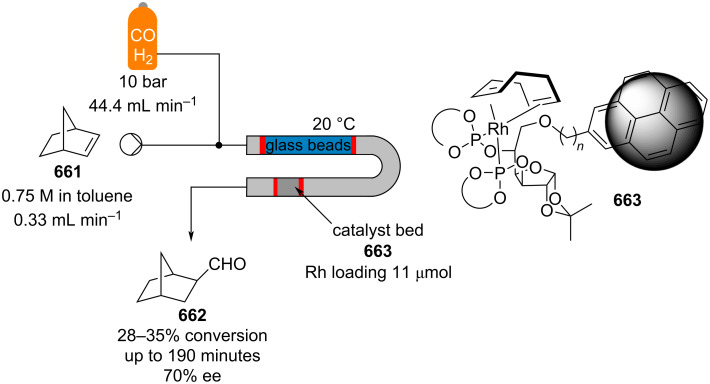
Continuous asymmetric hydroformylation employing heterogenous catalst supported on carbon-based supports.

In 2019, a supported catalyst for AHF was developed by Garcia-Suarez, Godard and co-workers [565]. The employed ligands consists of 1,3-diphosphites with furanose backbone carrying a pyrene functional group capable of π–π stacking interactions with hydrophobic supports. The catalyst was then immobilised on several carbonic supports such as MWNT, reduced graphene oxide (rGO), and carbon beads (CBs). After immobilisation, the catalysts maintained their activity. The heterogeneous catalysts were then investigated in continuous-flow mode using a U-shaped tube which was partially filled with cotton wool to enhance the initial gas–liquid mixing, and then partially filled with the catalyst bed (Scheme 154). The flow apparatus increases the enantioselectivity of the reaction (**661** → **662**), however, substantial metal leaching and activity loss was observed using MWCN, and rGO. Increasing the catalyst loading in the fixed bed and employing the CB-supported catalyst, the authors managed to setup a stable flow system allowing conversion up to 35% over 190 minutes of runtime with an ee of 70%.

#### Acetylation

**Scheme 155:**
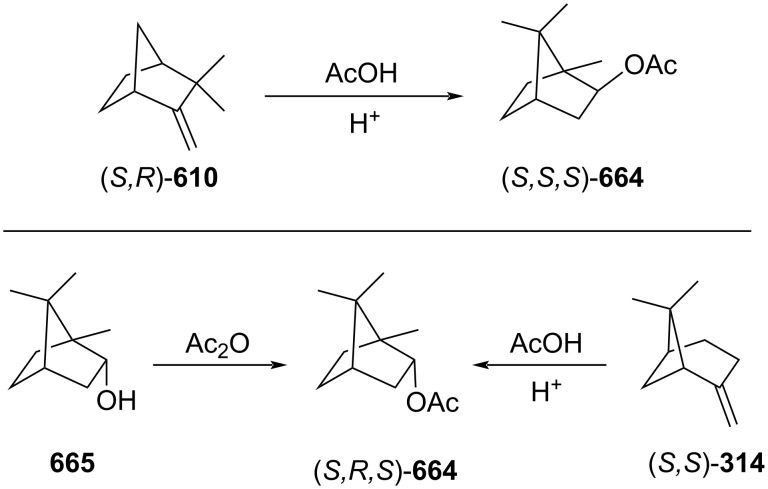
Examples of acetylation in F&F industry: synthesis of bornyl (*S,R,S-***664**) and isobornyl (*S,S,S-***664**) acetate.

Acyl functionalities are often encountered in both fragrance products and their intermediates. Indeed, acetylation is a widely used transformation throughout of all synthetic chemistry; used as a protecting group, for activating an alcohol or as small molecular weight esters. As examples of acetylation in F&F industry, bornyl (*S,R,S-***664**) and isobornyl (*S,S,S-***664**) acetate are prepared via acetylation of their correspondent terpene derivatives such as borneol (**665**), pinene (*S,S-***314**), and camphene (*S,R-***610**, Scheme 155) [566].

**Scheme 156:**
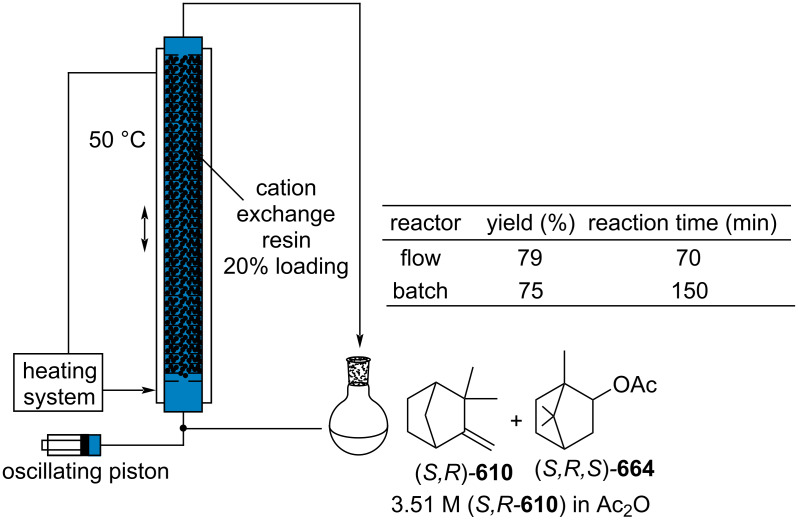
Continuous-flow preparation of bornyl acetate (*S,R,S-***664**) employing the oscillating flow reactor.

These above processes are acid-catalysed reactions that go via carbocation intermediates. Therefore, the control of the conditions and the nature of the catalyst are essential to the resulting product composition. A continuous-flow approach for the synthesis of compound *S,R,S-***664** has been reported using an oscillatory flow reactor and a heterogeneous catalyst (an acid cation exchange resin). The homogenous heat control and excellent dispersity of the catalyst in the reactor resulted in a higher selectivity and overall faster reaction compared to its corresponding batch mode (Scheme 156) [567].

**Scheme 157:**
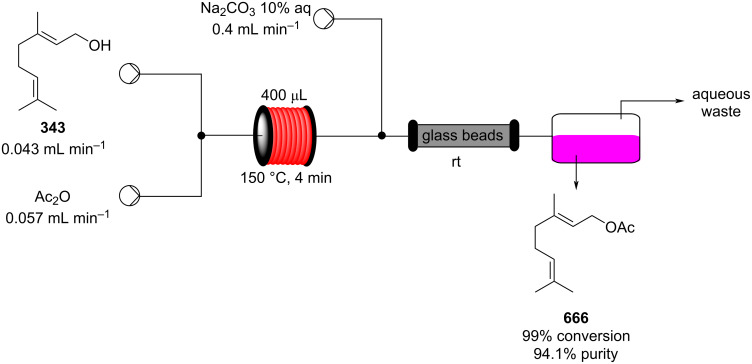
Continuous-flow synthesis of geranyl acetate (**666**) from acetylation of geraniol (**343**) developed by Adarme et al.

Another example of F&F chemical preparation is the acetylation of geraniol (**343**) through acetic anhydride developed by Adarme and co-workers in 2018 (Scheme 157) [568]*.*

The reagent streams were combined together neat and passed through a heated coil reactor at 150 °C. After 4 minutes, the reaction mixture was quenched in-line with a 10% solution of Na_2_CO_3_, thoroughly mixed using a glass beads-filled reactor, and then the two phases were continuously separated. The process lead to the final material **666** in 94.1% of purity (99% conversion). The authors also developed a biocatalysed transesterification using ethyl acetate as the acetylating agent to yield the same material in high conversion (99% conversion).

Only a handful of reports exist that deal exclusively with acetylation in flow. In 2015 a polymer grafted dimethylaminopyridine (*g*-DMAP) catalyst was used to promote a continuous acetylation as disclosed by Takeda and co-workers [569]. The immobilised reagent was developed as an alternative to DMAP supported on polystyrene (2% cross-linked with divinylbenzene) and was shown to give superior yields both in batch and flow. The starting material 1-phenyl-1-hexanol (**427**) was used as a model substrate, for which, it was found that the use of a 1.5 mm i.d. packed-bed reactor gave the best results. The packed column reactor was found to be highly robust, giving consistent conversions over the course of 50 hours. The reactors use in parallel for scale-up was also investigated (Table 14). Good scalability was demonstrated with the authors using up to 8 parallel reactors simultaneously.

**Table 14:** Conversion values for individual reactors when used in parallel for continuous g-DMAP catalysed acetylation scale-up.

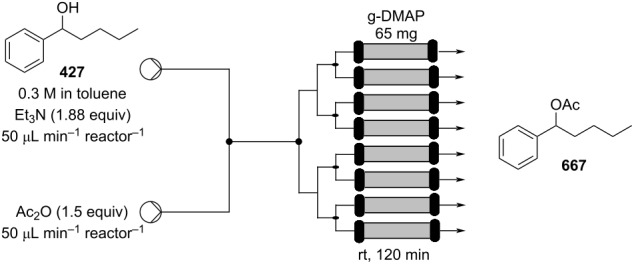									

Entry	Number of parallel reactors	Conversion (%)^a^ reactor number							
		1	2	3	4	5	6	7	8

1	2	99	98	–	–	–	–	–	–
2	3	94	99	97	95	–	–	–	–
3	4	98	98	102	102	97	100	98	99

^a^Determined by HPLC.

**Scheme 158:**
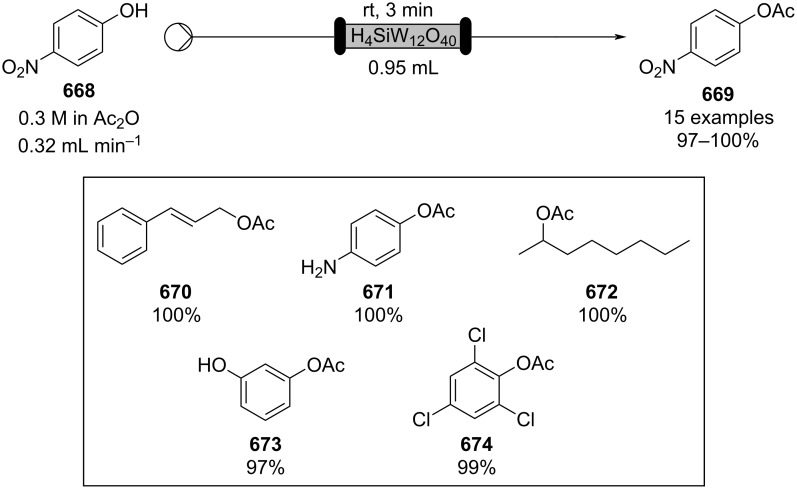
12-Ttungstosilicic acid-supported silica monolith-catalysed acetylation in flow.

In 2012 the use of a 12-tungstosilicic acid-supported silica monolith (H_4_SiW_12_O_40_-monolith) for the acetylation of a range of substrates in flow was reported by Haswell et al. (Scheme 158) [570].

The reactors were prepared in two sizes by immobilising the heteropolyacid (HPA) onto the monolith by adsorption and calcination. They were then clad in a heat-shrinkable Teflon^®^ tube equipped with glass connectors at each end and heated to 330 °C. The resultant columns were used for the acetylation of various aromatic and aliphatic alcohols at room temperature with very high yields being obtained in all cases.

**Scheme 159:**
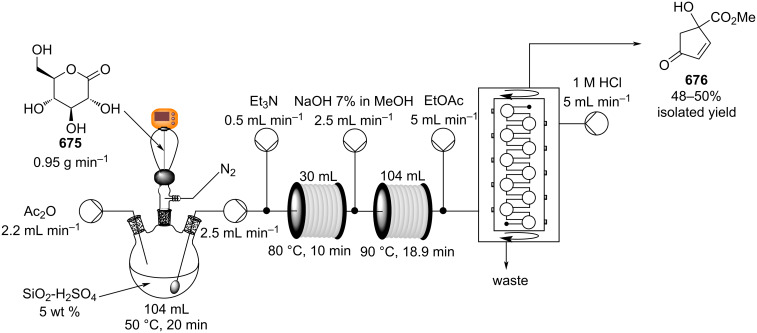
Continuous-flow preparation of cyclopentenone **676**.

Another example of solid-supported catalyst is the acetylation of ᴅ-glucono-1,5-lactone (**675**) for the telescoped preparation of cyclopentenone **676** [571]. A CSTR system was developed where acetic anhydride and **675** were slowly added to a silica-supported sulfuric acid catalyst suspended in an stirred flask. The calculated residence time was around 20 minutes and the penta-acetylated material was obtained in quantitative yield with a productivity of roughly 200 g h^−1^. When evaluated in batch mode, the authors noticed the reaction requires high amounts of Ac_2_O (4.5 equiv) and a precise control of the temperature to ensure high and clean conversion. Due to a low solublity of the lactone **675** in the acetylating agent, the continuous apparatus could only employ a CSTR, where the starting material solubilises during reaction. In the CSTR only 3.6 equivalents of Ac_2_O were employed enabling telescoping into a subsequent transformation. As such, in Scheme 159, a base-catalysed elimination followed by a methanolysis reaction were sequenced.

The final product **676** was then purified with a countercurrent extraction, employing a AM technologies ACR device allowing isolation of **676** in 48–50% overall yield. The authors also described a more efficient alternative procedure which employed an ion exchange resin for the last step. In this case, the cyclopentenone **676** was isolated in 71% yield with a producvtivity of 112 g over a 30 h runtime.

**Scheme 160:**
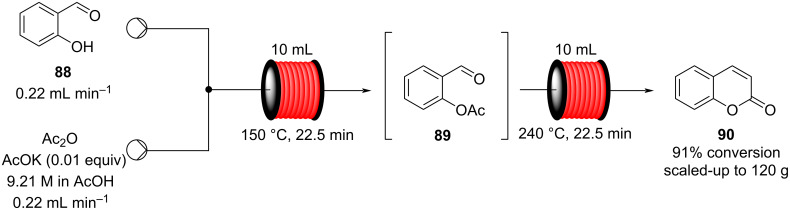
Two-stage synthesis of coumarin (**90**) via acetylation of salicylaldehyde (**88**).

The acetylation of salicylaldehyde (**88**) in a continuous two-stage synthesis of coumarin (**90**) has also described [572]. The first step was performed mixing a solution of **88** with a stream containing acetic anhydride and potassium acetate in acetic acid as the solvent. These were directed into a heated coil reactor maintained at 150 °C. Complete conversion to the acetylated compound was reported, which converts to coumarin (**90**) after heating at 240 °C. This process resulted in much shorter reaction times compared to working in batch and the apparatus was used to scale-up and allow collection of 120 g of material (Scheme 160).

**Scheme 161:**
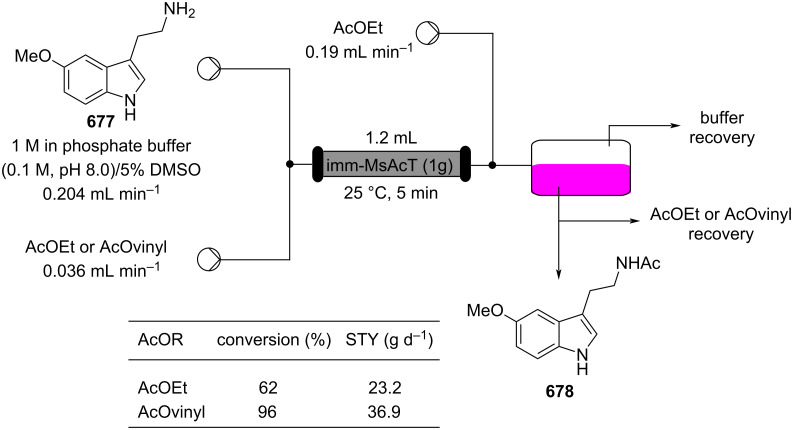
Intensification process for acetylation of 5-methoxytryptamine (**677**) to melatonin (**678**) developed by Paradisi, Tamborini, and co-workers.

Other examples of acetylation in flow include the acylation of racemic alcohols by lipases in continuous-flow bioreactors [573] and the high temperature transesterification of glycerol derivatives with alkyl and vinyl acetates [574]. In 2019, intensification process for the synthesis of melatonin was achieved employing solid-supported enzymes (Scheme 161) [575].

The reaction consists of a transesterification using ethyl acetate by MsAcT, which is an acetotransferase known for its remarkable activity. The authors investigated several hydrophilic carriers and found out that glyoxyl agarose was the most suitable. Once packed in a column reactor, a 1 M solution of tryptamine **677** in 85:15 pH 8 buffer/AcOEt was streamed though at 25 °C. The heterogeneous mixture was then separated using a Zaiput liquid/liquid separator to yield melatonin in 62% conversions. Higher isolated yields (96%) were obtained when vinyl acetate was used as an acetyl donor. The flow system allowed the authors to prepare roughly 36.9 g d^−1^ of the desired material **678**. While there are numerous reports that feature acetylation as part of a multistep flow sequence [67,125,426,576], there is still much work to be done.

#### Metathesis

Discovered in the early 1950s, olefin metathesis (OM) is a ground-breaking tool for organic synthesis, as highlighted by the award of the 2005 Nobel prize in chemistry jointly to Yves Chauvin, Robert H. Grubbs and Richard R. Schrock "for the development of the metathesis method in organic synthesis". Since the development and commercialisation of the first generation Grubbs catalyst, OM has found many applications [577] and it has now been industrially applied for the preparation of petrochemicals [578], polymers [579], and drugs [580–581]. Often disregarded by the F&F industry due to issues with patent coverage and freedom to operate, there is now with many of the original patents expiring, a renewed interest, particularly for large ring formations. Ring-closing metathesis (RCM) allows for the easily formation of medium-large size rings, which would be less practicable using alternative strategies. Several catalysts have been developed for this task, achieving high activities (with TONs up to 315,000) and selectivities [582–585].

**Scheme 162:**
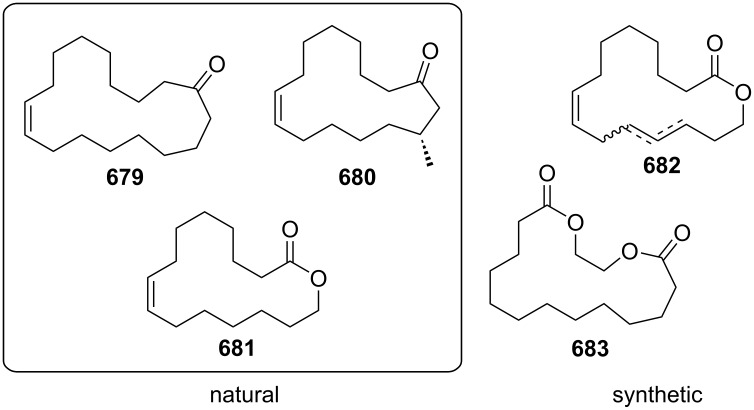
Examples of macrocyclic musky odorants both natural (**679**–**681**) and synthetic (**682** and **683**).

Beyond a few exceptional examples on small rings (damascone and galbanone derivatives) [586–587], the RCM has found more application in the synthesis of macrocyclic musky odorants. Starting from the natural civettone (**679**), muscone (**680**), and omega-pentadecalactone (**681**), numerous medium size macrocycles have been developed and are now commercially available (Scheme 162).

At the end of the twentieth century, only a few reported syntheses employing OM were known [588–590], often due to issues with selectivity and poor activity of the catalysts. Later, with progress in the catalyst design (especially stabilising ligand framework), various multistep preparations of macrocycles were being described [591–599]. However, common to all these examples is that ring-closing metathesis requires high dilution to avoid dimerization and as such it also needs long reaction times, a problematic issue when considering industrially application. Consequently, enabling technologies such as flow chemistry have been employed to aid with process intensification.

**Scheme 163:**
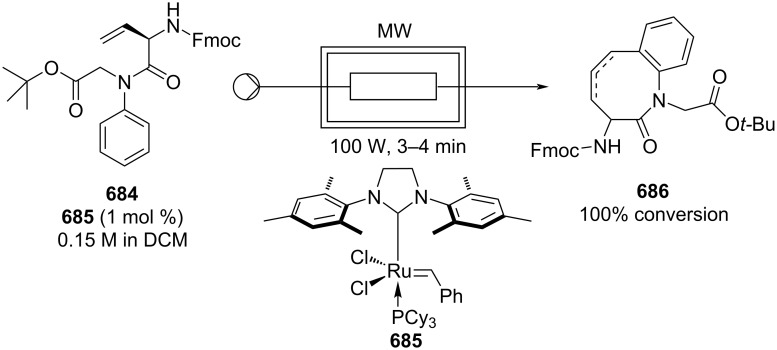
Flow setup combined with microwave for the synthesis of macrocycle **686** via RCM.

In 2005, Organ and Comer employed a flow OM setup combined with microwave irradiation to boost reaction times from 30 minutes to as little as 3 minutes yielding the products in full conversion (Scheme 163) [600]. Similar improvements were obtained when RCM was employed for the formation of smaller rings [601].

**Scheme 164:**
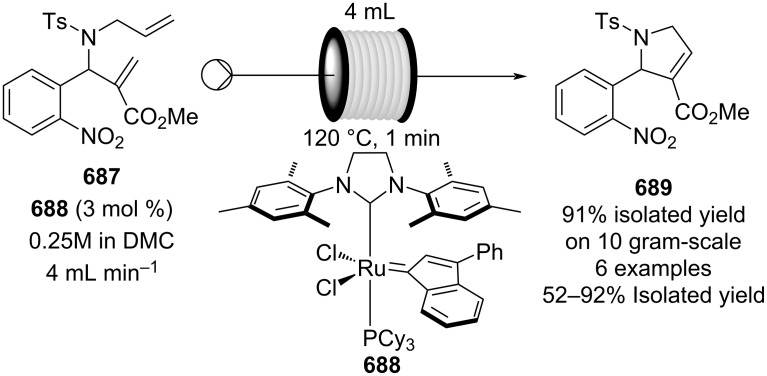
Continuous synthesis of 2,5-dihydro-1*H*-pyrroles via ring-closing metathesis.

Lamaty et al. described a continuous-flow setup where a reaction in dimethyl carbonate (DMC) was passed into the coil at 110–120 °C for 1–5 minutes and 2,5-dihydro-1*H*-pyrroles were isolated in moderate to high yields (Scheme 164) [602]. The system allowed the larger scale processing of **687** (10 g) and after 37 minutes the product **689** was isolated in 91% yield, with comparable result as obtained on a 1 mmol scale in batch.

**Scheme 165:**
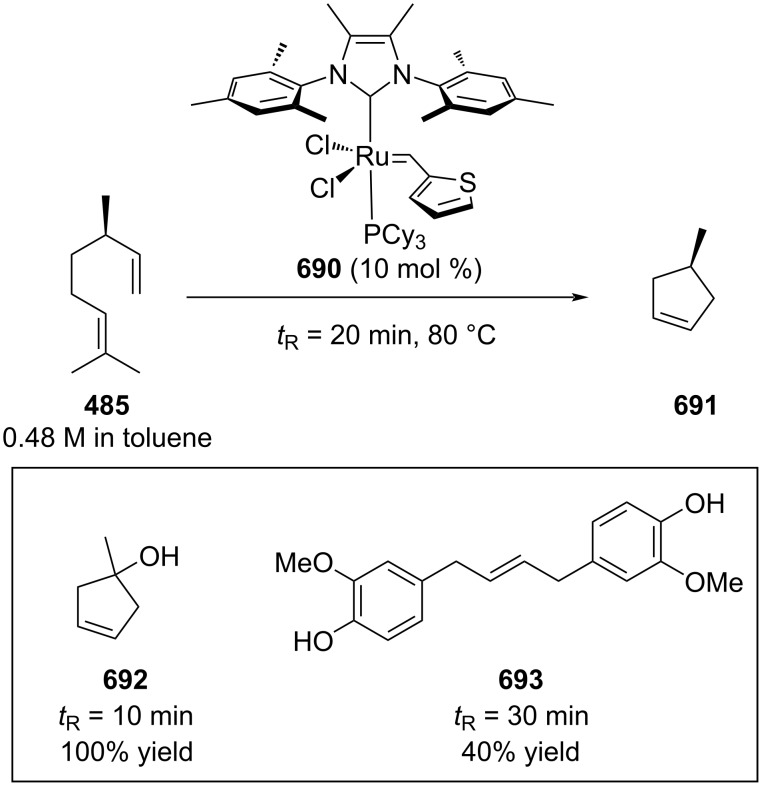
Continuous-flow metathesis of **485** developed by Leadbeater et al.

Leadbeater et al. applied a similar system to several other feedstocks such as linalool, citronellene (**485**), and eugenol which gave moderate to high conversions (40–100%) to their products **691**–**693** in residence times of 10–30 minutes (Scheme 165) [603].

**Figure 12:**
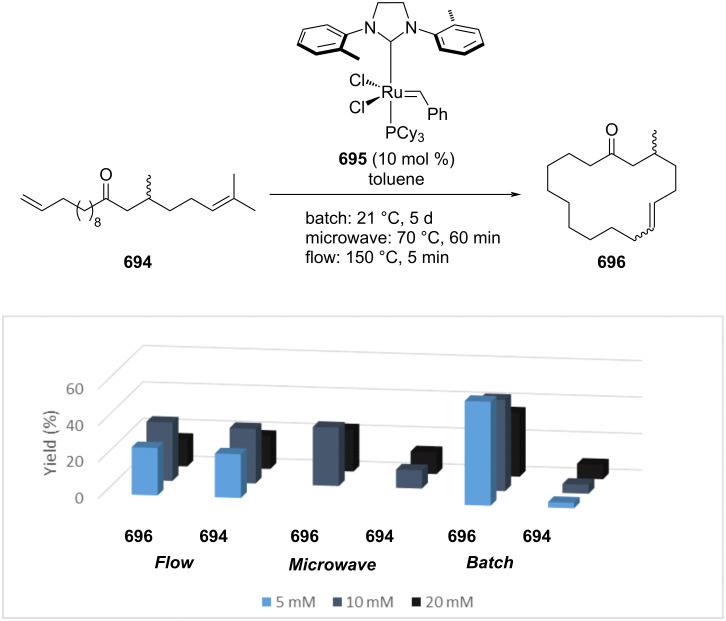
Comparison between RCM performed using different routes for the preparation of **696**. On the left the conversion of **696** and the percentage of unreacted material **694** on the right.

Recently, ring-closing metathesis has been used in the synthesis of 3-methylcyclohexandec-6-enone (**696**), a Firmenich musky odorant product, which was performed in a continuous-flow mode (Figure 12). The results were then compared to the reaction obtained in batch under conventional thermal and with microwave heating [604].

Although showing lower yields (32% vs 57%), the flow and microwave reactions allowed the reaction to be conducted at a higher concentration (10 mM vs 5 mM) and gave much shorter reaction times (5–60 minutes vs 5 days). Additionally, the flow protocol was easily scaled up, yielding 1 g of product **696** in less than 5 hours. The high percentage of unreacted starting material suggests a more selective reaction and presents the possibility to recover unreacted starting material. The methodology was also further exploited in the synthesis of different 16-membered analogues and resulted in highly reproducible processes.

A general issue in many catalysis processes is the need for careful final purification of the material to remove residual metal and ligands, which is not always trivial. This is especially true as many examples still required high loading of the catalyst and this also raises the issue of its necessary recovery due to the high metal costs. One possible option is to move to heterogeneous catalysis, but this still presents issues in terms of activity and recyclability.

**Scheme 166:**
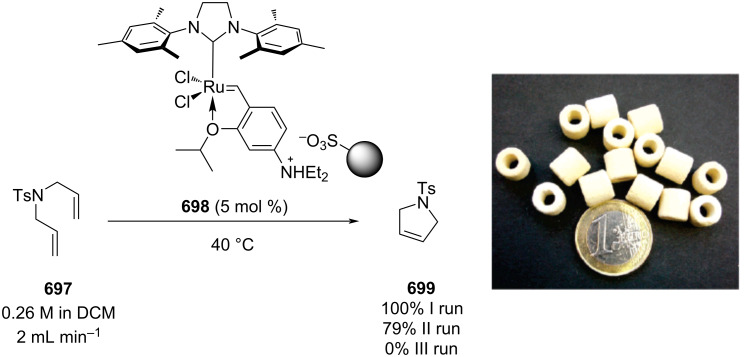
Continuous-flow RCM of **697** employed the solid-supported catalyst **698** developed by Grela, Kirschning et al. [605] Picture showing the glass Raschig rings compared to a 1 € coin’s dimension. Adapted with permission from [605]. Copyright 2006 American Chemical Society.

As an excellent example of heterogeneous catalysis, Kirschning, Grela, and co-workers performed ruthenium immobilisation on a sulfonic acid resin by means of ionic interactions [605]. An amine-functionalised ligand was flushed through a sulfonic acid functionalised composite material shaped to fit in a stainless steel column (Scheme 166).

The ruthenium catalyst **698** was then installed via cross-metathesis of Grubbs II catalyst with the solid support. Despite the advantages in employing such immobilisation (fast support recovery and catalyst regeneration on large scales), the authors described a rapid drop in catalytic activity after 2 runs (100% first run, 79% second run, 0% third run) when applied to the RCM of **697**.

**Scheme 167:**
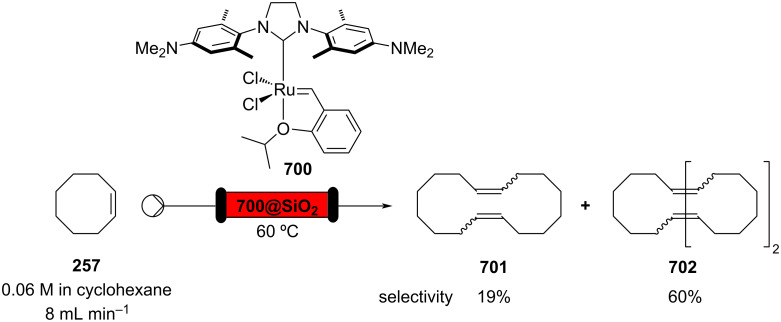
Continuous-flow RORCM of cyclooctene employing the silica-absorbed catalyst **700**.

In 2012, Limbach et al. described a continuous-flow system equipped with a catalyst absorbed on silica gel [606]. The catalyst presents amino groups on the *N-*heterocyclic carbene moieties which enables ionic interactions between the catalyst and the silanol groups of the silica gel. The most active catalyst **700** (TON = 4350) was used for ring-opening/ring-closing metathesis (RORCM) of cyclooctene (**257**), however, it achieved low selectivity for the desired dimer **701** (only 19%, Scheme 167). The system was also exploited for the self-metathesis of methyl oleate (**703**), where turnover numbers of 4950 was used, but unfortunately, coupled with moderate Rh leaching (19%).

Over the years, other immobilization concepts have been investigated. The immobilization strategy was performed through either impregnation–absorption [607] or binding of the metal to a ligand on the support [605,608–613]. Several supports were evaluated such as silica gel [606,608,613], mesoporous silica [607,609–610], polymers [612], and oligomers [611]. A rhenium catalyst (Re_2_O_7_) has also been reported in flow for the cross-metathesis of 1-octene (**650**) using scCO_2_ as the solvent to allow a better mass transport (higher pressure) and catalyst recovery [614].

**Scheme 168:**
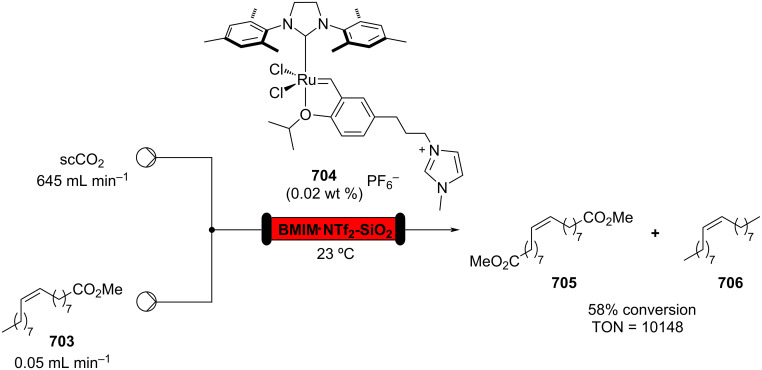
Continuous-flow self-metathesis of methyl oleate (**703**) employing SILP catalyst **704**.

Nolan et al. also employed an elegant catalyst immobilisation strategy involving impregnation of the supported ionic liquid phase (SILP) catalyst on silica [615]. The resulting silica-supported catalyst **704** was place into a 9 mL column reactor and the substrate were flushed through the system using scCO_2_ as the solvent (Scheme 168).

The system was found highly efficient for the self-metathesis of methyl oleate (**703**), reaching catalyst turnover numbers >10000, with low metal leaching (8 ppm). Cross-metathesis between substrate **703** and dimethyl maleate was also found to work well, however, with lower activity and therefore longer reaction time required. The system was however found unsuitable for metathesis of terminal alkenes.

**Scheme 169:**
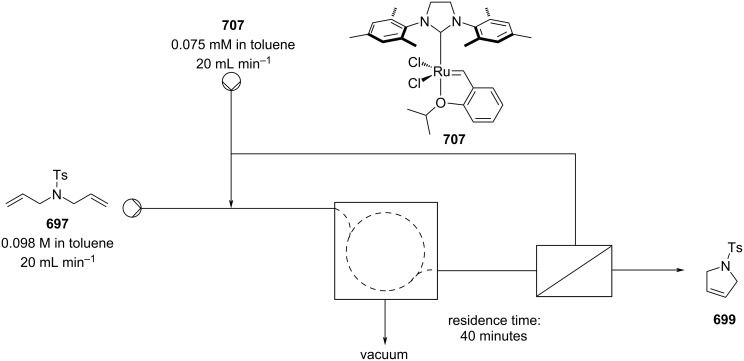
Flow apparatus for the RCM of **697** using a nanofiltration membrane for the recovery and reuse of the catalyst **707***.*

More recently an alternative approach using an organic solvent nanofiltration (OSN) membrane for the RCM reaction has been reported [616–618]. The system uses a homogeneous catalyst but simplifies the catalyst removal by nanofiltration making it more industrially applicable. As an illustration Jensen et al. used an organic solvent nanofiltration (OSN) membrane to facilitate recovery of catalyst **707** enabling easy recycling (Scheme 169) [619].

In the reaction a co-feed of catalyst **707** (2 mg) and substrate (ratio 1:1300) enabled 50 hours of continuous operation with a constant output of 94% of the desired product **699**. Moreover, the metal content in the final material was less than 1 ppm. The authors highlighted the apparatus could be further optimised to maximize the catalyst TON, which was measured to be 950.

Unfortunately, standard commercially available catalysts such as Hoveyda–Grubbs and Umicore M series [618], would be of too low molecular weight to allow their separation by current molecular membranes. For these reasons many modified higher molecular weight catalysts have been prepared and applied in continuous systems for a better catalyst recovery [616–617].

**Scheme 170:**
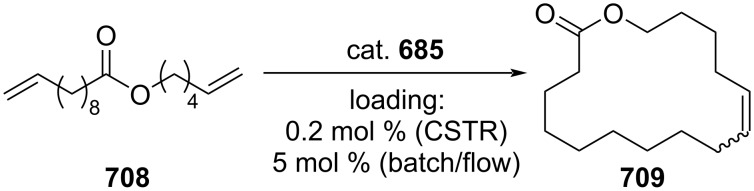
Comparison of loadings between RCMs performed with different routes for the synthesis of **709**.

Despite the extensive work on catalyst design and activity improvements, low catalyst activity and poor selectivity for certain substrates is still a big issue in the wider adoption of this reaction within industrial settings. As claimed by Fogg et al., the ethane produced by the reaction could stay entrapped in the system, causing catalyst’s decomposition. Using catalyst **685**, they obtained slightly better results using a continuous stirred-tank reactor (CSTR) than a tubular flow reactor, confirming their hypothesis (Scheme 170) [620]. This is interesting and may offer further usage of reactor systems like the tube-in-tube reactor setup for the removal of volatiles such as ethane in flow processes to improve activity or change product compositions.

Indeed, this approach has been investigated by Jensen and O’Neal [619] and Skowerski et al. [621] who adopted a membrane reactor using Teflon AF2400 to allow the ethane removal by pervaporation (Scheme 169).

## Conclusion and Outlook

Liquid fragrance ingredients and precursors are ideal candidates for continuous manufacturing. The majority of the examples in this review concerned smaller/research scale synthesis but in many cases scalability was also robust achieved. Other reaction types such as the Prins reaction and decarboxylation were not touched upon due to a lack of literature precedence. These should be acknowledged looking forward as should other problems associated with flow chemistry. There are currently limitations and weaknesses with regards to the integration of flow chemistry into manufacturing that need to be addressed. Much of the technology developed alongside flow equipment carries with it implications for various stages of production (camera integration [622], the use of feed-back algorithms [623] and in-line analytics techniques already mentioned), however, more research is needed to bring together flow chemistry and industrially relevant purification techniques such as distillation. Industry has generally been slow to adopt flow technology due to the “like what you know, know what you like” sentiment and the initial costs of investing in the necessary equipment to replace well established and well optimised processes. Despite this, the pharmaceutical industry is beginning to take advantage of the benefits offered by continuous manufacturing [624–626]. Undoubtedly, there are processes currently being used within the flavour and fragrance industry for which flow would offer an attractive alternative with regards to both cost and green/environmental implications. These are very early days for flow within the fragrance industry, however, and much more work is needed to fully assess the feasibility of coupling the areas of flow chemistry and fragrance production.

## References

[1] (2021). Flavors and Fragrances Market Size, Share & Industry Analysis, By Type (Flavors {Natural and Synthetic} and Fragrances {Natural and Synthetic}) By Application (Food & Beverage, Cosmetics & Personal Care, Pharmaceutical, Home & Floor Care, Fine Fragrances.

[2] Sell C S (2006). Appendix I: Some of the More Important Natural Fragrance Materials. The Chemistry of Fragrances: From Perfumer to Consumer.

[3] Fukuyama T, Rahman M, Sato M, Ryu I (2008). Synlett.

[4] Baxendale I R, Brocken L, Mallia C J (2013). Green Process Synth.

[5] Ley S V, Baxendale I R (2002). Nat Rev Drug Discovery.

[6] Baxendale I R (2013). J Chem Technol Biotechnol.

[7] Jas G, Kirschning A (2003). Chem – Eur J.

[8] Sell C S (2006). The Chemistry of Fragrances: From Perfumer to Consumer.

[9] Bauer K, Garbe D, Surburg H (2001). Common Fragrance and Flavor Materials Preparation, Properties and Uses.

[10] (2020). About the IFRA Transparency List.

[11] Salvito D, Lapczynski A, Sachse-Vasquez C, McIntosh C, Calow P, Greim H, Escher B (2011). Ecotoxicol Environ Saf.

[12] Ohloff G, Pickenhagen W, Kraft P (2011). Scent and Chemistry. The Molecular World of Odors.

[13] Zarzo M, Stanton D T (2009). Atten Percept Psychophys.

[14] Webb D, Jamison T F (2010). Chem Sci.

[15] Wegner J, Ceylan S, Kirschning A (2011). Chem Commun.

[16] Rossetti I, Compagnoni M (2016). Chem Eng J.

[17] Brzozowski M, O’Brien M, Ley S V, Polyzos A (2015). Acc Chem Res.

[18] Ramanjaneyulu B T, Vishwakarma N K, Vidyacharan S, Adiyala P R, Kim D-P (2018). Bull Korean Chem Soc.

[19] Trojanowicz M (2020). Molecules.

[20] Guidi M, Seeberger P H, Gilmore K (2020). Chem Soc Rev.

[21] Britton J, Jamison T F (2017). Nat Protoc.

[22] Darvas F, Dorman G, Hessel V (2014). Flow Chemistry – Applications.

[23] Vaccaro L (2017). Sustainable Flow Chemistry: Methods and Applications.

[24] Jamison T, Koch G (2018). Flow Chemistry in Organic Synthesis.

[25] Luis S V, Garcia-Verdugo E (2019). Flow Chemistry: Integrated Approaches for Practical Applications.

[26] Len C, Luisi R (2020). Catalytic Methods in Flow Chemistry.

[27] Noël T, Luque R (2020). Accounts on Sustainable Flow Chemistry.

[28] Kitching M O, Dixon O E, Baumann M, Baxendale I R (2017). Eur J Org Chem.

[29] Gérardy R, Emmanuel N, Toupy T, Kassin V-E, Tshibalonza N N, Schmitz M, Monbaliu J-C M (2018). Eur J Org Chem.

[30] Baumann M, Baxendale I R (2015). Beilstein J Org Chem.

[31] Johnson M D, May S A, Calvin J R, Lambertus G R, Kokitkar P B, Landis C R, Jones B R, Abrams M L, Stout J R (2016). Org Process Res Dev.

[32] Abrams M L, Buser J Y, Calvin J R, Johnson M D, Jones B R, Lambertus G, Landis C R, Martinelli J R, May S A, McFarland A D (2016). Org Process Res Dev.

[33] Kappe C O (2002). Curr Opin Chem Biol.

[34] Watts P (2005). QSAR Comb Sci.

[35] Wiles C, Watts P, Haswell S J (2007). Lab Chip.

[36] Pastre J C, Browne D L, Ley S V (2013). Chem Soc Rev.

[37] Baxendale I, Ley S (2005). Curr Org Chem.

[38] Akwi F M, Watts P (2018). Chem Commun.

[39] Baumann M, Moody T S, Smyth M, Wharry S (2020). Org Process Res Dev.

[40] Anastas P, Warner J (1998). Green Chemistry: Theory and Practice.

[41] Wiles C, Watts P (2012). Green Chem.

[42] Anastas P T, Zimmerman J B (2003). Environ Sci Technol.

[43] Sheldon R A (2007). Green Chem.

[44] Browne D L, Deadman B J, Ashe R, Baxendale I R, Ley S V (2011). Org Process Res Dev.

[45] Deadman B J, Browne D L, Baxendale I R, Ley S V (2015). Chem Eng Technol.

[46] Sharma M K, Suru A, Joshi A, Kulkarni A A (2020). Ind Eng Chem Res.

[47] Bianchi P, Williams J D, Kappe C O (2020). J Flow Chem.

[48] Doyle B J, Gutmann B, Bittel M, Hubler T, Macchi A, Roberge D M (2020). Ind Eng Chem Res.

[49] Ley S V, Baxendale I R, Bream R N, Jackson P S, Leach A G, Longbottom D A, Nesi M, Scott J S, Storer R I, Taylor S J (2000). J Chem Soc, Perkin Trans 1.

[50] Sussman S (1946). Ind Eng Chem, Ind Ed.

[51] Booth R J, Hodges J C (1999). Acc Chem Res.

[52] Adamo A, Heider P L, Weeranoppanant N, Jensen K F (2013). Ind Eng Chem Res.

[53] Kralj J G, Sahoo H R, Jensen K F (2007). Lab Chip.

[54] Sahoo H R, Kralj J G, Jensen K F (2007). Angew Chem.

[55] Hornung C H, Mackley M R, Baxendale I R, Ley S V (2007). Org Process Res Dev.

[56] Mason B P, Price K E, Steinbacher J L, Bogdan A R, McQuade D T (2007). Chem Rev.

[57] Lee C-Y, Chang C-L, Wang Y-N, Fu L-M (2011). Int J Mol Sci.

[58] Carter C F, Lange H, Ley S V, Baxendale I R, Wittkamp B, Goode J G, Gaunt N L (2010). Org Process Res Dev.

[59] Perro A, Lebourdon G, Henry S, Lecomte S, Servant L, Marre S (2016). React Chem Eng.

[60] Poh J-S, Browne D L, Ley S V (2016). React Chem Eng.

[61] Glotz G, Lebl R, Dallinger D, Kappe C O (2017). Angew Chem, Int Ed.

[62] Segal-Rosenheimer M, Dubowski Y (2007). J Phys Chem C.

[63] Giraudeau P, Felpin F-X (2018). React Chem Eng.

[64] Thomson C G, Jones C M S, Rosair G, Ellis D, Marques-Hueso J, Lee A-L, Vilela F (2020). J Flow Chem.

[65] Sagmeister P, Williams J D, Hone C A, Kappe C O (2019). React Chem Eng.

[66] Archambault C M, Leadbeater N E (2016). RSC Adv.

[67] Mancino V, Cerra B, Piccinno A, Gioiello A (2018). Org Process Res Dev.

[68] Hopkin M D, Baxendale I R, Ley S V (2010). Chem Commun.

[69] Krishnadasan S, Brown R J C, deMello A J, deMello J C (2007). Lab Chip.

[70] Trunina D, Liauw M A (2020). Chem Ing Tech.

[71] Loren B P, Wleklinski M, Koswara A, Yammine K, Hu Y, Nagy Z K, Thompson D H, Cooks R G (2017). Chem Sci.

[72] Browne D L, Wright S, Deadman B J, Dunnage S, Baxendale I R, Turner R M, Ley S V (2012). Rapid Commun Mass Spectrom.

[73] Benz C, Boomhoff M, Appun J, Schneider C, Belder D (2015). Angew Chem, Int Ed.

[74] Schwolow S, Braun F, Rädle M, Kockmann N, Röder T (2015). Org Process Res Dev.

[75] Mozharov S, Nordon A, Littlejohn D, Wiles C, Watts P, Dallin P, Girkin J M (2011). J Am Chem Soc.

[76] Hamlin T A, Leadbeater N E (2013). Beilstein J Org Chem.

[77] Pelletier M J, Fabiilli M L, Moon B (2007). Appl Spectrosc.

[78] Baxendale I R, Ley S V, Seeberger P H, Blume T (2007). Solid Supported Reagents in Multi-Step Flow Synthesis. New Avenues to Efficient Chemical Synthesis.

[79] Baumann M, Baxendale I R, Ley S V, Smith C D, Tranmer G K (2006). Org Lett.

[80] Dräger G, Kiss C, Kunz U, Kirschning A (2007). Org Biomol Chem.

[81] Baxendale I R, Ley S V, Smith C D, Tranmer G K (2006). Chem Commun.

[82] Smith C D, Baxendale I R, Tranmer G K, Baumann M, Smith S C, Lewthwaite R A, Ley S V (2007). Org Biomol Chem.

[83] Smith C D, Baxendale I R, Lanners S, Hayward J J, Smith S C, Ley S V (2007). Org Biomol Chem.

[84] Baumann M, Baxendale I R, Ley S V, Nikbin N, Smith C D (2008). Org Biomol Chem.

[85] Baumann M, Baxendale I R, Ley S V (2008). Synlett.

[86] Mallia C J, Baxendale I R (2016). Org Process Res Dev.

[87] Wiles C, Watts P, Haswell S J, Pombo-Villar E (2001). Lab Chip.

[88] Kawaguchi T, Miyata H, Ataka K, Mae K, Yoshida J-i (2005). Angew Chem, Int Ed.

[89] Wiles C, Watts P, Haswell S J, Pombo-Villar E (2004). Lab Chip.

[90] Hua J, Liu X, Xu W, An L, Zhang J, Wang K, Wan M, Zhang F (2013). Production Process of Anisyl Propionaldehyde. Chin. Patent.

[91] Knorr A, Weissenborn A (1932). Manufacture of Para-Isopropyl-Alpha-Methyl Hydrocinnamic Aldehyde. U.S. Patent.

[92] Chapuis C, Skuy D, Richard C-A (2019). Chimia.

[93] Mine K, Fukada K (2004). Process for Producing Cycloalkanone Derivatives. Eur. Patent.

[94] Stetter H P D, Kuhlmann H D C (1976). Verfahren Zur Herstellung von Ketonen. Ger. Patent.

[95] Pecunioso A, Menicagli R (1988). J Org Chem.

[96] Tanaka K, Motomatsu S, Koyama K, Fukase K (2008). Tetrahedron Lett.

[97] Tanaka K, Fukase K (2009). Org Process Res Dev.

[98] Tanaka K, Motomatsu S, Koyama K, Tanaka S-i, Fukase K (2007). Org Lett.

[99] Doi T, Otaka H, Umeda K, Yoshida M (2015). Tetrahedron.

[100] Gauthier D R, Sherry B D, Cao Y, Journet M, Humphrey G, Itoh T, Mangion I, Tschaen D M (2015). Org Lett.

[101] Mukherjee S, Yang J W, Hoffmann S, List B (2007). Chem Rev.

[102] Zhou Q-L (2016). Angew Chem, Int Ed.

[103] Odedra A, Seeberger P H (2009). Angew Chem, Int Ed.

[104] Schober L, Ratnam S, Yamashita Y, Adebar N, Pieper M, Berkessel A, Hessel V, Gröger H (2019). Synthesis.

[105] Munirathinam R, Huskens J, Verboom W (2015). Adv Synth Catal.

[106] Bortolini O, Caciolli L, Cavazzini A, Costa V, Greco R, Massi A, Pasti L (2012). Green Chem.

[107] Ayats C, Henseler A H, Pericàs M A (2012). ChemSusChem.

[108] Ötvös S B, Mándity I M, Fülöp F (2012). J Catal.

[109] Nakashima E, Yamamoto H (2018). Chem – Eur J.

[110] Brown A J, Brunelli N A, Eum K, Rashidi F, Johnson J R, Koros W J, Jones C W, Nair S (2014). Science.

[111] Rezaei F, Lively R P, Labreche Y, Chen G, Fan Y, Koros W J, Jones C W (2013). ACS Appl Mater Interfaces.

[112] Moschetta E G, Negretti S, Chepiga K M, Brunelli N A, Labreche Y, Feng Y, Rezaei F, Lively R P, Koros W J, Davies H M L (2015). Angew Chem, Int Ed.

[113] He Y, Jawad A, Li X, Atanga M, Rezaei F, Rownaghi A A (2016). J Catal.

[114] Babich L, Hartog A F, van Hemert L J C, Rutjes F P J T, Wever R (2012). ChemSusChem.

[115] Kikhtyanin O, Hora L, Kubička D (2015). Catal Commun.

[116] Kaizik A, Fridag D, Lueken H G, Bueschken W (2013). Process for Preparing α,β-Unsaturated C10-Aldehydes. U.S. Patent.

[117] Bermann D, Frey G D, Schalapski K, Strutz H (2014). Method for Carrying Out Multiphase Aldol Condensation Reactions to Give Mixed α,β-Unsaturated Aldehydes. U.S Pat. Appl..

[118] Viviano M, Glasnov T N, Reichart B, Tekautz G, Kappe C O (2011). Org Process Res Dev.

[119] Bonrath W, Pressel Y, Schütz J, Ferfecki E, Topp K-D (2016). ChemCatChem.

[120] Laroche B, Saito Y, Ishitani H, Kobayashi S (2019). Org Process Res Dev.

[121] Zhou F, Liu H, Wen Z, Zhang B, Chen G (2018). Ind Eng Chem Res.

[122] Dobler W, Bahr N, Breuer K, Kindler A (2006). Continuous Process for Producing Pseudoionones and Ionones. U.S. Patent.

[123] Waldron C, Cao E, Cattaneo S, Brett G L, Miedziak P J, Wu G, Sankar M, Hutchings G J, Gavriilidis A (2019). Chem Eng J.

[124] Stevens J G, Bourne R A, Poliakoff M (2009). Green Chem.

[125] Chen A B, Li X, Zhou Y Z, Huang L L, Fang Z, Gan H F, Guo K (2013). Adv Mater Res.

[126] Zak J, Ron D, Riva E, Harding H P, Cross B C S, Baxendale I R (2012). Chem – Eur J.

[127] Alvarez-Casao Y, Marques-Lopez E, Herrera R P (2011). Symmetry.

[128] Milner S E, Moody T S, Maguire A R (2012). Eur J Org Chem.

[129] Luzzio F A (2001). Tetrahedron.

[130] Ballini R, Palmieri A (2006). Curr Org Chem.

[131] Ballini R, Petrini M, Rosini G (2008). Molecules.

[132] Rosini G, Ballini R, Sorrenti P (1983). Tetrahedron.

[133] Soldi L, Ferstl W, Loebbecke S, Maggi R, Malmassari C, Sartori G, Yada S (2008). J Catal.

[134] Biradar A V, Sharma K K, Asefa T (2010). Appl Catal, A.

[135] Lee J-W, Mayer-Gall T, Opwis K, Song C E, Gutmann J S, List B (2013). Science.

[136] Wang L, Xu G, Xiao J, Tao M, Zhang W (2019). Ind Eng Chem Res.

[137] Ogawa T, Kumagai N, Shibasaki M (2013). Angew Chem, Int Ed.

[138] Hashimoto K, Kumagai N, Shibasaki M (2014). Org Lett.

[139] Nonoyama A, Hashimoto K, Saito A, Kumagai N, Shibasaki M (2016). Tetrahedron Lett.

[140] Karasawa T, Oriez R, Kumagai N, Shibasaki M (2018). J Am Chem Soc.

[141] Nonoyama A, Kumagai N, Shibasaki M (2017). Tetrahedron.

[142] Parra-Cabrera C, Achille C, Kuhn S, Ameloot R (2018). Chem Soc Rev.

[143] Au A K, Huynh W, Horowitz L F, Folch A (2016). Angew Chem, Int Ed.

[144] Neumaier J M, Madani A, Klein T, Ziegler T (2019). Beilstein J Org Chem.

[145] Rossi S, Porta R, Brenna D, Puglisi A, Benaglia M (2017). Angew Chem, Int Ed.

[146] Dai Y-f, Tian H-y, Sun B-g, Sun Y-m, Chen H-t, Liu X-y (2012). J Chem Res.

[147] Mane J, Clinet J-C (2010). Substituted Octane(Ene) Nitriles, Methods for the Synthesis Thereof and Uses Thereof in Perfumery. U.S. Pat. Appl..

[148] DeSimone R S (1976). Process for the Preparation of α,β-Unsaturated Nitriles. U.S. Pat. Appl..

[149] Bonrath W, Medlock J, Lehmann H, Beumer R, Fischesser J (2015). Process for the Manufacture of Hydrogenated Nitriles. WO Pat. Appl..

[150] Hopp R, Thielmann T, Gottsch W (1993). Cyclopentyl-Cyanomethyl-Cyclopentenes, Process for Their Preparation and Their Use as Odoriferous Substances. U.S. Pat. Appl..

[151] Weiss M, Brunner B, Ebel K, Krause W (2010). rocess for the Preparation of Ethylgeranonitrile. U.S. Pat. Appl..

[152] Johnson F, Paul K G, Favara D (1977). Process for Preparing Methyl Jasmonate and Related Compounds. U.S. Pat. Appl..

[153] Flachsmann F, Bachmann J-P (2010). Cycloalkylidene-(Ortho Substituted Phenyl)-Acetonitriles and Their Use as Odorants. U.S. Patent.

[154] Angeletti E, Canepa C, Martinetti G, Venturello P (1989). J Chem Soc, Perkin Trans 1.

[155] Xu D Z, Shi S, Wang Y (2013). RSC Adv.

[156] Lai S M, Ng C P, Martin-Aranda R, Yeung K L (2003). Microporous Mesoporous Mater.

[157] Sylla M, Joseph D, Chevallier E, Camara C, Dumas F (2006). Synthesis.

[158] Zhang G, Zhang X, Lv J, Liu H, Qiu J, Yeung K L (2012). Catal Today.

[159] Zhang G, Zhang T, Zhang X, Yeung K L (2015). Catal Commun.

[160] Jackson T, Clark J H, Macquarrie D J, Brophy J H (2004). Green Chem.

[161] Fletcher P D I, Haswell S J, Pombo-Villar E, Warrington B H, Watts P, Wong S Y F, Zhang X (2002). Tetrahedron.

[162] Nikbin N, Watts P (2004). Org Process Res Dev.

[163] Vivier D, Soussia I B, Rodrigues N, Lolignier S, Devilliers M, Chatelain F C, Prival L, Chapuy E, Bourdier G, Bennis K (2017). J Med Chem.

[164] dos Anjos J V, Srivastava R M, Costa-Silva J H, Scotti L, Scotti M T, Wanderley A G, Leite E S, de Melo S J, Mendonça Junior F J B (2012). Molecules.

[165] Wang Y, Shi D, Tao S, Song W, Wang H, Wang X, Li G, Qiu J, Ji M (2016). ACS Sustainable Chem Eng.

[166] Song W, Shi D, Tao S, Li Z, Wang Y, Yu Y, Qiu J, Ji M, Wang X (2016). J Colloid Interface Sci.

[167] Long L-Y, Weng Y-X, Wang Y-Z (2018). Polymers (Basel, Switz).

[168] Tsutsumi Y, Koga H, Qi Z-D, Saito T, Isogai A (2014). Biomacromolecules.

[169] Qiao Y, Teng J, Wang S, Ma H (2017). Molecules.

[170] Xu G, Jin M, Kalkhajeh Y K, Wang L, Tao M, Zhang W (2019). J Cleaner Prod.

[171] Zhu H, Xu G, Du H, Zhang C, Ma N, Zhang W (2019). J Catal.

[172] Ishitani H, Kanai K, Saito Y, Tsubogo T, Kobayashi S (2017). Eur J Org Chem.

[173] Crawford D E, Miskimmin C K G, Albadarin A B, Walker G, James S L (2017). Green Chem.

[174] Crawford D E, Miskimmin C K, Cahir J, James S L (2017). Chem Commun.

[175] Sharma B M, Atapalkar R S, Kulkarni A A (2019). Green Chem.

[176] Yudin A K (2006). Aziridines and Epoxides in Organic Synthesis.

[177] de los Santos J M, Ochoa de Retana A M, Martínez de Marigorta E, Vicario J, Palacios F (2018). ChemCatChem.

[178] Rotstein B H, Zaretsky S, Rai V, Yudin A K (2014). Chem Rev.

[179] Brechtelsbauer C, Lewis N, Oxley P, Ricard F, Ramshaw C (2001). Org Process Res Dev.

[180] Michael A (1887). Am Chem J.

[181] Stork G, Brizzolara A, Landesman H, Szmuszkovicz J, Terrell R (1963). J Am Chem Soc.

[182] Thweatt J G (1969). Preparation of Hydrocoumarin, Coumarin, and Alkyl Substituted Derivatives. U.S. Pat. Appl..

[183] Wilson R A, Mookherjee B D, Hall J B, Stork G (1981). Process for the Preparation of Methyl Dihydrojasmonate and Lower Alkyl Homologues. U.S. Pat. Appl..

[184] Cohen A M (1975). Proces for the Preparation of 3-Mono-Alkyl and 3,6-Dialkyl-Resorcylic Esters. Brit. Pat. Appl..

[185] Strotman N A, Tan Y, Powers K W, Soumeillant M, Leung S W (2018). Org Process Res Dev.

[186] Christoffers J, Baro A (2003). Angew Chem, Int Ed.

[187] Zheng K, Liu X, Feng X (2018). Chem Rev.

[188] Christoffers J, Koripelly G, Rosiak A, Rössle M (2007). Synthesis.

[189] Hayashi M, Matsubara R (2017). Tetrahedron Lett.

[190] Hattori H (1995). Chem Rev.

[191] Borah P, Yamashita Y, Kobayashi S (2017). Angew Chem, Int Ed.

[192] Tsubogo T, Yamashita Y, Kobayashi S (2012). Chem – Eur J.

[193] Tsubogo T, Oyamada H, Kobayashi S (2015). Nature.

[194] Bandini M, Fagioli M, Umani-Ronchi A (2004). Adv Synth Catal.

[195] Melchiorre P, Marigo M, Carlone A, Bartoli G (2008). Angew Chem, Int Ed.

[196] Mondal A, Bhowmick S, Ghosh A, Chanda T, Bhowmick K C (2017). Tetrahedron: Asymmetry.

[197] Atodiresei I, Vila C, Rueping M (2015). ACS Catal.

[198] Tůma J, Kohout M (2018). RSC Adv.

[199] Scatena G S, de la Torre A F, Cass Q B, Rivera D G, Paixão M W (2014). ChemCatChem.

[200] Arakawa Y, Wennemers H (2013). ChemSusChem.

[201] Osorio-Planes L, Rodríguez-Escrich C, Pericàs M A (2016). Catal Sci Technol.

[202] Kasaplar P, Rodríguez-Escrich C, Pericàs M A (2013). Org Lett.

[203] Izquierdo J, Ayats C, Henseler A H, Pericàs M A (2015). Org Biomol Chem.

[204] Sagamanova I, Rodríguez-Escrich C, Molnár I G, Sayalero S, Gilmour R, Pericàs M A (2015). ACS Catal.

[205] Du J, Shuai B, Tao M, Wang G, Zhang W (2016). Green Chem.

[206] Ötvös S B, Pericàs M A, Kappe C O (2019). Chem Sci.

[207] Ogasawara S, Hayashi Y (2017). Synthesis.

[208] Hayashi Y, Ogasawara S (2016). Org Lett.

[209] Du L-H, Ling H-M, Luo X-P (2014). RSC Adv.

[210] Jadhav A S, Anand R V (2017). Eur J Org Chem.

[211] Jadhav A S, Anand R V (2017). Org Biomol Chem.

[212] Murray P R D, Browne D L, Pastre J C, Butters C, Guthrie D, Ley S V (2013). Org Process Res Dev.

[213] Feringa B L, Badorrey R, Pena D, Harutyunyan S R, Minnaard A J (2004). Proc Natl Acad Sci U S A.

[214] Bonrath W, Crevatin A, Aquino F (2005). Process for the Preparation of Methylheptenone.

[215] Campbell K N, Eby L T (1941). J Am Chem Soc.

[216] Vani P V S N, Chida A S, Srinivasan R, Chandrasekharam M, Singh A K (2001). Synth Commun.

[217] McMurry J E, Melton J (1971). J Am Chem Soc.

[218] Buchi G, Egger B (1971). J Org Chem.

[219] Selka A, Levesque N A, Foucher D, Clarisse O, Chemat F, Touaibia M (2017). Org Process Res Dev.

[220] Hall J B, Wiegers W J (1977). Process and Product Produced by Said Process. U.S. Pat. Appl..

[221] Gralla G, Heydrich G, Bergner E J, Ebel K (2011). Process for Preparation of Menthol by Hydrogenation of Isopulegol. U.S. Patent.

[222] Manuale D L, Betti C, Marchi A J, Yori J C, Romeo E (2010). Quim Nova.

[223] Cossar P J, Hizartzidis L, Simone M I, McCluskey A, Gordon C P (2015). Org Biomol Chem.

[224] Abdel-Hamid M K, Macgregor K A, Odell L R, Chau N, Mariana A, Whiting A, Robinson P J, McCluskey A (2015). Org Biomol Chem.

[225] Fiasella A, Nuzzi A, Summa M, Armirotti A, Tarozzo G, Tarzia G, Mor M, Bertozzi F, Bandiera T, Piomelli D (2014). ChemMedChem.

[226] Baumann M, Baxendale I R, Deplante F (2017). Beilstein J Org Chem.

[227] Fabry D C, Heddrich S, Sugiono E, Liauw M A, Rueping M (2019). React Chem Eng.

[228] Rossi S, Puglisi A, Benaglia M (2018). ChemCatChem.

[229] Silm E, Kaabel S, Järving I, Kanger T (2019). Synthesis.

[230] Boichenko M A, Babkin I Y, Kobylskoy S G, Chagarovskiy A O, Ivanova O A, Trushkov I V (2019). Molbank.

[231] Szabó B, Tamás B, Faigl F, Éles J, Greiner I (2019). J Flow Chem.

[232] Sharma Y, Nikam A V, Kulkarni A A (2019). Org Process Res Dev.

[233] Szabó B, Szakter K, Thurner A, Faigl F, Éles J, Greiner I (2020). Period Polytech, Chem Eng.

[234] Carter C F, Lange H, Sakai D, Baxendale I R, Ley S V (2011). Chem – Eur J.

[235] Chen Z, Mitchell S, Krumeich F, Hauert R, Yakunin S, Kovalenko M V, Pérez-Ramírez J (2019). ACS Sustainable Chem Eng.

[236] Büchele S, Chen Z, Mitchell S, Hauert R, Krumeich F, Pérez‐Ramírez J (2019). ChemCatChem.

[237] Chen Z, Vorobyeva E, Mitchell S, Fako E, López N, Collins S M, Leary R K, Midgley P A, Hauert R, Pérez-Ramírez J (2018). Natl Sci Rev.

[238] Albani D, Shahrokhi M, Chen Z, Mitchell S, Hauert R, López N, Pérez-Ramírez J (2018). Nat Commun.

[239] Coetzee J, Manyar H G, Hardacre C, Cole-Hamilton D J (2013). ChemCatChem.

[240] Schmölzer C, Nowikow C, Kählig H, Schmid W (2013). Carbohydr Res.

[241] Tran K T, Pallesen J S, Solbak S M Ø, Narayanan D, Baig A, Zang J, Aguayo-Orozco A, Carmona R M C, Garcia A D, Bach A (2019). J Med Chem.

[242] Sharma S, Yamini Y, Das P (2019). New J Chem.

[243] Manzoni L, Belvisi L, Bianchi A, Conti A, Drago C, de Matteo M, Ferrante L, Mastrangelo E, Perego P, Potenza D (2012). Bioorg Med Chem.

[244] De Vita E, Smits N, van den Hurk H, Beck E M, Hewitt J, Baillie G, Russell E, Pannifer A, Hamon V, Morrison A (2020). ChemMedChem.

[245] Bryan M C, Wernick D, Hein C D, Petersen J V, Eschelbach J W, Doherty E M (2011). Beilstein J Org Chem.

[246] Bryan M C, Hein C D, Gao H, Xia X, Eastwood H, Bruenner B A, Louie S W, Doherty E M (2013). ACS Comb Sci.

[247] Gintner M, Yoneda Y, Schmölzer C, Denner C, Kählig H, Schmid W (2019). Carbohydr Res.

[248] Rattray N J W, Zalloum W A, Mansell D, Latimer J, Jaffar M, Bichenkova E V, Freeman S (2013). Tetrahedron.

[249] Cooper C G F, Lee E R, Silva R A, Bourque A J, Clark S, Katti S, Nivorozhkin V (2012). Org Process Res Dev.

[250] Bana P, Szigetvári Á, Kóti J, Éles J, Greiner I (2019). React Chem Eng.

[251] Ponomarev K Y, Suslov E V, Zakharenko A L, Zakharova O D, Rogachev A D, Korchagina D V, Zafar A, Reynisson J, Nefedov A A, Volcho K P (2018). Bioorg Chem.

[252] Liu J, Fitzgerald A, Mani N (2012). Synthesis.

[253] Baumann M, Baxendale I R, Kuratli C, Ley S V, Martin R E, Schneider J (2011). ACS Comb Sci.

[254] Escribà-Gelonch M, de Leon Izeppi G A, Kirschneck D, Hessel V (2019). ACS Sustainable Chem Eng.

[255] Tukacs J M, Sylvester Á, Kmecz I, Jones R V, Óvári M, Mika L T (2019). R Soc Open Sci.

[256] Cova C M, Zuliani A, Muñoz-Batista M J, Luque R (2019). Green Chem.

[257] Cova C M, Zuliani A, Manno R, Sebastian V, Luque R (2020). Green Chem.

[258] Polyzos A, O'Brien M, Petersen T P, Baxendale I R, Ley S V (2011). Angew Chem, Int Ed.

[259] Mercadante M A, Kelly C B, (Xiang) Lee C, Leadbeater N E (2012). Org Process Res Dev.

[260] Dalla-Vechia L, Reichart B, Glasnov T, Miranda L S M, Kappe C O, de Souza R O M A (2013). Org Biomol Chem.

[261] Glasnov T N, Kappe C O (2010). Adv Synth Catal.

[262] Baxendale I R, Deeley J, Griffiths-Jones C M, Ley S V, Saaby S, Tranmer G K (2006). Chem Commun.

[263] Obermayer D, Glasnov T N, Kappe C O (2011). J Org Chem.

[264] Brasholz M, Macdonald J M, Saubern S, Ryan J H, Holmes A B (2010). Chem – Eur J.

[265] O'Brien M, Taylor N, Polyzos A, Baxendale I R, Ley S V (2011). Chem Sci.

[266] Kobayashi J, Mori Y, Okamoto K, Akiyama R, Ueno M, Kitamori T, Kobayashi S (2004). Science.

[267] Kobayashi J, Mori Y, Kobayashi S (2005). Chem Commun.

[268] Bakker J J W, Zieverink M M P, Reintjens R W E G, Kapteijn F, Moulijn J A, Kreutzer M T (2011). ChemCatChem.

[269] Britton J, Stubbs K A, Weiss G A, Raston C L (2017). Chem – Eur J.

[270] Phillips J M, Ahamed M, Duan X, Lamb R N, Qu X, Zheng K, Zou J, Chalker J M, Raston C L (2019). ACS Appl Bio Mater.

[271] Avril A, Hornung C H, Urban A, Fraser D, Horne M, Veder J-P, Tsanaktsidis J, Rodopoulos T, Henry C, Gunasegaram D R (2017). React Chem Eng.

[272] Hornung C H, Nguyen X, Carafa A, Gardiner J, Urban A, Fraser D, Horne M D, Gunasegaram D R, Tsanaktsidis J (2017). Org Process Res Dev.

[273] Nguyen X, Carafa A, Hornung C H (2018). Chem Eng Process.

[274] Gardiner J, Nguyen X, Genet C, Horne M D, Hornung C H, Tsanaktsidis J (2018). Org Process Res Dev.

[275] Takahashi Y, Mitsudome T, Mizugaki T, Jitsukawa K, Kaneda K (2013). Green Chem.

[276] Russell M G, Veryser C, Hunter J F, Beingessner R L, Jamison T F (2020). Adv Synth Catal.

[277] Sachse A, Linares N, Barbaro P, Fajula F, Galarneau A (2013). Dalton Trans.

[278] Linares N, Moreno-Marrodan C, Barbaro P (2016). ChemCatChem.

[279] Strohmann M, Bordet A, Vorholt A J, Leitner W (2019). Green Chem.

[280] Lázaro N, Franco A, Ouyang W, Balu A, Romero A, Luque R, Pineda A (2019). Catalysts.

[281] Ouyang W, Zhao D, Wang Y, Balu A M, Len C, Luque R (2018). ACS Sustainable Chem Eng.

[282] Brandi F, Bäumel M, Molinari V, Shekova I, Lauermann I, Heil T, Antonietti M, Al-Naji M (2020). Green Chem.

[283] Hattori T, Ida T, Tsubone A, Sawama Y, Monguchi Y, Sajiki H (2015). Eur J Org Chem.

[284] Miyamura H, Suzuki A, Yasukawa T, Kobayashi S (2018). J Am Chem Soc.

[285] Kobayashi S, Okumura M, Akatsuka Y, Miyamura H, Ueno M, Oyamada H (2015). ChemCatChem.

[286] Oyamada H, Naito T, Kobayashi S (2011). Beilstein J Org Chem.

[287] Johnson M D, May S A, Calvin J R, Remacle J, Stout J R, Diseroad W D, Zaborenko N, Haeberle B D, Sun W-M, Miller M T (2012). Org Process Res Dev.

[288] Meemken F, Baiker A (2017). Chem Rev.

[289] Ager D J, de Vries A H M, de Vries J G (2012). Chem Soc Rev.

[290] Sullivan R J, Newman S G (2018). Chem Sci.

[291] Tripathi S, Singh S N, Yadav L D S (2016). RSC Adv.

[R292] 292Fritzsche. No Title. DE 207,702, 1909.

[293] Kapelle I B D, Irawadi T T, Rusli M S, Mangunwidjaja D, Mas’ud Z A (2015). Procedia Chem.

[294] Fisher G S, Stinson J S, Goldblatt L A (1953). J Am Chem Soc.

[295] Wang S, Zhang A (2008). Org Prep Proced Int.

[296] Gavriilidis A, Constantinou A, Hellgardt K, Hii K K (Mimi), Hutchings G J, Brett G L, Kuhn S, Marsden S P (2016). React Chem Eng.

[297] Gemoets H P L, Su Y, Shang M, Hessel V, Luque R, Noël T (2016). Chem Soc Rev.

[298] Wang N, Matsumoto T, Ueno M, Miyamura H, Kobayashi S (2009). Angew Chem, Int Ed.

[299] Osako T, Torii K, Uozumi Y (2015). RSC Adv.

[300] Mannel D S, Stahl S S, Root T W (2014). Org Process Res Dev.

[301] Zotova N, Hellgardt K, Kelsall G H, Jessiman A S, Hii K K (Mimi) (2010). Green Chem.

[302] Kaizuka K, Lee K-Y, Miyamura H, Kobayashi S (2012). J Flow Chem.

[303] Wu G, Constantinou A, Cao E, Kuhn S, Morad M, Sankar M, Bethell D, Hutchings G J, Gavriilidis A (2015). Ind Eng Chem Res.

[304] Dhakshinamoorthy A, Navalon S, Asiri A M, Garcia H (2020). Chem Commun.

[305] Vanoye L, Pablos M, Smith N, de Bellefon C, Favre-Réguillon A (2014). RSC Adv.

[306] Vanoye L, Aloui A, Pablos M, Philippe R, Percheron A, Favre-Réguillon A, de Bellefon C (2013). Org Lett.

[307] Ötvös S B, Llanes P, Pericàs M A, Kappe C O (2020). Org Lett.

[308] Sedelmeier J, Ley S V, Baxendale I R, Baumann M (2010). Org Lett.

[309] Bourne S L, Ley S V (2013). Adv Synth Catal.

[310] Burkholder M, Gilliland S E, Luxon A, Tang C, Gupton B F (2019). Catalysts.

[311] Pieber B, Kappe C O (2013). Green Chem.

[312] Park C Y, Park J H, Lim H J, Hwang G-S, Park C P (2014). Bull Korean Chem Soc.

[313] Park C Y, Kim Y J, Lim H J, Park J H, Kim M J, Seo S W, Park C P (2015). RSC Adv.

[314] Maurya R A, Park C P, Kim D-P (2011). Beilstein J Org Chem.

[315] Lévesque F, Seeberger P H (2011). Org Lett.

[316] Hook B D A, Dohle W, Hirst P R, Pickworth M, Berry M B, Booker-Milburn K I (2005). J Org Chem.

[317] Matsuura S-i, Yokoyama T, Ishii R, Itoh T, Tomon E, Hamakawa S, Tsunoda T, Mizukami F, Nanbu H, Hanaoka T-a (2012). Chem Commun.

[318] Cvjetko M, Vorkapić-Furač J, Žnidaršič-Plazl P (2012). Process Biochem (Oxford, U K).

[319] Illner S, Hofmann C, Löb P, Kragl U (2014). ChemCatChem.

[320] Greene J F, Hoover J M, Mannel D S, Root T W, Stahl S S (2013). Org Process Res Dev.

[321] Ye X, Johnson M D, Diao T, Yates M H, Stahl S S (2010). Green Chem.

[322] Sharma Y, Moolya S, Joshi R A, Kulkarni A A (2017). React Chem Eng.

[323] Bogdan A, McQuade D T (2009). Beilstein J Org Chem.

[324] Okuno Y, Kitagawa Y, Kamiya S, Hasegawa A, Kawashima T, Otani K, Aoki S, Kanno J, Isomura S, Sato Y (2018). Asian J Org Chem.

[325] Schulze J S, Migenda J, Becker M, Schuler S M M, Wende R C, Schreiner P R, Smarsly B M (2020). J Mater Chem A.

[326] Aellig C, Scholz D, Conrad S, Hermans I (2013). Green Chem.

[327] Albanese D C M, Foschi F, Penso M (2016). Org Process Res Dev.

[328] Leduc A B, Jamison T F (2012). Org Process Res Dev.

[329] Kairouz V, Collins S K (2018). J Chem Educ.

[330] Zhang Y, Born S C, Jensen K F (2014). Org Process Res Dev.

[331] Peer M, Weeranoppanant N, Adamo A, Zhang Y, Jensen K F (2016). Org Process Res Dev.

[332] He W, Fang Z, Tian Q, Shen W, Guo K (2016). Ind Eng Chem Res.

[333] He W, Fang Z, Tian Q, Ji D, Zhang K, Guo K (2015). Chem Eng Process.

[334] Cossar P J, Baker J R, Cain N, McCluskey A (2018). R Soc Open Sci.

[335] Vanoye L, Wang J, Pablos M, de Bellefon C, Favre-Réguillon A (2016). Catal Sci Technol.

[336] Vanoye L, Hamami Z E, Wang J, de Bellefon C, Fongarland P, Favre‐Réguillon A (2017). Eur J Lipid Sci Technol.

[337] Wan Y S S, Chau J L H, Gavriilidis A, Yeung K L (2002). Chem Commun.

[338] Aigner M, Grosso-Giordano N A, Okrut A, Zones S, Katz A (2017). React Chem Eng.

[339] Wulff H P (1974). Heterogeneous Catalysts for Olefin Epoxidation. U.S. Pat. Appl..

[340] Dai W, Li G, Chen B, Wang L, Gao S (2015). Org Lett.

[341] Dai W, Mi Y, Lv Y, Shang S, Li G, Chen G, Gao S (2016). Synthesis.

[342] Kee S-P, Gavriilidis A (2009). Org Process Res Dev.

[343] Wiles C, Hammond M J, Watts P (2009). Beilstein J Org Chem.

[344] Wada Y, Schmidt M A, Jensen K F (2006). Ind Eng Chem Res.

[345] Hübner S, Bentrup U, Budde U, Lovis K, Dietrich T, Freitag A, Küpper L, Jähnisch K (2009). Org Process Res Dev.

[346] Steinfeldt N, Abdallah R, Dingerdissen U, Jähnisch K (2007). Org Process Res Dev.

[347] Steinfeldt N, Bentrup U, Jähnisch K (2010). Ind Eng Chem Res.

[348] Irfan M, Glasnov T N, Kappe C O (2011). Org Lett.

[349] O’Brien M, Baxendale I R, Ley S V (2010). Org Lett.

[350] Kendall A J, Barry J T, Seidenkranz D T, Ryerson A, Hiatt C, Salazar C A, Bryant D J, Tyler D R (2016). Tetrahedron Lett.

[351] Schiaffo C E, Dussault P H (2008). J Org Chem.

[352] Roydhouse M D, Ghaini A, Constantinou A, Cantu-Perez A, Motherwell W B, Gavriilidis A (2011). Org Process Res Dev.

[353] Roydhouse M D, Motherwell W B, Constantinou A, Gavriilidis A, Wheeler R, Down K, Campbell I (2013). RSC Adv.

[354] Ren J, He S, Ye C, Chen G, Sun C (2012). Chem Eng J.

[355] Lundin M D, Danby A M, Akien G R, Venkitasubramanian P, Martin K J, Busch D H, Subramaniam B (2017). AIChE J.

[356] Qi B, Yang H-Y, Wang Z-Q (2007). Chin J Chem.

[357] Gallimore P J, Griffiths P T, Pope F D, Reid J P, Kalberer M (2017). J Geophys Res: Atmos.

[358] Porterfield J P, Eibenberger S, Patterson D, McCarthy M C (2018). Phys Chem Chem Phys.

[359] Hone C A, O'Kearney-McMullan A, Munday R, Kappe C O (2017). ChemCatChem.

[360] Borlinghaus J, Albrecht F, Gruhlke M C H, Nwachukwu I D, Slusarenko A J (2014). Molecules.

[361] Albrecht F, Leontiev R, Jacob C, Slusarenko A (2017). Molecules.

[362] Silva F, Khokhar S S, Williams D M, Saunders R, Evans G J S, Graz M, Wirth T (2018). Angew Chem, Int Ed.

[363] Block E, Ahmad S, Jain M K, Crecely R W, Apitz-Castro R, Cruz M R (1984). J Am Chem Soc.

[364] Dong B S, Mi Y Y, Ho M S, Jae H H, Sang H L, Yong S C (2012). Process for Preparing Ajoene from Garlic. U.S. Pat. Appl..

[365] Colomer J P, Traverssi M, Oksdath-Mansilla G (2020). J Flow Chem.

[366] Doherty S, Knight J G, Carroll M A, Clemmet A R, Ellison J R, Backhouse T, Holmes N, Thompson L A, Bourne R A (2016). RSC Adv.

[367] Doherty S, Knight J G, Carroll M A, Ellison J R, Hobson S J, Stevens S, Hardacre C, Goodrich P (2015). Green Chem.

[368] Silva F, Baker A, Stansall J, Michalska W, Yusubov M S, Graz M, Saunders R, Evans G J S, Wirth T (2018). Eur J Org Chem.

[369] Laudadio G, Straathof N J W, Lanting M D, Knoops B, Hessel V, Noël T (2017). Green Chem.

[370] Gu C A, Gu L (2015). Continuous Oxidation Method for Preparing Hexanoic Acid by Using Nitric Acid Oxidation Octanol. Chin. Pat. Appl..

[371] Wang Z, Deng J (2014). Method and Equipment for Preparing Aldehyde or Ketone through Continuous Oxidization by Oxygen. Chin. Pat. Appl..

[372] Sharley J S, Baxendale I R, Espinos Ferri E, Collado Perez A M, Fernandez Fernandez I, Quesada J S (2018). Chemical Process of Preparing Dehydrohedione. WO Pat. Appl..

[373] Klein E, Farnow H, Rojahn W (1964). Justus Liebigs Ann Chem.

[374] Miyake A, Nishino M, Kondo H (1974). 5-Cyclohexadecen-1-on Verfahren zu dessen Herstellung und seine Verwendung als Riechstoff.

[375] Watanabe S, Ujihara H, Yamamoto T, Hagiwara T (2001). Production Process of Cyclohexenyl Methyl Ketones. Eur. Pat. Appl..

[376] Serra S (2015). Molecules.

[377] Grachev A A, Klochkov A O, Shiryaev V I (2012). Russ J Appl Chem.

[378] Goldbach M, Danieli E, Perlo J, Kaptein B, Litvinov V M, Blümich B, Casanova F, Duchateau A L L (2016). Tetrahedron Lett.

[379] Huck L, de la Hoz A, Díaz-Ortiz A, Alcázar J (2017). Org Lett.

[380] Deng Y, Wei X-J, Wang X, Sun Y, Noël T (2019). Chem – Eur J.

[381] Riva E, Gagliardi S, Martinelli M, Passarella D, Vigo D, Rencurosi A (2010). Tetrahedron.

[382] Hamlin T A, Lazarus G M L, Kelly C B, Leadbeater N E (2014). Org Process Res Dev.

[383] Hamlin T A, Kelly C B, Cywar R M, Leadbeater N E (2014). J Org Chem.

[384] Kupracz L, Kirschning A (2013). Adv Synth Catal.

[385] Battilocchio C, Baxendale I R, Biava M, Kitching M O, Ley S V (2012). Org Process Res Dev.

[386] Battilocchio C, Guetzoyan L, Cervetto C, Di Cesare Mannelli L, Frattaroli D, Baxendale I R, Maura G, Rossi A, Sautebin L, Biava M (2013). ACS Med Chem Lett.

[387] Korwar S, Amir S, Tosso P N, Desai B K, Kong C J, Fadnis S, Telang N S, Ahmad S, Roper T D, Gupton B F (2017). Eur J Org Chem.

[388] de M. Muñoz J, Alcázar J, de la Hoz A, Díaz-Ortiz Á, Alonso de Diego S-A (2012). Green Chem.

[389] Mateos C, Rincón J A, Villanueva J (2013). Tetrahedron Lett.

[390] Williams J D, Kerr W J, Leach S G, Lindsay D M (2018). Angew Chem, Int Ed.

[391] Zhang C-T, Zhu R, Wang Z, Ma B, Zajac A, Smiglak M, Xia C-N, Castle S L, Wang W-L (2019). RSC Adv.

[392] Pedersen M J, Holm T L, Rahbek J P, Skovby T, Mealy M J, Dam-Johansen K, Kiil S (2013). Org Process Res Dev.

[393] Brodmann T, Koos P, Metzger A, Knochel P, Ley S V (2012). Org Process Res Dev.

[394] Wiss J, Länzlinger M, Wermuth M (2005). Org Process Res Dev.

[395] Hellings M, Van den Kerkhof T (2020). Applying PAT in Chemical Process Development.

[396] Odille F G J, Stenemyr A, Pontén F (2014). Org Process Res Dev.

[397] Kopach M E, Cole K P, Pollock P M, Johnson M D, Braden T M, Webster L P, McClary Groh J, McFarland A D, Schafer J P, Adler J J (2016). Org Process Res Dev.

[398] Wu J, Yang X, He Z, Mao X, Hatton T A, Jamison T F (2014). Angew Chem, Int Ed.

[399] He Z, Jamison T F (2014). Angew Chem, Int Ed.

[400] Maier M C, Lebl R, Sulzer P, Lechner J, Mayr T, Zadravec M, Slama E, Pfanner S, Schmölzer C, Pöchlauer P (2019). React Chem Eng.

[401] Deng Q, Shen R, Ding R, Zhang L (2014). Adv Synth Catal.

[402] Lixiong Z, Lin D, Shen W (2014). Method for Preparing Propargyl Alcohol by Using Micro-Structural Reactor. Chin. Pat. Appl..

[403] Watanabe S, Nakaya N, Akai J, Kanaori K, Harada T (2018). Org Lett.

[404] Nowicki J (2000). Molecules.

[405] Luettin A, Stritt C (2010). Process for the Preparation of B-Ionones and Vitmain A, Vitamin A Derivatives, Carotenes and Carotenoids. U.S. Pat. Appl..

[406] Schulte-Elte K-H, Willhalm B, Gautchl F (1979). Cyclic C6 Ketones in Perfumes. U.S. Patent.

[407] Shen D L, He X, Guan W, Liu Y P, Sun B G (2013). Adv Mater Res.

[408] Blackmore R L (1966). Production of Terpene Compounds. U.S. Pat. Appl..

[409] Cantillo D, de Frutos O, Rincón J A, Mateos C, Kappe C O (2014). J Org Chem.

[410] Fabry D C, Ronge M A, Rueping M (2015). Chem – Eur J.

[411] Baxendale I R, Lee A-L, Ley S V (2002). Synlett.

[412] Billaud E M F, Shahbazali E, Ahamed M, Cleeren F, Noël T, Koole M, Verbruggen A, Hessel V, Bormans G (2017). Chem Sci.

[413] Sauks J M, Mallik D, Lawryshyn Y, Bender T, Organ M (2014). Org Process Res Dev.

[414] Shahbazali E, Spapens M, Kobayashi H, Ookawara S, Noël T, Hessel V (2015). Chem Eng J.

[415] Hessel V, Shahbazali E, Noël T, Zelentsov S (2014). Chem Ing Tech.

[416] Maeda H, Nashihara S, Mukae H, Yoshimi Y, Mizuno K (2013). Res Chem Intermed.

[417] Rincón J A, Barberis M, González-Esguevillas M, Johnson M D, Niemeier J K, Sun W-M (2011). Org Process Res Dev.

[418] Egami H, Tamaoki S, Abe M, Ohneda N, Yoshimura T, Okamoto T, Odajima H, Mase N, Takeda K, Hamashima Y (2018). Org Process Res Dev.

[419] Kobayashi H, Driessen B, van Osch D J G P, Talla A, Ookawara S, Noël T, Hessel V (2013). Tetrahedron.

[420] Baxendale I R, Hornung C, Ley S V, de Mata Muñoz Molina J, Wikström A (2013). Aust J Chem.

[421] Xin Q, Jindong H, Xiangyang T, Wentao Z (2015). Method for Preparing Ocimene from Thermo Isomeric Alpha-Pinene in Liquid Phase. Chin. Pat. Appl..

[422] Ouchi T, Mutton R J, Rojas V, Fitzpatrick D E, Cork D G, Battilocchio C, Ley S V (2016). ACS Sustainable Chem Eng.

[423] Funel J-A, Abele S (2013). Angew Chem, Int Ed.

[424] Fráter G, Müller U, Schröder F (2004). Tetrahedron: Asymmetry.

[425] Ayyar K S, Cookson R C, Kagi D A (1975). J Chem Soc, Perkin Trans 1.

[426] Baxendale I R (2015). Chem Eng Technol.

[427] Hornung C H, Álvarez-Diéguez M Á, Kohl T M, Tsanaktsidis J (2017). Beilstein J Org Chem.

[428] Filipponi P, Gioiello A, Baxendale I R (2016). Org Process Res Dev.

[429] Hartman R L (2012). Org Process Res Dev.

[430] Polster C S, Cole K P, Burcham C L, Campbell B M, Frederick A L, Hansen M M, Harding M, Heller M R, Miller M T, Phillips J L (2014). Org Process Res Dev.

[431] Hartman R L, Naber J R, Zaborenko N, Buchwald S L, Jensen K F (2010). Org Process Res Dev.

[432] Seghers S, Lefevere J, Mullens S, De Vylder A, Thybaut J W, Stevens C V (2018). ChemSusChem.

[433] Seghers S, Protasova L, Mullens S, Thybaut J W, Stevens C V (2017). Green Chem.

[434] He W, Fang Z, Yang Z, Ji D, Guo K (2015). RSC Adv.

[435] Sachse A, Hulea V, Finiels A, Coq B, Fajula F, Galarneau A (2012). J Catal.

[436] Ahrendt K A, Borths C J, MacMillan D W C (2000). J Am Chem Soc.

[437] Austin J F, MacMillan D W C (2002). J Am Chem Soc.

[438] Chiroli V, Benaglia M, Cozzi F, Puglisi A, Annunziata R, Celentano G (2013). Org Lett.

[439] Porta R, Benaglia M, Chiroli V, Coccia F, Puglisi A (2014). Isr J Chem.

[440] Chiroli V, Benaglia M, Puglisi A, Porta R, Jumde R P, Mandoli A (2014). Green Chem.

[441] Altava B, Burguete M I, García-Verdugo E, Luis S V, Vicent M J (2006). Green Chem.

[442] Abele S, Höck S, Schmidt G, Funel J-A, Marti R (2012). Org Process Res Dev.

[443] Damm M, Glasnov T N, Kappe C O (2010). Org Process Res Dev.

[444] Tsoung J, Wang Y, Djuric S W (2017). React Chem Eng.

[445] Nakashima E, Yamamoto H (2015). Chem Commun.

[446] Bai‐cheng F, Xi‐chao H, Tie‐lin W, Jian‐qiang L, Yan J (2020). ChemistrySelect.

[447] Karl D, Löwe H (2017). J Flow Chem.

[448] Misuk V, Mai A, Zhao Y, Heinrich J, Rauber D, Giannopoulos K, Löwe H (2015). J Flow Chem.

[449] Khadra A, Organ M G (2016). J Flow Chem.

[450] Spare L K, Harman D G, Aldrich-Wright J R, Nguyen T V, Gordon C P (2018). Adv Synth Catal.

[451] Martin R E, Morawitz F, Kuratli C, Alker A M, Alanine A I (2012). Eur J Org Chem.

[452] Lehmann J, Alzieu T, Martin R E, Britton R (2013). Org Lett.

[453] Yokozawa S, Ohneda N, Muramatsu K, Okamoto T, Odajima H, Ikawa T, Sugiyama J-i, Fujita M, Sawairi T, Egami H (2015). RSC Adv.

[454] Bah J, Franzén J (2014). Chem – Eur J.

[455] Nekkaa I, Palkó M, Mándity I M, Fülöp F (2018). Beilstein J Org Chem.

[456] Nekkaa I, Palkó M, Mándity I M, Miklós F, Fülöp F (2018). Eur J Org Chem.

[457] Ötvös S B, Fülöp F (2015). Catal Sci Technol.

[458] Bao J, Tranmer G K (2015). Chem Commun.

[459] Kappe C O, Van der Eycken E (2010). Chem Soc Rev.

[460] Johansson J R, Beke-Somfai T, Said Stålsmeden A, Kann N (2016). Chem Rev.

[461] Haldón E, Nicasio M C, Pérez P J (2015). Org Biomol Chem.

[462] Banerji B, Chandrasekhar K, Killi S K, Pramanik S K, Uttam P, Sen S, Maiti N C (2016). R Soc Open Sci.

[463] Meščić A, Šalić A, Gregorić T, Zelić B, Raić-Malić S (2017). RSC Adv.

[464] Teci M, Tilley M, McGuire M A, Organ M G (2016). Org Process Res Dev.

[465] Sadler S, Sebeika M M, Kern N L, Bell D E, Laverack C A, Wilkins D J, Moeller A R, Nicolaysen B C, Kozlowski P N, Wiles C (2014). J Flow Chem.

[466] Leforestier B, Vögtle M (2016). Synlett.

[467] Salvador C E M, Pieber B, Neu P M, Torvisco A, Andrade C K Z, Kappe C O (2015). J Org Chem.

[468] Bédard A-C, Santandrea J, Collins S K (2015). J Flow Chem.

[469] Pan S, Yan S, Osako T, Uozumi Y (2017). ACS Sustainable Chem Eng.

[470] Wen J, Wu K, Yang D, Tian J, Huang Z, Filatov A S, Lei A, Lin X-M (2018). ACS Appl Mater Interfaces.

[471] Burilov V A, Nurmukhametova A N, Belov R N, Mironova D A, Vorob´ev V V, Osin Y N, Antipin I S (2018). Russ Chem Bull.

[472] De Angelis S, Franco M, Triminì A, González A, Sainz R, Degennaro L, Romanazzi G, Carlucci C, Petrelli V, de la Esperanza A (2019). Chem – Asian J.

[473] Manzano J S, Weinstein Z B, Sadow A D, Slowing I I (2017). ACS Catal.

[474] Pucci A, Albano G, Pollastrini M, Lucci A, Colalillo M, Oliva F, Evangelisti C, Marelli M, Santalucia D, Mandoli A (2020). Catalysts.

[475] Cao J, Xu G, Li P, Tao M, Zhang W (2017). ACS Sustainable Chem Eng.

[476] Manneveau M, Tanii S, Gens F, Legros J, Chataigner I (2020). Org Biomol Chem.

[477] Baumann M, Baxendale I R, Ley S V (2010). Synlett.

[478] Grafton M, Mansfield A C, Fray M J (2010). Tetrahedron Lett.

[479] Cludius-Brandt S, Kupracz L, Kirschning A (2013). Beilstein J Org Chem.

[480] Chandrasekhar D, Borra S, Kapure J S, Shivaji G S, Srinivasulu G, Maurya R A (2015). Org Chem Front.

[481] Britton J, Jamison T F (2017). Angew Chem, Int Ed.

[482] Audubert C, Gamboa Marin O J, Lebel H (2017). Angew Chem, Int Ed.

[483] Comas-Barceló J, Blanco-Ania D, van den Broek S A M W, Nieuwland P J, Harrity J P A, Rutjes F P J T (2016). Catal Sci Technol.

[484] Jamison T F, Palde P B (2013). Use of Azides in Synthesis. U.S. Pat. Appl..

[485] Daisuke-shi N, Imagawa T, Hirokawa K, Fumitaka M (2017). Method for Producing 1H-Tetrazole Derivative. WO Pat. Appl..

[486] Castellano S, Tamborini L, Viviano M, Pinto A, Sbardella G, Conti P (2010). J Org Chem.

[487] Brasholz M, Saubern S, Savage G P (2011). Aust J Chem.

[488] Mojzesová M, Mečiarová M, Almássy A, Marti R, Šebesta R (2015). Chem Pap.

[489] Takeda K, Oohara T, Shimada N, Nambu H, Hashimoto S (2011). Chem – Eur J.

[490] Chandrasekhar D, Borra S, Nanubolu J B, Maurya R A (2016). Org Lett.

[491] Rincón J A, Mateos C, García-Losada P, Mergott D J (2015). Org Process Res Dev.

[492] Sarkar D, Bera N, Ghosh S (2020). Eur J Org Chem.

[493] Namyslo J C, Kaufmann D E (2003). Chem Rev.

[494] Crimmins M T, Reinhold T L (1993). Enone Olefin [2 + 2] Photochemical Cycloadditions. Organic Reactions.

[495] Levterov V V, Michurin O, Borysko P O, Zozulya S, Sadkova I V, Tolmachev A A, Mykhailiuk P K (2018). J Org Chem.

[496] Knowles J P, Elliott L D, Booker-Milburn K I (2012). Beilstein J Org Chem.

[497] Sambiagio C, Noël T (2020). Trends Chem.

[498] Mukae H, Maeda H, Nashihara S, Mizuno K (2007). Bull Chem Soc Jpn.

[499] Vasudevan A, Villamil C, Trumbull J, Olson J, Sutherland D, Pan J, Djuric S (2010). Tetrahedron Lett.

[500] Bachollet S, Terao K, Aida S, Nishiyama Y, Kakiuchi K, Oelgemöller M (2013). Beilstein J Org Chem.

[501] Poscharny K, Fabry D C, Heddrich S, Sugiono E, Liauw M A, Rueping M (2018). Tetrahedron.

[502] Fukuyama T, Hino Y, Kamata N, Ryu I (2004). Chem Lett.

[503] Telmesani R, Park S H, Lynch-Colameta T, Beeler A B (2015). Angew Chem, Int Ed.

[504] Telmesani R, White J A H, Beeler A B (2018). ChemPhotoChem.

[505] Schachtner J, Bayer P, Jacobi von Wangelin A (2016). Beilstein J Org Chem.

[506] Ratković A, Marinić Ž, Škorić I (2018). J Mol Struct.

[507] Elliott L D, Knowles J P, Koovits P J, Maskill K G, Ralph M J, Lejeune G, Edwards L J, Robinson R I, Clemens I R, Cox B (2014). Chem – Eur J.

[508] Ralph M, Ng S, Booker-Milburn K I (2016). Org Lett.

[509] DeLaney E N, Lee D S, Elliott L D, Jin J, Booker-Milburn K I, Poliakoff M, George M W (2017). Green Chem.

[510] Maskill K G, Knowles J P, Elliott L D, Alder R W, Booker-Milburn K I (2013). Angew Chem, Int Ed.

[511] Blackham E E, Booker-Milburn K I (2017). Angew Chem, Int Ed.

[512] Yu W L, Nunns T, Richardson J, Booker-Milburn K I (2018). Org Lett.

[513] Nakano M, Nishiyama Y, Tanimoto H, Morimoto T, Kakiuchi K (2016). Org Process Res Dev.

[514] Sender M, Ziegenbalg D (2017). Chem Ing Tech.

[515] Williams J D, Nakano M, Gérardy R, Rincón J A, de Frutos Ó, Mateos C, Monbaliu J-C M, Kappe C O (2019). Org Process Res Dev.

[516] Yang C, Li R, Zhang K A I, Lin W, Landfester K, Wang X (2020). Nat Commun.

[517] Elliott L D, Berry M, Harji B, Klauber D, Leonard J, Booker-Milburn K I (2016). Org Process Res Dev.

[518] Kurahashi K, Takemoto Y, Takasu K (2012). ChemSusChem.

[519] Hafez A M, Taggi A E, Wack H, Drury W J, Lectka T (2000). Org Lett.

[520] Hafez A M, Taggi A E, Lectka T (2002). Chem – Eur J.

[521] Hafner A, Ley S V (2015). Synlett.

[522] Battilocchio C, Iannucci G, Wang S, Godineau E, Kolleth A, De Mesmaeker A, Ley S V (2017). React Chem Eng.

[523] Ueda M, Hayama M, Hashishita H, Munechika A, Fukuyama T (2019). Eur J Org Chem.

[524] García-Lacuna J, Domínguez G, Blanco-Urgoiti J, Pérez-Castells J (2018). Org Lett.

[525] Saaby S, Baxendale I R, Ley S V (2005). Org Biomol Chem.

[526] Wilson N S, Sarko C R, Roth G P (2004). Org Process Res Dev.

[527] Scriabine I (1962). Alkylaromatic Aldehydes and Their Preparation. U.S. Pat. Appl..

[528] Wood T, Heilweil E (1962). Process for Producing 1,1,3,4,4,6-Hexamethyl-1,2,3,4-Tetrahydronapthalene (Hmt). U.S. Pat. Appl..

[529] Polak E H (1958). A Process for the Preparation of Musky Odor Polyalkylindanderivaten. Ger. Patent.

[530] Calloway N O (1935). Chem Rev.

[531] Rueping M, Nachtsheim B J (2010). Beilstein J Org Chem.

[532] Arata K, Toyoshima I (1977). J Catal.

[533] Hitzler M G, Smail F R, Poliakoff M, Hitzler M G, Ross S K (1998). Chem Commun.

[534] Bogdan A R, Poe S L, Kubis D C, Broadwater S J, McQuade D T (2009). Angew Chem, Int Ed.

[535] Snead D R, Jamison T F (2015). Angew Chem, Int Ed.

[536] Tietze L F, Liu D (2008). ARKIVOC.

[537] Li W, Yang S, Guo X, He G, Jin H (2018). Chin J Chem Eng.

[538] Fang H, Xiao Q, Wu F, Floreancig P E, Weber S G (2010). J Org Chem.

[539] Hone C A, Boyd A, O'Kearney-McMullan A, Bourne R A, Muller F L (2019). React Chem Eng.

[540] Koo H, Kim H Y, Oh K (2019). Org Chem Front.

[541] Koo H, Kim H Y, Oh K (2019). Org Lett.

[542] Wang X, Wang S, Ge X, Qui T, Wang H, Wang Q, Chen J, Huang Z, Ye C, Li L (2019). Micro-Continuous Flow Process for Producing Musk Tonalide. Chin. Pat. Appl..

[543] Aribert N, Camy S, Peres Lucchese Y, Condoret J-S, Cognet P (2010). Int J Chem React Eng.

[544] Chen Z, Chen W, Tong T, Zeng A (2015). J Mol Catal A: Chem.

[545] Rao X, Ishitani H, Yoo W-J, Kobayashi S (2019). Asian J Org Chem.

[546] Fang Z, He W, Tu T, Lv N, Qiu C, Li X, Zhu N, Wan L, Guo K (2018). Chem Eng J.

[547] Ji P, Feng X, Oliveres P, Li Z, Murakami A, Wang C, Lin W (2019). J Am Chem Soc.

[548] Kang Q, Zhao Z-A, You S-L (2007). J Am Chem Soc.

[549] Osorio-Planes L, Rodríguez-Escrich C, Pericàs M A (2014). Chem – Eur J.

[550] Rodríguez‐Rodríguez M, Maestro A, Andrés J M, Pedrosa R (2020). Adv Synth Catal.

[551] Bahrmann H, Cornils B, Diekhaus G, Kascha W, Weber J (1980). Verfahren Zur Herstellung von Aldehyden. Ger. Pat. Appl..

[552] Frisch A C, Webb P B, Zhao G, Muldoon M J, Pogorzelec P J, Cole-Hamilton D J (2007). Dalton Trans.

[553] Webb P B, Sellin M F, Kunene T E, Williamson S, Slawin A M Z, Cole-Hamilton D J (2003). J Am Chem Soc.

[554] Burguete M I, García-Verdugo E, Luis S V (2011). Beilstein J Org Chem.

[555] Wolf P, Logemann M, Schörner M, Keller L, Haumann M, Wessling M (2019). RSC Adv.

[556] Kasinathan S, Bourne S L, Tolstoy P, Koos P, O’Brien M, Bates R W, Baxendale I R, Ley S V (2011). Synlett.

[557] Masui H, Honda E, Niitsu S, Shoji M, Takahashi T (2018). Int J Org Chem.

[558] Keybl J, Jensen K F (2011). Ind Eng Chem Res.

[559] Zhu C, Raghuvanshi K, Coley C W, Mason D, Rodgers J, Janka M E, Abolhasani M (2018). Chem Commun.

[560] Raghuvanshi K, Zhu C, Ramezani M, Menegatti S, Santiso E E, Mason D, Rodgers J, Janka M E, Abolhasani M (2020). ACS Catal.

[561] Kaiser N M, Jokiel M, McBride K, Flassig R J, Sundmacher K (2017). Ind Eng Chem Res.

[562] Jokiel M, Kaiser N M, Kováts P, Mansour M, Zähringer K, Nigam K D P, Sundmacher K (2019). Chem Eng J.

[563] Wang S, Zhang J, Peng F, Tang Z, Sun Y (2020). Ind Eng Chem Res.

[564] Watkins A L, Landis C R (2010). J Am Chem Soc.

[565] Cunillera A, Blanco C, Gual A, Marinkovic J M, Garcia‐Suarez E J, Riisager A, Claver C, Ruiz A, Godard C (2019). ChemCatChem.

[566] Gutmann R, Gscheidmeier M, Wiesmüller J, Riedel A (1997). Process for the Contiunuous Preparation of Terpene Esters. U.S. Patent.

[567] Zheng H, Yan Z, Chu S, Chen J (2018). Chem Eng Process.

[568] Adarme C A A, Leão R A C, de Souza S P, Itabaiana I, de Souza R O M A, Rezende C M (2018). Mol Catal.

[569] Okuno Y, Isomura S, Kamakura T, Sano F, Tamahori K, Goto T, Hayashida T, Kitagawa Y, Fukuhara A, Takeda K (2015). ChemSusChem.

[570] He P, Haswell S J, Fletcher P D I, Kelly S M, Mansfield A (2012). J Flow Chem.

[571] Baumann M, Baxendale I R, Filipponi P, Hu T (2017). Org Process Res Dev.

[572] Li X, Chen A, Zhou Y, Huang L, Fang Z, Gan H, Guo K (2015). J Flow Chem.

[573] Csajági C, Szatzker G, Rita Tőke E, Ürge L, Darvas F, Poppe L (2008). Tetrahedron: Asymmetry.

[574] Calmanti R, Galvan M, Amadio E, Perosa A, Selva M (2018). ACS Sustainable Chem Eng.

[575] Contente M L, Farris S, Tamborini L, Molinari F, Paradisi F (2019). Green Chem.

[576] Battilocchio C, Deadman B J, Nikbin N, Kitching M O, Baxendale I R, Ley S V (2013). Chem – Eur J.

[577] Trnka T M, Grubbs R H (2001). Acc Chem Res.

[578] Mol J C (2004). J Mol Catal A: Chem.

[579] Sinclair F, Alkattan M, Prunet J, Shaver M P (2017). Polym Chem.

[580] Hughes D, Wheeler P, Ene D (2017). Org Process Res Dev.

[581] Yu M, Lou S, Gonzalez-Bobes F (2018). Org Process Res Dev.

[582] Grela K, Harutyunyan S, Michrowska A (2002). Angew Chem, Int Ed.

[583] Kadyrov R (2013). Chem – Eur J.

[584] Gawin R, Tracz A, Chwalba M, Kozakiewicz A, Trzaskowski B, Skowerski K (2017). ACS Catal.

[585] Dumas A, Colombel-Rouen S, Curbet I, Forcher G, Tripoteau F, Caijo F, Queval P, Rouen M, Baslé O, Mauduit M (2019). Catal Sci Technol.

[586] Dörrich S, Ulmer A, Mahler C, Burschka C, Baus J A, Tacke R, Chai A, Ding C, Zou Y, Brunner G (2014). Eur J Inorg Chem.

[587] Lovchik M A, Kraft P (2017). Chem – Eur J.

[588] Williams A S (1999). Synthesis.

[589] Wiegers W J, Van Loveren A G, Hanna M R, Luccarelli D, Bowen D R, Vock M H (1984). Macrocyclic Carbonates, Processes for Preparing Same, Organoleptic Uses Thereof and Intermediates Used in Said Process. U.S. Pat. Appl..

[590] Tsuji J, Hashiguchi S (1980). Tetrahedron Lett.

[591] Kraft P, Berthold C (2008). Synthesis.

[592] Lehr K, Fürstner A (2012). Tetrahedron.

[593] Narula A P S, Arruda E M, Amorelli B, Schiet F T (2013). 3-Methyl-6-Cyclohexadecen-1-One and Its Use in Perfume Compositions. U.S. Patent.

[594] Shen J, Shi Y, Tian W (2015). Chin J Chem.

[595] Callejo R, Corr M J, Yang M, Wang M, Cordes D B, Slawin A M Z, O'Hagan D (2016). Chem – Eur J.

[596] Sytniczuk A, Leszczyńska A, Kajetanowicz A, Grela K (2018). ChemSusChem.

[597] Reckziegel A, Wohrle I, Sonnenberg S (2013). (Z)-7-Cyclohexadecen-1-One as an Odorant. U.S. Pat. Appl..

[598] Michrowska A, Wawrzyniak P, Grela K (2004). Eur J Org Chem.

[599] Mathys M, Kraft P (2014). Chem Biodiversity.

[600] Comer E, Organ M G (2005). J Am Chem Soc.

[601] Ahmed-Omer B, Barrow D A, Wirth T (2011). ARKIVOC.

[602] Drop M, Bantreil X, Grychowska K, Mahoro G U, Colacino E, Pawłowski M, Martinez J, Subra G, Zajdel P, Lamaty F (2017). Green Chem.

[603] Alexander K A, Paulhus E A, Lazarus G M L, Leadbeater N E (2016). J Organomet Chem.

[604] Morin É, Sosoe J, Raymond M, Amorelli B, Boden R M, Collins S K (2019). Org Process Res Dev.

[605] Michrowska A, Mennecke K, Kunz U, Kirschning A, Grela K (2006). J Am Chem Soc.

[606] Cabrera J, Padilla R, Bru M, Lindner R, Kageyama T, Wilckens K, Balof S L, Schanz H-J, Dehn R, Teles J H (2012). Chem – Eur J.

[607] Bru M, Dehn R, Teles J H, Deuerlein S, Danz M, Müller I B, Limbach M (2013). Chem – Eur J.

[608] Solodenko W, Doppiu A, Frankfurter R, Vogt C, Kirschning A (2013). Aust J Chem.

[609] Balcar H, Žilková N, Kubů M, Polášek M, Zedník J (2018). Catal Today.

[610] Lim J, Seong Lee S, Ying J Y (2010). Chem Commun.

[611] Riva E, Rencurosi A, Gagliardi S, Passarella D, Martinelli M (2011). Chem – Eur J.

[612] Gmeiner J, Seibicke M, Lang C, Gärtner U, Trapp O (2014). Adv Synth Catal.

[613] Borré E, Rouen M, Laurent I, Magrez M, Caijo F, Crévisy C, Solodenko W, Toupet L, Frankfurter R, Vogt C (2012). Chem – Eur J.

[614] Selva M, Guidi S, Perosa A, Signoretto M, Licence P, Maschmeyer T (2012). Green Chem.

[615] Duque R, Öchsner E, Clavier H, Caijo F, Nolan S P, Mauduit M, Cole-Hamilton D J (2011). Green Chem.

[616] Schoeps D, Buhr K, Dijkstra M, Ebert K, Plenio H (2009). Chem – Eur J.

[617] Rabiller-Baudry M, Nasser G, Renouard T, Delaunay D, Camus M (2013). Sep Purif Technol.

[618] Ormerod D, Bongers B, Porto-Carrero W, Giegas S, Vijt G, Lefevre N, Lauwers D, Brusten W, Buekenhoudt A (2013). RSC Adv.

[619] O'Neal E J, Jensen K F (2014). ChemCatChem.

[620] Monfette S, Eyholzer M, Roberge D M, Fogg D E (2010). Chem – Eur J.

[621] Skowerski K, Czarnocki S J, Knapkiewicz P (2014). ChemSusChem.

[622] Ley S V, Ingham R J, O’Brien M, Browne D L (2013). Beilstein J Org Chem.

[623] Kreutz J E, Shukhaev A, Du W, Druskin S, Daugulis O, Ismagilov R F (2010). J Am Chem Soc.

[624] Rogers L, Jensen K F (2019). Green Chem.

[625] Hughes D L (2018). Org Process Res Dev.

[626] Testa C J, Hu C, Shvedova K, Wu W, Sayin R, Casati F, Halkude B S, Hermant P, Shen D E, Ramnath A (2020). Org Process Res Dev.

